# Integrated Metabolome and Transcriptome Analysis Reveals a Potential Mechanism for Water Accumulation Mediated Translucency in Pineapple (*Ananas comosus* (L.) Merr.) Fruit

**DOI:** 10.3390/ijms24087199

**Published:** 2023-04-13

**Authors:** Jing Chen, Yanli Yao, Hui Zeng, Xiumei Zhang

**Affiliations:** 1 The South Subtropical Crops Research Institute of Chinese Academy of Tropical Agricultural Sciences, Zhanjiang 524091, China; 2 Key Laboratory of Tropical Fruit Tree Biology, Ministry of Agriculture, Zhanjiang 524091, China

**Keywords:** pineapple, translucency, flavonoid, transcriptome, metabolome

## Abstract

A physiological disease of the pineapple fruit called pineapple translucency causes the pulp to become water-soaked, which affects the fruit’s taste, flavor, shelf life, and integrity. In the present study, we analyzed seven pineapple varieties, of which three were watery and four were non-watery. There were no apparent macronutritional (K, P, or N) differences in their pulp, but the non-watery pineapple varieties had higher dry matter and soluble sugar content. The metabolomic analysis found 641 metabolites and revealed differential expression of alkaloids, phenolic acids, nucleotide derivatives, lipids, and other metabolites among the seven species. Transcriptome analysis and further KEGG enrichment showed downregulation of ‘flavonoid biosynthesis’ pathways, differential expression of metabolic pathways, secondary metabolites biosynthesis, plant–pathogen interaction, and plant hormone signal transduction. We believe this study will provide critical molecular data supporting a deeper understanding of pineapple translucency formation and greatly benefit future research on this commercially important crop.

## 1. Introduction

The world-renowned fruit pineapple (*Ananas comosus* L. Merr.) is widely planted in more than 80 countries and regions between the South and the Tropic of Cancer. It is one of the major crops in tropical and subtropical countries [1] and the third most important tropical fruit in China, utilizing 70,000 hectares of land [2]. In 2019, China ranked 5th in pineapple production, with an annual harvest of 1727 thousand metric tons [3]. Pineapple translucency (also known as water core) has been a recurring problem in marketing of the fruits [4]. Since the inception of a special research project in 2006 by the Ministry of Agriculture of China, pineapple fruit quality improvement has been a point of research interest [5]. The translucent pineapple flash has a drenched appearance [6] because the intercellular spaces are filled with water [7]. This makes the translucent fruits delicate and vulnerable to mechanical damage during harvest and postharvest handling [8]. Damaged pineapples remain wet after harvesting and leak fluid, causing the damaged area to become wet and prone to fungal diseases [9]. They also have poor test and flavor. Pineapple fruit translucency affects approximately 10% of fresh fruit, and losses can exceed 30% [10].

Pineapple translucency is closely related to the accumulation and deficiency of various nutrients and metabolites, including calcium deficiency; K, P, and N accumulation; and other environmental factors, such as crown size and fruit temperature [9,11]. A key element of pineapple quality is its sugar content [8]. Chen and Paull [12] found a quick spike in pineapple fruits sucrose and fructose content at four weeks before harvest. Murai, Chen, and Paull [4] proposed that the pineapple crown decreases translucency by providing shade or changes in fruit water relations, altered sugar unloading, and cellular uptake. These revealed a connection between fruit sugar buildup and pineapple translucency. An increased apoplastic solute concentration and water passage into the apoplast resulted in increased sugar accumulation and may result in pineapple translucency [12]. However, translucency may also be a result of heat or cultivar difference. The primary mechanism of pineapple translucency is yet to be revealed.

Currently, the researchers focused mainly on evaluating fruit texture, regulation of fruit texture, reproduction and breeding, functional activity, physiology and biochemistry, diversity analysis, and development of improved variety for pineapple. With the development of molecular biology and sequencing technology, transcriptome technology could open up new possibilities for discovering the genes responsible for pineapple transparency [13]. Transcriptome sequencing helps find new transcripts and examine gene expression [14]. RNA-seq or transcriptomic analysis has been used extensively to study many plant species, but for pineapples, limited transcriptome data exist [13]. Through RNA-seq technology, a large amount of transcriptome sequence information can be generated and manipulated to evaluate gene expression, function, and related metabolic pathways.

Herein, illumina paired-end sequencing and label-free analysis of the pulp from watery pineapple and the non-watery varieties were conducted to understand the genetic mechanism of pineapple translucency. We listed both variations’ differentially expressed genes and annotated their function. We further performed the metabolome evaluation using translucent and non-translucent pineapples. Then, we compared metabolome profiles among different varieties, revealed the differentially accumulated metabolites in translucent pineapples, and annotated the highly enriched molecular pathways and gene ontological terms.

We hope this comprehensive metabolome and transcriptome study will substantially improve the understanding of the potential molecular mechanisms of translucency in pineapple fruits and pave the way for further analysis. This study will not only provide important molecular data supporting a deeper understanding of translucent growth but also greatly benefit the improvement of future research on this commercially important crop.

## 2. Results

We investigated the watery and non-watery pineapple pulp to understand the basis of water accumulation in the fruit during maturity. Results from the nutritional values, accumulation level of various metabolites, and gene differential expression are as follows.

### 2.1. Morphological and Nutritional Estimates of the Watery and Non-Watery Pineapples 

**Figure 1:**
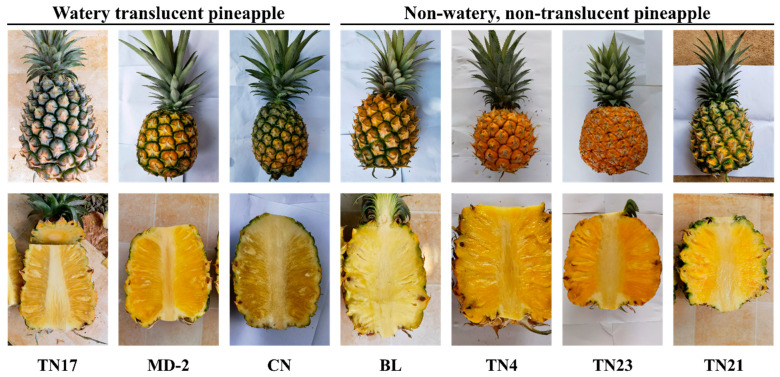
Different varieties of watery and non-watery pineapples with vertical cross sections. From left to right, watery translucent pineapple varieties are Golden diamond or Tainong 17 (TN17), Golden pineapple (MD-2), and Caine or Smooth cayenne (CN), which accumulate water in mature fruits. In contrast, non-watery, non-translucent varieties Bali or Comte-de-Paris (BL), Shredded pineapple or Tainong 4 (TN4), Mango pineapple or Tainong 23 (TN23), and Golden pineapple or Tainong 21 (TN21) retain less water during fruit maturity.

Pineapple fruits with watery pulp tend to have reduced fruit compactness and soluble sugars while accumulating dry matter. Compared with the non-watery pineapples, the watery pineapples have significantly less fruit physiology and morphology (Figure 1). The watery pineapple showed increased darkness of fruit pulp color and dry matter content with a considerably deteriorated quality. In contrast, dry matter contents and soluble sugar were substantially higher in non-watery pineapple (Figure 1). However, the two groups we studied had no significant difference in macronutrient accumulation (Table 1). Overall, the study showed the profound effects of water accumulation in pineapple fruit on quality and dry matter accumulation (Table 1).

### 2.2. Metabolite Profiling between the Watery and Non-Watery Pineapples in Low Nitrogen (LN) Stress Conditions

To understand the difference in the level of the metabolites between the watery and non-watery pineapples, metabolome profiles of the fruit pulp were analyzed using the UPLC-ESI-MS/MS system. We detected 641 metabolites in pineapple pulp, belonging to 9 major and 27 subclasses, as summarized in Table 2 and Table S1. Among the 10 major metabolites classes, 47 were flavonoids, followed by 101 lipids, 126 phenolic acids, 83 amino acid derivatives, 48 nucleotides and derivatives, 51 organic acids, 57 alkaloids, 22 lignans and coumarins, 3 terpenoids, and 103 various other metabolites.

**Figure 2:**
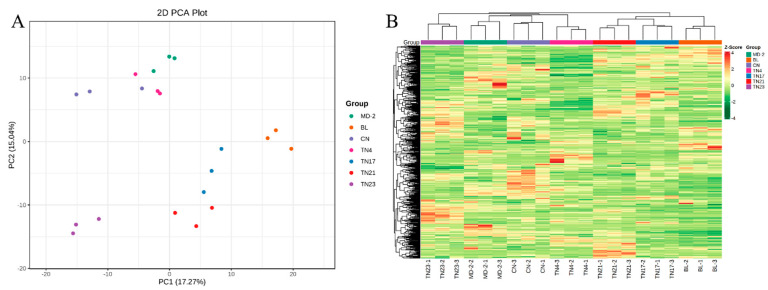
Metabolite profiling of the watery and non-watery pineapples. (**A**) Principal component analysis using UPLC-ESI-MS/MS data. (**B**) Clustered heatmap of 641 metabolites among the pineapple samples. The cluster lines on the left represent the metabolite clusters. The cluster lines at the top represent sample clusters. Red and green indicate high and low metabolite contents, respectively.

The general result from water accumulation on the metabolite profile pineapple pulp was first tested by principal component analysis (PCA). The dispersion between quality control (QC) specimens showed that the metabolic analysis instrument had stable and reliable data detection and could, thus, be used for subsequent analysis. A scatter plot was drawn on the basis of PCA scores to compare the sample distribution pattern. There was an apparent separation between samples within the watery and non-watery pineapples (Figure 2). However, the watery pineapple TN17 also showed relevance to non-watery pineapple TN23. The findings demonstrated how reliable metabolite identification and metabolomics analysis are. The first principal component (PC1) explained 17.27% of the overall variance in leaf metabolome between samples, which distinguished the watery and non-watery pineapples (Figure 2), indicating that the water accumulation had a substantial effect on metabolite concentration in pineapple fruit. The heatmap-based cluster analysis also revealed a clear cluster of watery pineapple MD-2 and CN (SC), while the TN17 resembled TN23. PC2 showed variation of TN23 from other non-watery pineapples, with 13.27% of total variation among samples (Figure 2). The closer clustering of replicates in each biological replicate revealed the higher sampled quality, while the distant neighbor showed relatively higher variation among samples.

### 2.3. Differentially Accumulated Metabolites under Low Nitrogen (LN) Treatment

Orthogonal partial least squares discriminant analysis (OPLS-DA) was performed on the metabolic profiles to identify metabolites affected by water accumulation in pineapple fruit. Significant differentially accumulated metabolites (DAM) were selected with variable importance for projection (VIP) > 1. A total of 513 DAMs (80.03% of total metabolites) affected by water accumulation were detected in non-watery pineapple pulp (Table S1).

When compared with the BL, 248, 232, 193, 222, and 262 DAMs were obtained from CN, TN4, TN17, TN21, and TN23, respectively. Among these metabolites, 172, 129, 120, 137, and 175 were up-accumulated, while 76, 103, 73, 85, and 87 metabolites were down-accumulated at CN, TN4, TN17, TN21, and TN23, respectively. Compared with the CN, 210, 198, 221, and 187 DAMs were obtained from TN4, TN17, TN21, and TN23, respectively. Among these metabolites, 71, 93, 102, and 87 were up-accumulated, while 139, 105, 119, and 100 were down-accumulated at TN4, TN17, TN21, and TN23, respectively. Compared with the MD-2, 228, 169, 204, 206, 193, and 217 DAMs were obtained from BL, CN, TN4, TN17, TN21, and TN23, respectively. Among these metabolites, 84, 87, 76, 91, 82, and 107 were up-accumulated, while 144, 82, 128, 115, 111, and 110 were down-accumulated at BL, CN, TN4, TN17, TN21, and TN23, respectively. Compared with the TN4, 229, 222, and 227 DAMs were obtained from TN17, TN21, and TN23, respectively. Among these metabolites, 131, 141, and 139 were up-accumulated, while 98, 81, and 88 were down-accumulated at TN17, TN21, and TN23, respectively. Compared with the TN17, 160 and 206 DAMs were obtained from TN21, and TN23, respectively. Among these metabolites, 89 and 120 were up-accumulated, while 71 and 86 were down-accumulated at TN21, and TN23, respectively. Compared with the TN21, 173 DAMs were obtained from TN23, where 99 were up-accumulated and 74 were down-accumulated. 

**Figure 3:**
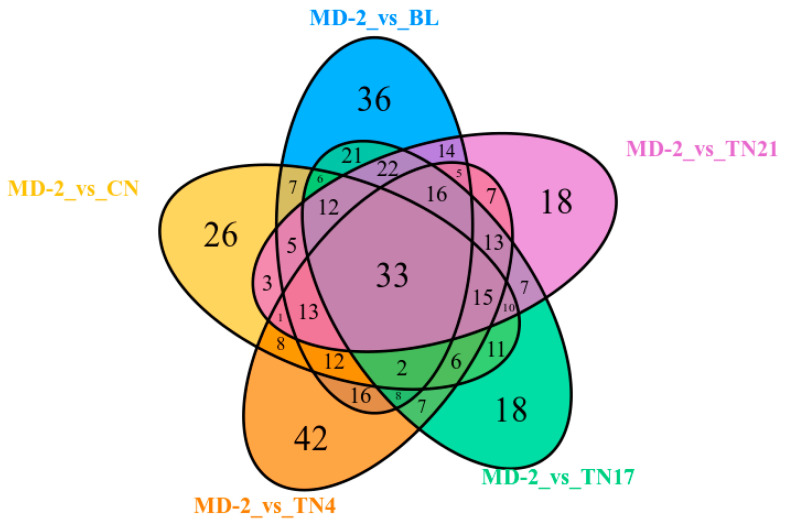
Wayne diagram of comparative analysis among samples for DAMs. Each circle represents a comparison group; circle numbers and overlapping parts represent the number of differential metabolites common among the comparison groups, and the numbers without overlap represent the numbers of differential metabolites specific to the comparison group.

When BL, TN21, TN17, TN4, and CN groups were compared with MD-2 using a Wayne diagram, among the total 420 DAMs, 33 were accumulated in all samples, representing the core metabolome responsive to LN. In addition, 58 were differentially accumulated among 4 of the 5 samples, while 88 were differentially accumulated among 3 of the 5 samples, and 101 were differentially accumulated among 2 samples of the 5 samples. The remaining 140 DAMs were specially accumulated in BL, TN21, TN17, TN4, and CN, with 36, 18, 18, and 42 DAMs in each case (Figure 3).

Among the DAM, most of them (22.03%) were phenolic acids, followed by amino acids and their derivatives (14.23%), lipids (12.67%), alkaloids (8.97%), nucleotides and derivatives (8.97%), flavonoids (8.38%), organic acids (7.41%), lignans and coumarins (4.29%), terpenoids (0.39%), and others (12.67%). Among the core, 33 DAMs conserved responsive to translucency (Table S1), and 15 metabolites, including 6 alkaloids, 3 phenolic acids, 2 nucleotide derivatives, 1 lipid, and 3 other compounds, were down-accumulated. In contrast, the remaining seven metabolites were up-accumulated in response to the LN stress.

**Figure 4:**
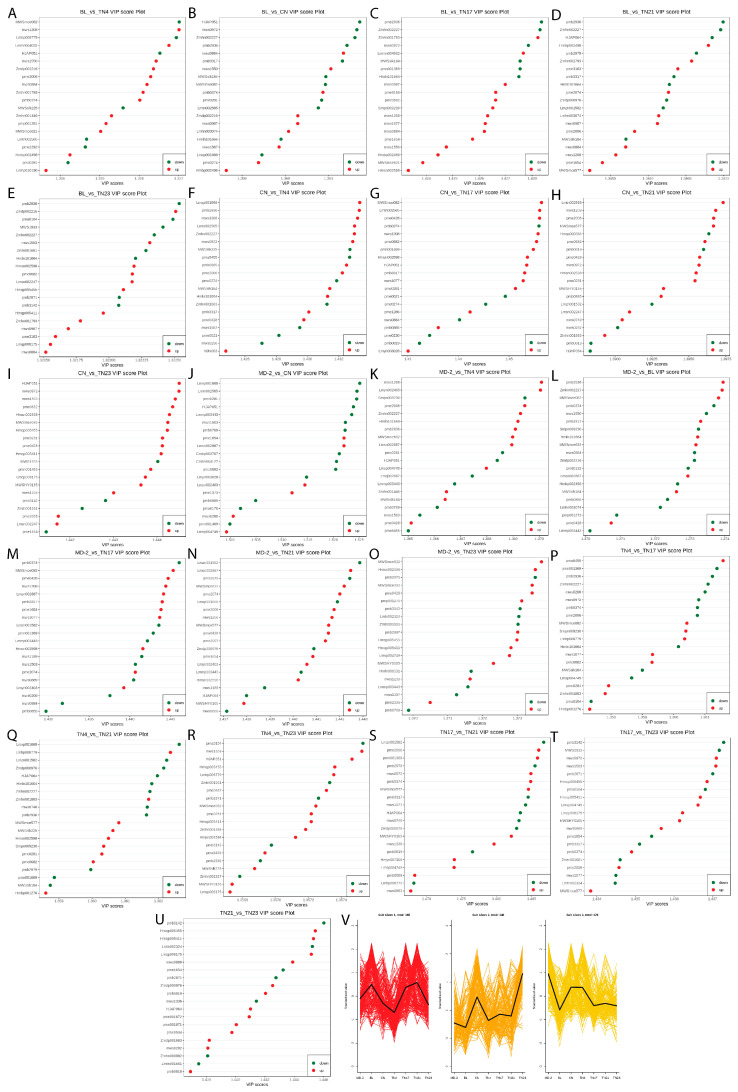
Variable importance for projection (VIP) scores analysis based on the weighted coefficients of the OPLS–DA for the differential metabolites identified on the basis of the screening criteria of each group comparison; the top 20 metabolites with the most significant VIP values of the OPLS–DA model was selected for display (**A**–**U**). The K-means cluster analysis of the metabolites of seven pineapples pulps revealed three subclasses (**V**).

For the differential metabolites identified on the basis of the screening criteria of each group comparison, the top 20 metabolites with the largest variable importance of projection (VIP) score of the OPLS-DA model were selected to rank the contribution of metabolites to the discrimination among different species (Figure 4). Phenolic acids, Disinapoyl glucoside had highest VIP in BL vs. TN17 (1.43), BL vs. TN21 (1.35), BL vs. TN23 (1.32), MD-2 vs. BL (1.37), and TN4 vs. TN23 (1.36); 3-(3,4,5-Trimethoxyphenyl)propan-1-ol in BL vs. CN (1.36) and CN vs. TN23 (1.44); 2-Methoxy-4-ethenylphenol in CN vs. TN4 (1.43) and MD-2 vs. CN (1.53); 1-O-Vanilloyl-D-Glucose in CN vs. TN21 (1.40); 5’-Glucosyloxyjasmanic acid in MD-2 vs. TN21 (1.44) and TN17 vs TN21 (1.49); Vanillin acetate in MD-2 vs. TN23 (1.37); and Salicylic acid-2-O-glucoside in TN17 vs. TN23 (1.44). Among other metabolites, 1,10-Decanediol had the highest VIP in MD-2 vs. TN4 (1.37); 2-Aminopurine in MD-2 vs. TN17 (1.45); Ribulose-5-phosphate in TN4 vs. TN17 (1.36); and Scoparone in BL vs. TN4 (1.33) and CN vs. TN17 (1.46).

### 2.4. KEGG Pathway Enrichment Study

To further understand the functions of the differentially accumulated metabolites and the related biological processes in which they participate, pathway enrichment study of DAMs was conducted using the KEGG database. The list of pathways that correspond to the experimental dataset’s largest amount of metabolites is included in the Supplemental Materials (Table S2).

The DAMs in BL_vs_CN, BL_vs_TN17, BL_vs_TN21, BL_vs_TN23, BL_vs_TN4, CN_vs_TN17, CN_vs_TN21, CN_vs_TN23, CN_vs_TN4, MD-2_vs_BL, MD-2_vs_CN, MD-2_vs_TN17, MD-2_vs_TN21, MD-2_vs_TN23, MD-2_vs_TN4, TN17_vs_TN21, TN17_vs_TN23, TN21_vs_TN23, TN4_vs_TN17, TN4_vs_TN21, and TN4_vs_TN23 were annotated in 77, 51, 58, 69, 64, 64, 64, 60, 78, 69, 70, 71, 70, 71, 71, 44, 60, 56, 72, 70, and 74 KEGG pathways, respectively. Overall, 89 pathways were discovered by pairwise comparison among the sample groups, of which 19 were involved regardless of compared samples. Among the annotated pathways, ‘Metabolic pathways’ was observed to be enriched with a maximum metabolite frequency of 83.47% and significance of differentially accumulated metabolites (Table S2, Figure S1). Generally, metabolites involved in these pathways were up-accumulated (Figure S2, Table S2).

We also evaluated the rich factor (RF) among different pathways, the ratio of the number of differentially accumulated metabolites in the corresponding pathway to the total number of metabolites detected by the path, and extracted the highest 20 enriched pathways. Among them, ‘Anthocyanin biosynthesis’ showed the highest enrichment score (Figure S2). 

### 2.5. Transcriptome Assembly for Pineapple Samples

For a comprehensive insight into the genes related to the development of the watery trait in pineapple, samples of both watery and non-watery species were collected (Figure 1). The cDNA libraries were constructed from three biological repeats. The high-throughput sequencing (Illumina hiseq 4000 platform) data were generated and then transformed into the raw data by base calling analysis. A maximum of 49.02, 48.86, 47.28, 52.21, 49.01, 48.72, and 48.03 million raw reads were extracted for BL, CN, MD-2, TN17, TN21, TN23, and TN4, respectively (Table 3). After cleaning the reads, maximums of 46.51, 45.16, 42.4, 49.77, 46.71, 45.42, and 46.62 million bases with 49.88%, 50.19%, 50.05%, 48.09%, 50.71%, 50.83%, and 48.4% GC contents; 97.46%, 97.45%, 98.06%, 97.61%, 97.42%, 97.57%, and 97.43% Q > 20; and 93.12%, 93.18%, 94.6%, 93.45%, 93.08%, 93.49%, and 93.05% Q > 30 were retained for BL, CN, MD-2, TN17, TN21, TN23, and TN4, respectively (Table 3). The assembly of clean reads provided 24,515 unigenes with an average length of 5131.11 bp (Table 3). All the screened unigenes were larger than 300 bp size, while 87.69% of unigenes (21,847) displayed extra-long sizes (>1000 bp) (Table 4). The superior expression quality (ExN50) of constructed contigs (N50) was shown by the most of the contigs (>1510 bp) (Table 4).

### 2.6. Total and Differential Gene Expression Analysis

**Figure 5:**
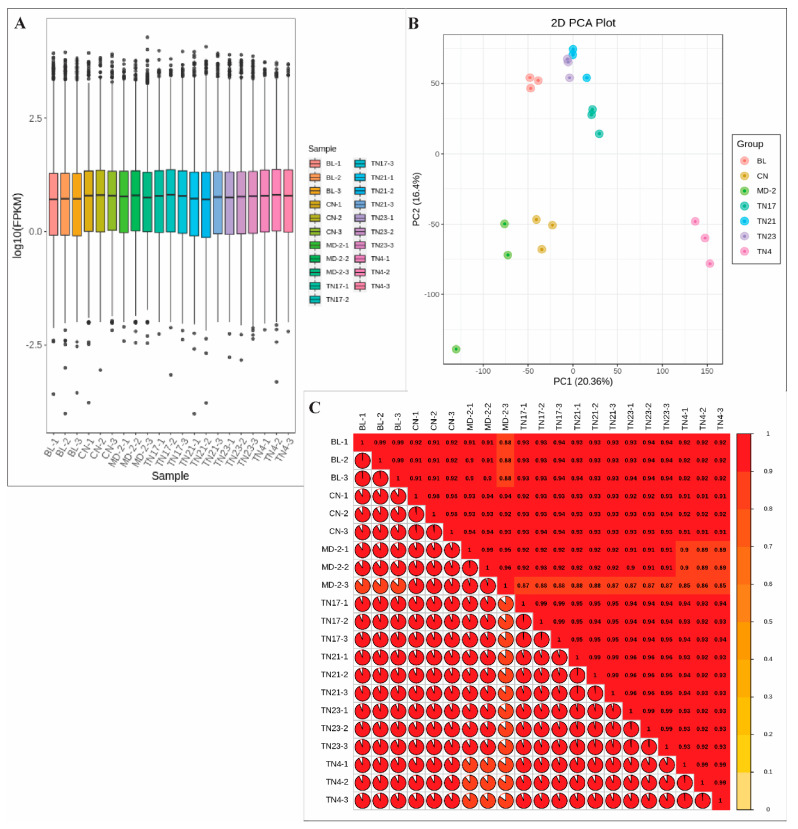
Relation among three replicates of the seven pineapple species shown by expression evaluation of FPKM values (**A**), the principal component analysis (**B**), and correlation between samples (**C**). Three replicates are indicated by numeric values 1 to 3 for seven species preceded by a hyphen; the number in each box in (**C**) is the value of Pearson’s coefficient of correlation, while the color scale indicates its significance from 0 to 1.

The total gene expression was higher in non-watery species, as observed by FPKM values (Figure 5A). The principal component analysis (PCA) indicated the close relation of samples within species but a relatively high distance between samples from the two species (Figure 5B). This was supported by the findings of the average Pearson’s coefficient of correlation (Figure 5C), which indicated extensive genetic dissimilarities between the two species.

**Figure 6:**
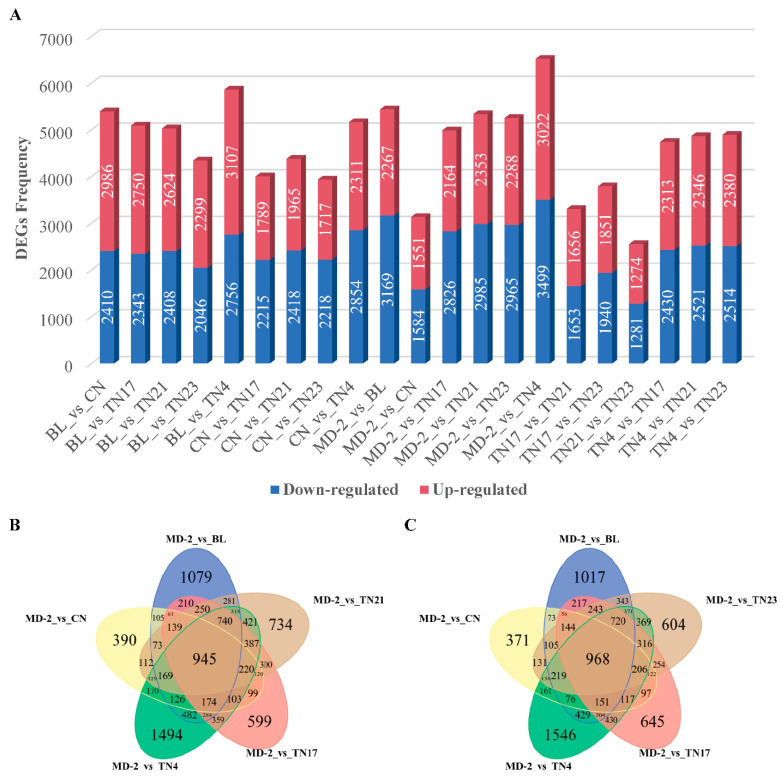
Summary of differentially expressed genes among seven pineapple species. (**A**) The DEGs among seven pineapple species and (**B**,**C**) the frequency of overlapping and unique DEGs.

The expressed genes were further screened for their differential expression (DEGs) using DESeq2 analysis based on |log2 Fold Change| ≥ 1, and false discovery rate (FDR) < 0.05 (Figure 6A–C). The differential expression analysis among the two species showed 5396, 5093, 5032, 4345, 5863, 4004, 4383, 3935, 5165, 5436, 3135, 4990, 5338, 5253, 6521, 3309, 3791, 2555, 4743, 4867, and 4894 DEGs in BL_vs_CN, BL_vs_TN17, BL_vs_TN21, BL_vs_TN23, BL_vs_TN4, CN_vs_TN17, CN_vs_TN21, CN_vs_TN23, CN_vs_TN4, MD-2_vs_BL, MD-2_vs_CN, MD-2_vs_TN17, MD-2_vs_TN21, MD-2_vs_TN23, MD-2_vs_TN4, TN17_vs_TN21, TN17_vs_TN23, TN21_vs_TN23, TN4_vs_TN17, TN4_vs_TN21, and TN4_vs_TN23 groups, respectively, resulting in a total of 14,426 unique DEGs among the total expressed unigenes (Figure 6B,C; Figure S3 and Table S3). By evaluating all of the DEGs from all pineapple species, we identified 968 (6.71%) core-conserved genes continually differentially expressed among the two species (Figure 6B,C; Table S3). Along with core-conserved DEGs, a high number of specific DEGs (45.20%, 6521) was observed between MD-2_vs_TN4, correlating with the water accumulation in TN4 species. These genes may represent key genes involved in the translucency trait in pineapples.

### 2.7. Functional Annotation and Enrichment Study of DEGs

Genes that were expressed differently were mapped to gene ontology (GO) terms in the GO database [15] to understand better the functions and annotations in the different developmental stages. GO functional enrichment analysis was performed, adjusting the *p*-value of 0.05 as the cutoff (Figure S4). A total of 3270 GO terms were annotated to the 12,032 unigenes hits (Table S4). Among these terms, the maximum 65.66% (2147) GO terms belonged to the class “Biological Processes” (BP) followed by “molecular functions” (MF) (22.39%, 732 terms), and “cellular components” (CC) (11.96%, 391 terms). In CC, the maximum enriched GO terms were ‘Golgi membrane’ with 129 genes, while in MF, the most enriched terms were ‘active transmembrane transporter activity’ with 132 genes. Among the biological processes, ‘response to abscisic acid’ with 159 genes was among the top hits (Figure S5). GO analysis showed 3270 GO terms annotated in 12,032 unigenes, with a descending order of ‘Biological Processes’ (65.66%), ‘molecular functions’ (22.39%), and ‘cellular components’ (11.96%).

The DEGs were further evaluated for their functional enrichment between pairwise comparisons on the basis of the KEGG database [16]. A total of 8554 DEGs between the comparison of seven species was enriched in 140 unique KEGG pathways. Cluster analysis was performed to determine the expression patterns of different genes under different experimental conditions and to identify the function of unknown genes or unknown functions of known genes by clustering genes with the same or similar expression patterns into classes, as these similar genes may have similar functions or participate in the same metabolic process or cellular pathway.

**Figure 7:**
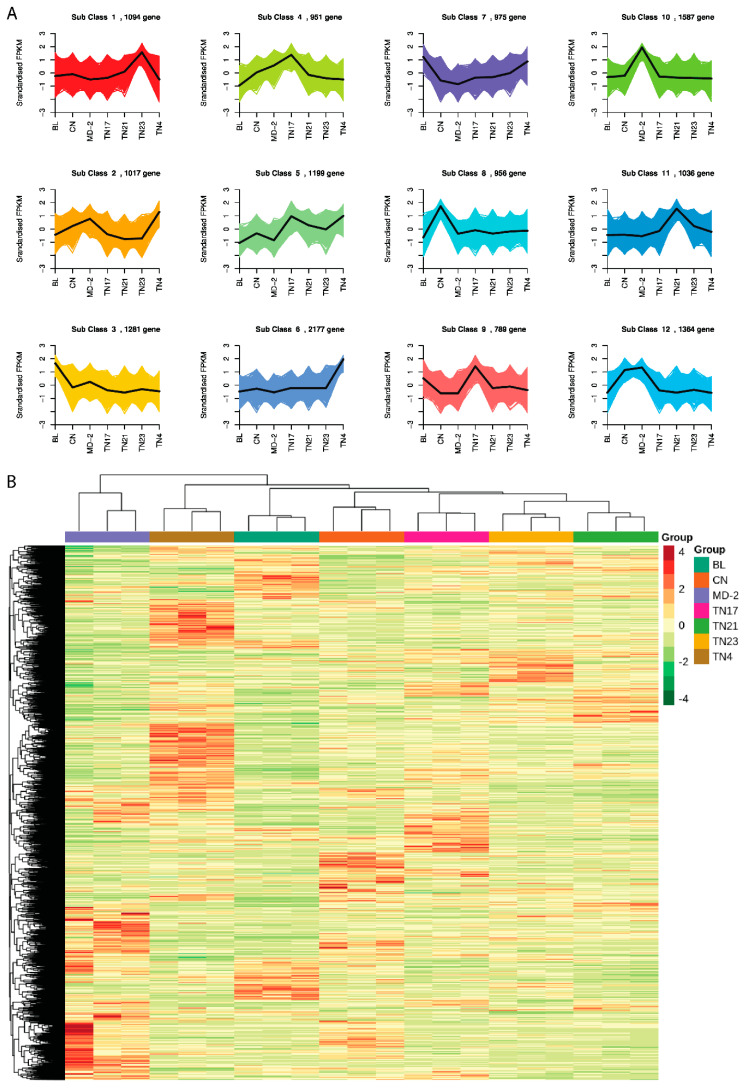
Gene expression clustering. (**A**) K-means cluster plot: the abscissa denotes the sample, and the ordinate denotes the amount of expression centralized and normalized. (**B**) Differential gene clustering heatmap: the abscissa designates the sample name and hierarchical clustering results, and the ordinate indicates the different genes and hierarchical clustering results. Red and green imply high expression and low expression, respectively.

To study the expression pattern of genes under different treatment conditions, the FPKM of the gene was first centralized and normalized, and then K-means clustering was performed. The same class of genes had similar mutation trends under different investigational treatments and may have had similar functions (Figure 7A). The centralized and normalized FPKM expression of differential genes was extracted, hierarchical cluster analysis was performed, and a cluster heatmap was drawn (Figure 7B).

**Figure 8:**
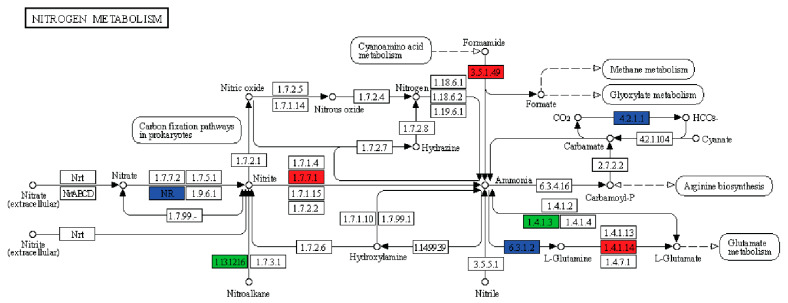
Differentially expressed genes involved in nitrogen assimilation. Differential control of nitrogen metabolism pathway among BL and CN pineapple varieties. Here, red, green, and blue, respectively, represent the upregulated, downregulated, and up/downregulated DEGs in BL compared with CN.

The KEGG pathway annotation results of differentially expressed genes among the 7 pineapple varieties shows that, compared to the watery variety CN, in the non-watery variety BL, three enzymes were upregulated, two were downregulated, and three were both up and downregulated (Figure 8).

The DEGs related to ‘flavonoid biosynthesis’ pathways were downregulated, while the genes involved in ‘Metabolic pathway’ had the most clutter frequency 45.16%, followed by ‘Biosynthesis of secondary metabolites’ (26.57%), ‘Plant–pathogen interaction’ (10.38%), and ‘Plant hormone signal transduction’ (8.50%). Among all 140 annotated pathways, 117 were conserved in all pineapple species (Table S4).

## 3. Discussion

As the third-most significant tropical fruit of China [2], the pineapple requires attention in terms of better nutrition management and identification of possible genes/pathways that may be improved in this respect. Fruit translucency due to water accumulation is a crucial factor that reduces its test and flavor, while making the fruit weak and vulnerable to mechanical damage [8], leading to up to a 30% loss in harvest [10]. In the present study, we examined the metabolomic and transcriptomic comparison among three watery and four non-watery varieties, a total of seven common varieties of pineapple grown in China.

Previous studies indicated that Ca, K, P, and N content and other environmental factors affect pineapple crown size [9,11] and carbon gain [8], which, in turn, influence water accumulation and translucency [12]. However, the tested pineapple species did not significantly differ in N, K, or Na content. This result also coincides with the finding of Chen, Zeng, and Zhang [3]. Shu et al. [17] observed both translucent and non-translucent pineapple pulp have similar total sugar content, but the translucent pulp has higher apoplastic sugar content. So, they hypothesized that when excessive sugar is stored in the pineapple fruit’s intercellular space, water is absorbed from the surrounding cells and forms translucency. However, our investigation found that the non-watery pineapple varieties have higher dry matter and soluble sugar content than their watery counterparts.

Irrespective of changes in sugar content, Yao, Li, Lin, Liu, Wu, Fu, Zhu, Gao, and Zhang [18] reported differential expression of the sugar metabolism genes in translucent pineapple. This is likely due to the difference in the mechanism of translucency development in translucent and non-translucent pineapple varieties. In the current study, 641 metabolites from 9 major and 27 subclasses were detected in pineapple pulp, including lipids, flavonoids, phenolic acids, amino acid derivatives, organic acids, nucleotides and derivatives, alkaloids, lignans and coumarins, terpenoids, and various other metabolites. The PCA analysis revealed that in the PC1 analysis, except for TN17 and TN23, there was an apparent deviation between the watery and non-watery pineapple species (Figure 2A). Clustered heatmap analysis (Figure 2B) revealed a similarity between the metabolic constituents of biological replicates of watery varieties MD-2 and CN. We also saw similarities between the watery variety CN17 and the non-watery variety TN21.

VIP analysis of the differentially accumulated metabolite between watery variety MD-2 and non-watery varieties (BL, TN4, TN23, and TN21) showed accumulation of Jasmonic acid (JA), its derivatives (e.g., 5′-Glucosyloxyjasmanic acid, (−)-Jasmonoyl-L-Isoleucine, and Cis-Jasmone), coumarins, and other phenolic acids (Figure 4K,L,N,O). JA activated by oxidative stress acts as an signaling transducer that leads to accumulation of coumarin in *Cucumis melo* L. [19]. In addition, JA has been associated with pineapple browning [20] and chilling-mediated injuries [21], leading to translucency formation [21]. Differential accumulation of similar compounds was also observed in the VIP score plot of comparisons between watery variety CN and non-watery varieties (Figure 4B,C,F,I) but was opposite in TN17 (Figure 4C,P,S,T). On the other hand, when the non-watery variety TN23 was compared with other non-watery varieties, it showed accumulation of JA and its derivatives (Figure 4E,R,U). PC2 also differentiated the TN23 from other samples, indicating its variation from other non-watery pineapples, which may cause its cluster with TN17 and explain the 13.27% of the total variation among samples (Figure 2A). The closer clustering of replicates in each biological replicate revealed the higher sampled quality, while the distant neighbor showed relatively higher variation among samples. K-means cluster analysis of the differentially accumulated metabolites showed TN21 had the highest higher standardized intensities of compounds in Subclass 1 (185), most of which were phenolic acids (43), amino acids and derivatives (31), and nucleotides and derivatives (20) (Figure 4V). TN23 was the highest in Subclass 2 (148), comprising mostly amino acids and derivatives (28) and phenolic acids (22) (Figure 4V). The watery variety MD-2, however, had the highest higher standardized intensities in Subclass 3 (178), which mostly consisted of phenolic acids (46) nucleotides and derivatives (21) (Figure 4V).

During the DAM analysis, a total of 513 DAMs (80.03%) were found from the comparison among seven pineapple species, of which, in descending order of numbers, were amino acids and derivatives, phenolic acids, alkaloids, lipids, nucleotides and derivatives, flavonoids, organic acids, lignans and coumarins, terpenoids, and others. Among the core, 33 DAMs conserved responsive to translucency (Table S1), and 15 metabolites, including 6 alkaloids, 3 phenolic acids, 2 nucleotide derivatives, 1 lipid, and 3 other compounds, were down-accumulated, while the remaining 7 metabolites were up-accumulated. Enrichment of DAMs indicates amino acids accumulation directly resulted from translucency formation. Similar metabolomic change was reported by Luengwilai et al. [22] in postharvest browned pineapples. Accumulation of amino acids and organic acids was reported to occur in stored freeze-stress-tolerant pineapple in response to freezing [22]. These changes in metabolite concentration are likely how non-watery pineapple variety resist translucency formation. Higher activity of polyphenol peroxidase is responsible for converting cold-stressed-induced phenolics to melatonin in the browned pineapple [22]. Metabolites with the top VIP scores were primarily phenolic acids, indicating their accumulation due to low polyphenol activity in the non-watery variety. This metabolomic analysis distinguished metabolite composition between watery and non-watery pineapples.

In addition, in the KEGG enrichment analysis, 89 pathways were discovered by pairwise comparison, among which 19 were ubiquitous. Metabolic pathways had a maximum metabolite frequency of 83.47% and significance of differentially accumulated metabolites. Activity of cell wall invertase (CWI), responsible for sugar metabolism, was correlated with pineapple flash translucency [12]. We also found accumulation of pyruvate invertase in watery and putative invertase inhibitors in the non-watery varieties (Table S3) and upregulation of sugar metabolism pathways in the KEGG enrichment analysis (Table S2).

During transcriptome analysis, 24,515 unigenes (average length 5131.11 bp) were reported, which showed a close cluster between replicates and a high difference between species in PCA and average Pearson’s coefficient of correlation. DEG analysis of the unigenes revealed 14,426 unique DEGs, of which 968 (6.71%) were core-conserved genes differentially expressed among all species. In addition, a high number of specific DEGs (45.20%, 6521) was observed in MD-2_vs_TN4, correlating with the water accumulation in TN4 species. DEGs upregulation in these pathways was responsible for water accumulation. These correlations between transcriptome and pineapple translucency were further investigated to identify responsible molecular pathways.

The KEGG enrichment analysis showed 140 unique KEGG pathways from 8554 DEGs during comparison of the 7 species. We found that the ‘flavonoid biosynthesis’ pathways related DEGs were downregulated. In contrast, the ‘Metabolic pathway’ had the most clutter frequency 45.16%, followed by ‘Biosynthesis of secondary metabolites’ (26.57%), ‘Plant–pathogen interaction’ (10.38%), and ‘Plant hormone signal transduction’ (8.50%). Among all 140 annotated pathways, 117 were conserved in all pineapple species (Table S4). Shu, Wang, Li, He, Ding, Zhan, and Chang [17] reported that the translucent pineapple flash had a higher expression of 250 proteins, namely calcium–ion-binding protein, EF-hand domain-containing protein, ethylene-synthesizing enzyme 1-aminpcyclopropane-1-carboxylate oxidase, and ROS-producing protein universal stress protein. Yao, Li, Lin, Liu, Wu, Fu, Zhu, Gao, and Zhang [18] reported a total of 38 differentially expressed transcription factors, among which WRKY was the most abundant, followed by MYB. In our investigation, enrichment of KEGG pathways ‘Biological Processes’ and ‘active transmembrane transporter activity’ was likely due to high CWI activity leading to enhanced apoplectic sugar unloading [12] and eventual water movement into intercellular space creating translucency effect.

## 4. Materials and Methods

### 4.1. Plant Material and Sampling

Seven pineapple (*Ananas comosus* (L.) Merr) cultivars were obtained from Zhejiang University, Zhejiang, China, to conduct this research. Among them, three varieties, Golden Pineapple (MD-2), Caine or Smooth cayenne (CN), and Golden diamond or Tainong-17 (TN17), accumulate water in mature fruits. In contrast, the other four, namely Bali or Comte-de-Paris (BL), Shredded pineapple or Tainong 4 (TN4), Golden pineapple or Tainong-21(TN21), and Mango pineapple or Tainong 23 (TN23), do not accumulate water during fruit maturity. All the collected pineapple varieties were planted in Qujie Town, Xuwen, Zhanjiang, Guangdong, in November 2019. The basalt soil developed into brick-red soil and was used for germplasm cultivation. In May 2021, mature fruits were harvested for study in metabolome and transcriptome evaluation experiments. Three ripened pineapple fruits were harvested, peeled, and cut into four parts from each variety. One portion of each fruit was chosen randomly, and the pulp from all three fruits was combined to create a homogeneous sample. This sample was immediately immersed in liquid nitrogen, lyophilized, and kept in a −80 °C refrigerator until further analysis. We used these samples to perform triplicate transcriptome and metabolome analysis studies.

### 4.2. Morphological and Nutritional Estimation

The homogeneous mixture of all seven samples was used to determine vitamin C, titratable acidity (TC), soluble solids (SS), total soluble sugar (TSS), and N, P, and K content. The vitamin C content and TT were determined by titrating juice from 100 g of fresh pulp for each using the 2-6 dichloroindophenol titrimetric method described by Uckiah et al. [23] and expressed as ascorbic acid (mg/100 g) and citric acid (mmol/100 g), respectively. The SS was measured using refractrophotometer and expressed as g/100 g of pulp. The TSS was measured using the high-performance liquid chromatography (HPLC) method described by Chen and Paull [12] and expressed as percent content. The HPLC had an analysis column (Fast Carbo-hydrate, 100 × 7.8 mm, Bio-Rad Laboratories, Hercules, Calif). N, P, and K contents were measured using the method described by Chen, Zeng, and Zhang [3] and were expressed as percent content.

### 4.3. Metabolome Evaluation

#### 4.3.1. Sample Preparation and Extraction

Twenty-one freeze-dried samples were crushed for 1.5 min at 30 Hz in a mixer mill (MM 400, Retsch, Haan, Germany) outfitted with a zirconia bead. Then, 100 mg of lyophilized powder was dissolved in 1.2 mL of 70% methanol. The solution was vortexed six times for 30 s with 30 min intervals and stored in a refrigerator overnight at 4 °C. Before the mass spectrometry analysis, the extracts were centrifuged for 10 min at 12,000 rpm and 4 °C, and the supernatants were filtered (SCAA-104, 0.22 m pore size; ANPEL, Shanghai, China).

#### 4.3.2. Metabolite Detection and Analysis

##### UPLC Conditions

The extracted samples were analyzed using ultra-high-performance liquid chromatography–electrospray ionization–tandem mass spectrometry (UPLC-ESI-MS) (UPLC, Shim-pack UFLC SHIMADZU CBM30A system,; MS, Applied Bio-systems 4500 Q TRAP,). The test conditions were as follows: UPLC: column, Agilent SB-C18 (1.8 µm, 2.1 mm × 100 mm) (Agilent Technologies, Inc., Santa Clara, CA, USA); and the mobile phase comprised pure water with 0.1% formic acid (solvent A) and acetonitrile along with 0.1% formic acid (solvent B). Samples were resolved with a gradient of 95% A and 5% B, reaching an isocratic elution of 5% A and 95% B in 9 min, which was maintained for 1 min. Subsequently, a composition of 95% A and 5.0% B was adjusted within 1.10 min and kept for 2.9 min. The column oven was set to 40 °C, and the injection quantity was 4 μL. The effluent was alternatively connected to an ESI–triple quadrupole–linear ion trap (QTRAP)-MS.

##### ESI-Q TRAP-MS/MS

On a triple quadrupole–linear ion trap mass spectrometer (Q-TRAP), API 4500 Q TRAP UPLC/MS/MS System, equipped with an ESI Turbo Ion-Spray interface, operating in positive and negative ion mode, and controlled by Analyst 1.6.3 software, linear ion trap (LIT) and triple quadrupole (QQQ) scans were acquired (AB Sciex Pte. Ltd., Framingham, MA, USA). Instrument setting and mass calibration were performed in QQQ and LIT modes with 10 and 100 μmol/L polypropylene glycol solutions, respectively. QQQ scans were obtained as metabolic response modifier (MRM) tests with collision gas (nitrogen) set to 5 Ψ (psi). De-clustering potential (DP) and collision energy (CE) for different MRM transitions were carried out with further DP and CE optimization. A particular set of MRM shifts were monitored for every period in accordance with the metabolites eluted during this period.

#### 4.3.3. Data Processing and Analysis

On the basis of the self-built metware database (http://www.metware.cn/; accessed on 16 September 2021) and according to the secondary spectrum information, the isotopic signals, recurrent signals containing Na^+^, K^+^, and NH4^+^ ions, as well as the repetitive signals of fragment ions with higher molecular weight, were removed during the analysis. Metabolite quantification was performed by multiple reaction monitoring (MRM) analysis using triple quadrupole mass spectrometry. In MRM mode, the precursor ion (parent ion) of the target material was screened by the four-stage rod to eliminate the representing ions of other molecular weight elements to eliminate the interference preliminarily. The precursor ion was induced and ionized by the collision chamber to form a lot of fragment ions. Then, the fragment ion was filtered with a triple four-stage rod to select a required characteristic fragment ion to eliminate the interference of non-target ions. It is more accurate and repeatable. After obtaining the mass spectrometry data of metabolites from different samples, the peak areas of all mass spectra peaks were integrated, and the peaks of the same metabolite in different samples were integrated and corrected [24].

##### Principal Component Analysis (PCA)

The principal component analysis (PCA) was performed by statistics functions “prcomp” within R (www.r-project.org). The data were unit variance scaled before performing unsupervised PCA. By applying absolute log2 FC (fold change) ≥ 1 and variable importance for projection (VIP) ≥ 1, significant regulated metabolites between groups were identified. The orthogonal partial least squares discriminant analysis (OPLS-DA) result, which also includes score plots and permutation plots, was produced using the R package MetaboAnalystR. VIP values were then retrieved from these data. Before using OPLS-DA, the data were mean-centered and log-transformed (log2). To prevent overfitting, a permutation test (with 200 permutations) was run.

##### Hierarchical Cluster Analysis and Pearson Correlation Coefficients

The HCA (hierarchical cluster analysis) results of samples and metabolites were expressed as heatmaps with dendrograms, while Pearson correlation coefficients (PCC) between samples were calculated by the core function in R and presented only as heatmaps. Both HCA and PCC were conducted by R package ComplexHeatmap. For HCA, normalized signal strengths of metabolites (unit variance scaling) were pictured as a color spectrum.

##### Differential Metabolites Selected

Significantly controlled metabolites between groups were found by VIP ≥ 1 and total log2FC (fold change) ≥ 1. VIP values were obtained from the OPLS-DA result, which also included score plots and permutation plots and were created using the R package MetaboAnalystR. The data was then log-transformed (log2) and mean-centered prior to OPLS-DA analysis. A permutation test with 200 permutations was carried out to prevent overfitting.

#### 4.3.4. Kyoto Encyclopedia of Gene and Genomes (KEGG) Functional Annotation and Enrichment Analysis

Identified metabolites were annotated using the Kyoto Encyclopedia of Gene and Genomes (KEGG) compound database (http://www.kegg.jp/kegg/compound/; accessed on 16 September 2021) and mapped to the KEGG Pathway database (http://www.kegg.jp/kegg/pathway.html; accessed on 16 September 2021). Pathways with significantly regulated metabolites were then fed into metabolite sets enrichment analysis (MSEA). The hypergeometric test’s *p*-values determined the significance.

### 4.4. Transcriptome Analysis

#### 4.4.1. RNA Extraction and Preparation of Library

The total RNA was extracted from the fruit pulp of each sample using the cetyltrimethylammonium bromide (CTAB) method [25] for all seven samples. The extracted RNA was further evaluated for concentration [by NanoDrop™ 2000 micro-spectrophotometer (Thermo Fisher Scientific Inc., Waltham, MA, USA)], purity [by Agilent 2100 Bioanalyzer (Agilent Technologies, Inc., Santa Clara, CA, USA)], and integrity (in agarose gel). The step-by-step process, including total RNA sample detection, mRNA enrichment with Oligo (dT) beads, fragmentation with fragmentation buffer, synthesis of double-stranded cDNA, purification and end repair, splice selection, PCR amplification library quality detection, and the computer-based sequencing, was performed at Wuhan medwell technology, China. Finally, the paired-end reads were generated.

#### 4.4.2. RNA Sequencing, Cleaning, and Assembly

The original image data files were obtained by high-throughput sequencing [Illumina hiseq 4000 (illumine, San Diego, CA, USA)] and were transformed into raw reads by base calling analysis. As per the machine’s sequencing strategy, an average read length of 150 bp was maintained. Raw data (raw reads) were processed using NGS QC Toolkit [26]. The unprocessed reads with joint sequences and/or less than 5 mass values, greater than or equal to 50% proportion rate, greater than or equal to 5% N-base (the base with undetermined data), or containing Poly-A were screened out to obtain the cleaned reads. For transcriptome assembly and to obtain the unigenes, the Trinity v 2.6.6 program [27] was used. The estimation of N50 and exN50 ensured the correctness and efficiency of the assembly results.

#### 4.4.3. Expression Evaluation and Differentially Expressed Gene Identification

The numbers of read counts on each gene were obtained from each sample, and the gene expression level was estimated by the fragments per kilobase of transcript per million mapped reads (FPKM) method. The FPKM value of each gene was determined using cufflinks [28], and the htseq-count provided the read counts of each gene [29]. Differentially expressed genes (DEGs) were identified using the DESeq (with replicates) [30]. *p*-value < 0.05 and log2 fold change > 1 for upregulated and fold change < −1 for downregulated DEGs were set as the thresholds for significant differential expression. Principal component analysis of DEGs was performed to explore gene expression patterns.

#### 4.4.4. Functional Annotation and Enrichment Study

Transcoder software v 4.1.0 was used to predict and translate the reading frames from extracted unigenes. Gene Ontology (GO) [31] enrichment and KEGG pathway [16] enrichment analysis of DEGs were performed using R based on the hypergeometric distribution. For GO and KEGG annotation, Blast2go [15] and Kaas software [32] (https://www.genome.jp/tools/kaas/; accessed on 27 September 2021) tools were used, respectively, and for enrichment analysis, the Phyper function in R software was used. The gene expression was revealed by comparing the sequenced reads with the unigene library in Bowtie [33].

## 5. Conclusions

The manifestation of pineapple translucency is a complex process involving multiple genes and various metabolic processes. This study showed no direct relation between macronutrient concentration and pineapple translucency. Translucency results in the difference in dry matter, soluble sugar content, and differential accumulation of alkaloids, phenolic acids, nucleotide derivatives, lipids, and other metabolites. The expression amounts of genes related to ‘flavonoid biosynthesis’ pathways were downregulated, and the Metabolic pathway, Biosynthesis of secondary metabolites, Plant–pathogen interaction, and Plant hormone signal transduction were also mostly differentially expressed. Upregulation of the sugar metabolism, such as CWI, was likely how inter- and intracellular sugar homeostasis was eventually breached, leading to water accumulation and translucency formation. These results provide an insight into the molecular mechanism for pineapple translucency in terms of metabolic and transcriptional levels. Future studies into techniques for reducing pineapple translucency will benefit from these findings.

## Figures and Tables

**Table 1:** Pulp quality and macronutrient composition of different varieties of pineapple.

	Variety	Pulp Quality				Macronutrients		
		Vitamin C (mg/100 g)	Titratable Acidity (mmol/100 g)	Soluble Solids	Total Soluble Sugar (%)	N%	P%	K%
Watery	TN17 ^1^	11.0 ± 3.6	6.1 ± 0.4	14.4 ± 2.1	16.0 ± 2.1	0.50 ± 0.04	0.082 ± 0.022	1.13 ± 0.13
	MD-2 ^2^	33.0 ± 4.2	4.7 ± 0.4	12.1 ± 0.9	14.6 ± 1.2	0.56 ± 0.01	0.077 ± 0.002	1.61 ± 0.08
	CN ^3^	4.0 ± 0.7	5.4 ± 0.9	15.5 ± 0.5	18.6 ± 0.8	0.51 ± 0.00	0.076 ± 0.002	1.17 ± 0.04
Non-watery	BL ^4^	19.6 ± 1.1	6.6 ± 0.4	15.7 ± 0.8	18.1 ± 0.7	0.56 ± 0.03	0.057 ± 0.005	1.29 ± 0.04
	TN4 ^5^	2.8 ± 1.7	4.7 ± 0.2	24.0 ± 0.6	27.1 ± 0.5	0.33 ± 0.01	0.052 ± 0.004	1.17 ± 0.07
	TN23 ^6^	5.4 ± 0.7	6.7 ± 0.4	18.9 ± 0.6	21.2 ± 1.0	0.42 ± 0.05	0.086 ± 0.014	1.22 ± 0.11
	TN21 ^7^	10.6 ± 0.2	7.9 ± 0.4	19.6 ± 1.1	22.7 ± 2.2	0.57 ± 0.06	0.088 ± 0.007	1.31 ± 0.08

^1^ Golden diamond or Tainong 17, ^2^ Golden pineapple, ^3^ Caine or Smooth cayenne (CN), ^4^ Bali or Comte-de-Paris, ^5^ Shredded pineapple or Tainong 4, ^6^ Mango pineapple or Tainong 23, and ^7^ Golden pineapple or Tainong 21.

**Table 2:** Classes and subclasses of metabolites detected in pineapple pulp.

Class	Count	Subclass	Count
Alkaloids	57	Alkaloids	31
		Phenolamine	9
		Piperidine alkaloids	2
		Plumerane	11
		Pyridine alkaloids	2
		Pyrrole alkaloids	1
		Quinoline alkaloids	1
Amino acids and derivatives	83	Amino acids and derivatives	83
Flavonoids	47	Flavanols	28
		Flavanones	4
		Flavones	13
		Flavonoid carbonoside	1
		Flavonols	1
Lignans and Coumarins	22	Coumarins	9
		Lignans	13
Lipids	101	Free fatty acids	50
		Glycerol ester	14
		Lysophosphatidylcholine	18
		Lysophosphatidylethanolamine	15
		Sphingolipids	4
Nucleotides and derivatives	48	Nucleotides and derivatives	48
Organic acids	51	Organic acids	51
Phenolic acids	126	Phenolic acids	126
Terpenoids	3	Triterpene	3
Others	103	Vitamin	16
		Saccharides and Alcohols	75
		Stilbene	2
		Others	10
		Total =	641

**Table 3:** De novo transcriptome assembly data summary.

Sample	Reads		Clean Base (G)	Error Rate (%)	Q20 (%)	Q30 (%)	GC Content (%)
	Raw	Clean					
BL							
1	47,415,582	45,242,070	6.79	0.03	97.54	93.35	50.34
2	46,471,154	44,323,968	6.65	0.03	97.61	93.51	50.18
3	49,024,380	46,511,002	6.98	0.03	97.46	93.12	49.88
CN							
1	48,863,120	45,156,688	6.77	0.03	97.45	93.18	50.19
2	47,933,266	44,519,648	6.68	0.03	97.46	93.21	49.85
3	45,033,994	40,490,924	6.07	0.02	98.12	94.78	49.67
MD-2							
1	45,625,802	41,102,634	6.17	0.02	98.05	94.61	50.78
2	47,281,298	42,400,008	6.36	0.02	98.06	94.6	50.05
3	44,750,384	41,204,434	6.18	0.03	97.48	93.26	50.13
TN17							
1	44,644,768	42,760,620	6.41	0.03	97.56	93.36	48.74
2	52,206,800	49,770,154	7.47	0.03	97.61	93.45	48.09
3	44,187,080	42,938,052	6.44	0.03	97.53	93.32	49.19
TN21							
1	48,813,048	46,177,996	6.93	0.03	97.48	93.27	50.61
2	49,007,308	46,710,094	7.01	0.03	97.42	93.08	50.71
3	44,521,588	41,933,646	6.29	0.03	97.48	93.23	50.14
TN23							
1	48,717,078	45,424,292	6.81	0.03	97.57	93.49	50.83
2	48,281,550	44,655,710	6.7	0.03	97.51	93.33	50.71
3	48,420,344	44,827,328	6.72	0.03	97.43	93.16	50.73
TN4							
1	47,701,848	45,525,630	6.83	0.03	97.59	93.45	49.05
2	47,011,974	45,047,748	6.76	0.03	97.49	93.25	49.05
3	48,026,532	46,617,234	6.99	0.03	97.43	93.05	48.4

**Table 4:** De novo transcriptome assembly characteristic descriptive.

Descriptive	Value
Total length (bp)	127,836,531
Total number	24,914
N50 (bp)	1510
Average (bp)	5131.112266
Minimum (bp)	34
Maximum (bp)	119,978
Number of contigs ≥ 300 bp	24,515
Number of contigs ≥ 500 bp	23,869
Number of contigs ≥ 1000 bp	21,847

## Data Availability

We summarized the datasets used in this manuscript and presented them as supporting information for publication. The corresponding author will make any other relevant information available upon reasonable request.

## Supplementary Materials

The following supporting information can be downloaded at: https://www.mdpi.com/article/10.3390/ijms24087199/s1.
