# Supplementary material for: Integrated Metabolome and Transcriptome Analysis Reveals a Potential Mechanism for Water Accumulation Mediated Translucency in Pineapple (Ananas comosus (L.) Merr.) Fruit
Source: Int J Mol Sci. 2023 Apr 13;24(8):7199. doi: 10.3390/ijms24087199 (PMC10139408; doi:10.3390/ijms24087199)

Figure S1: KEGG Barblots between different pineapple species.

BL_vs_CN_KEGG_Barplot

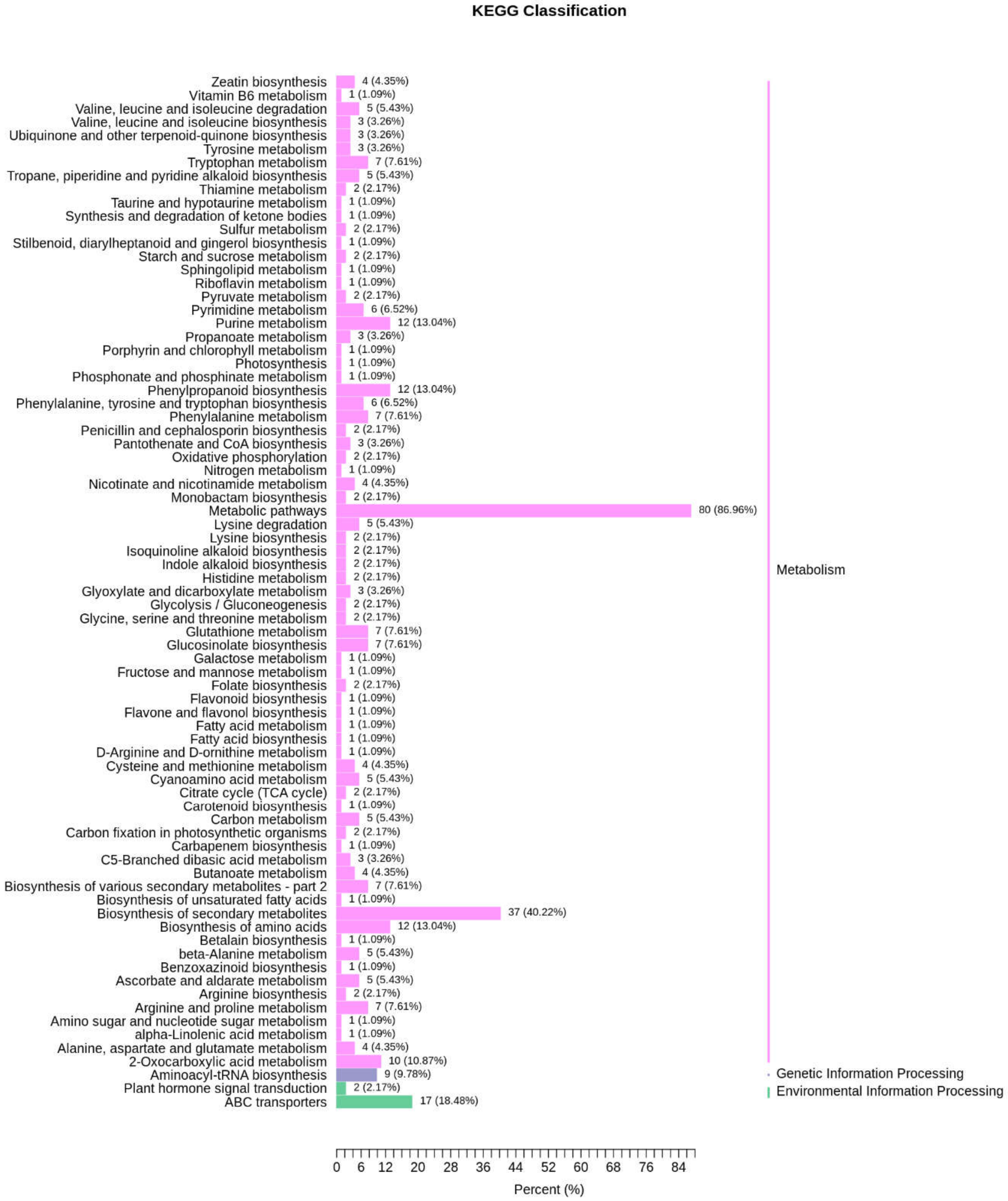

Figure S1: KEGG Barblots between different pineapple species.

BL_vs_TN17_KEGG_Barplot

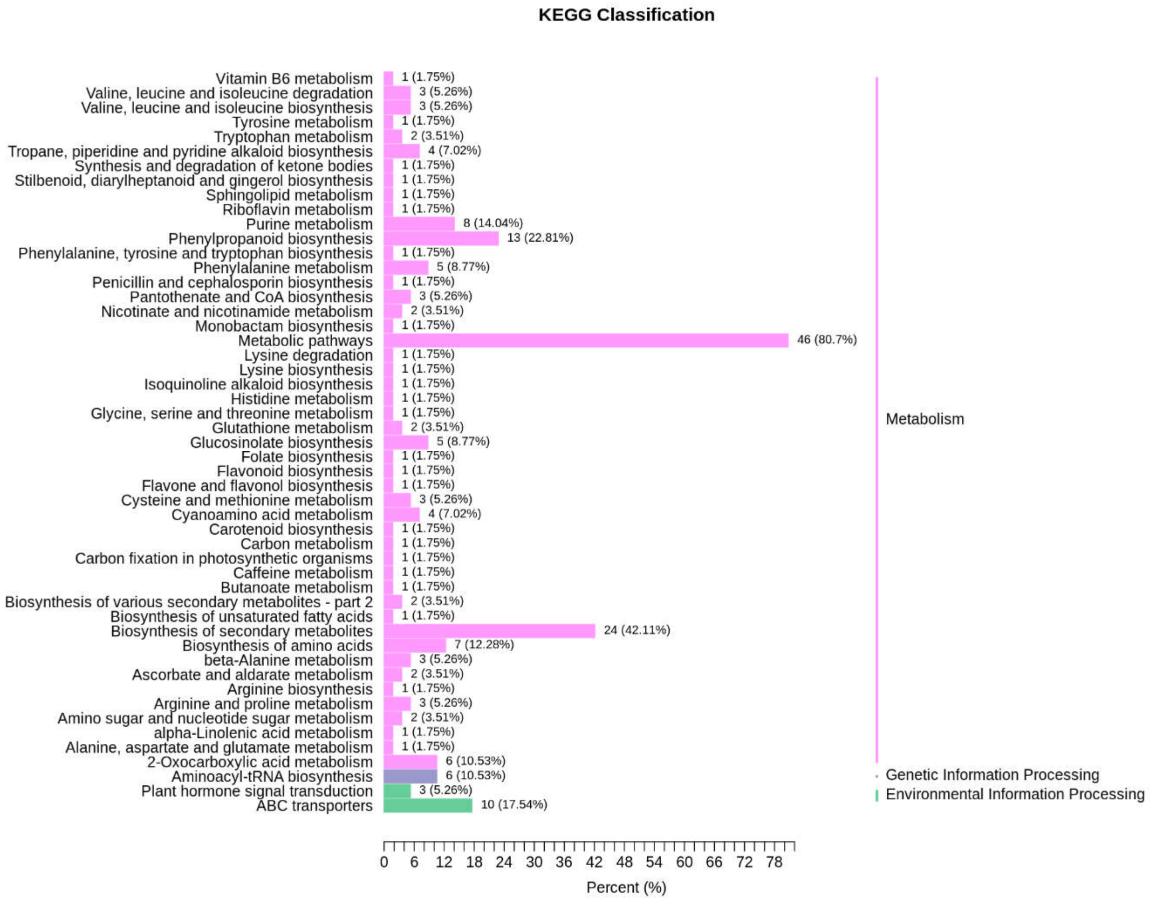

Figure S1: KEGG Barblots between different pineapple species.

BL_vs_TN21_KEGG_Barplot

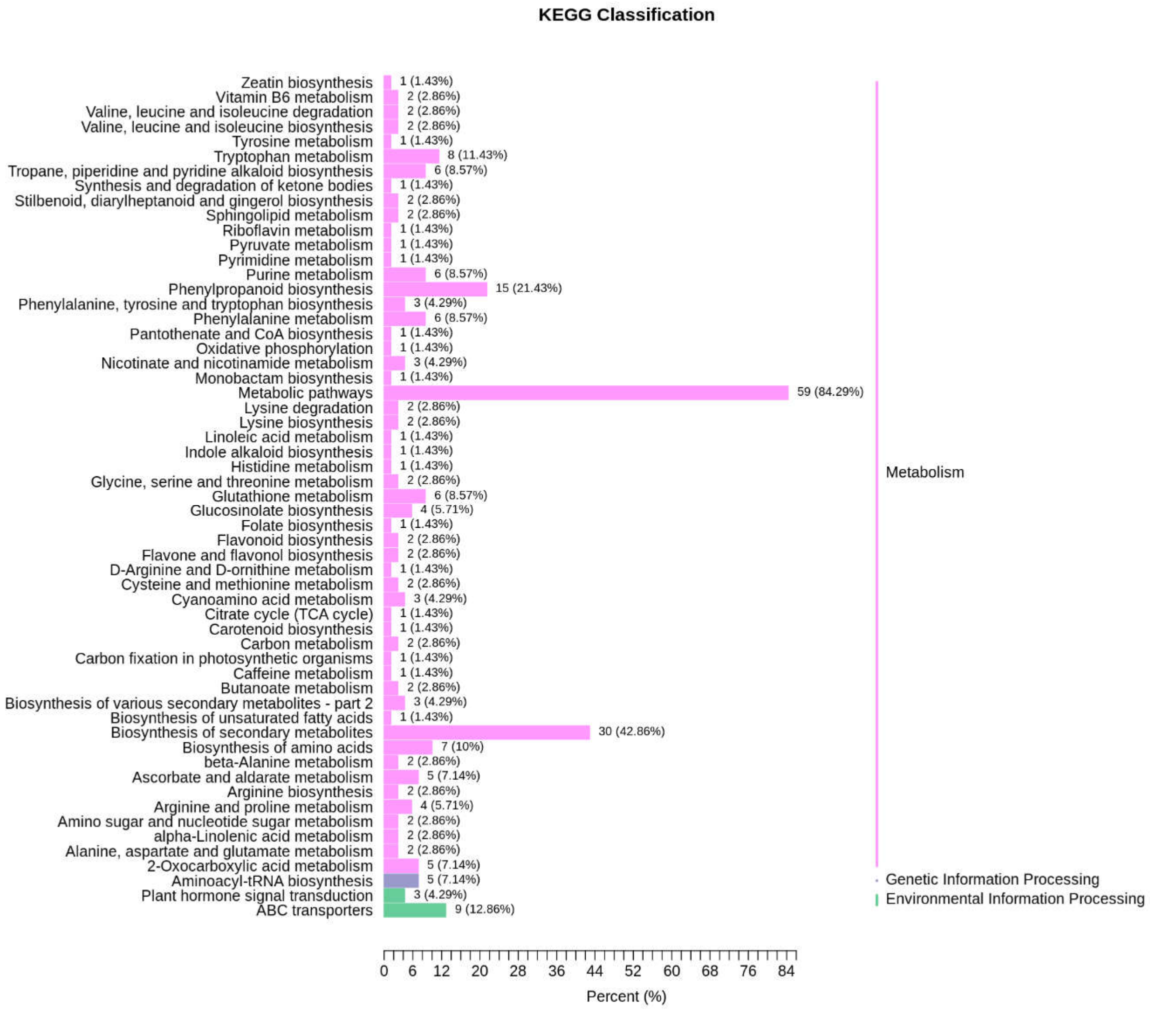

Figure S1: KEGG Barblots between different pineapple species.

BL_vs_TN23_KEGG_Barplot

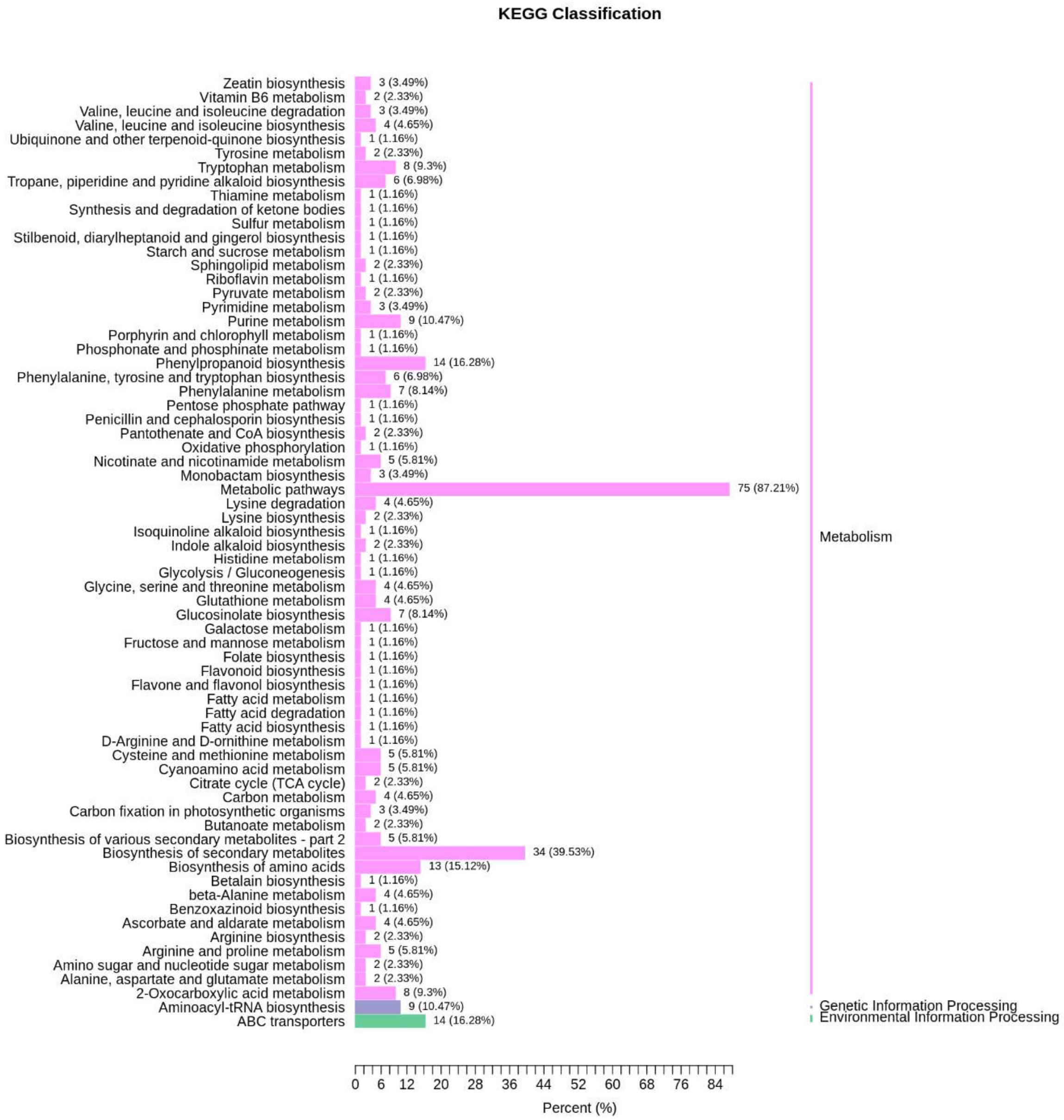

Figure S1: KEGG Barblots between different pineapple species.

BL_vs_TN4_KEGG_Barplot

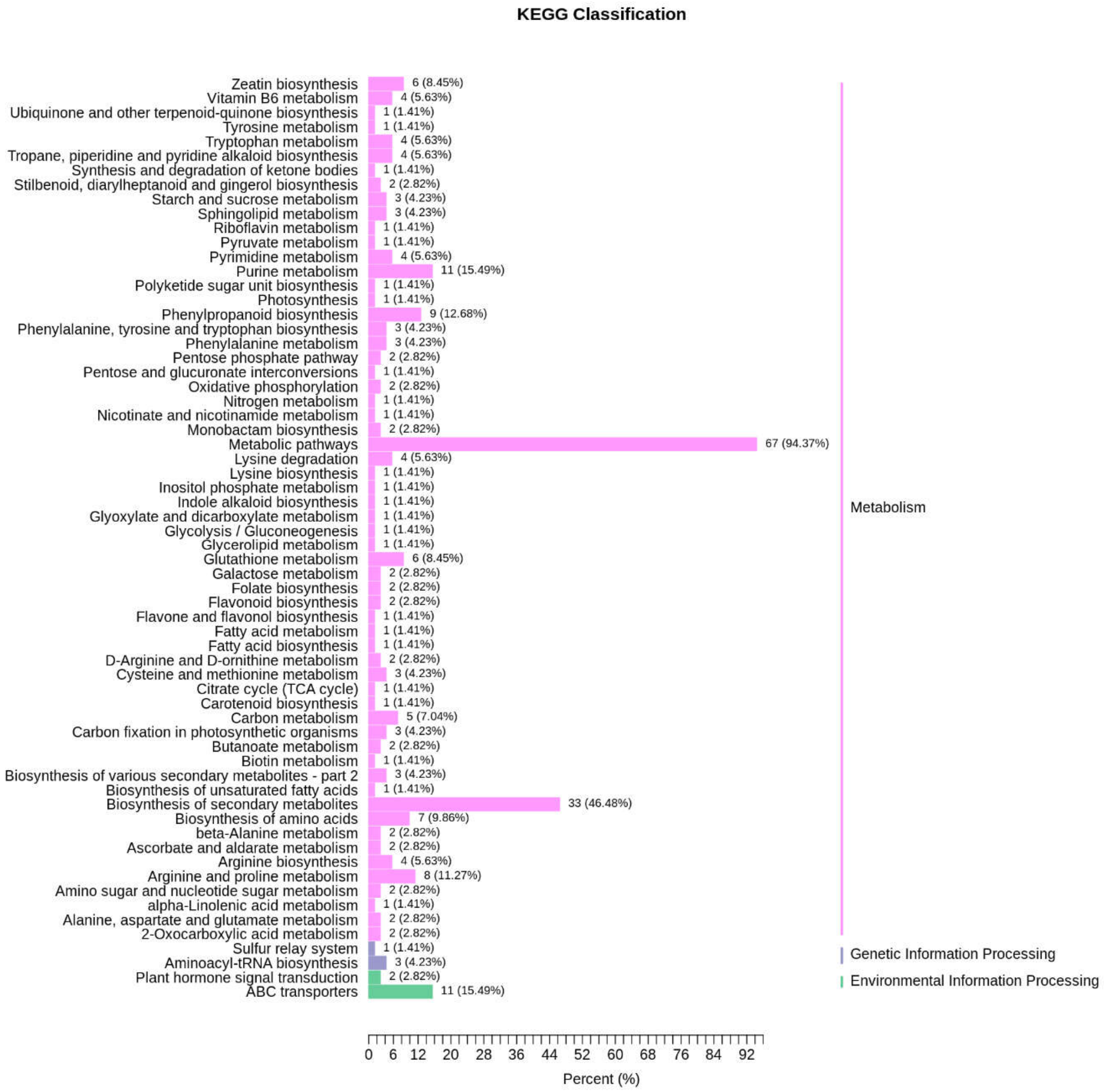

Figure S1: KEGG Barblots between different pineapple species.

CN_vs_TN17_KEGG_Barplot

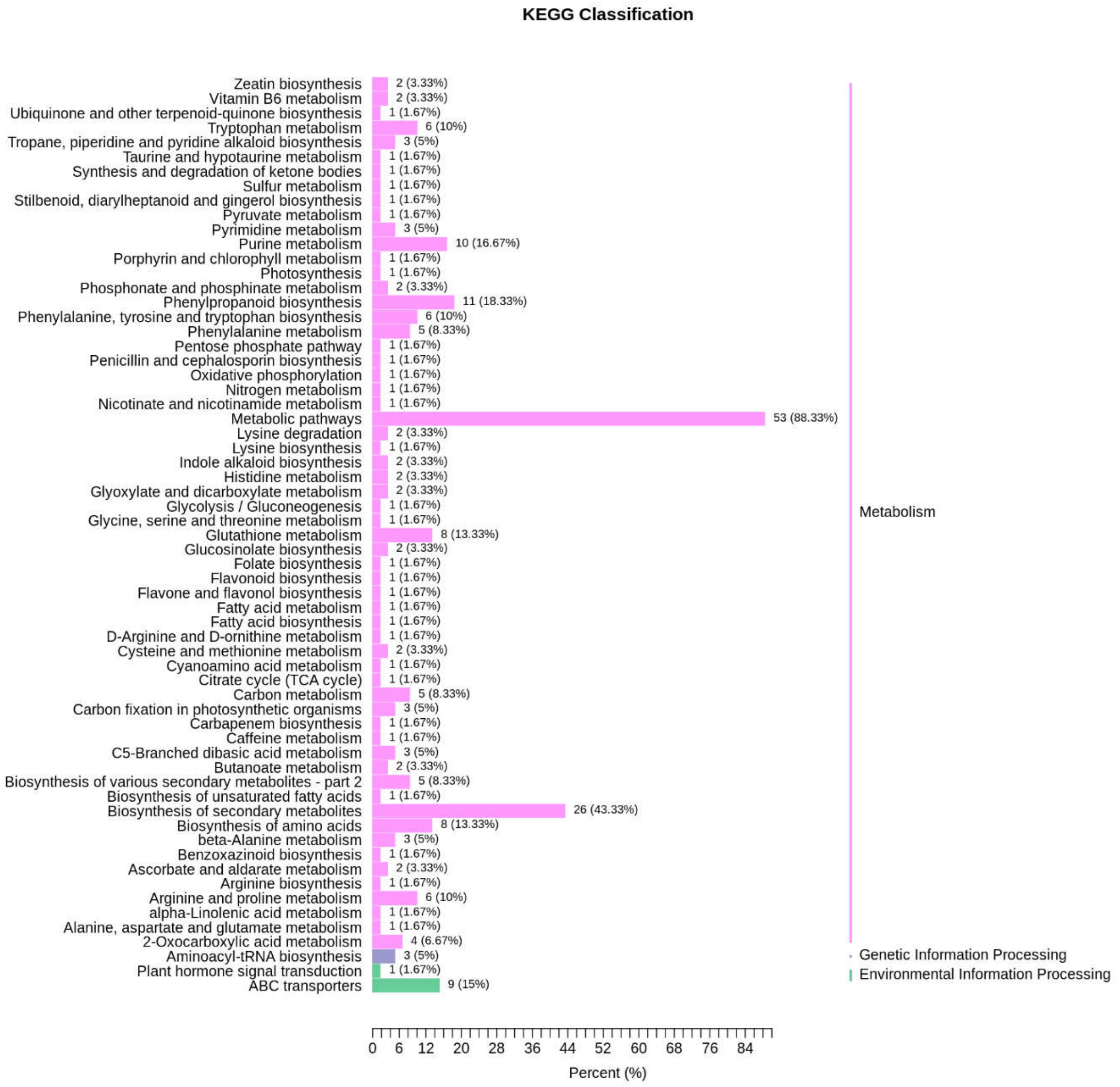

Figure S1: KEGG Barblots between different pineapple species.

CN_vs_TN21_KEGG_Barplot

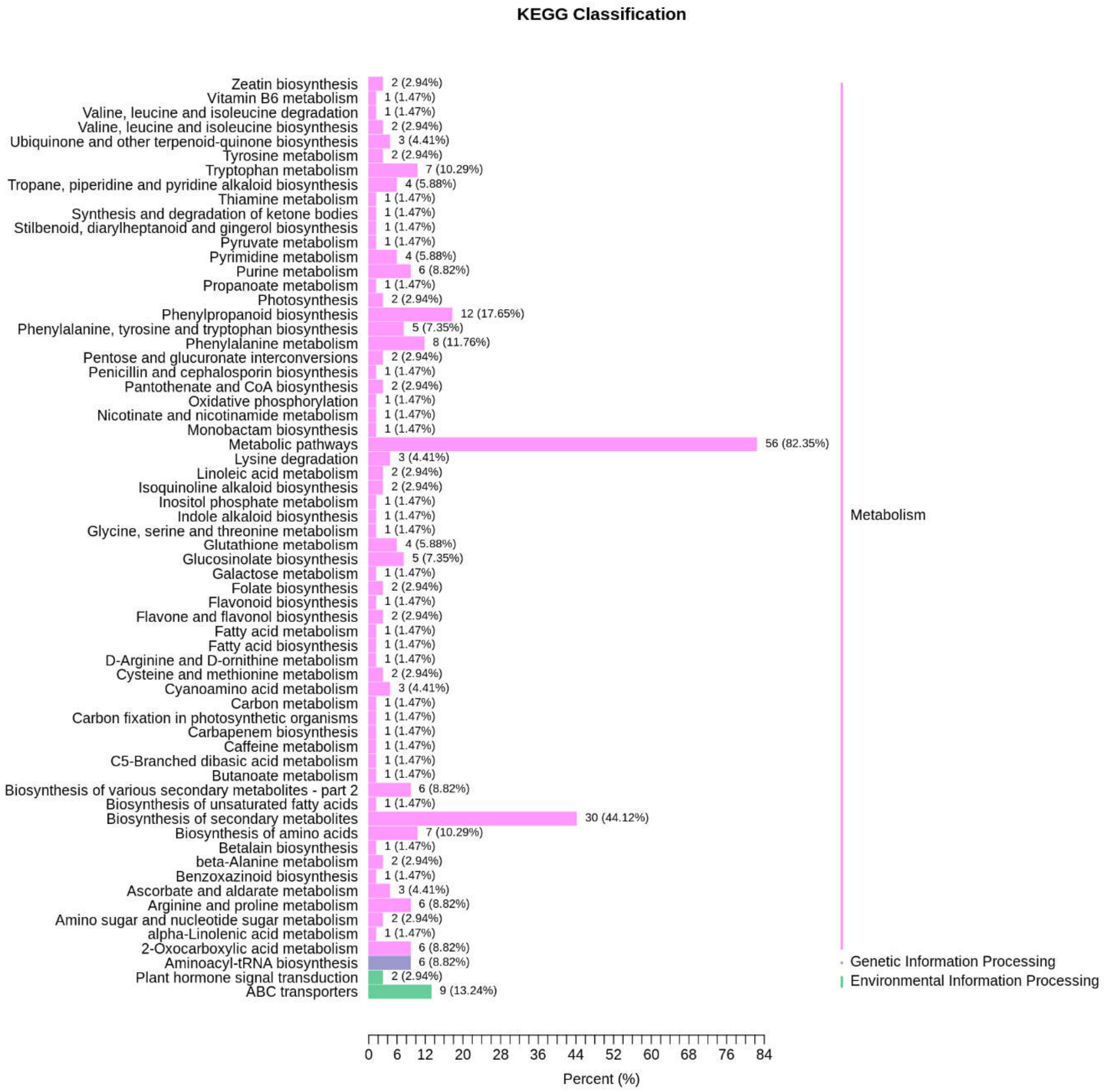

Figure S1: KEGG Barblots between different pineapple species.

CN_vs_TN23_KEGG_Barplot

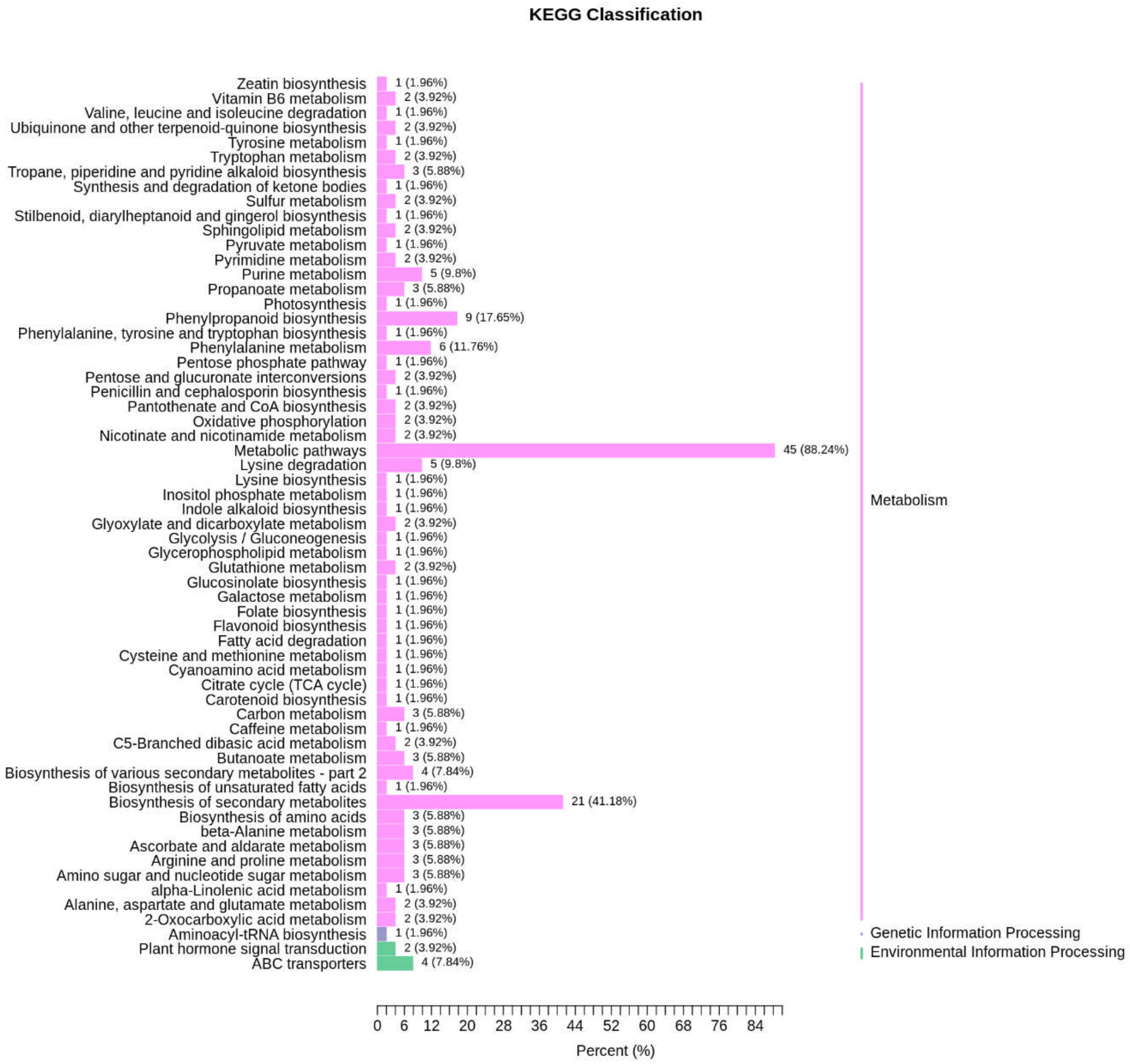

Figure S1: KEGG Barblots between different pineapple species.

CN_vs_TN4_KEGG_Barplot

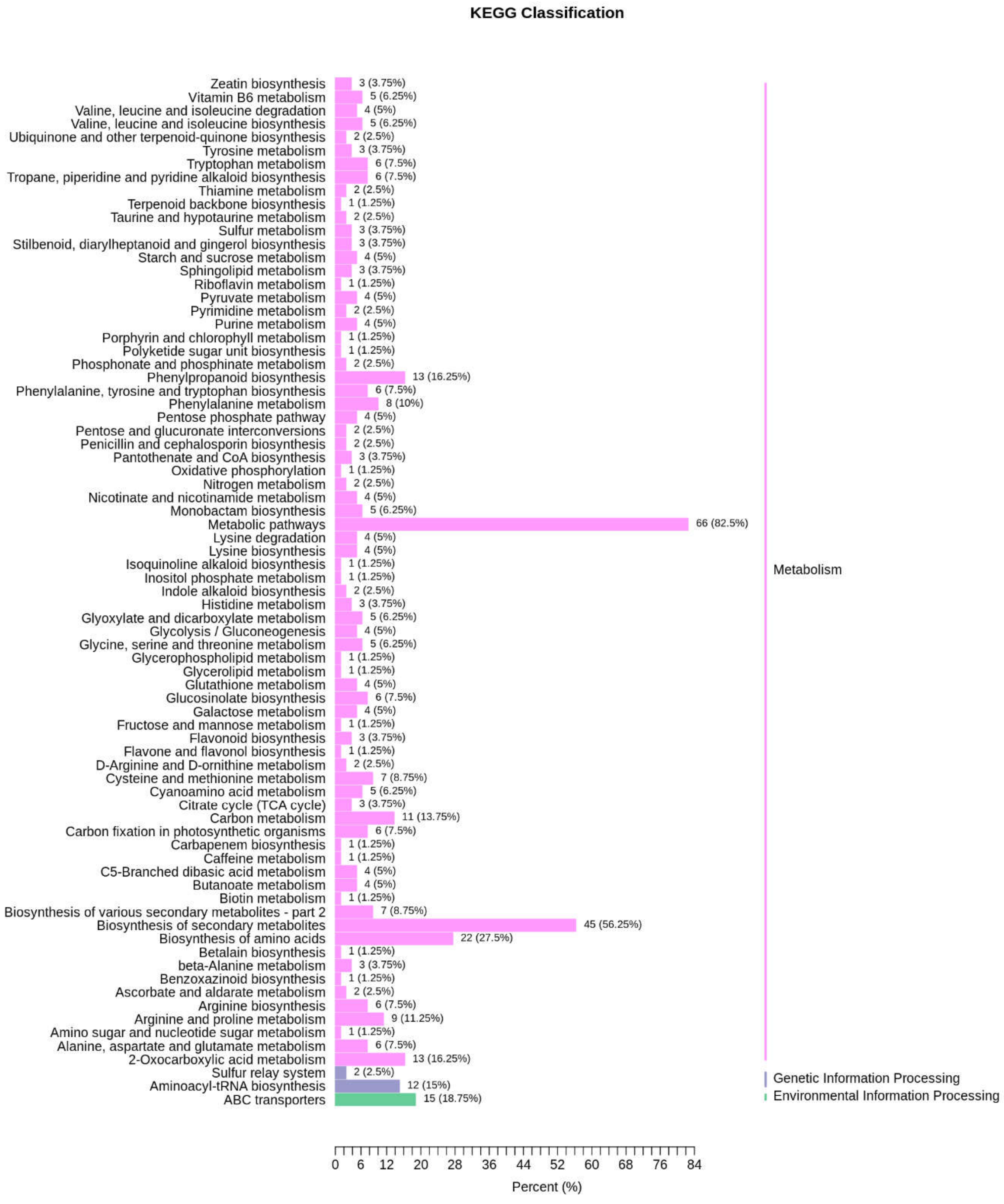

Figure S1: KEGG Barblots between different pineapple species.

MD-2_vs_BL_KEGG_Barplot

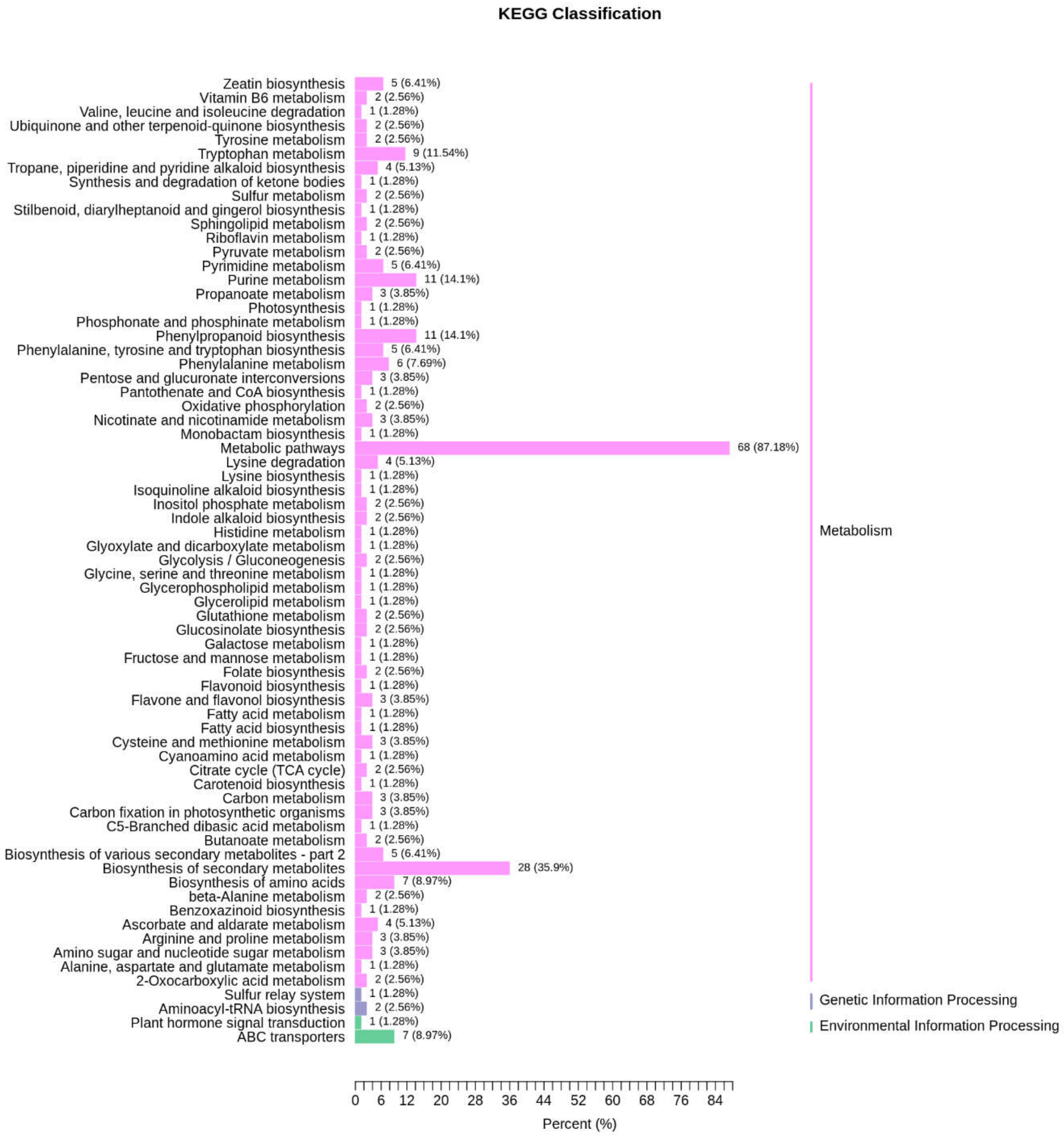

Figure S1: KEGG Barblots between different pineapple species.

MD-2_vs_CN_KEGG_Barplot

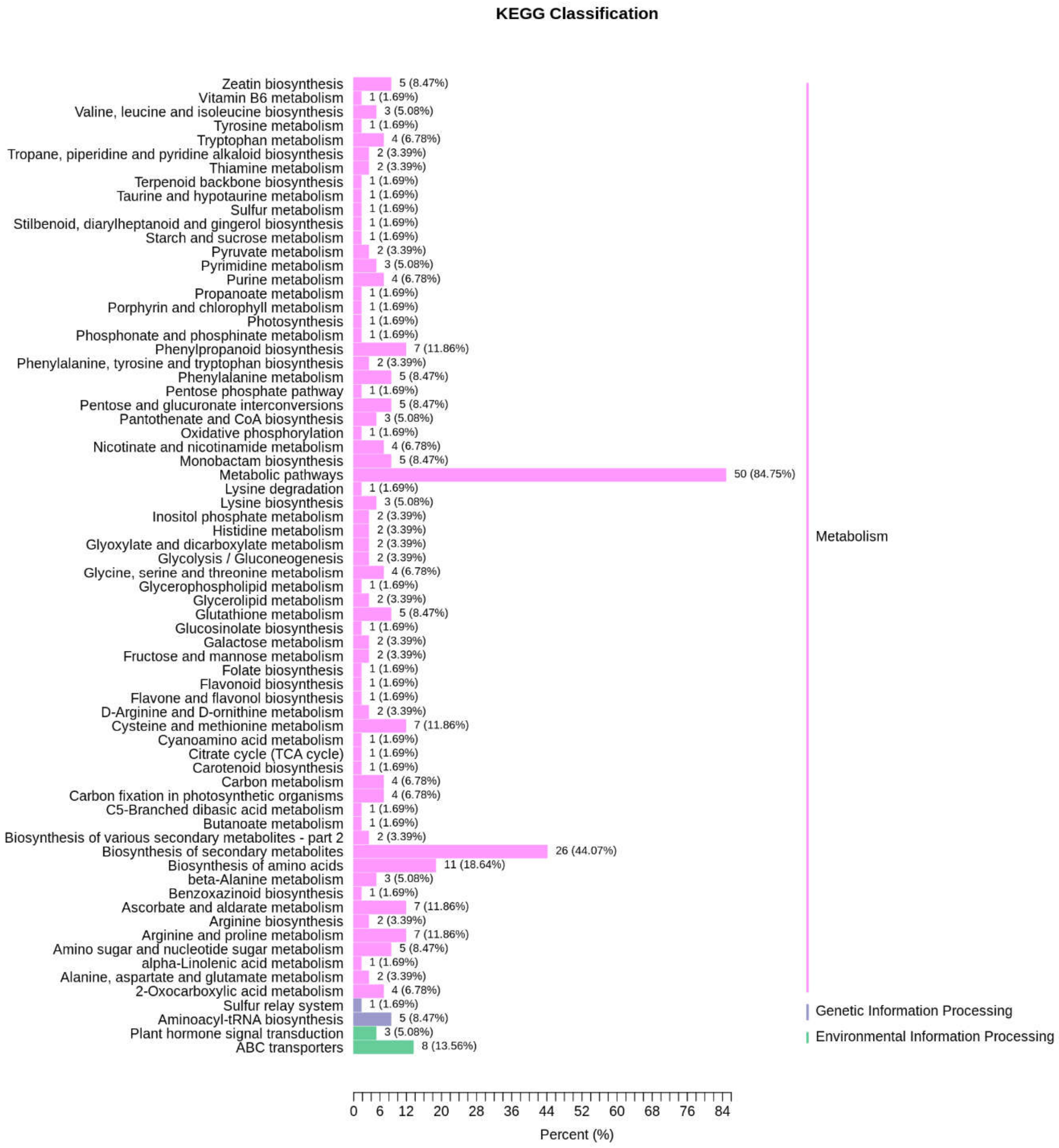

Figure S1: KEGG Barblots between different pineapple species.

MD-2_vs_TN17_KEGG_Barplot

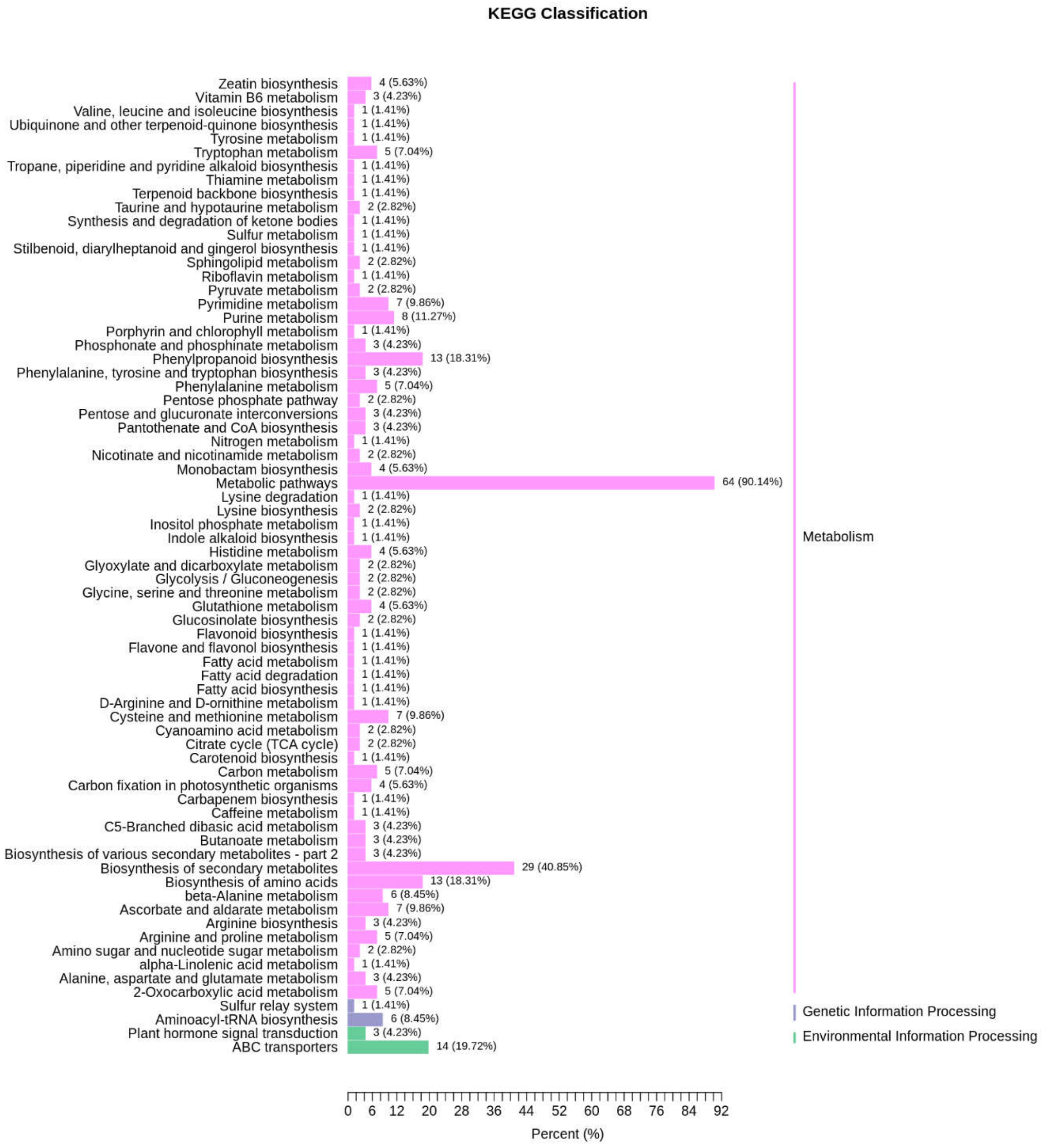

Figure S1: KEGG Barblots between different pineapple species.

MD-2_vs_TN21_KEGG_Barplot

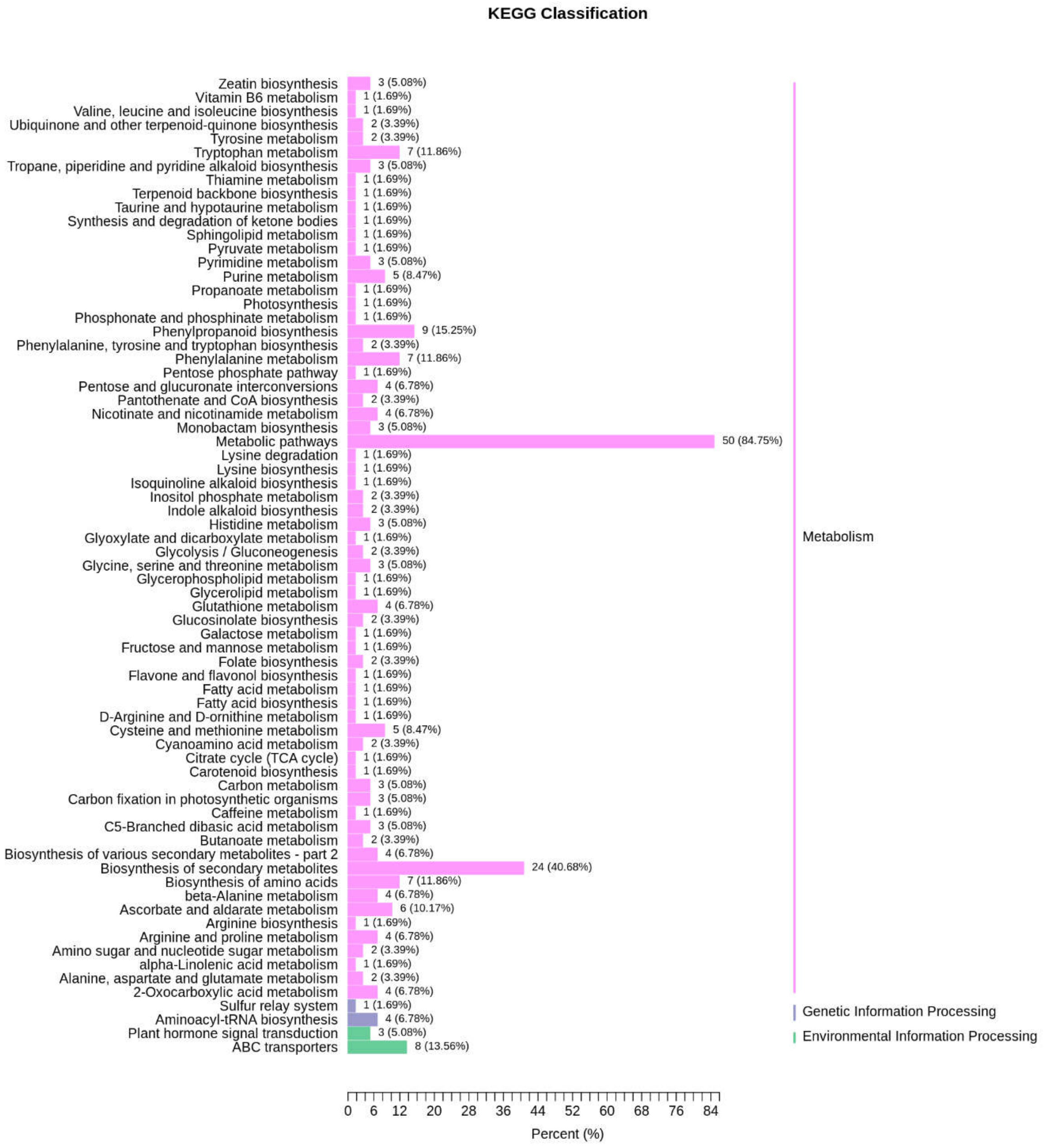

Figure S1: KEGG Barblots between different pineapple species.

MD-2_vs_TN23_KEGG_Barplot

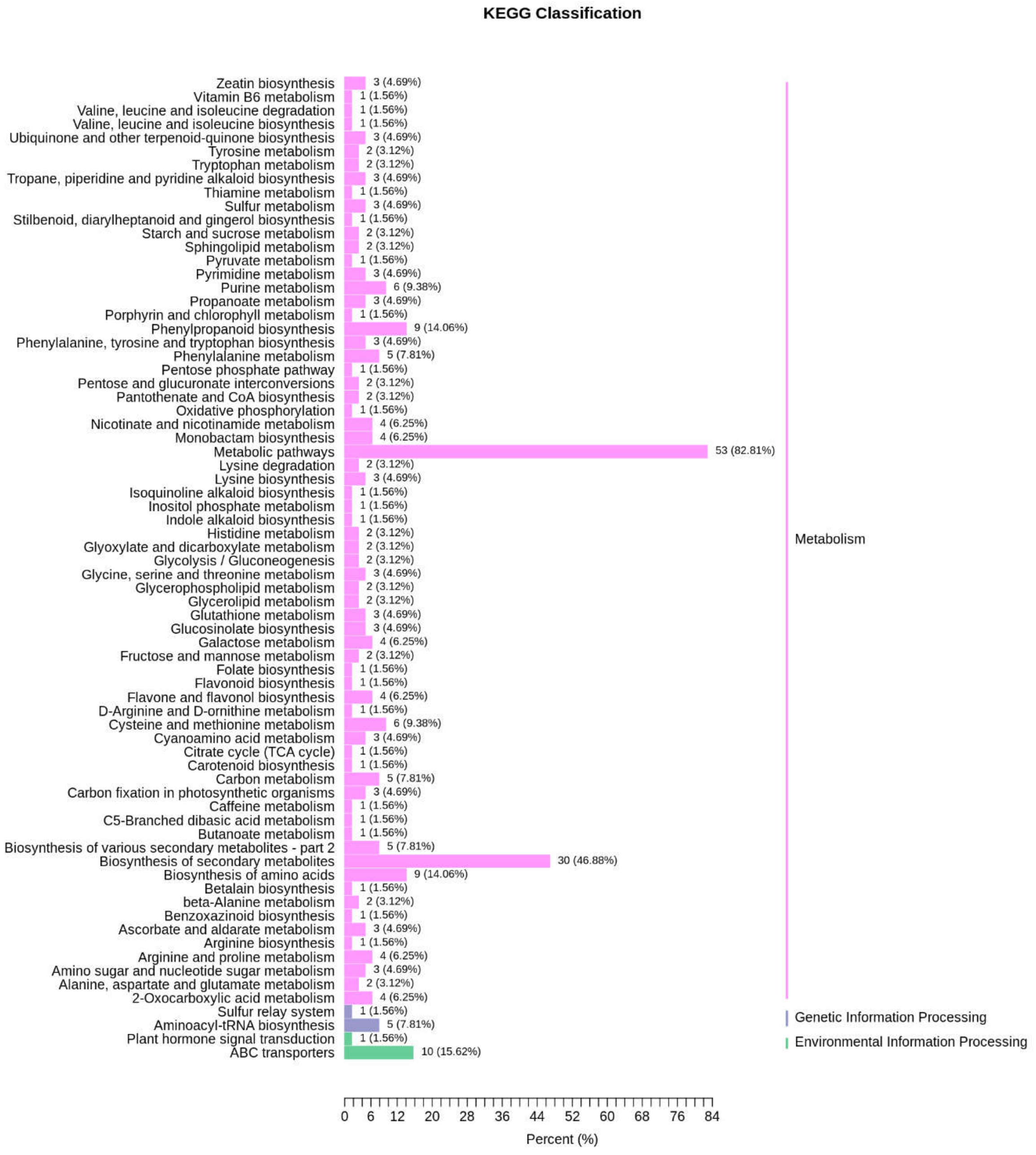

Figure S1: KEGG Barblots between different pineapple species.

MD-2_vs_TN4_KEGG_Barplot

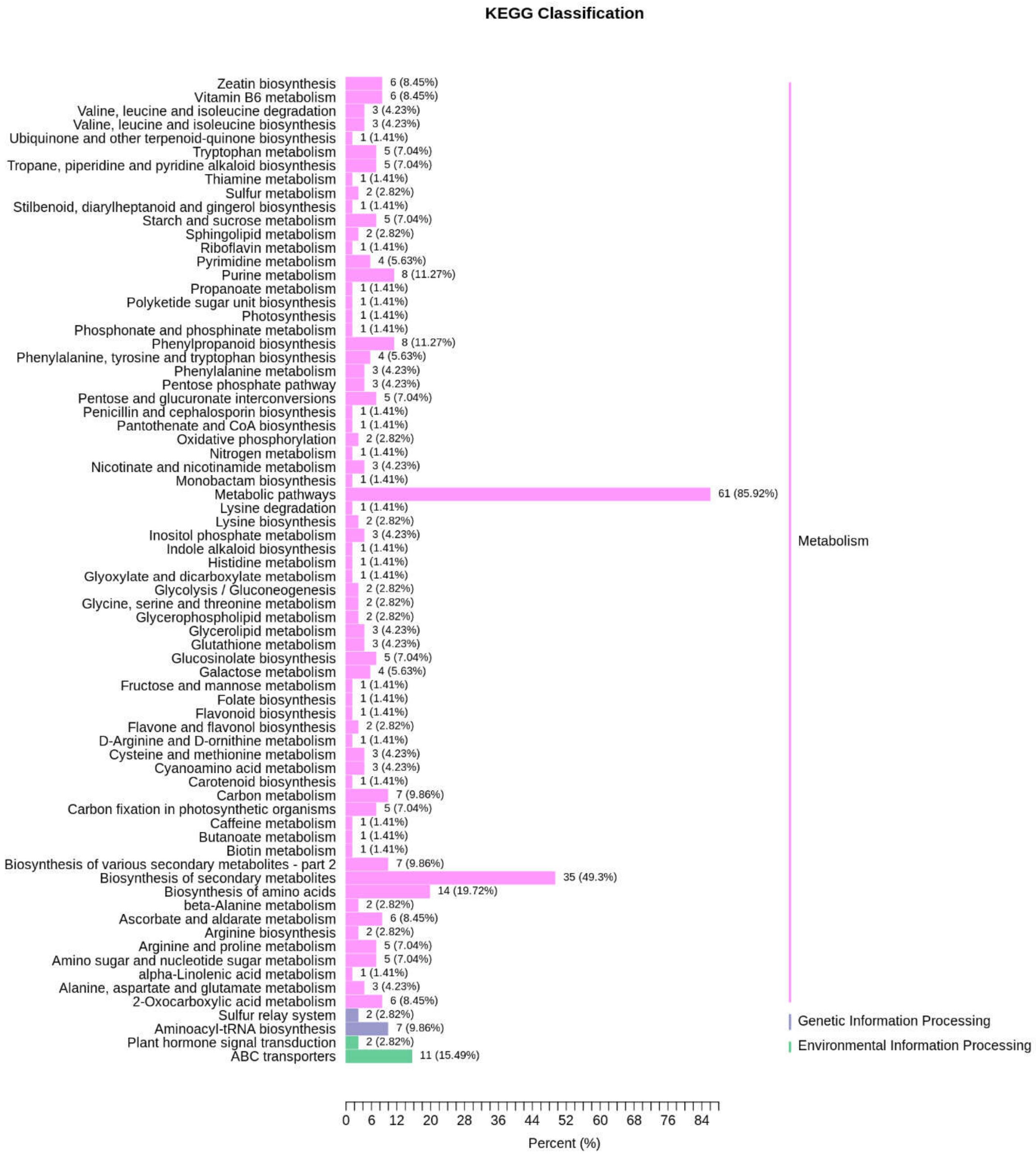

Figure S1: KEGG Barblots between different pineapple species.

TN17_vs_TN21_KEGG_Barplot

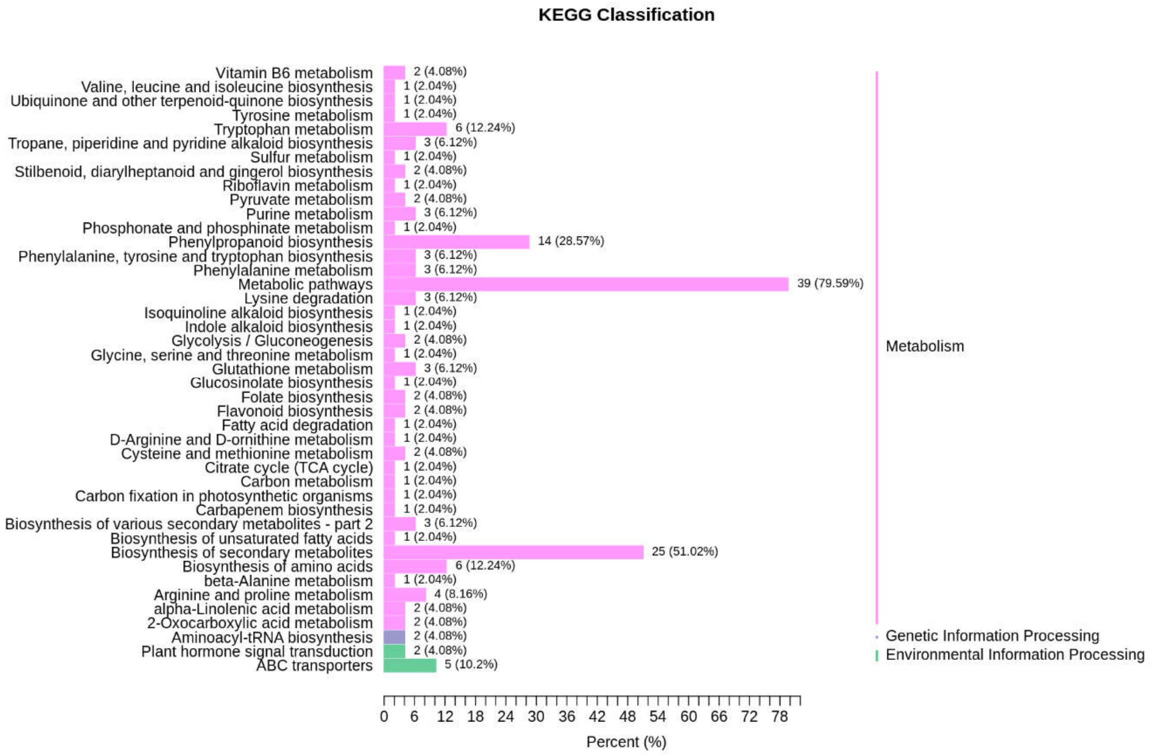

Figure S1: KEGG Barblots between different pineapple species.

TN17_vs_TN23_KEGG_Barplot

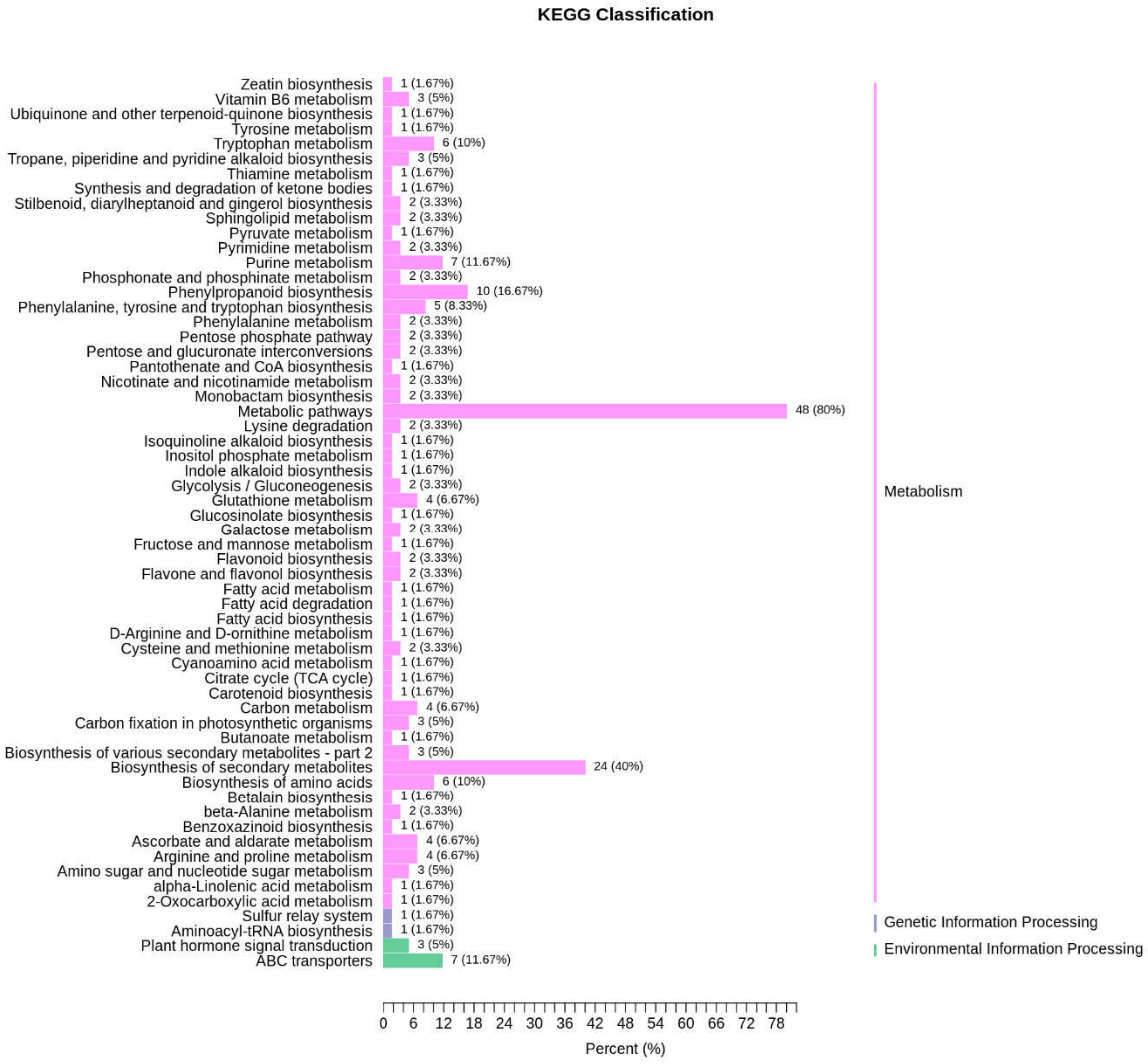

Figure S1: KEGG Barblots between different pineapple species.

TN21_vs_TN23_KEGG_Barplot

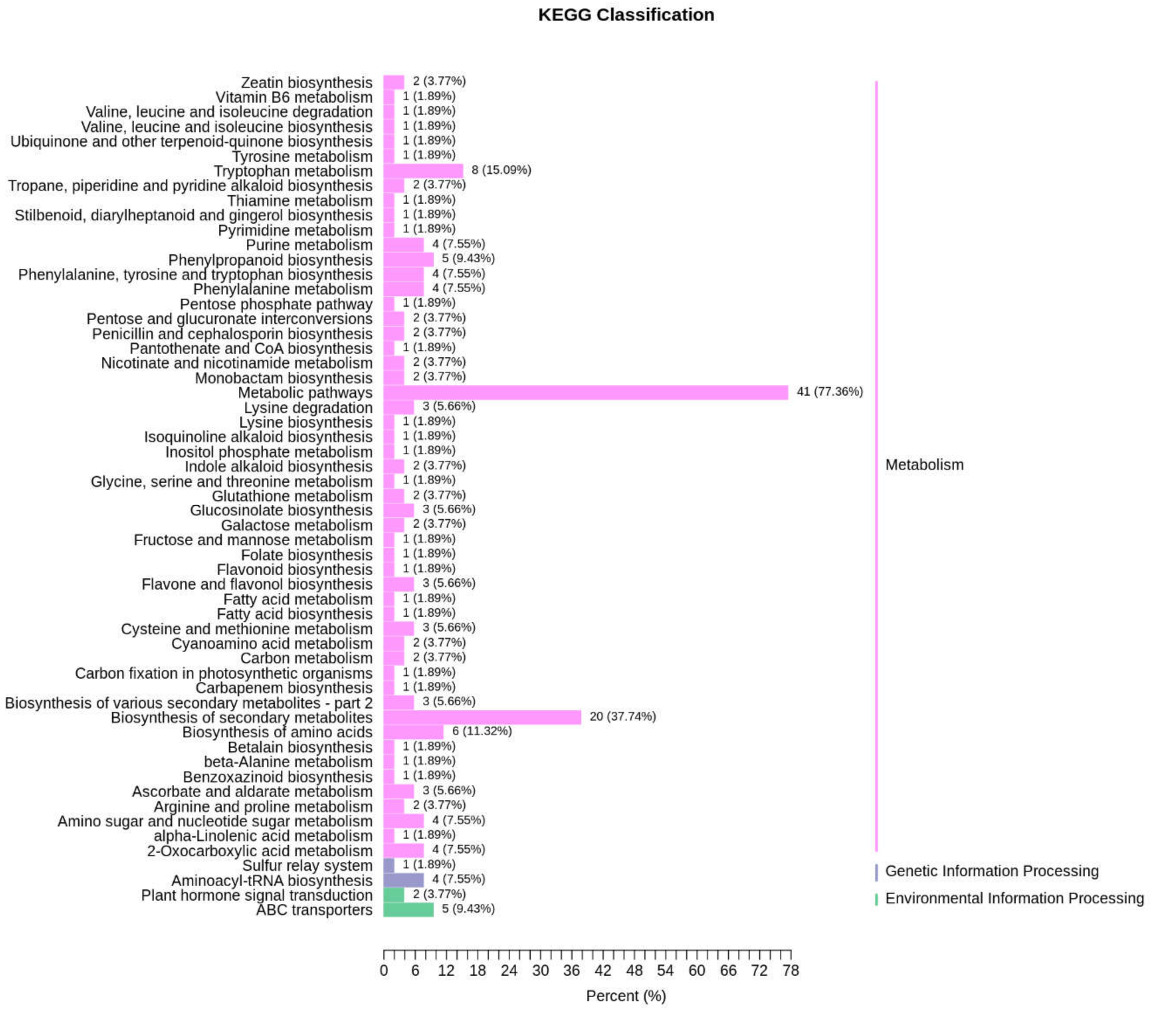

Figure S1: KEGG Barblots between different pineapple species.

TN4_vs_TN17_KEGG_Barplot

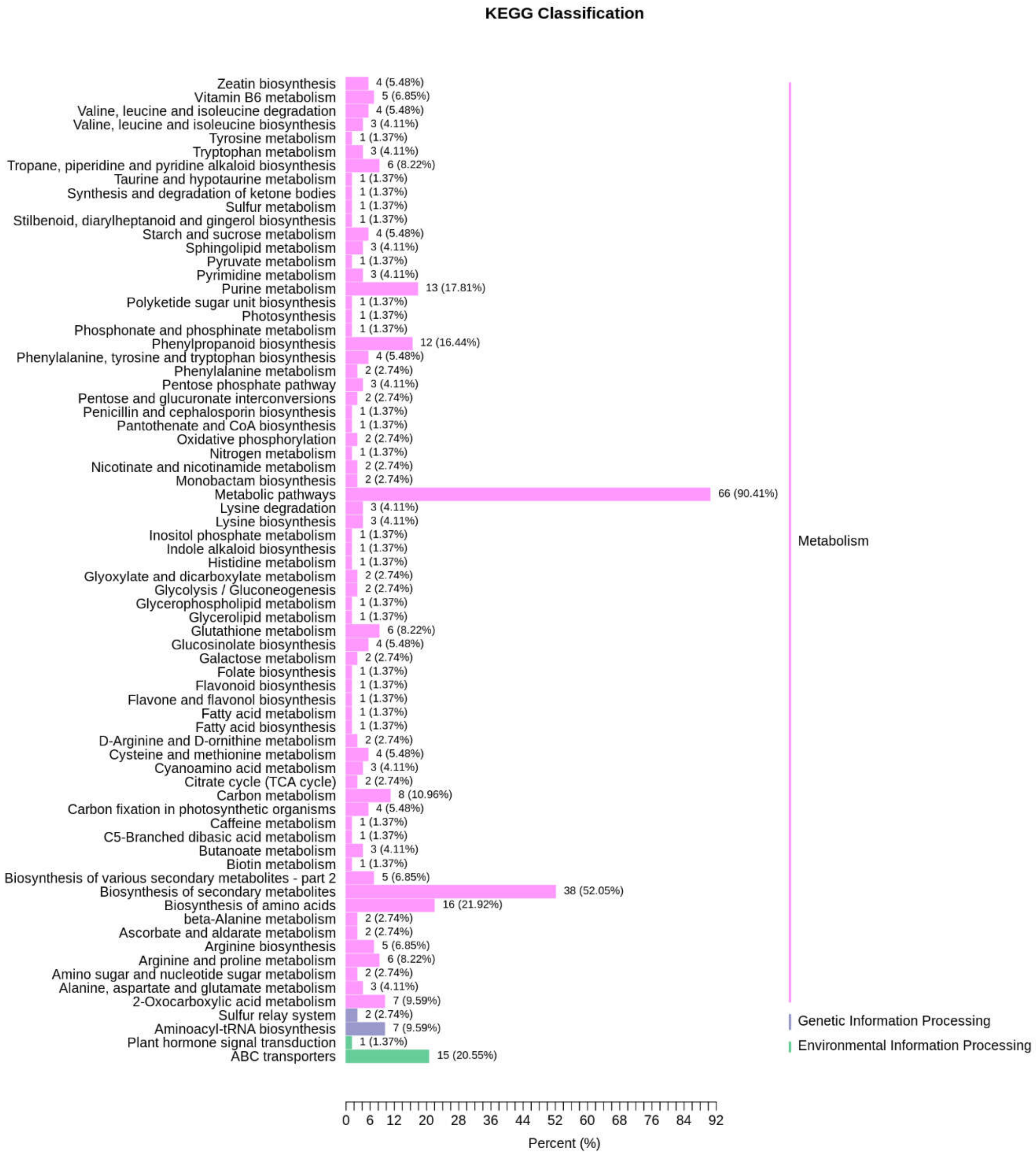

Figure S1: KEGG Barblots between different pineapple species.

TN4_vs_TN21_KEGG_Barplot

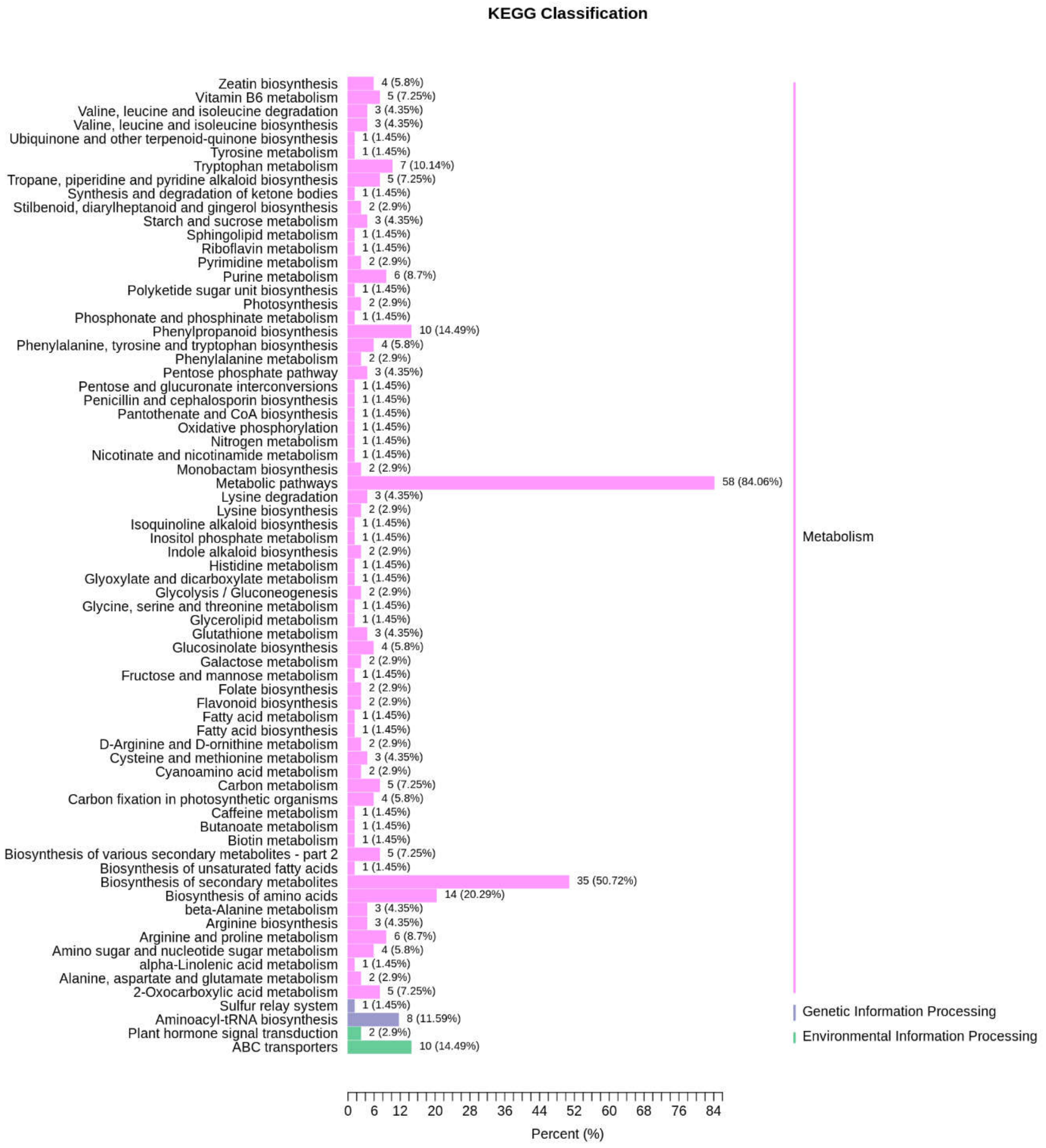

Figure S1: KEGG Barblots between different pineapple species.

TN4_vs_TN23_KEGG_Barplot

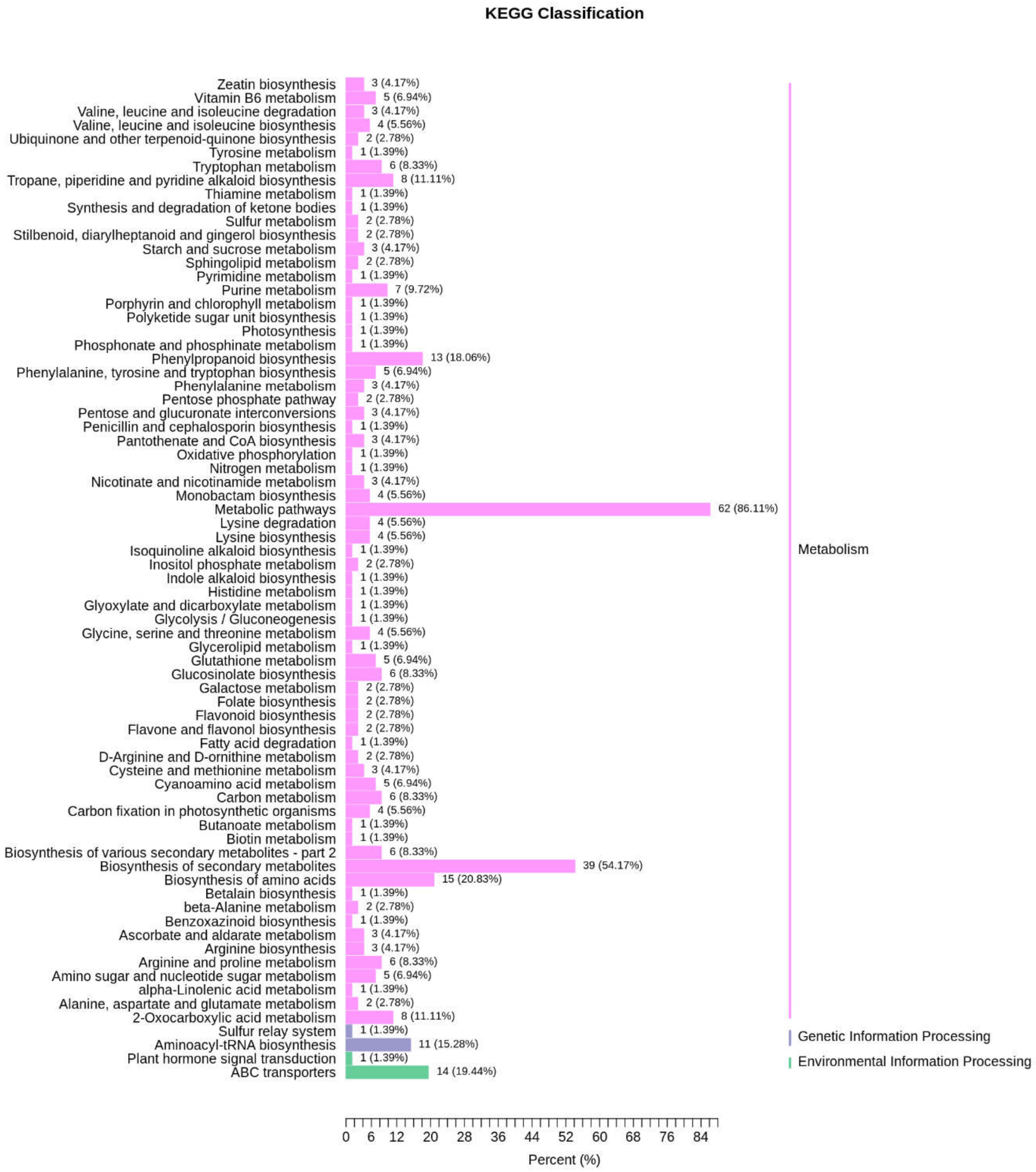

Supplement: Supplementary file 1 [file ijms-24-07199-s001.zip › ijms-2168485-supplementary/Supplementary Files/Figure S1.pdf]
