# Supplementary material for: Integrated Metabolome and Transcriptome Analysis Reveals a Potential Mechanism for Water Accumulation Mediated Translucency in Pineapple (Ananas comosus (L.) Merr.) Fruit
Source: Int J Mol Sci. 2023 Apr 13;24(8):7199. doi: 10.3390/ijms24087199 (PMC10139408; doi:10.3390/ijms24087199)

Figure S2: Metabolome enrichment analysis for among the top 20 annotated KEGG pathways.

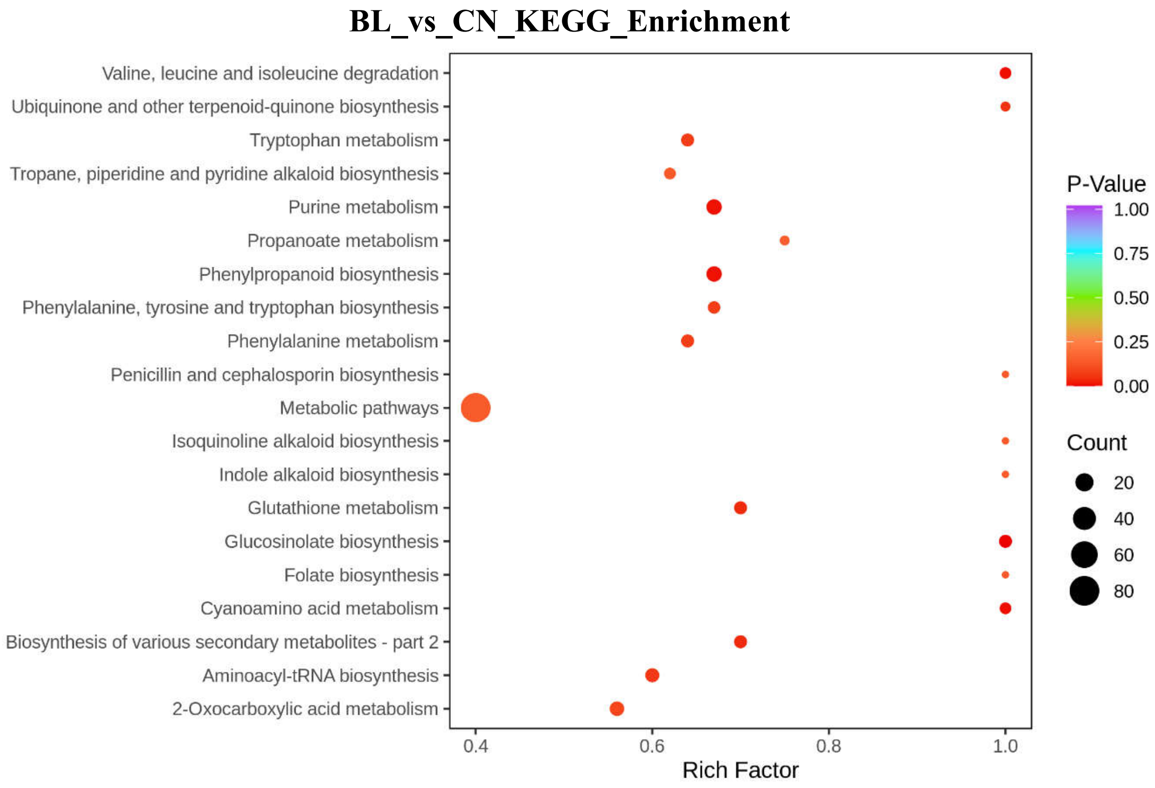

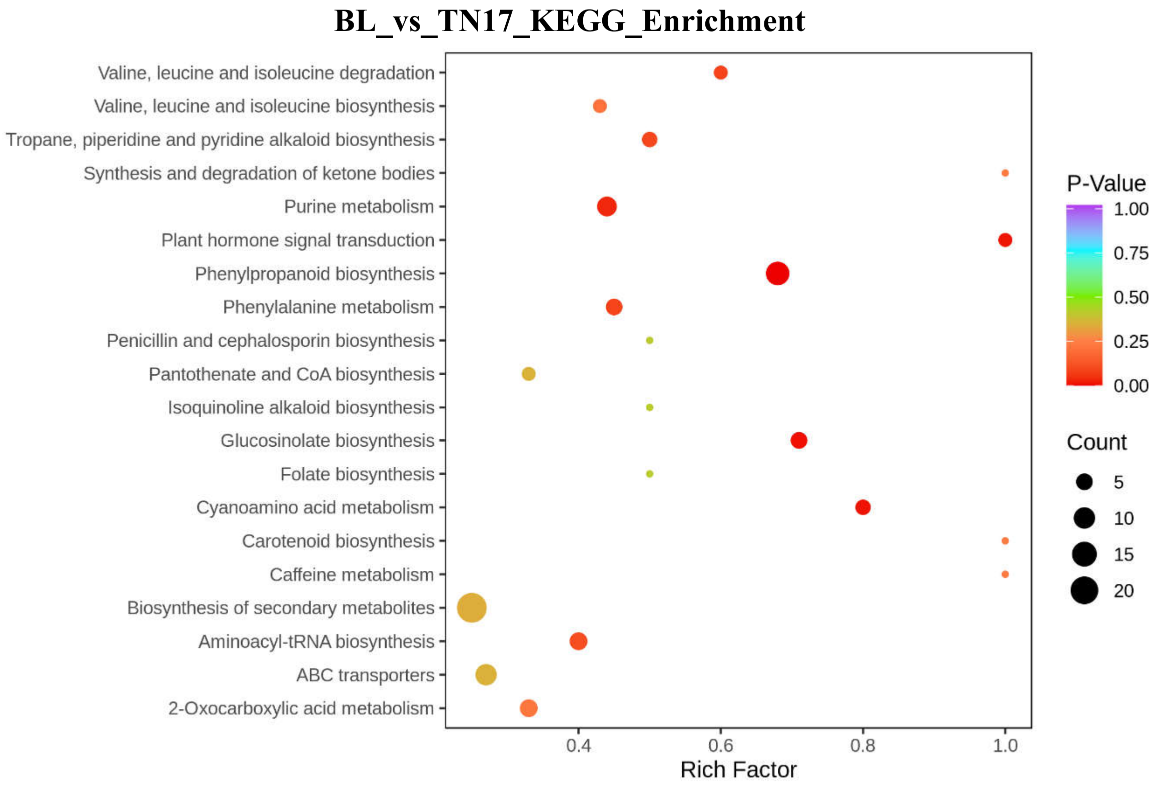

Figure S2: Metabolome enrichment analysis for among the top 20 annotated KEGG pathways.

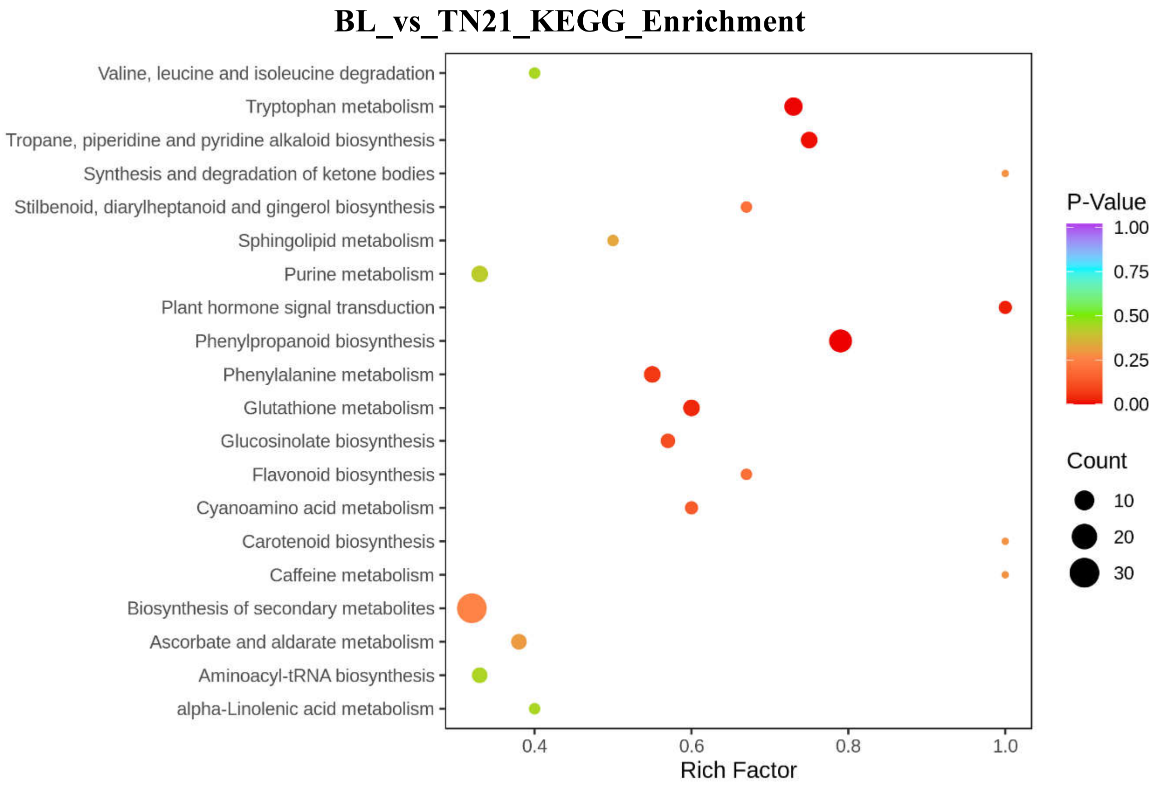

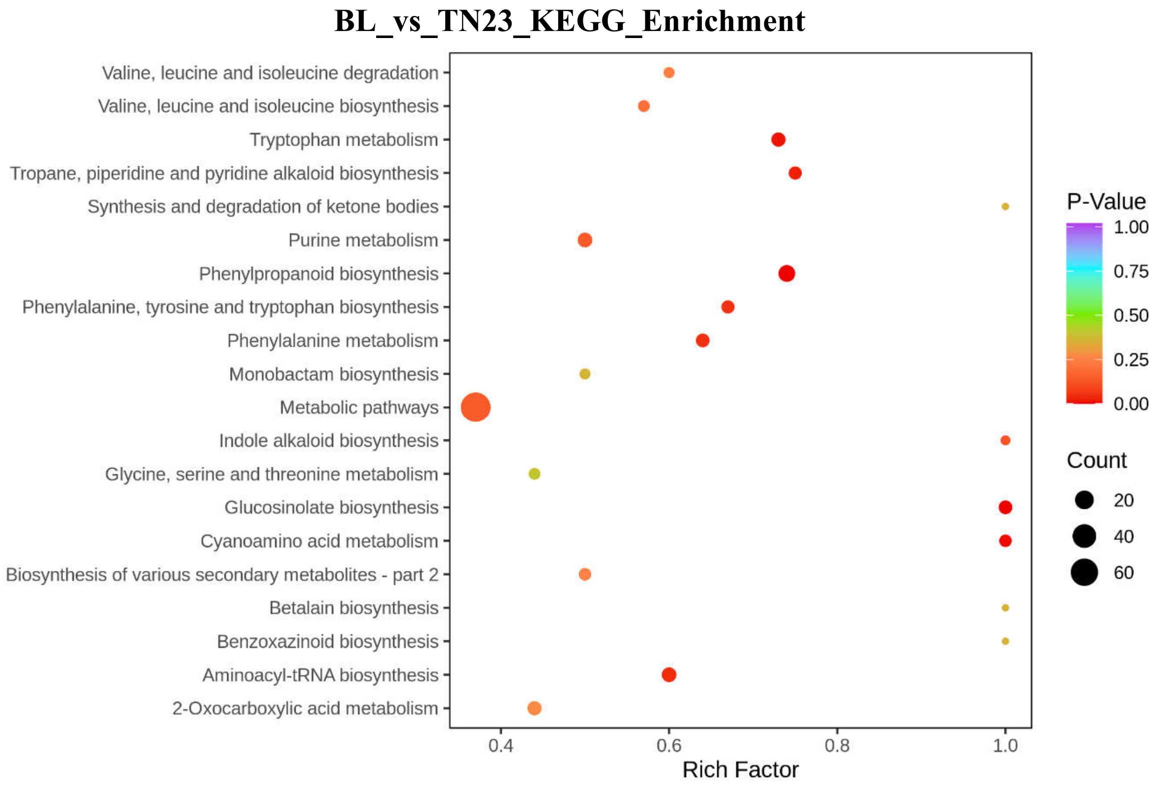

Figure S2: Metabolome enrichment analysis for among the top 20 annotated KEGG pathways.

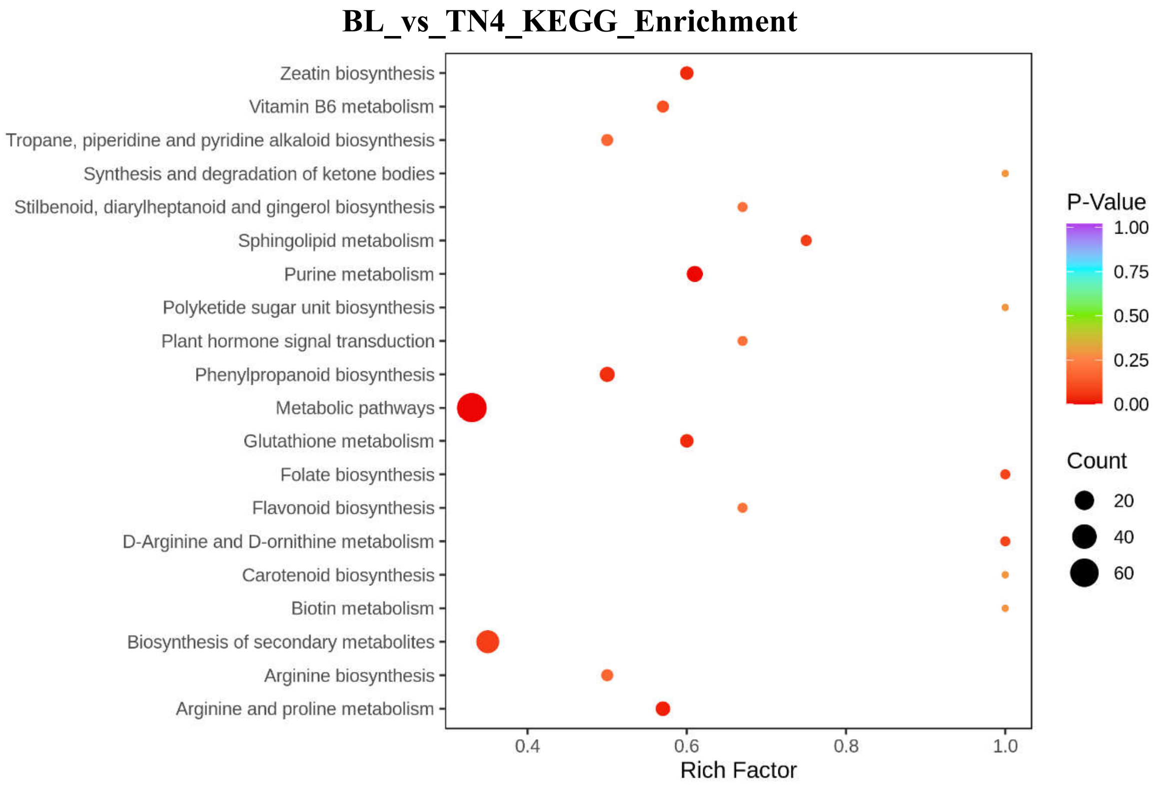

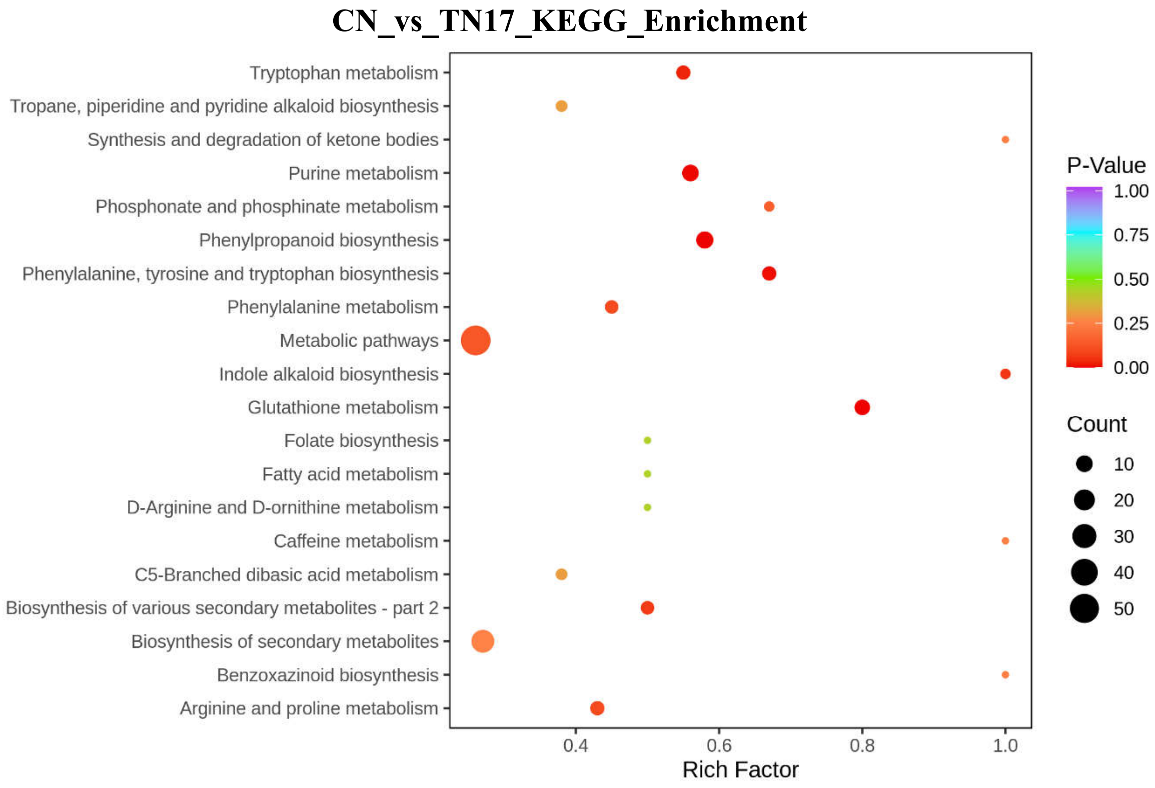

Figure S2: Metabolome enrichment analysis for among the top 20 annotated KEGG pathways.

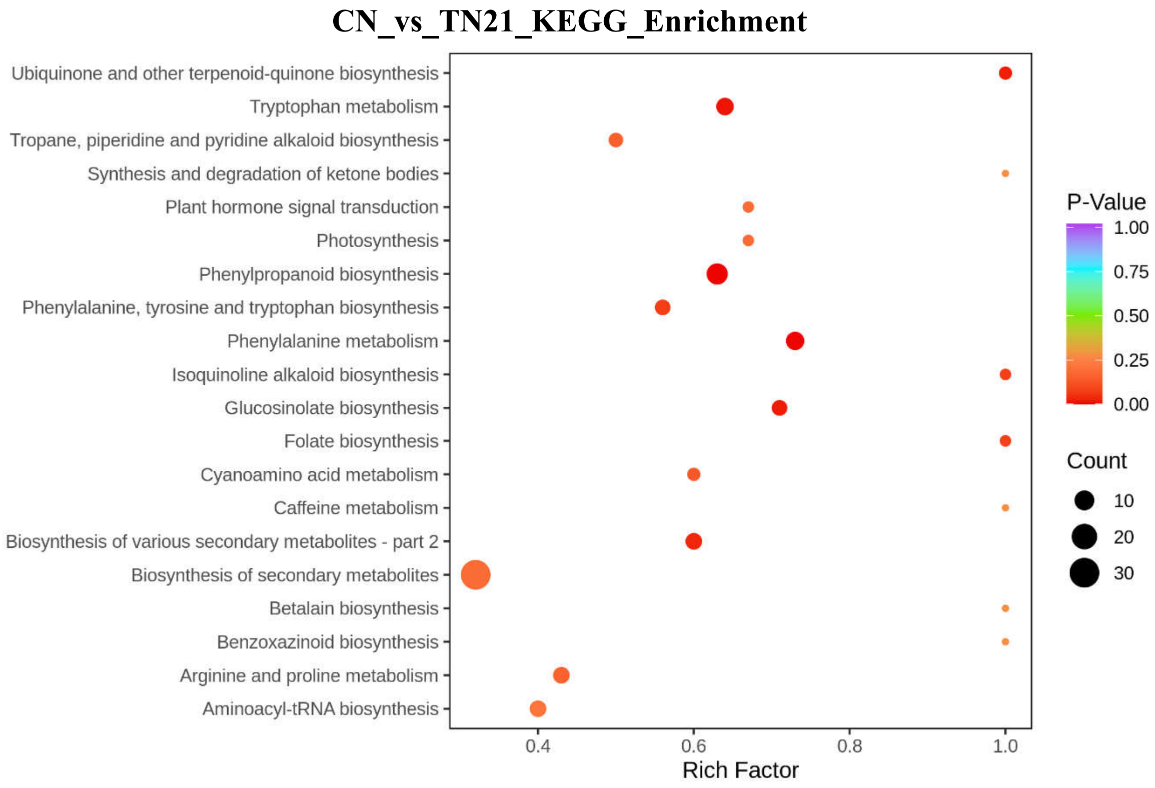

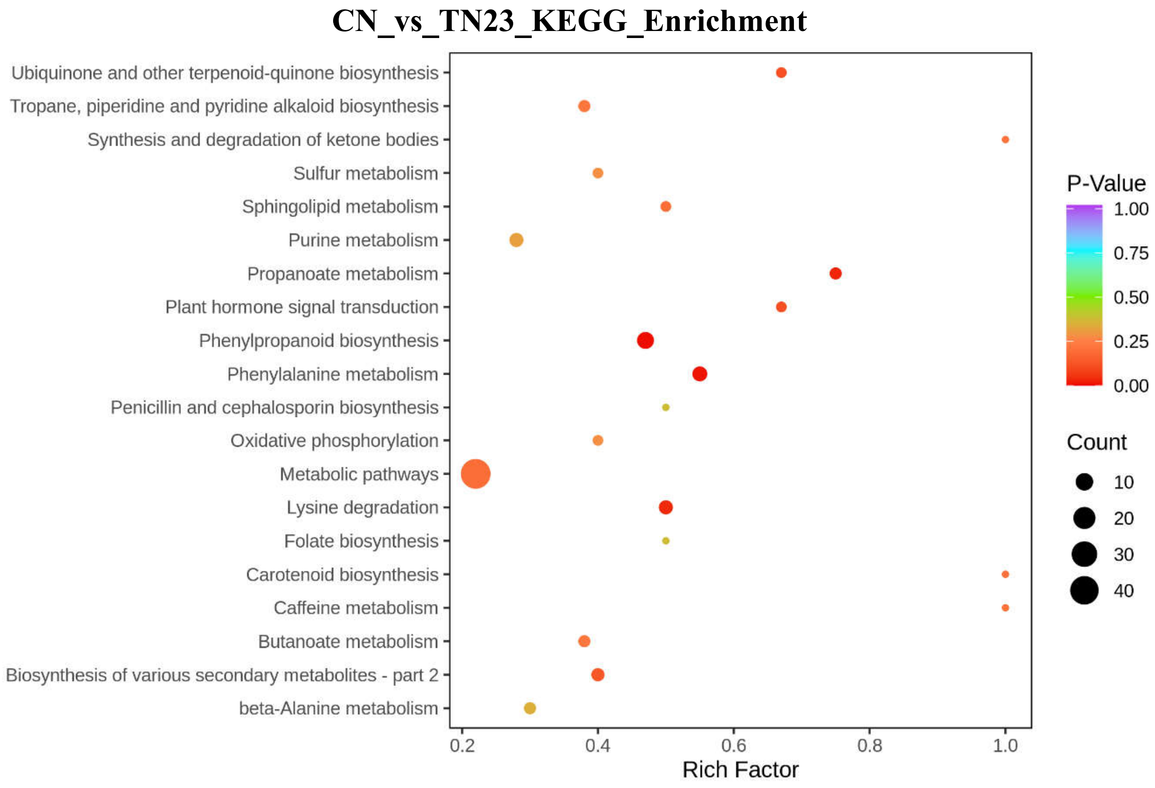

Figure S2: Metabolome enrichment analysis for among the top 20 annotated KEGG pathways.

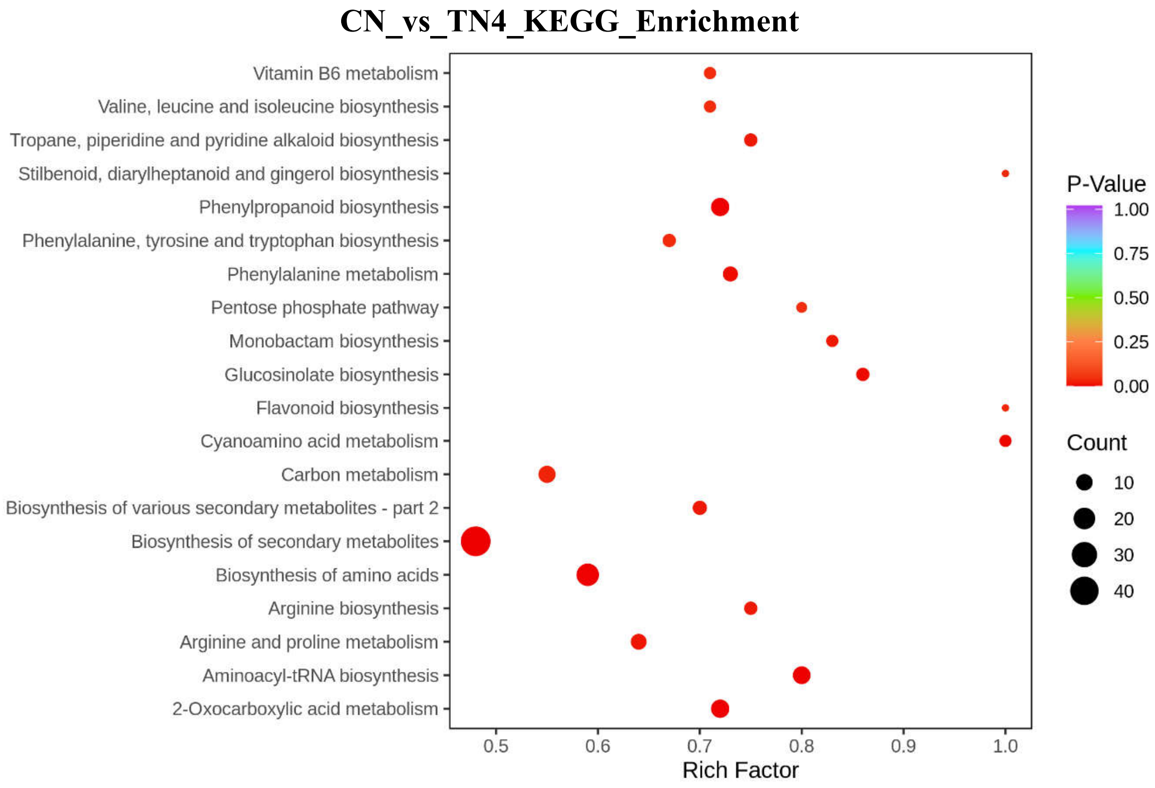

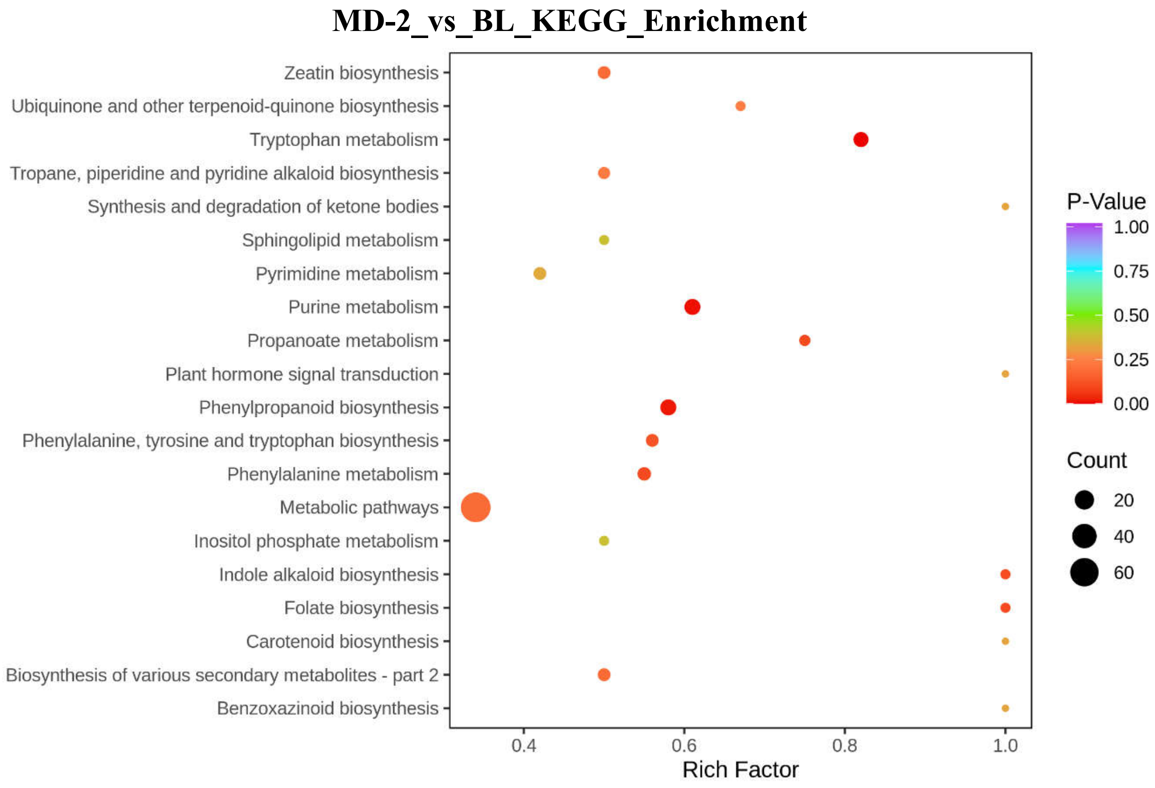

Figure S2: Metabolome enrichment analysis for among the top 20 annotated KEGG pathways.

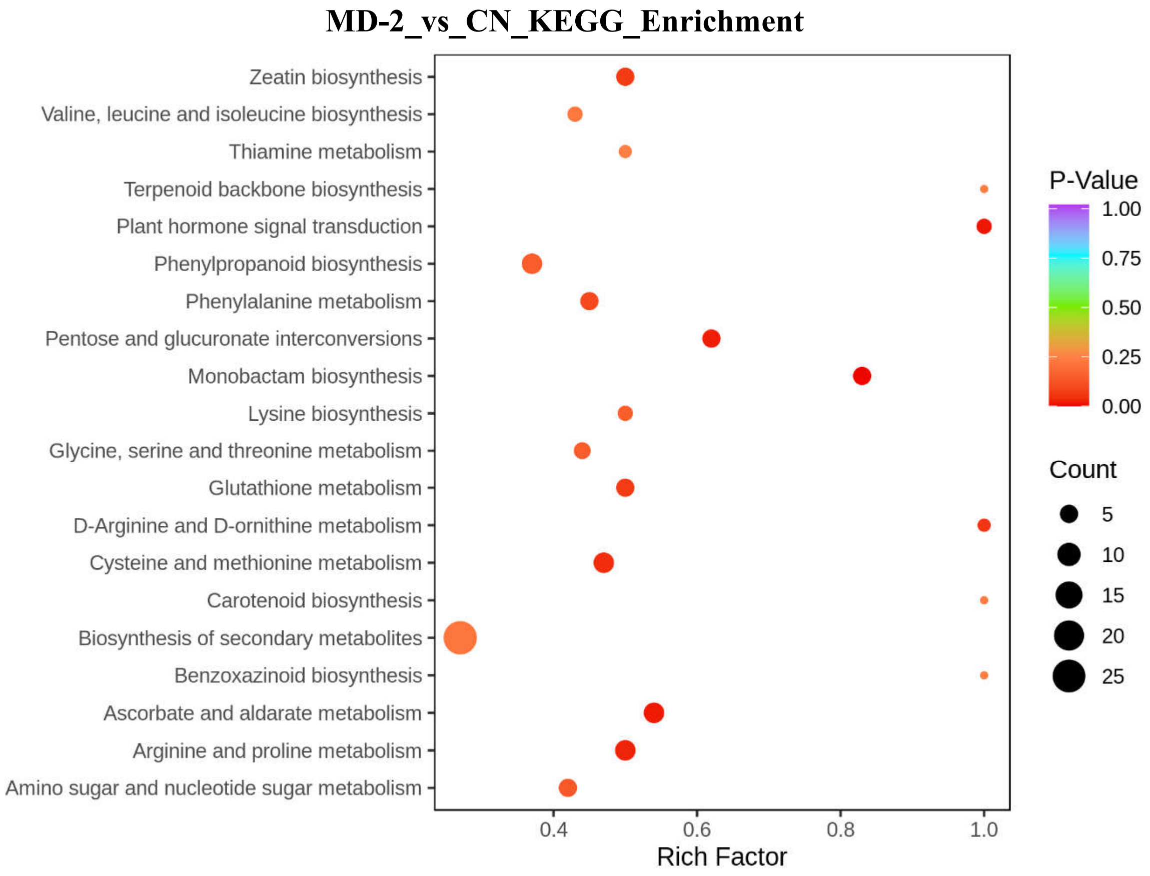

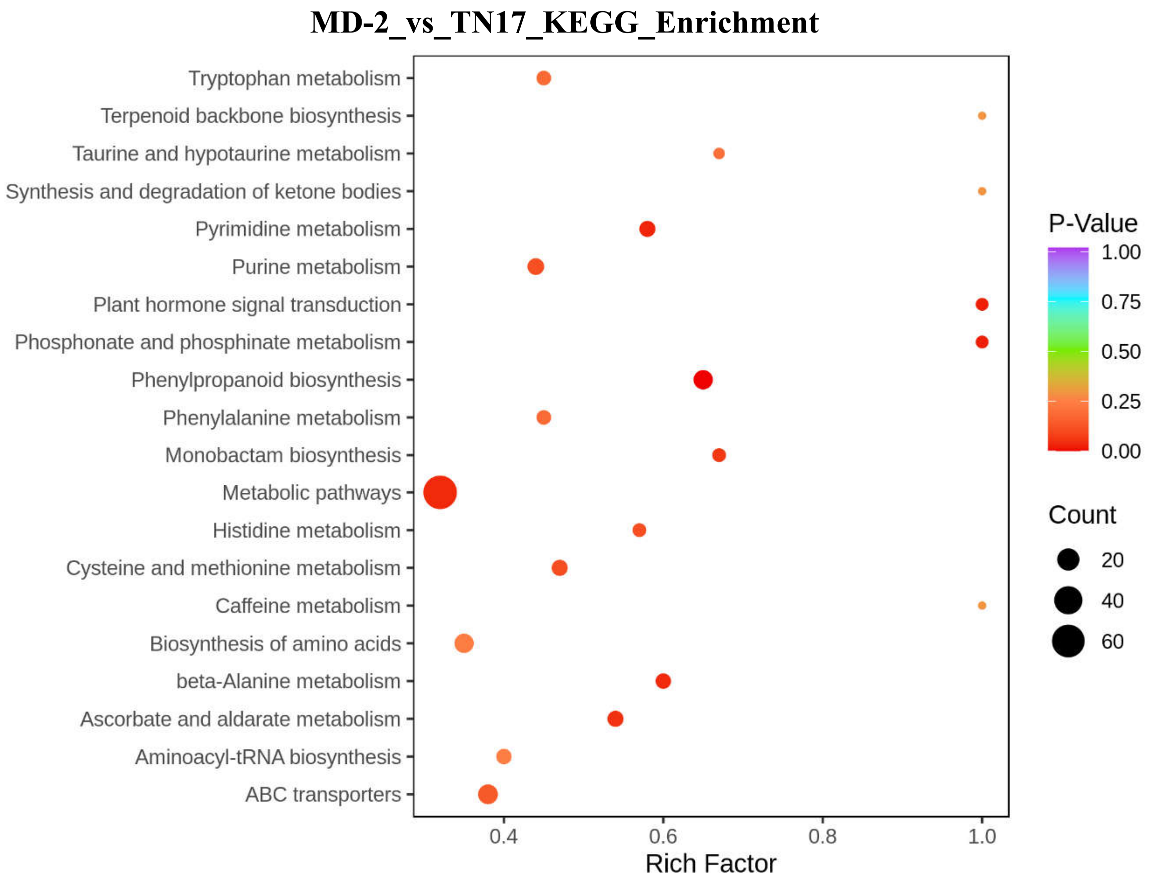

Figure S2: Metabolome enrichment analysis for among the top 20 annotated KEGG pathways.

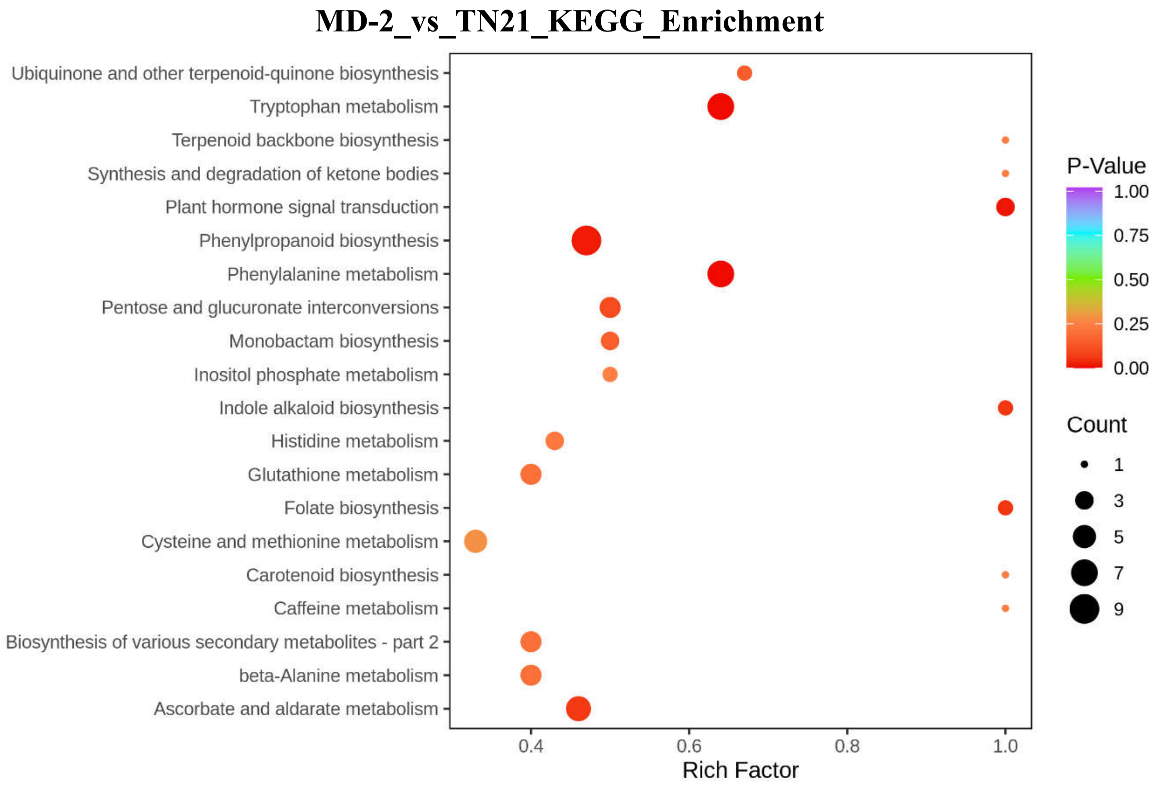

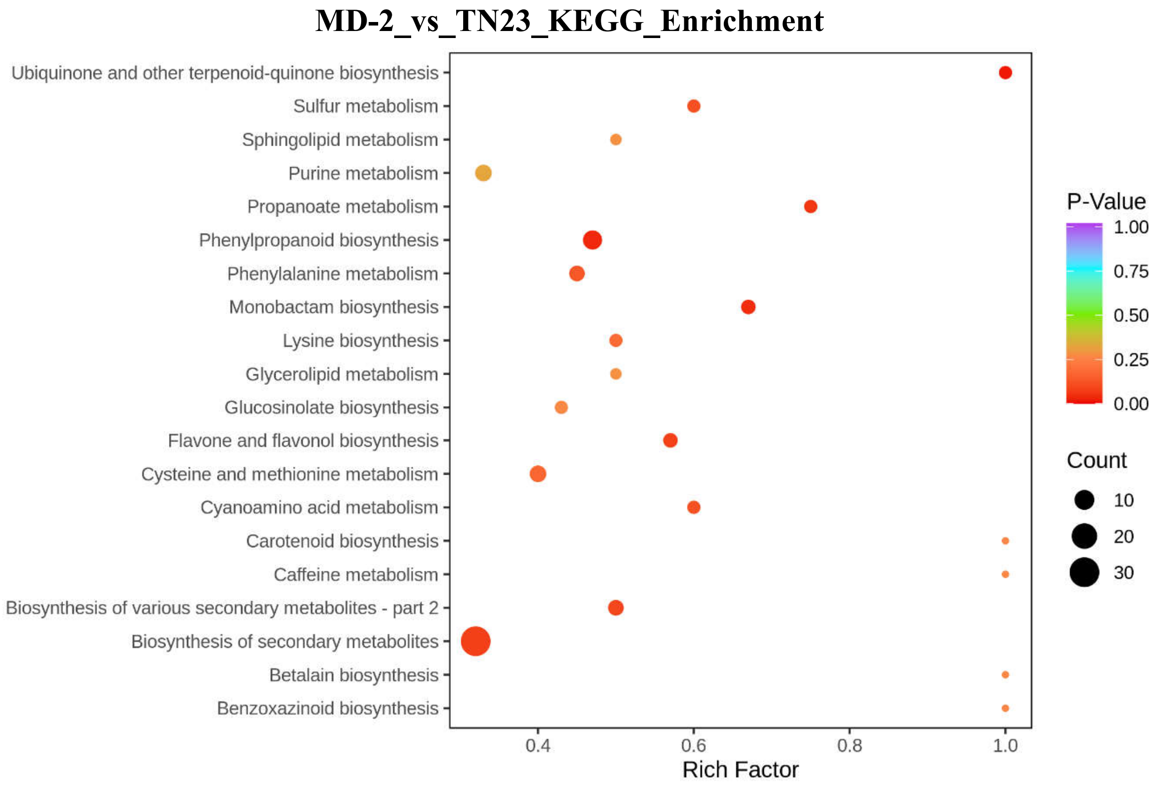

Figure S2: Metabolome enrichment analysis for among the top 20 annotated KEGG pathways.

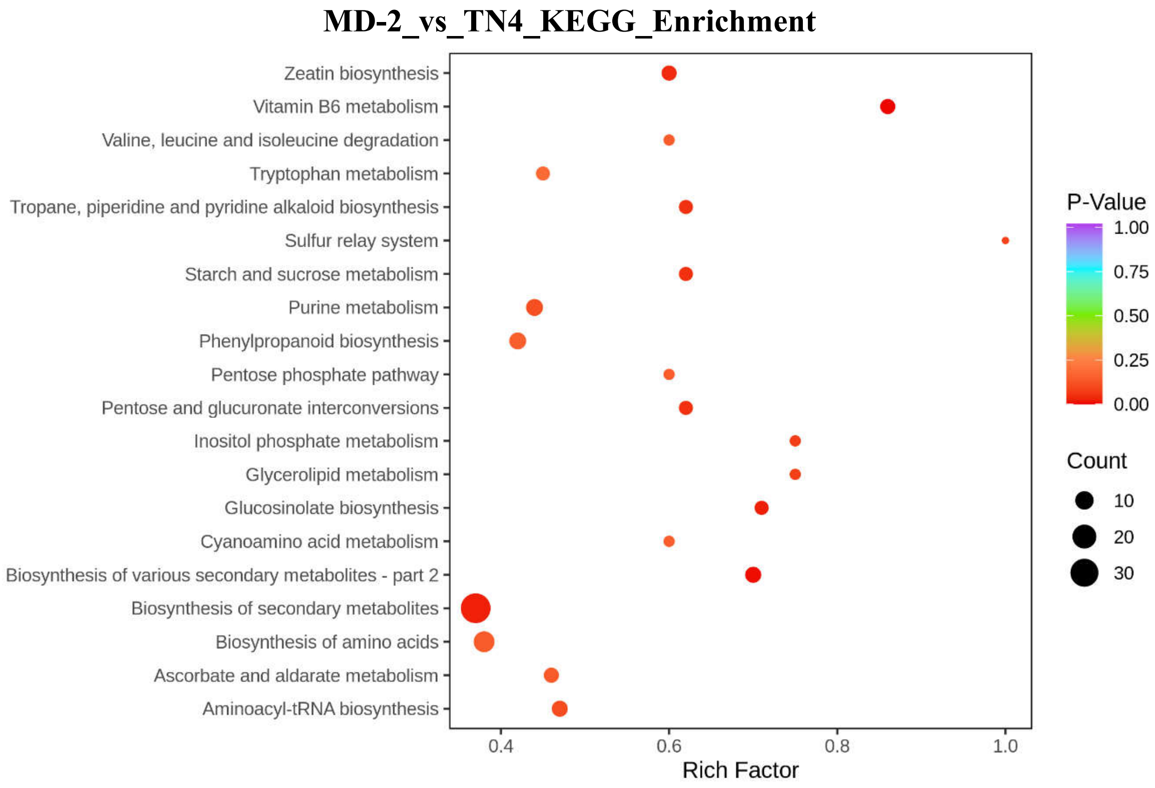

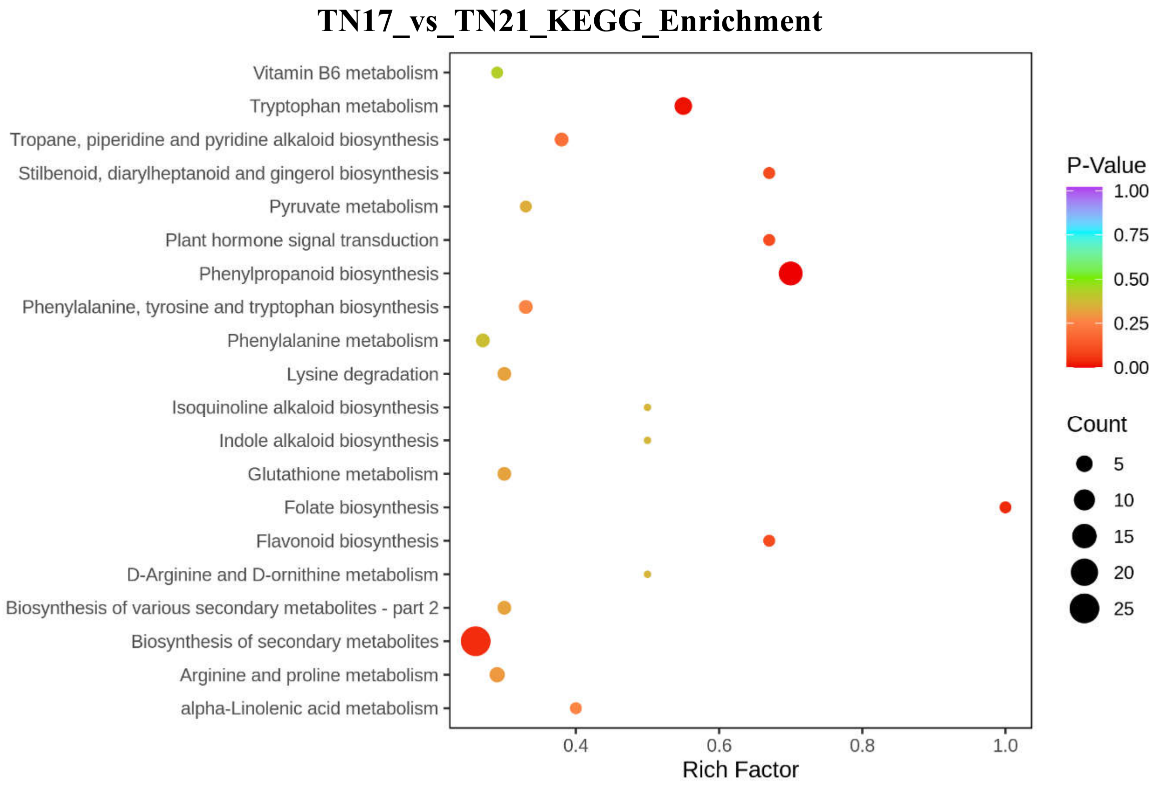

Figure S2: Metabolome enrichment analysis for among the top 20 annotated KEGG pathways.

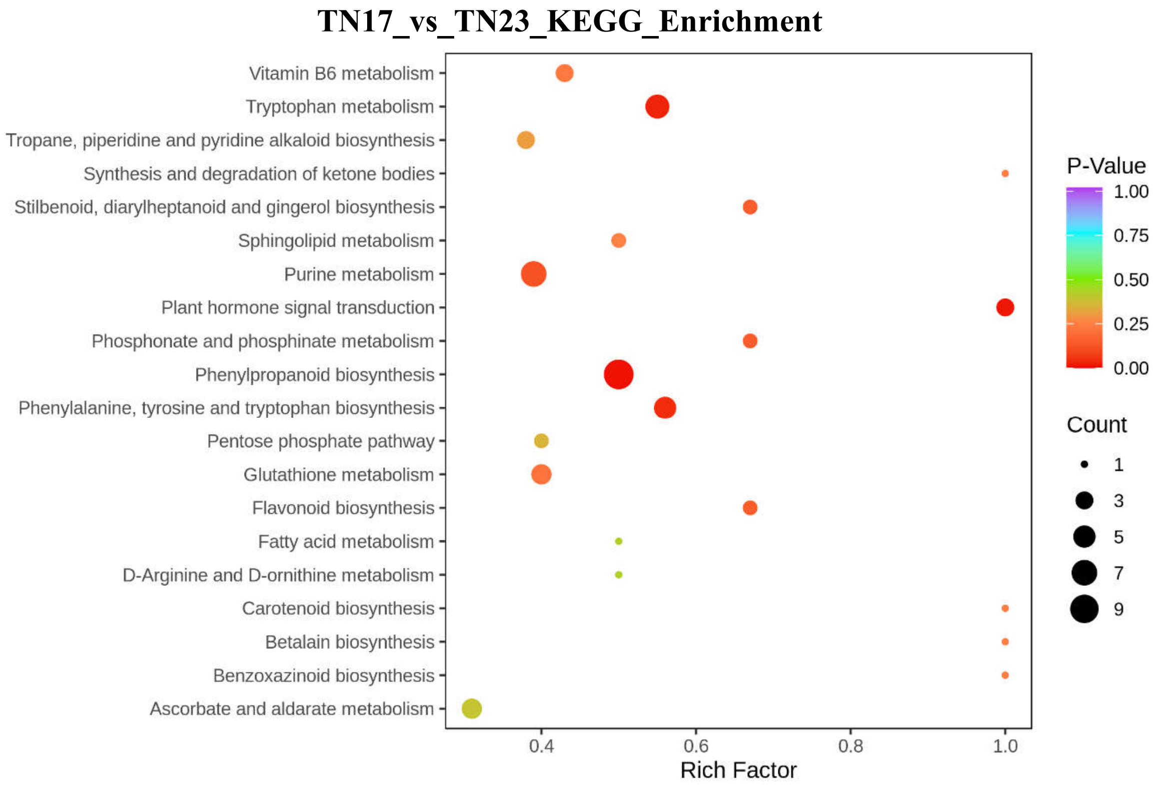

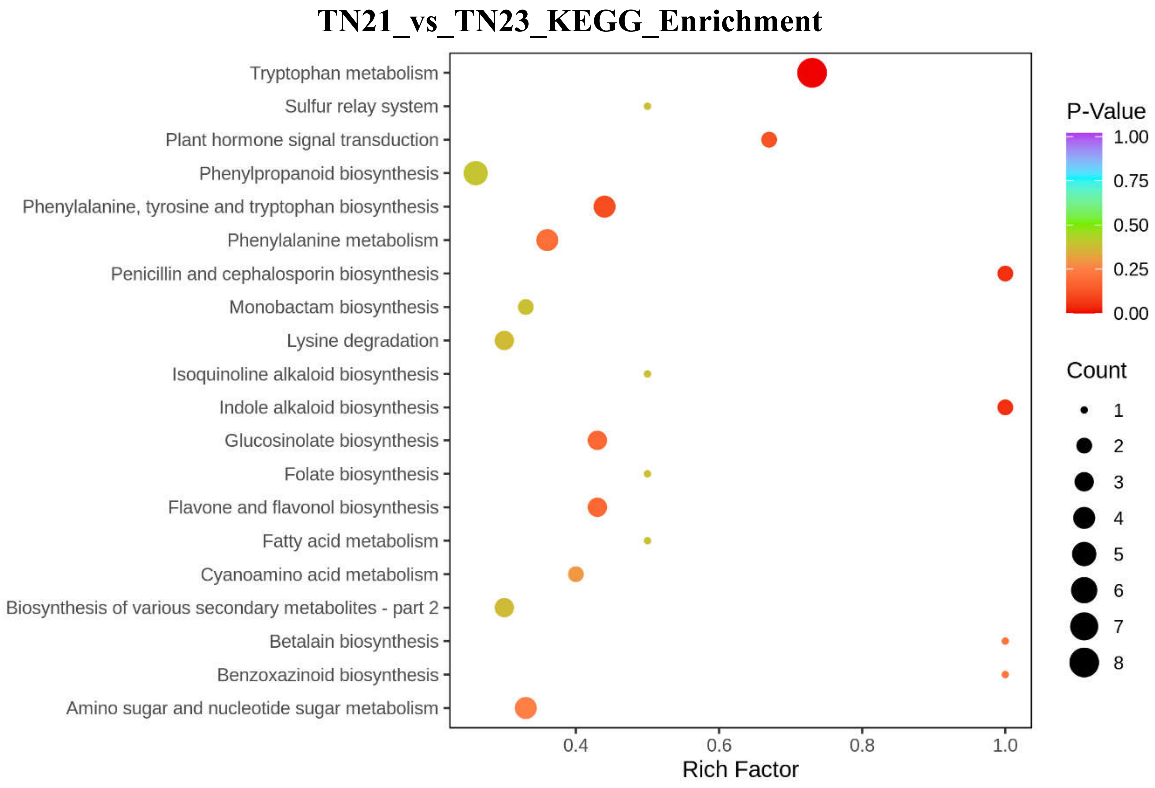

Figure S2: Metabolome enrichment analysis for among the top 20 annotated KEGG pathways.

TN4_vs_TN17_KEGG_Enrichment

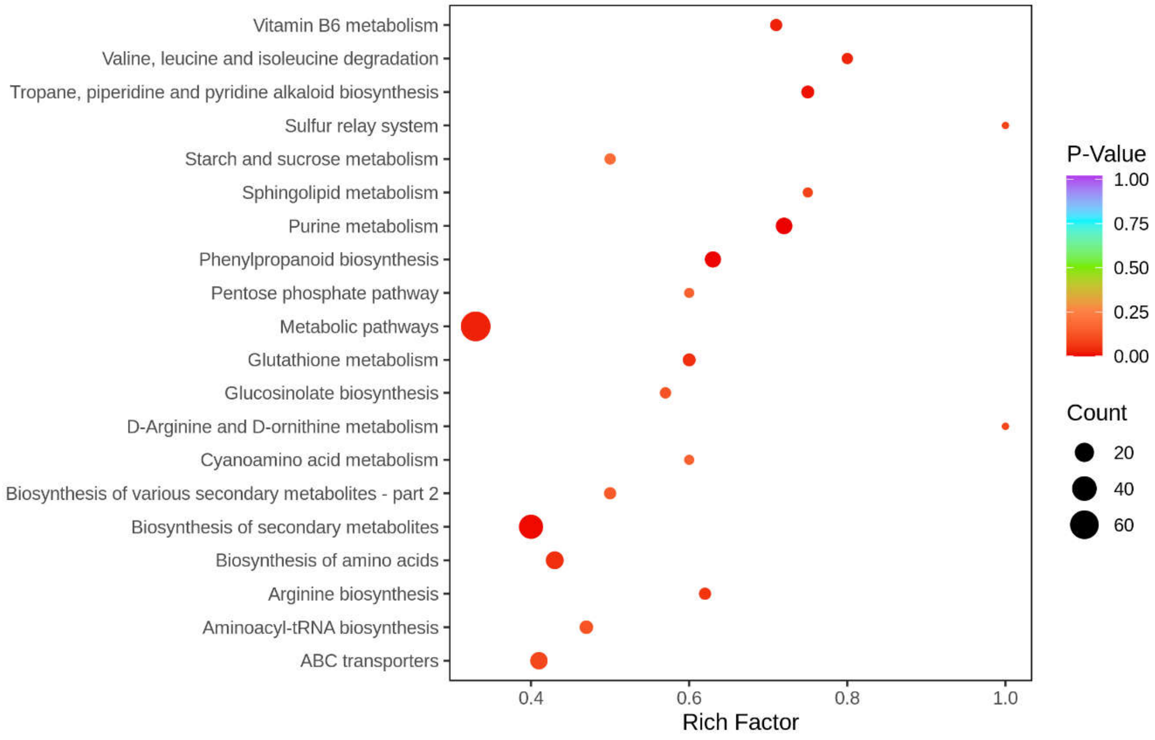

TN4_vs_TN21_KEGG_Enrichment

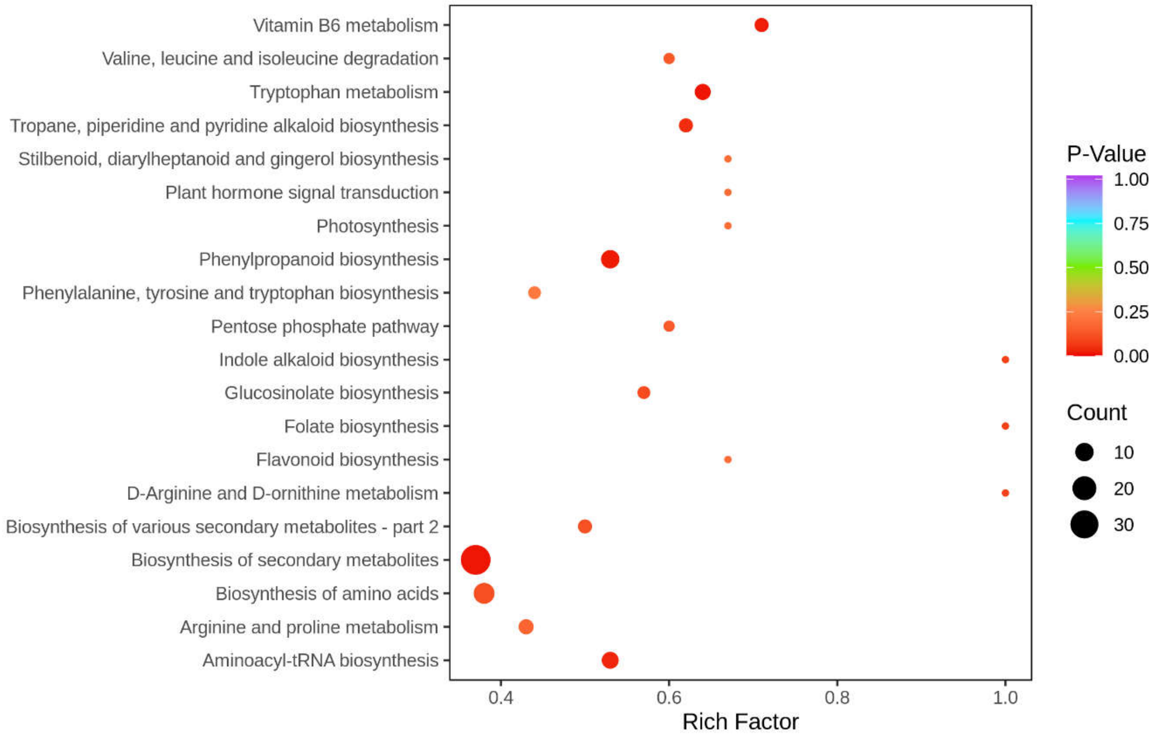

Figure S2: Metabolome enrichment analysis for among the top 20 annotated KEGG pathways.

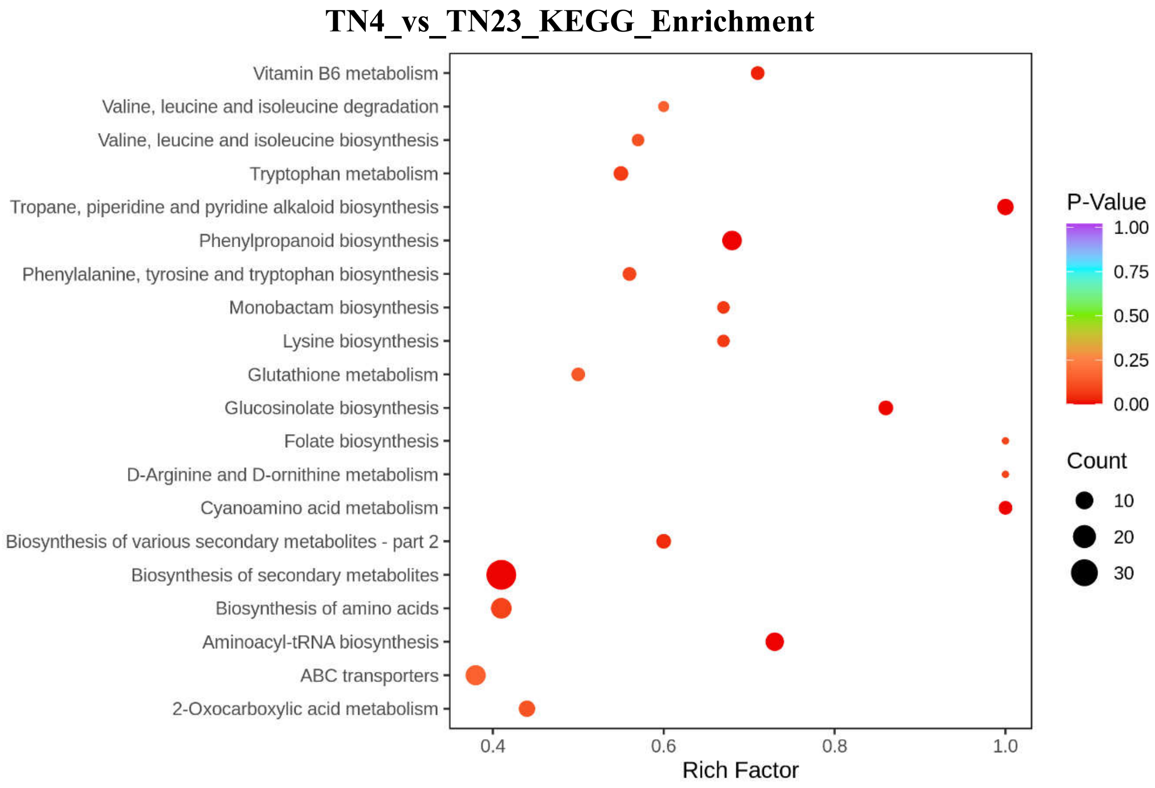

Supplement: Supplementary file 1 [file ijms-24-07199-s001.zip › ijms-2168485-supplementary/Supplementary Files/Figure S2.pdf]
