# Supplementary material for: Integrated Metabolome and Transcriptome Analysis Reveals a Potential Mechanism for Water Accumulation Mediated Translucency in Pineapple (Ananas comosus (L.) Merr.) Fruit
Source: Int J Mol Sci. 2023 Apr 13;24(8):7199. doi: 10.3390/ijms24087199 (PMC10139408; doi:10.3390/ijms24087199)

Figure S3: The volcano graph of DEG between different pineapple species

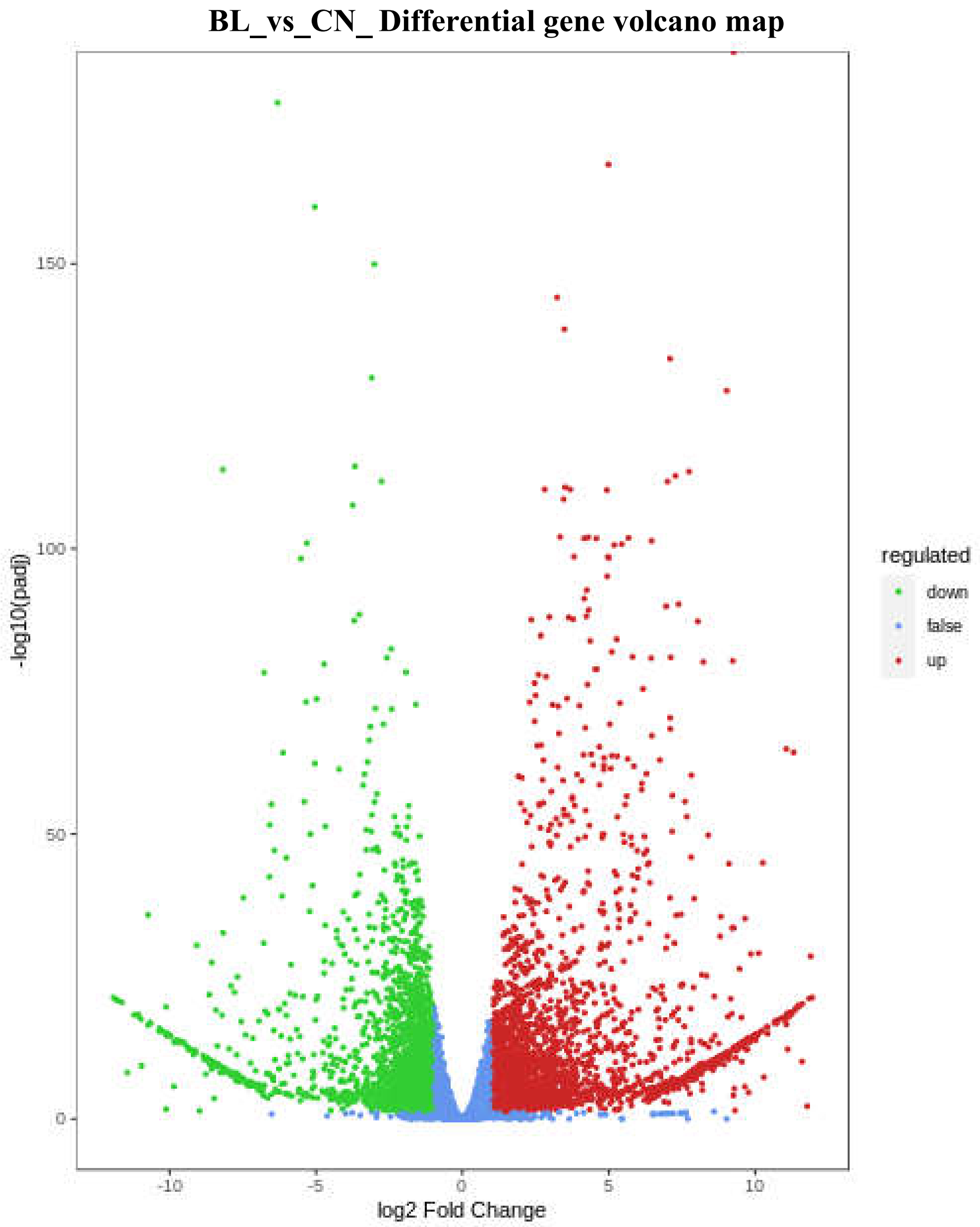

Figure S3: The volcano graph of DEG between different pineapple species

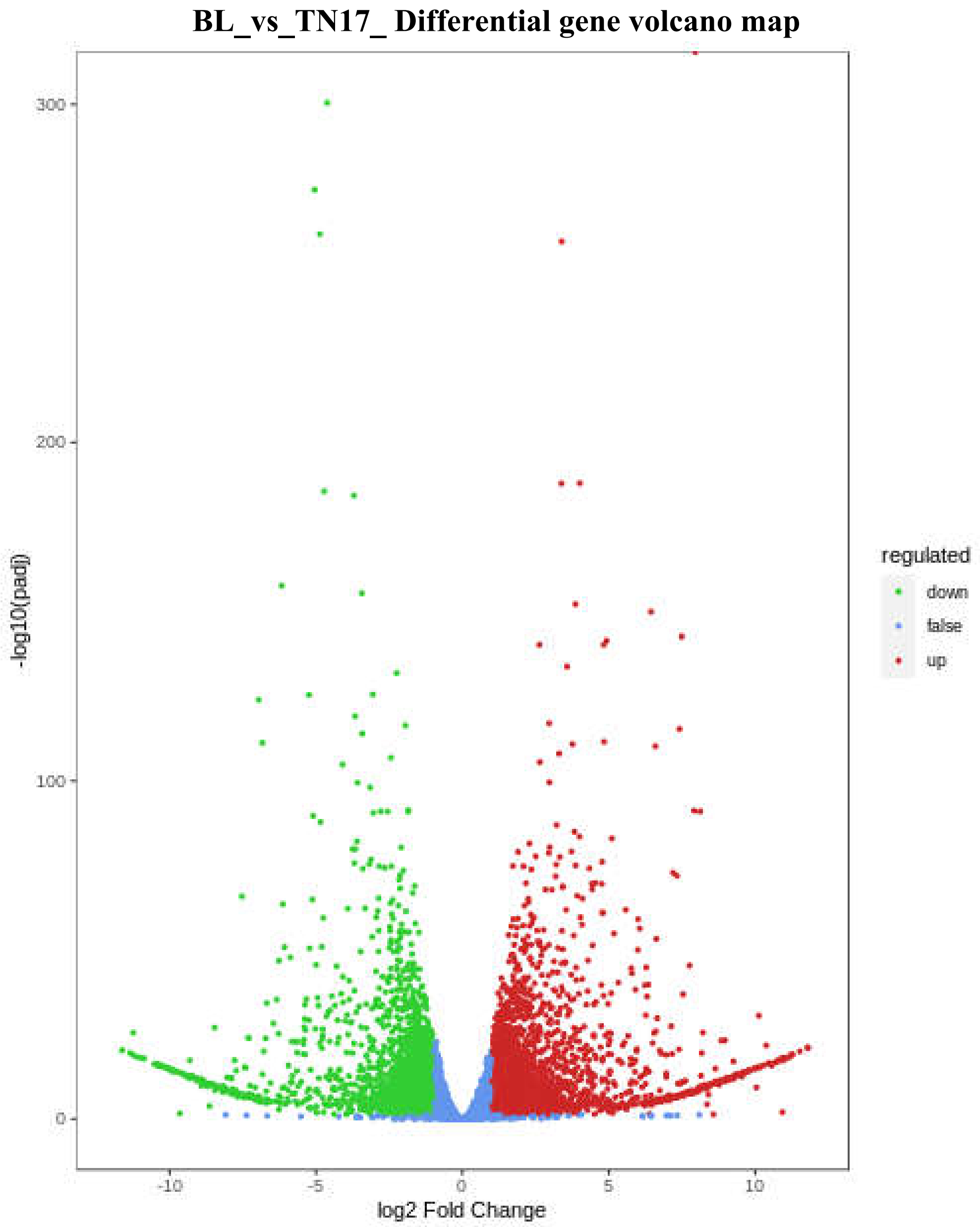

Figure S3: The volcano graph of DEG between different pineapple species

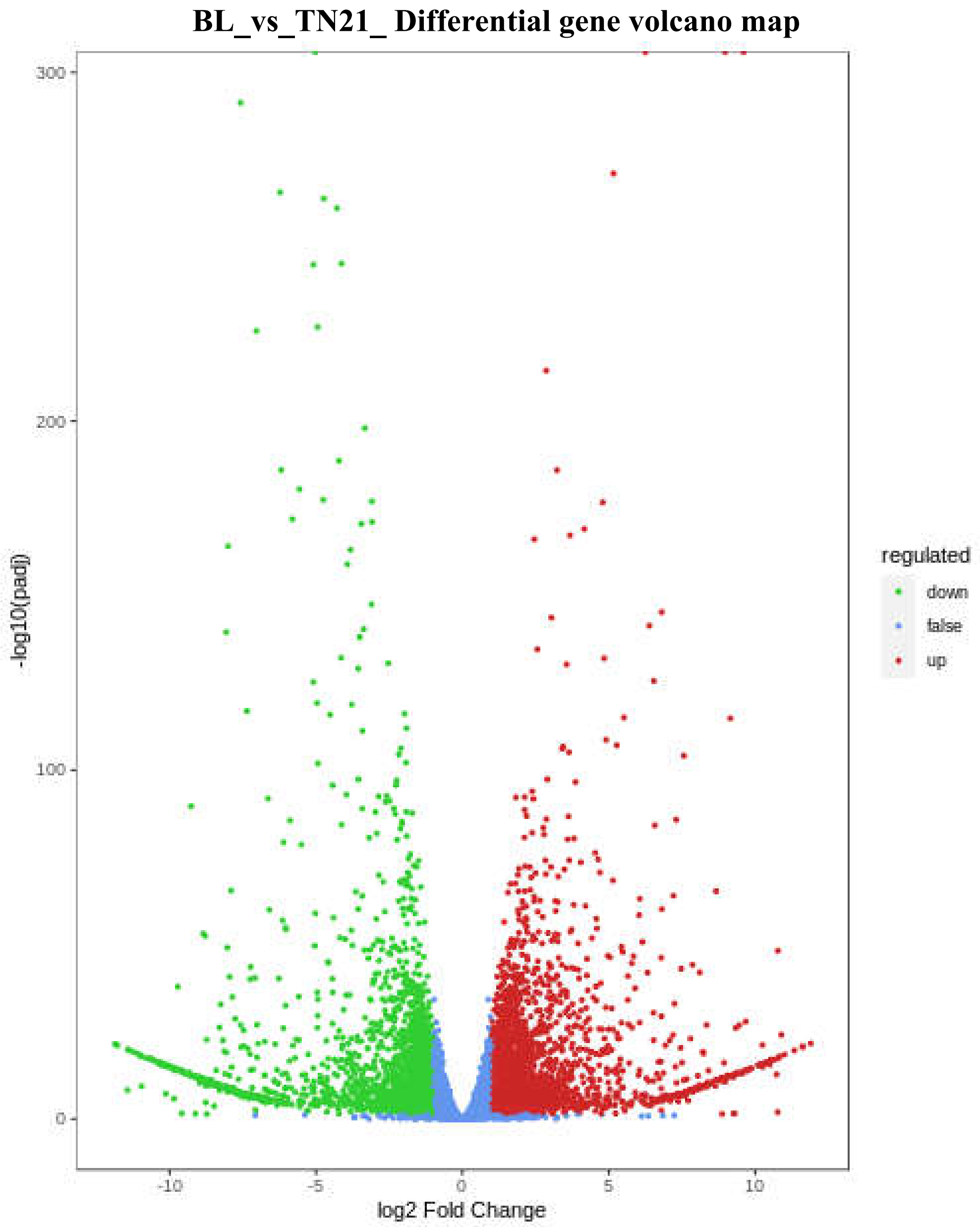

Figure S3: The volcano graph of DEG between different pineapple species

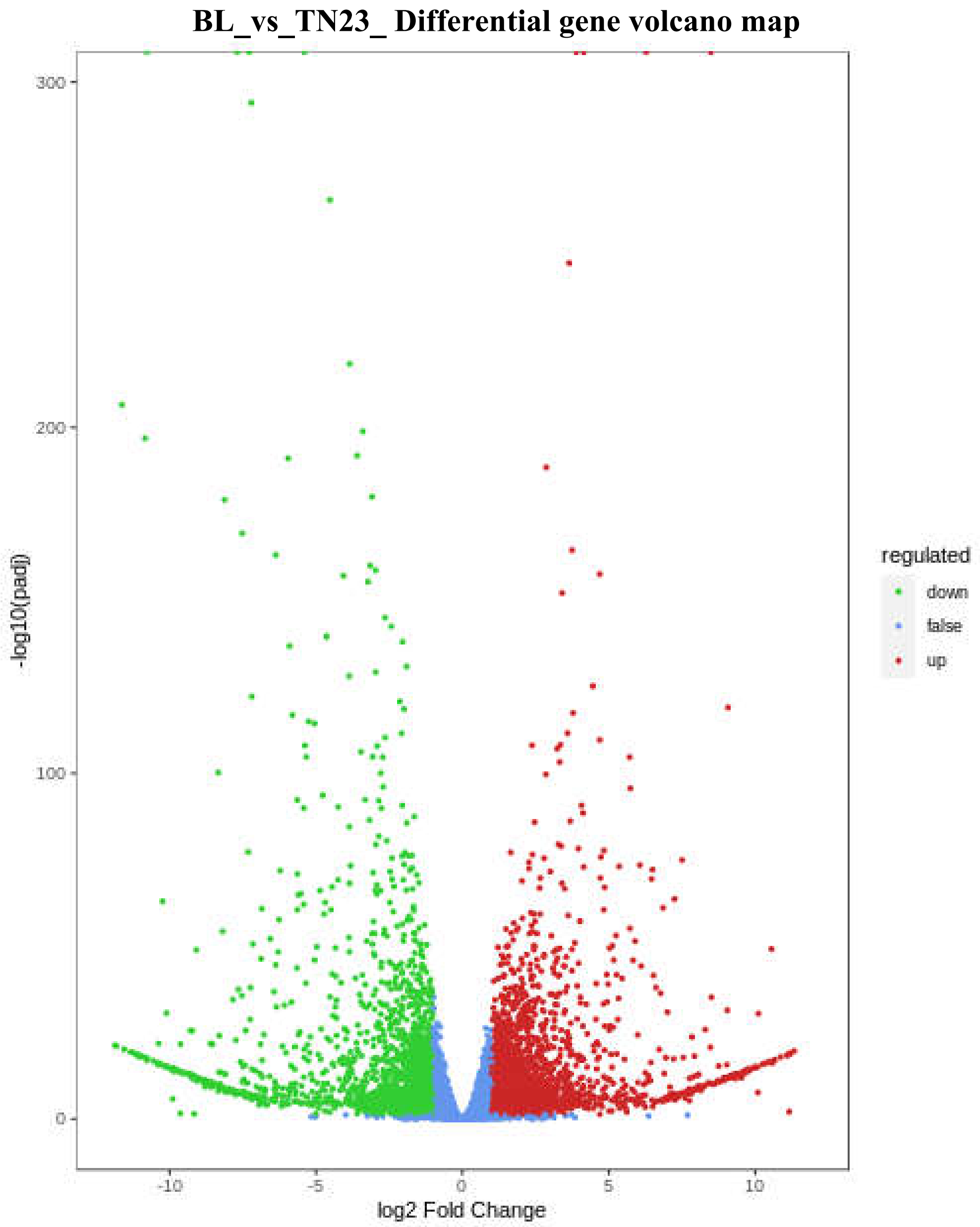

Figure S3: The volcano graph of DEG between different pineapple species

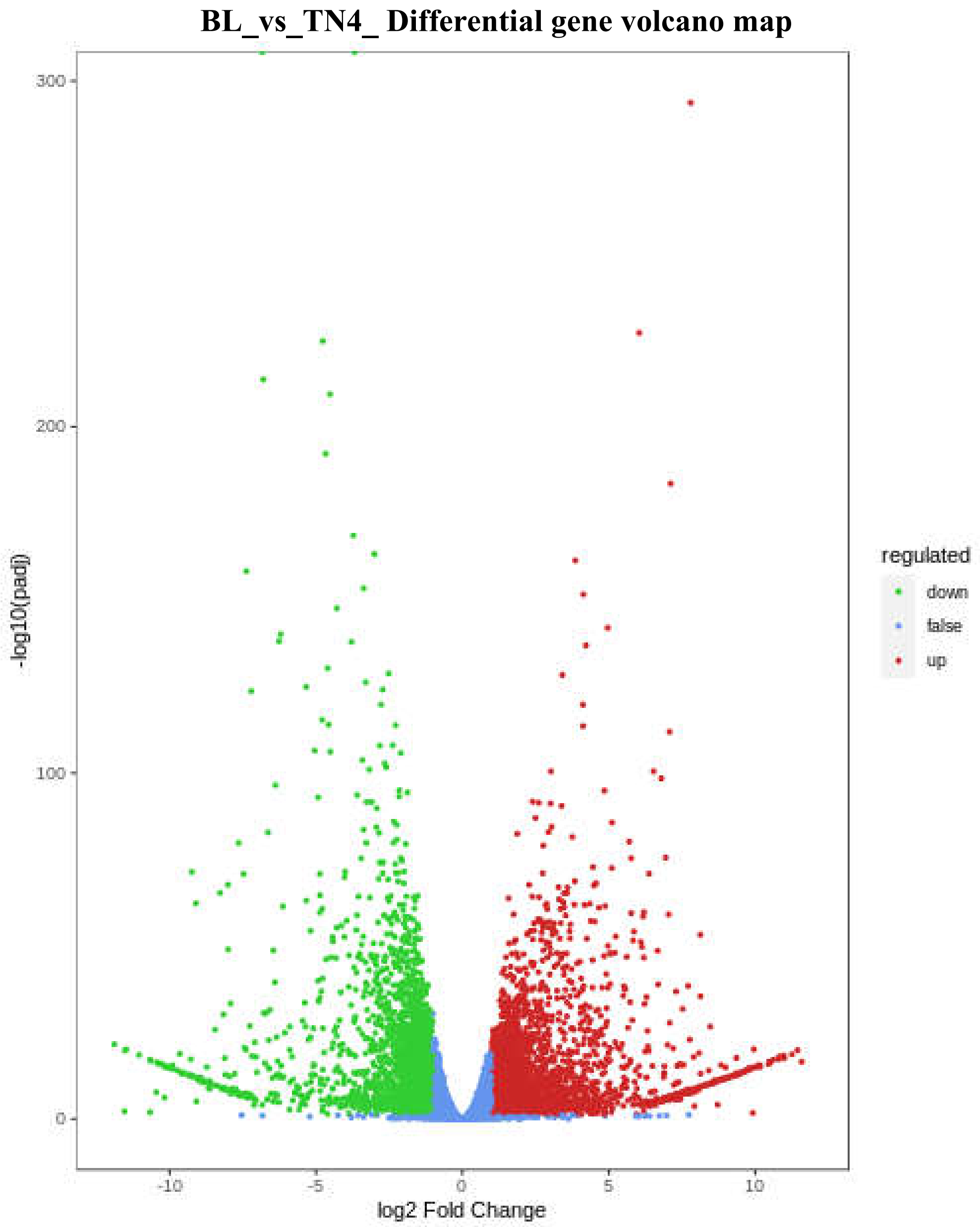

Figure S3: The volcano graph of DEG between different pineapple species

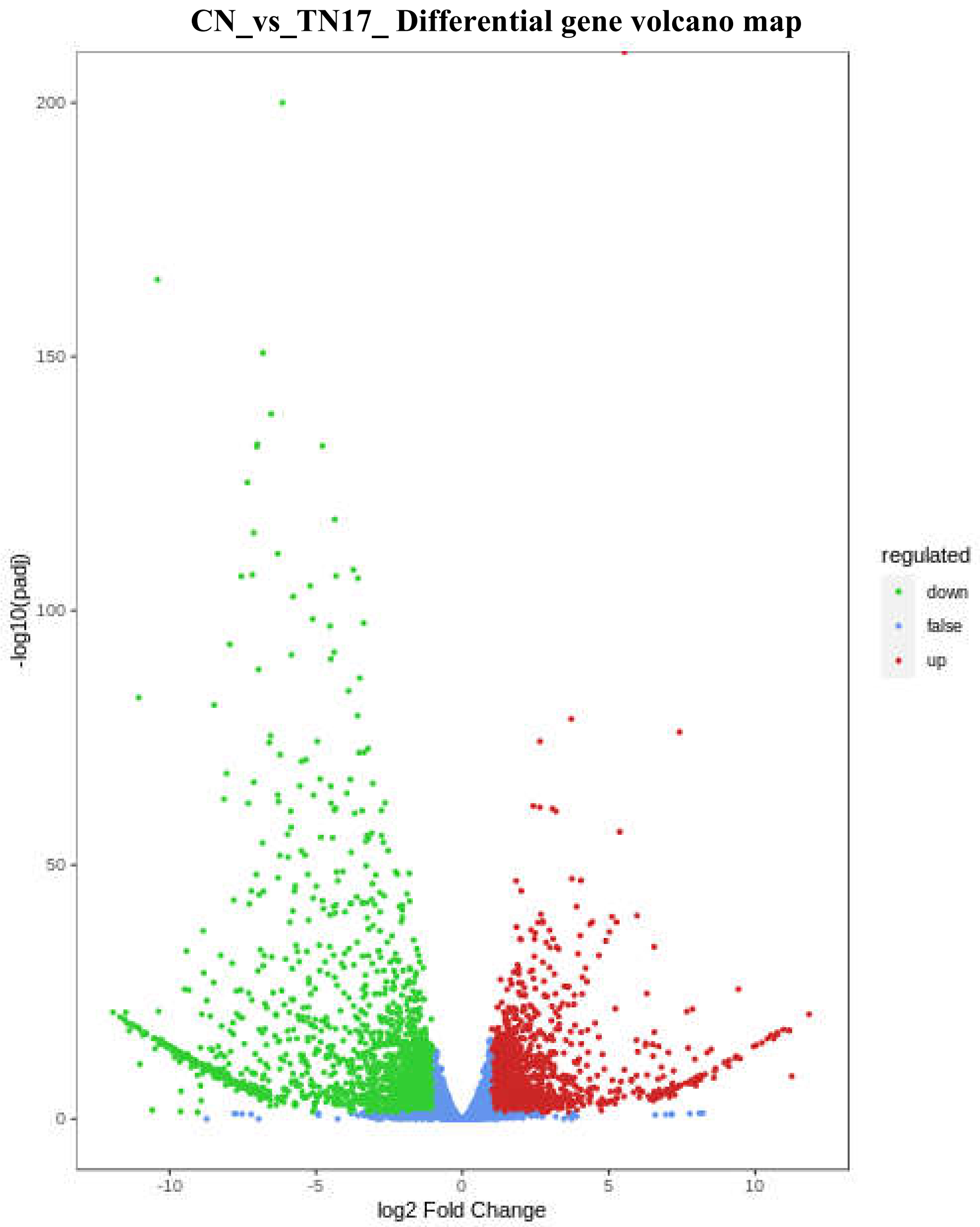

Figure S3: The volcano graph of DEG between different pineapple species

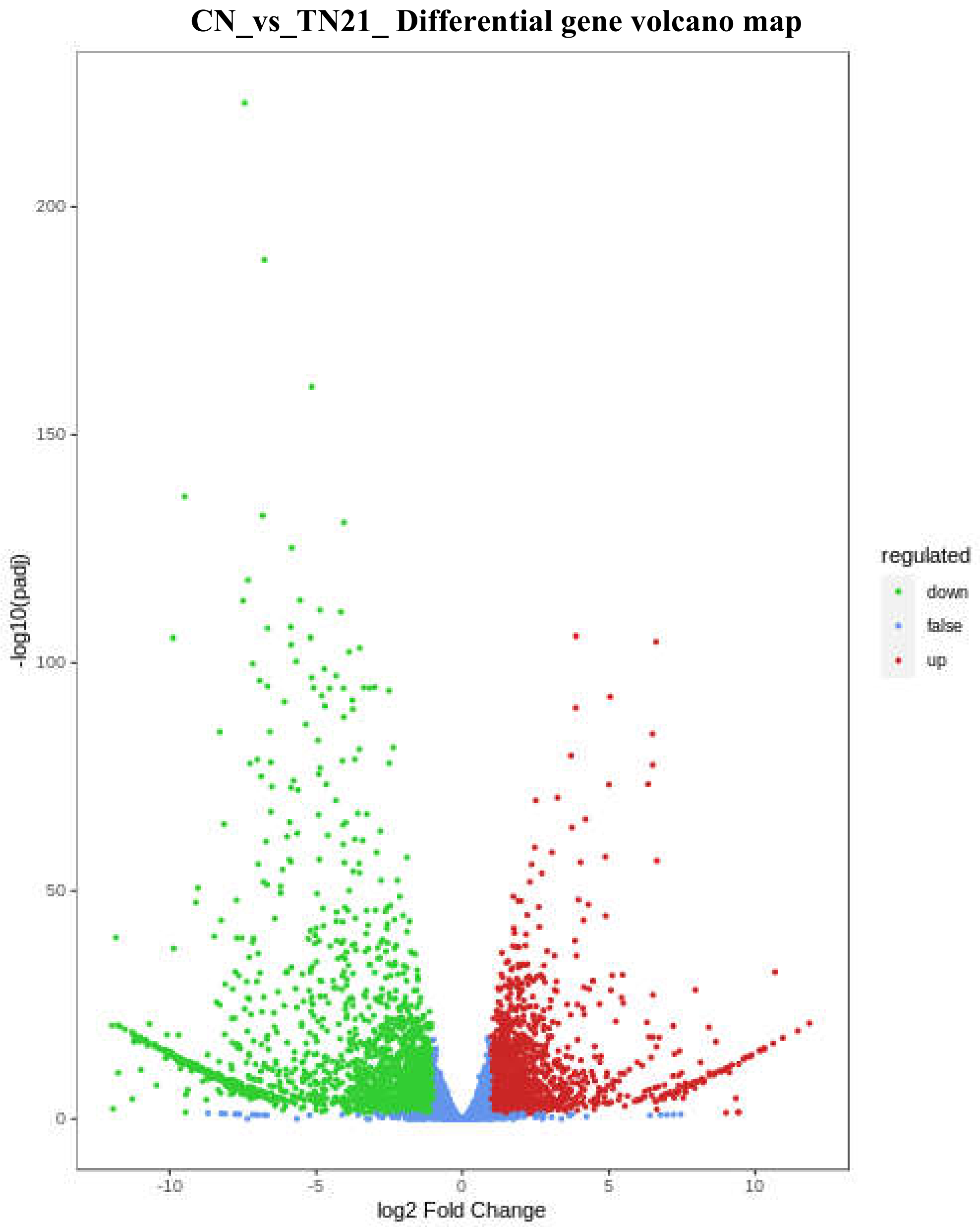

Figure S3: The volcano graph of DEG between different pineapple species

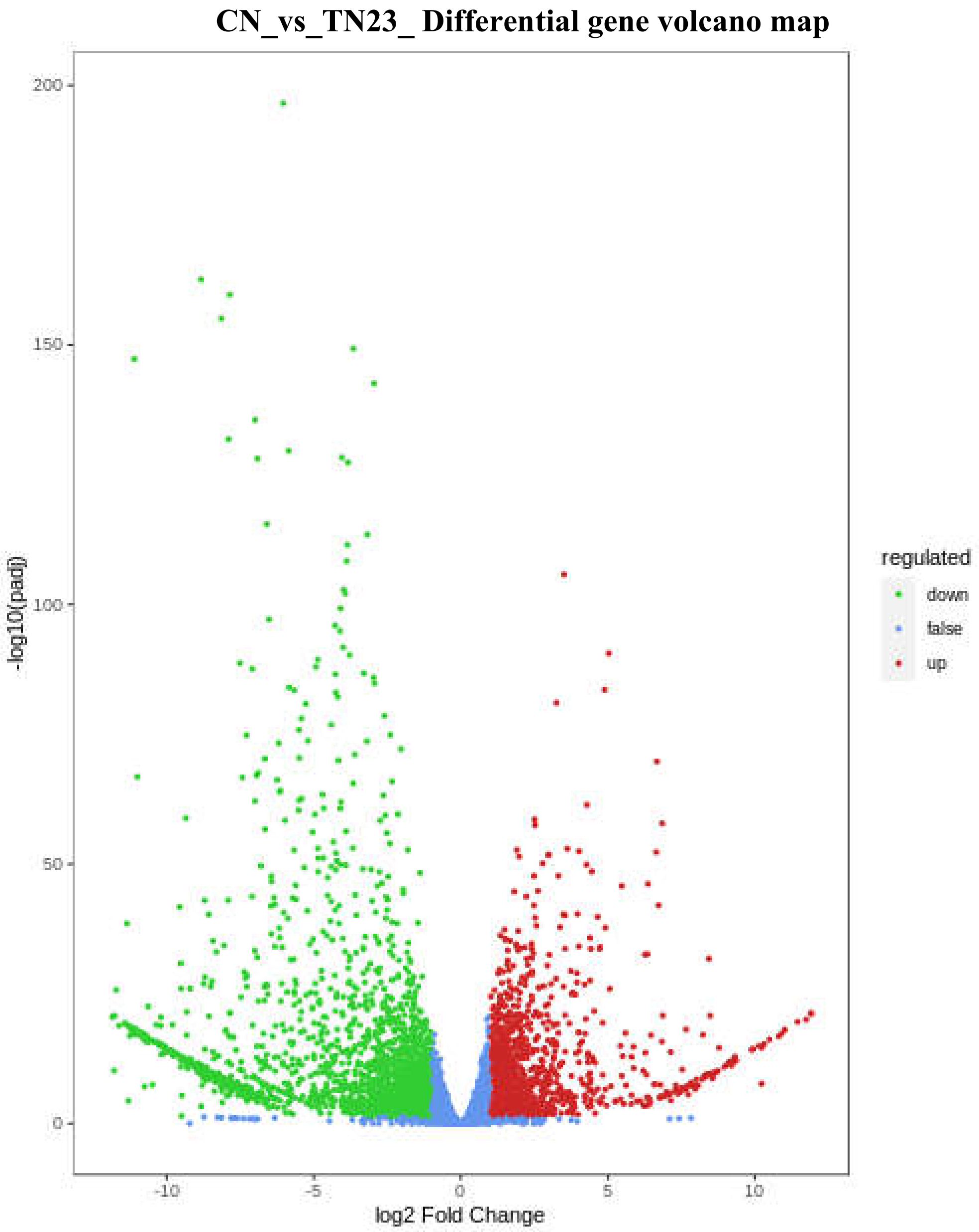

Figure S3: The volcano graph of DEG between different pineapple species

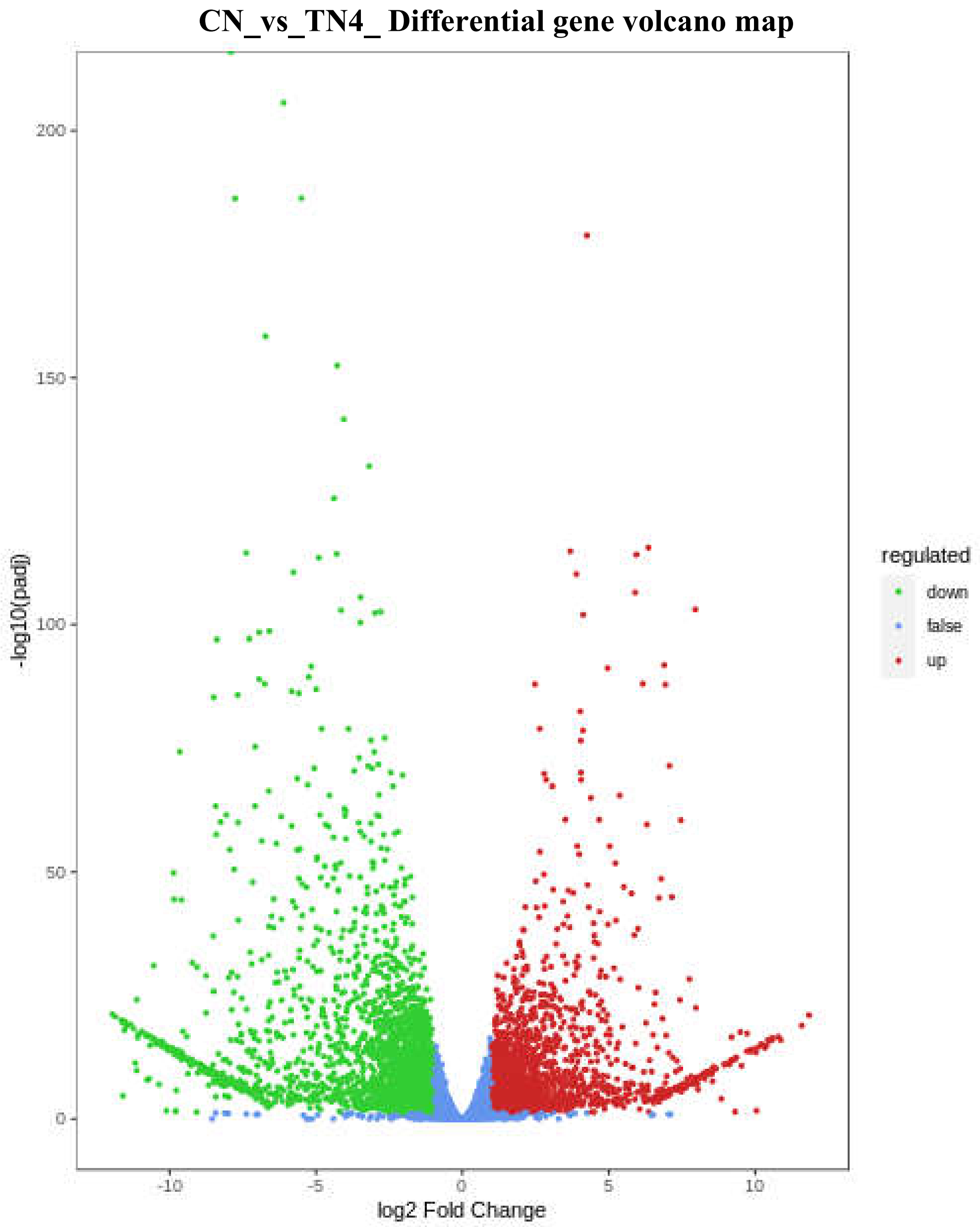

Figure S3: The volcano graph of DEG between different pineapple species

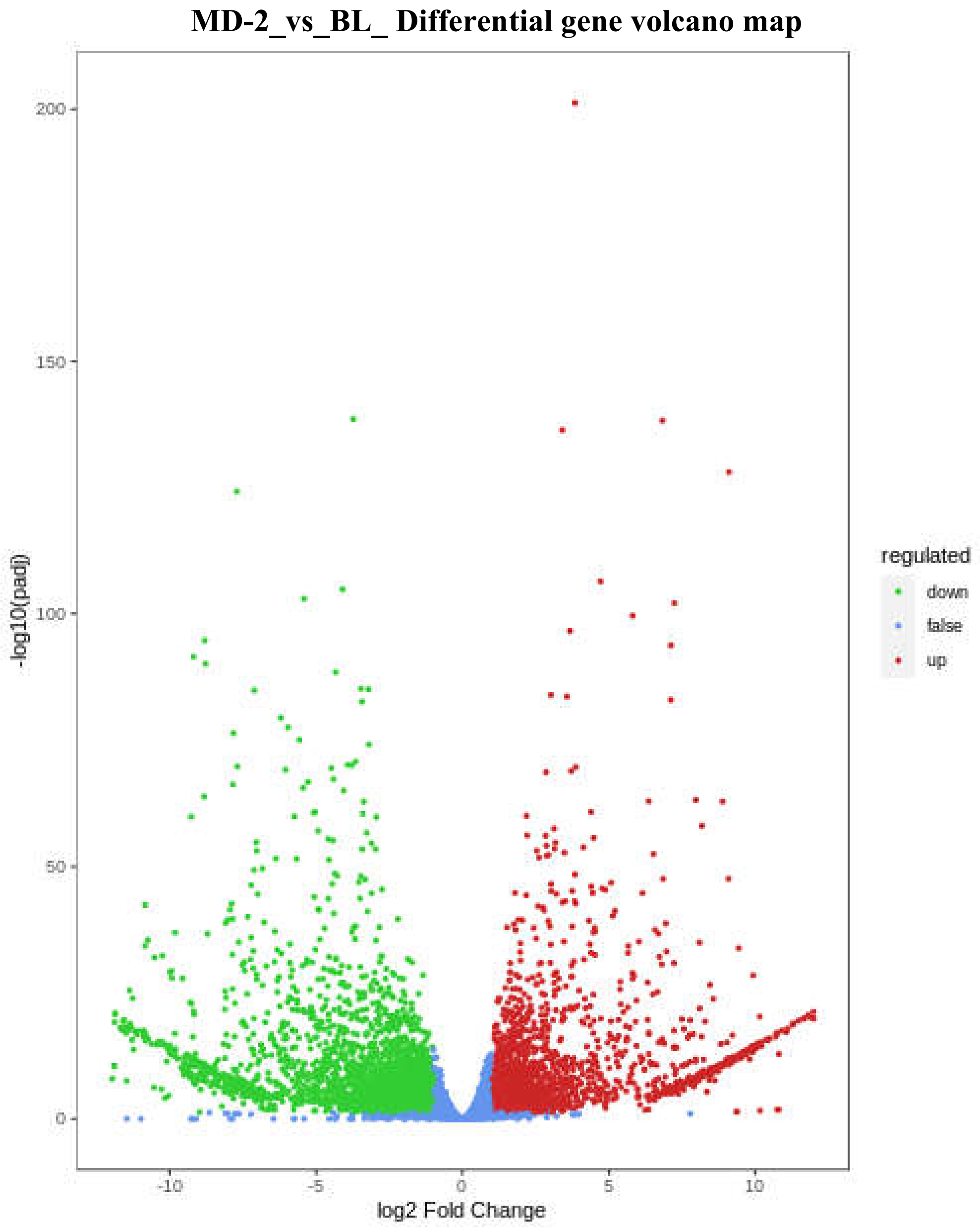

Figure S3: The volcano graph of DEG between different pineapple species

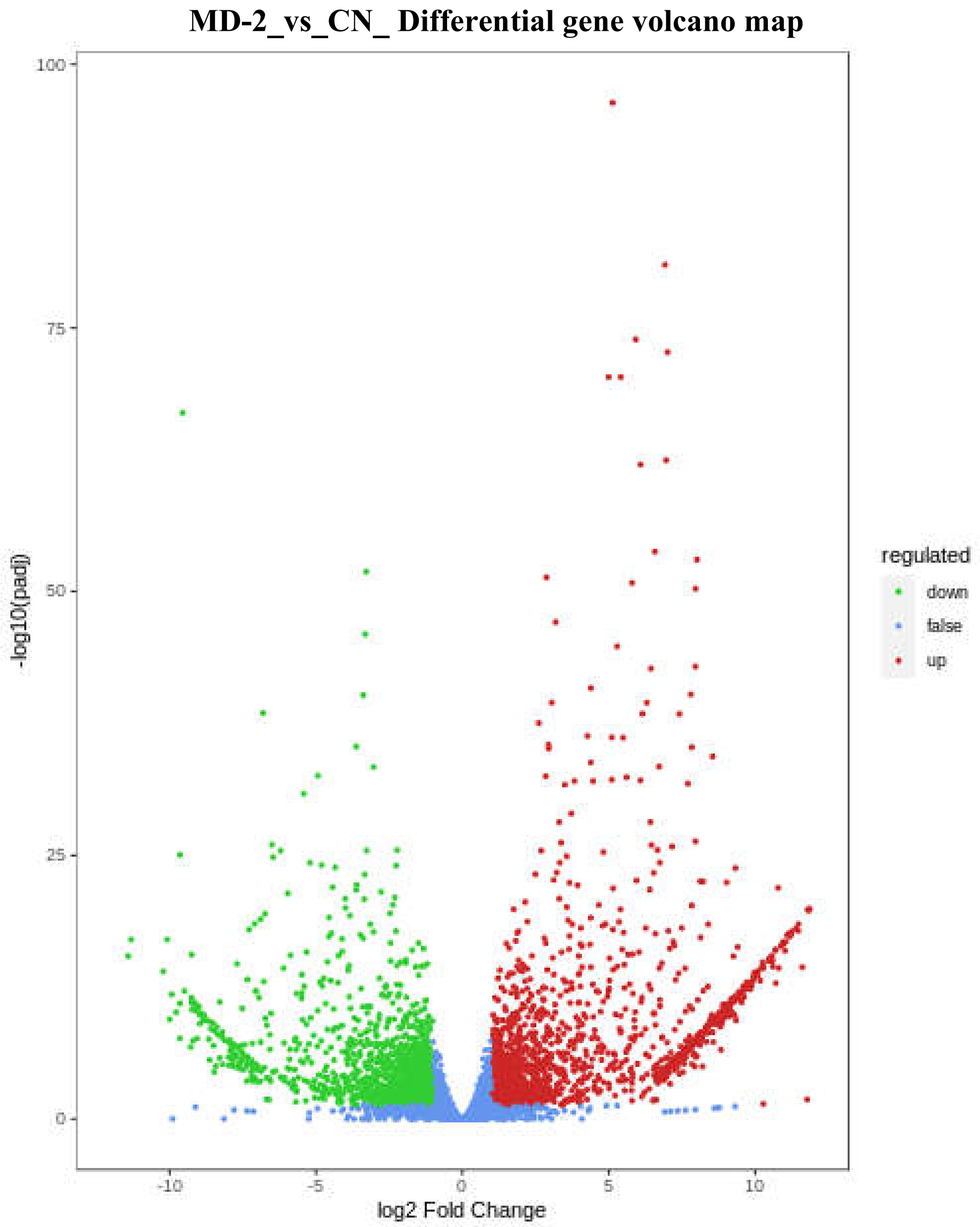

Figure S3: The volcano graph of DEG between different pineapple species

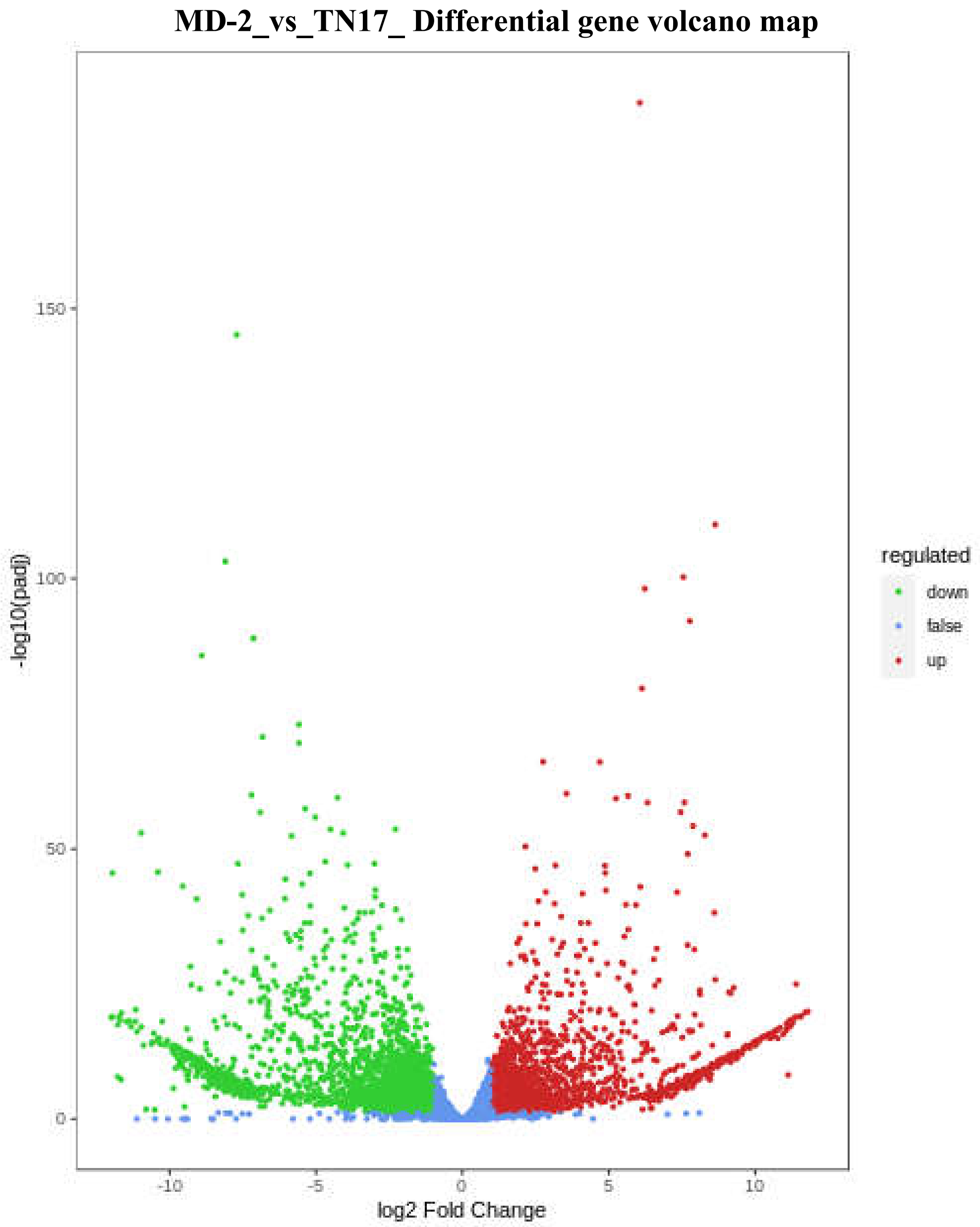

Figure S3: The volcano graph of DEG between different pineapple species

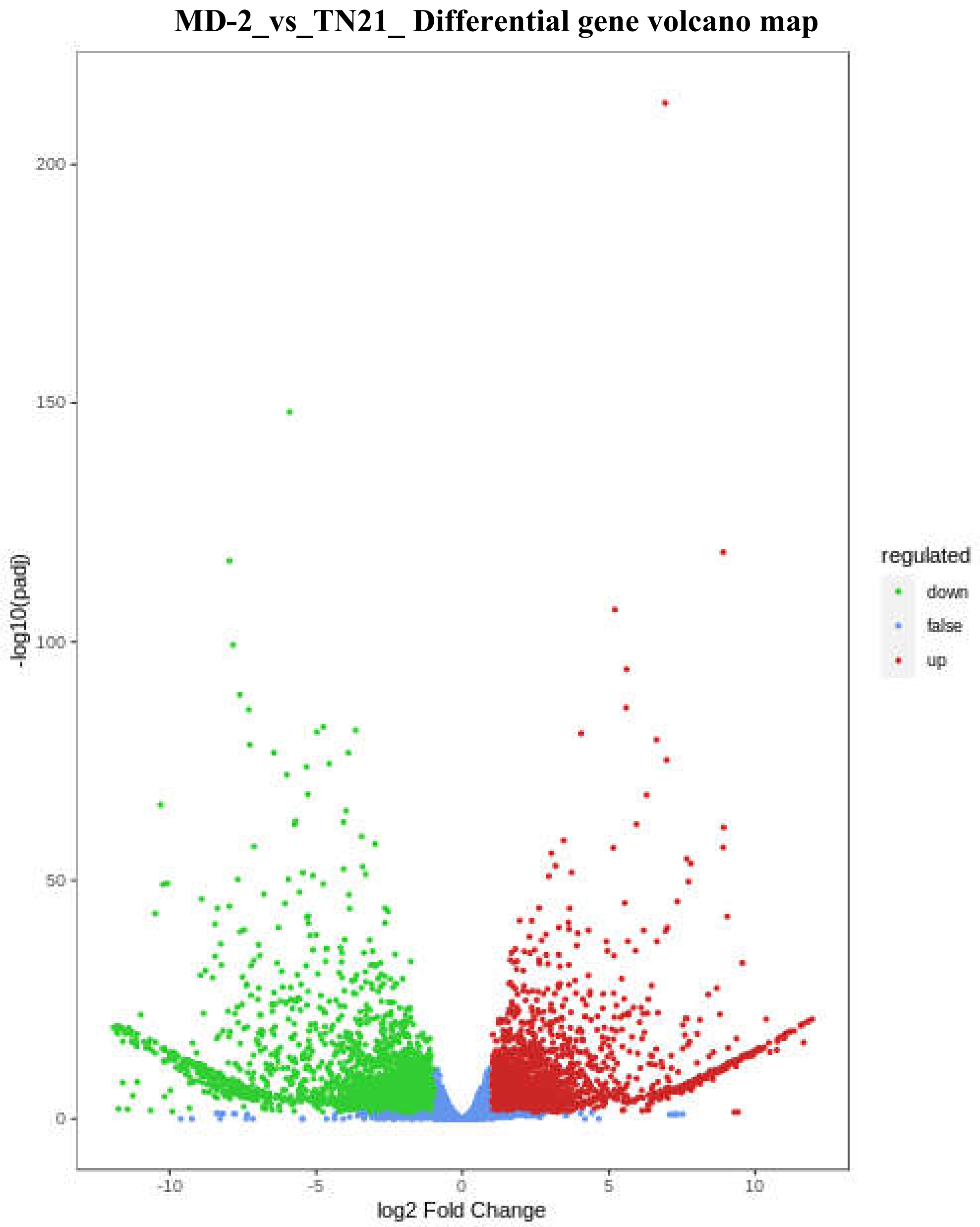

Figure S3: The volcano graph of DEG between different pineapple species

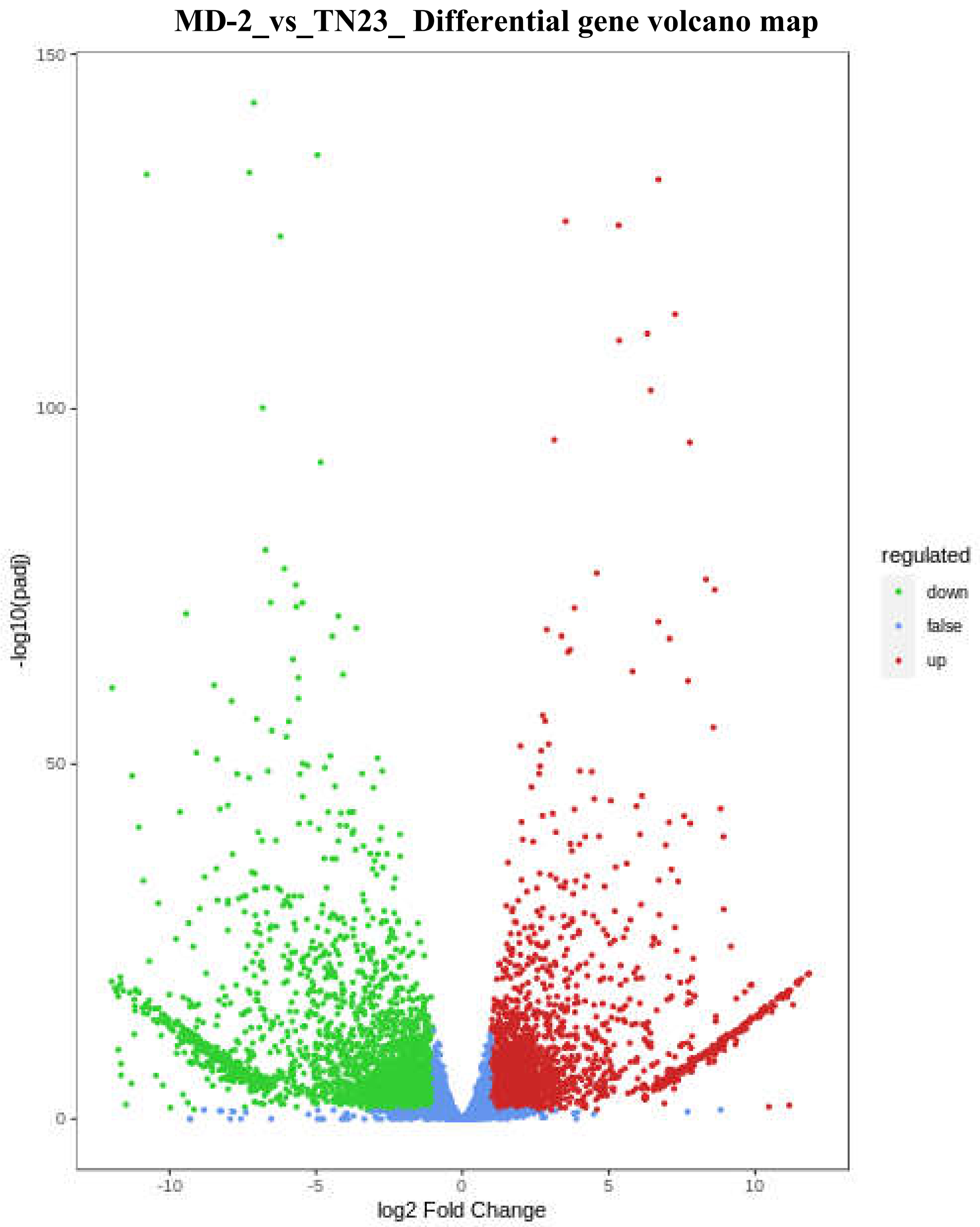

Figure S3: The volcano graph of DEG between different pineapple species

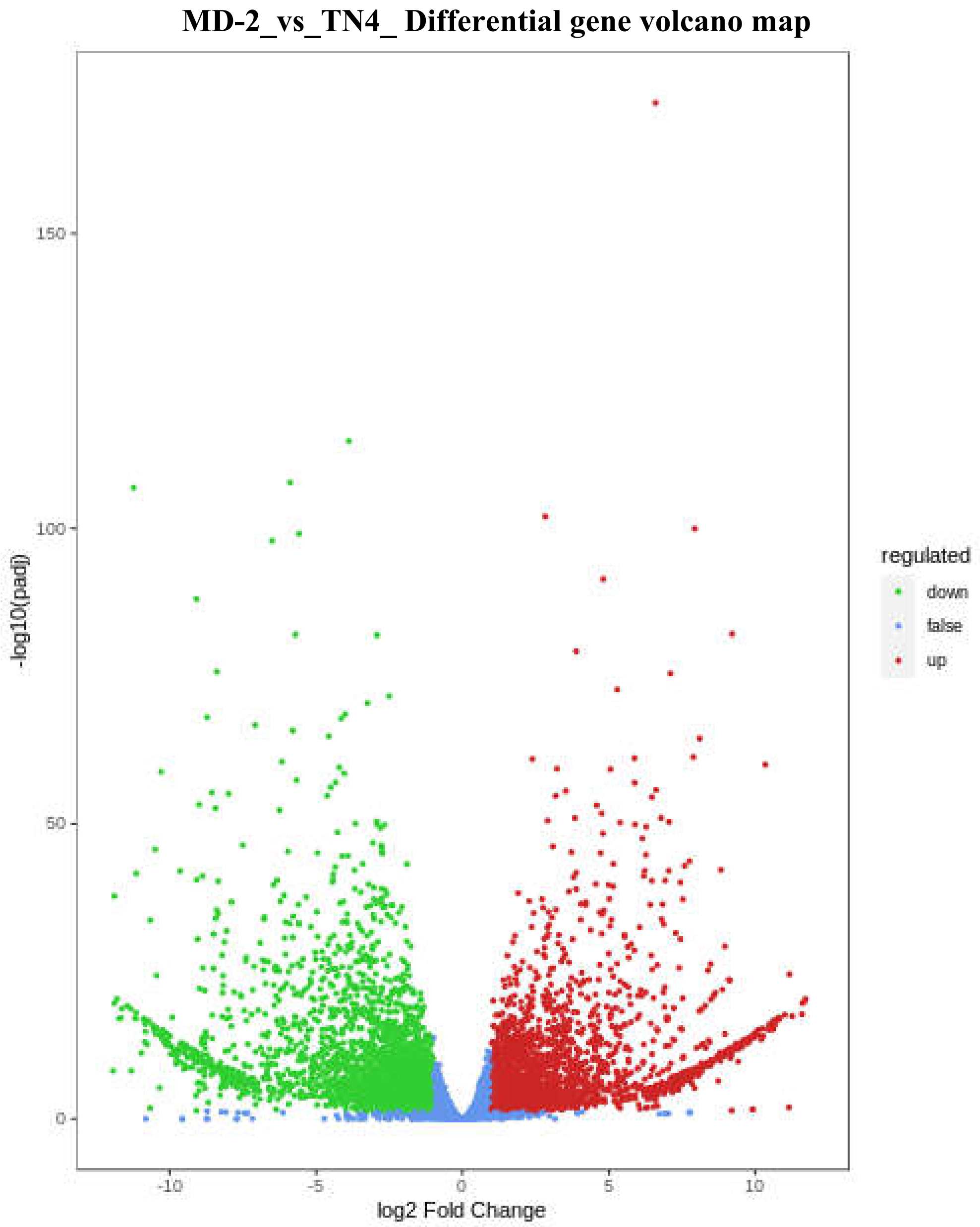

Figure S3: The volcano graph of DEG between different pineapple species

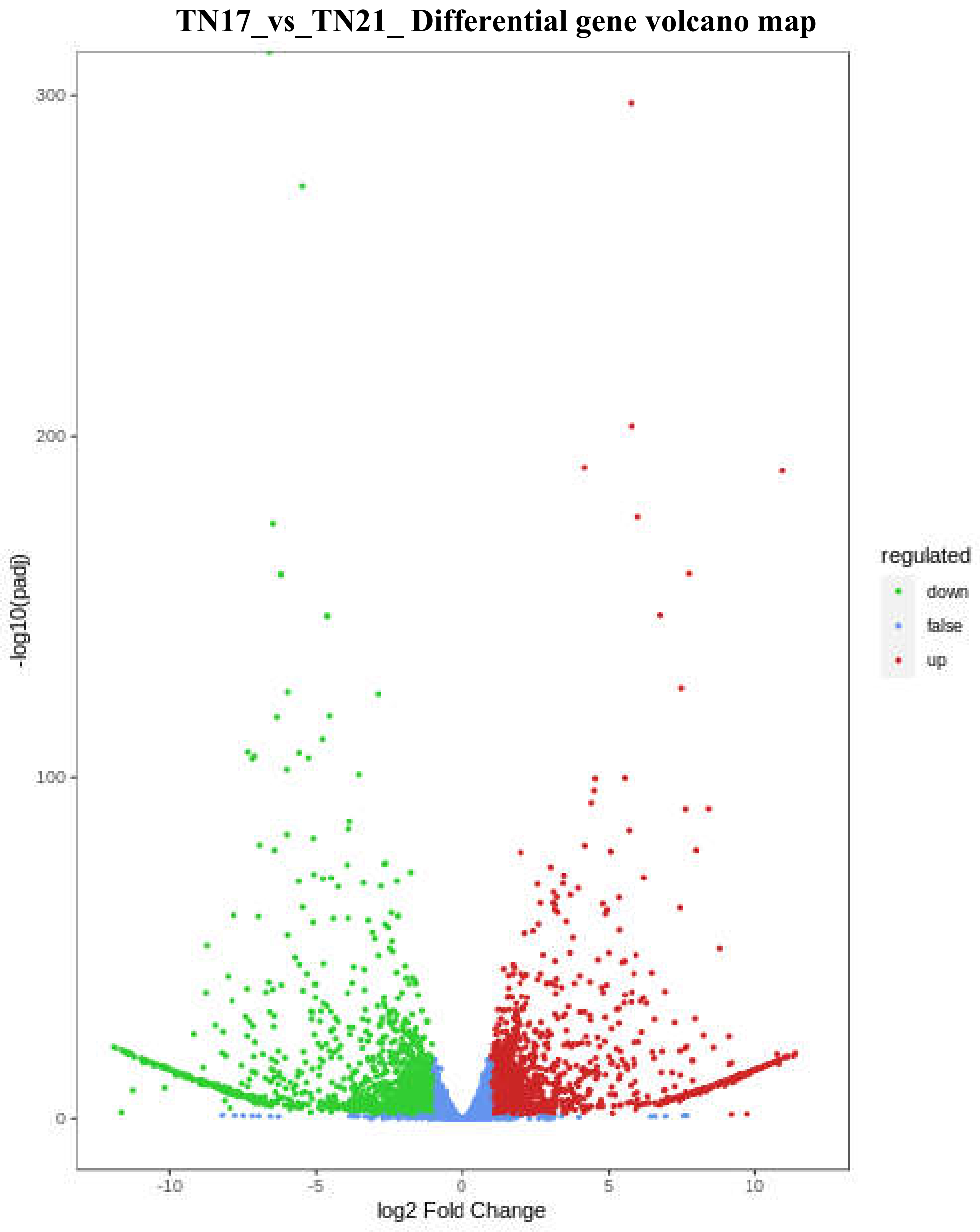

Figure S3: The volcano graph of DEG between different pineapple species

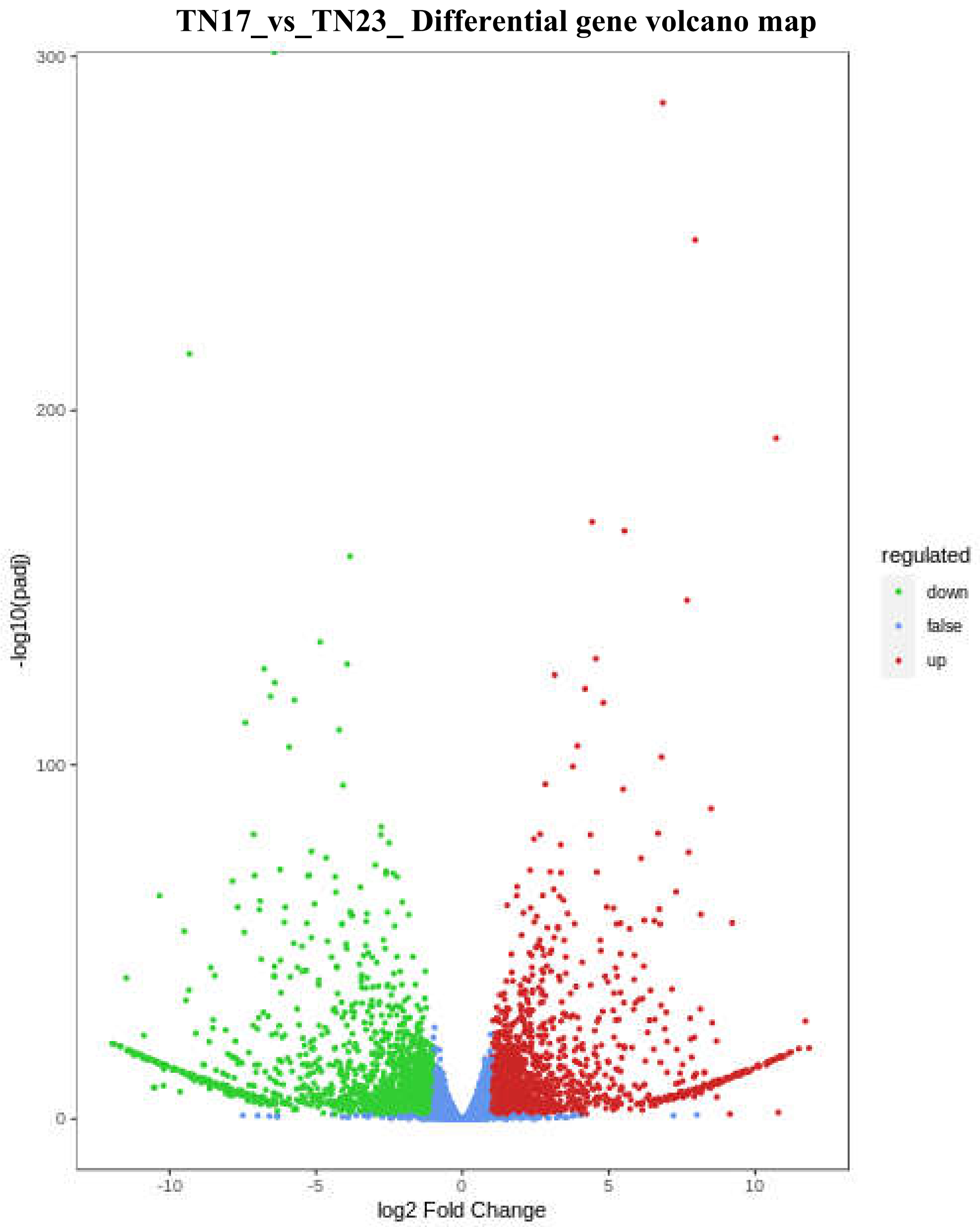

Figure S3: The volcano graph of DEG between different pineapple species

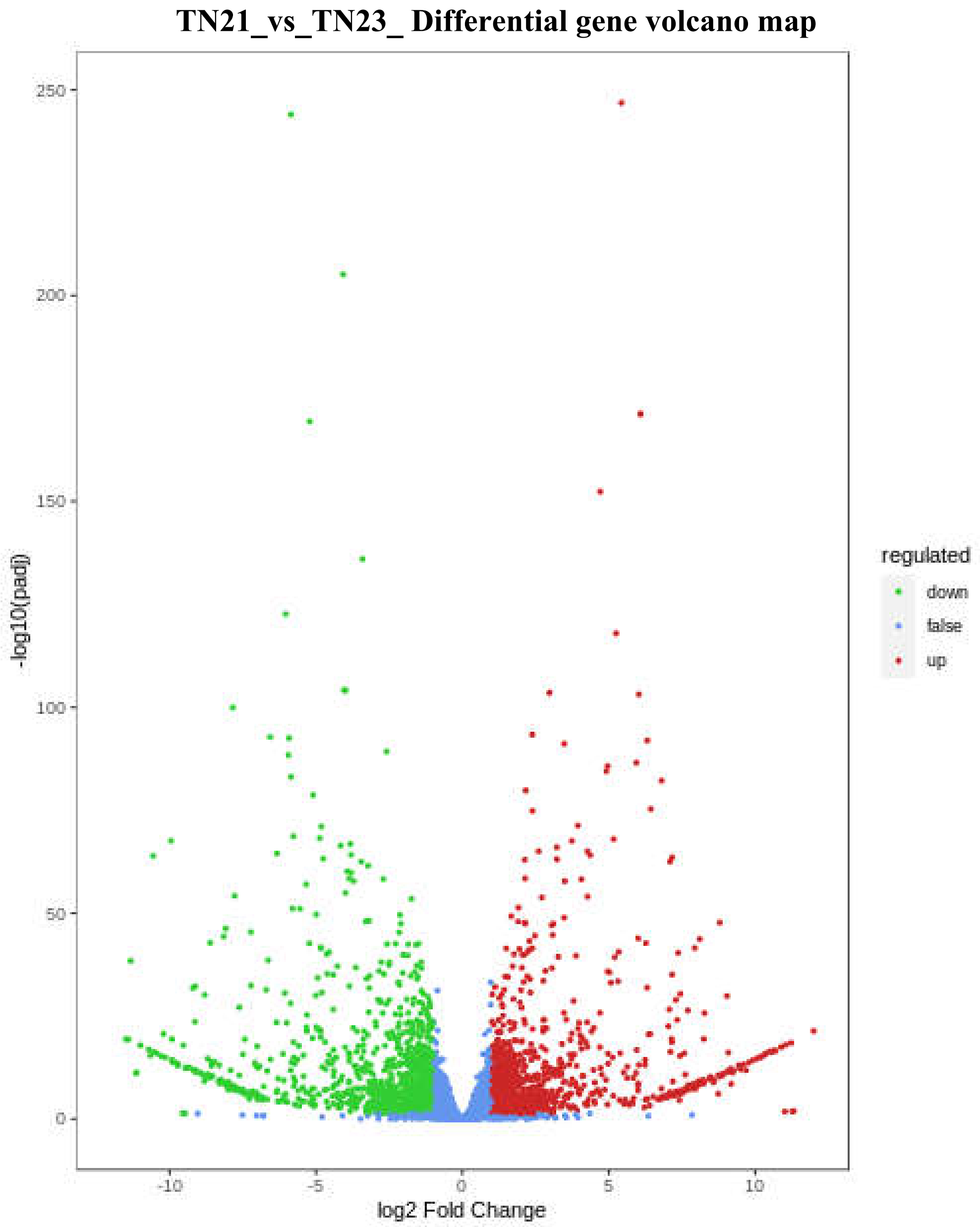

Figure S3: The volcano graph of DEG between different pineapple species

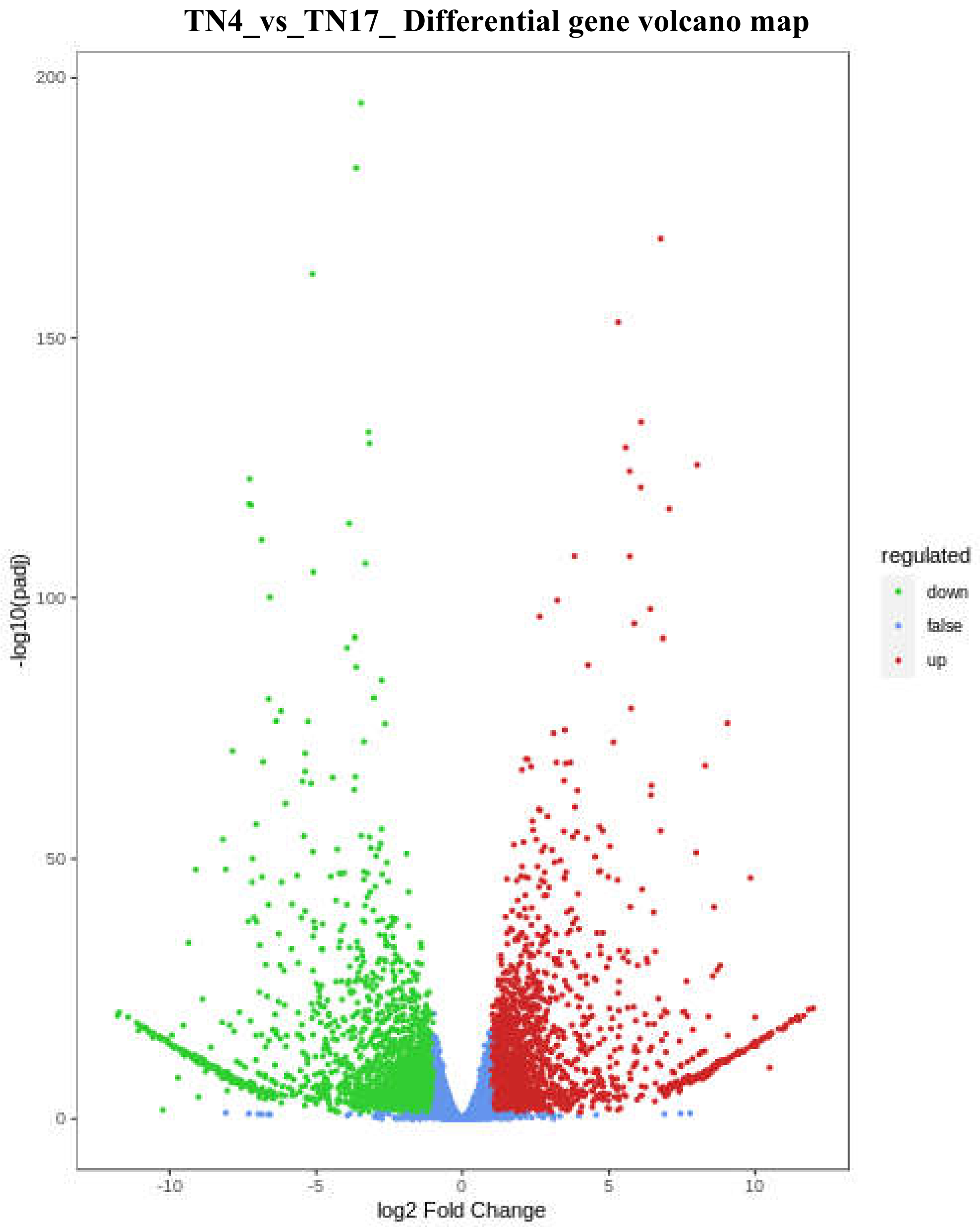

Figure S3: The volcano graph of DEG between different pineapple species

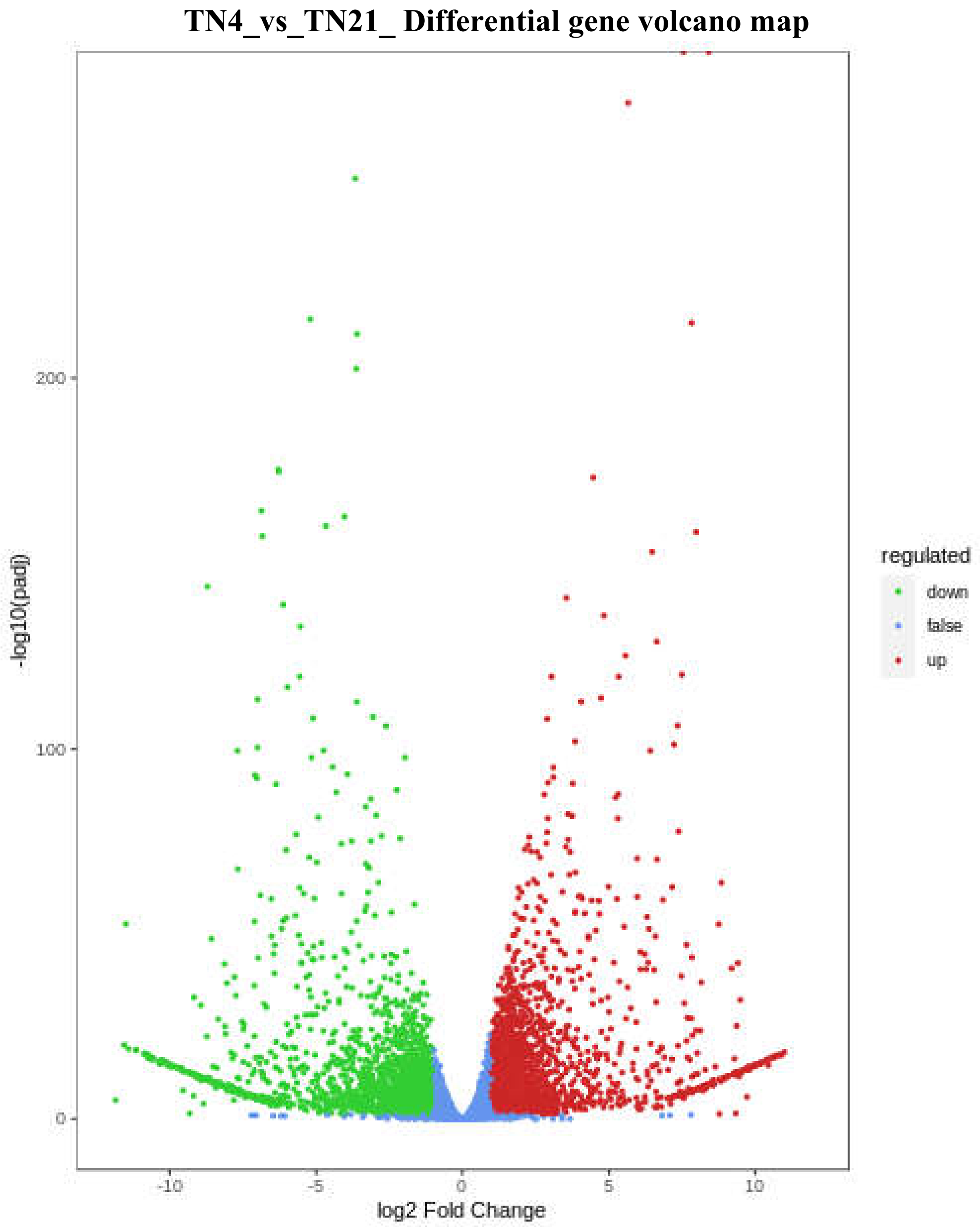

Figure S3: The volcano graph of DEG between different pineapple species

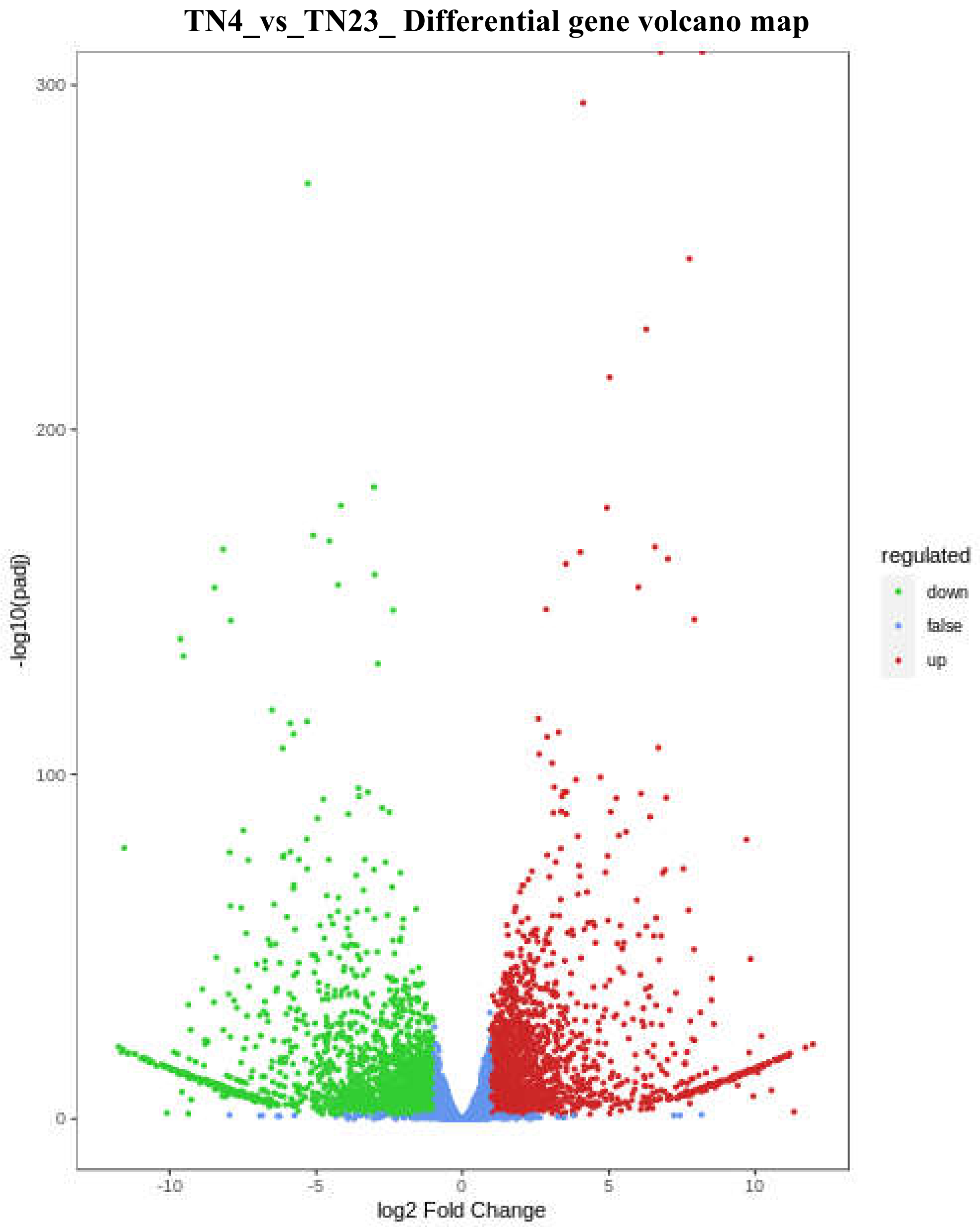

Supplement: Supplementary file 1 [file ijms-24-07199-s001.zip › ijms-2168485-supplementary/Supplementary Files/Figure S3.pdf]
