# Supplementary material for: Integrated Metabolome and Transcriptome Analysis Reveals a Potential Mechanism for Water Accumulation Mediated Translucency in Pineapple (Ananas comosus (L.) Merr.) Fruit
Source: Int J Mol Sci. 2023 Apr 13;24(8):7199. doi: 10.3390/ijms24087199 (PMC10139408; doi:10.3390/ijms24087199)

Figure S5: GO enrichment analysis of differentially expressed genes

BL_vs_CN

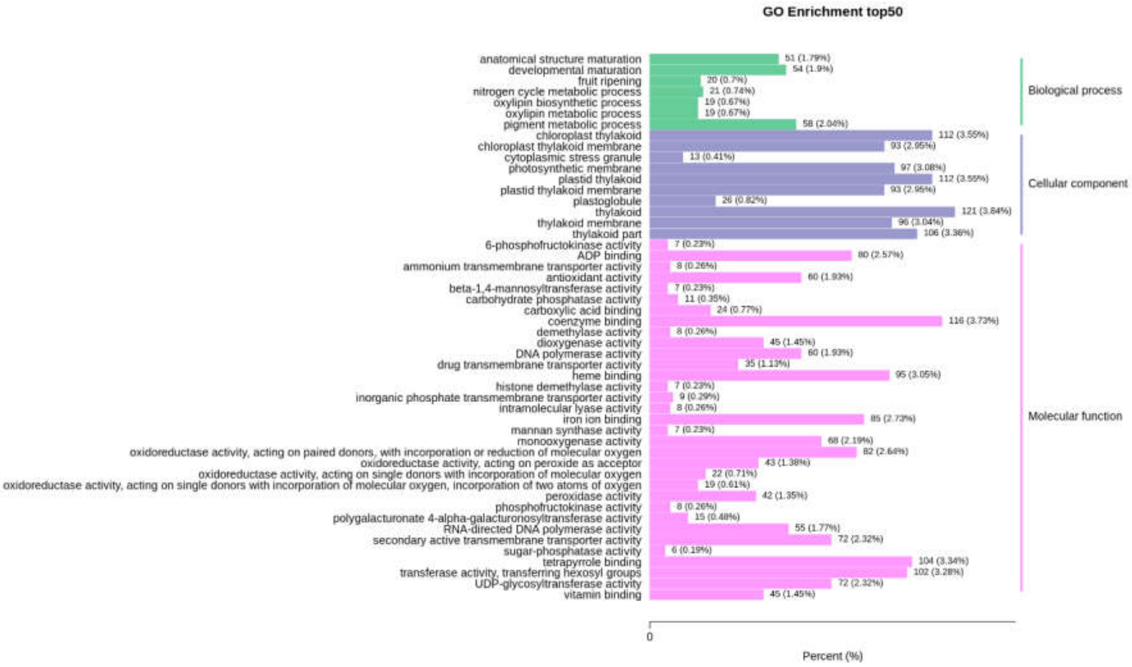

Figure S5: GO enrichment analysis of differentially expressed genes

BL_vs_TN17

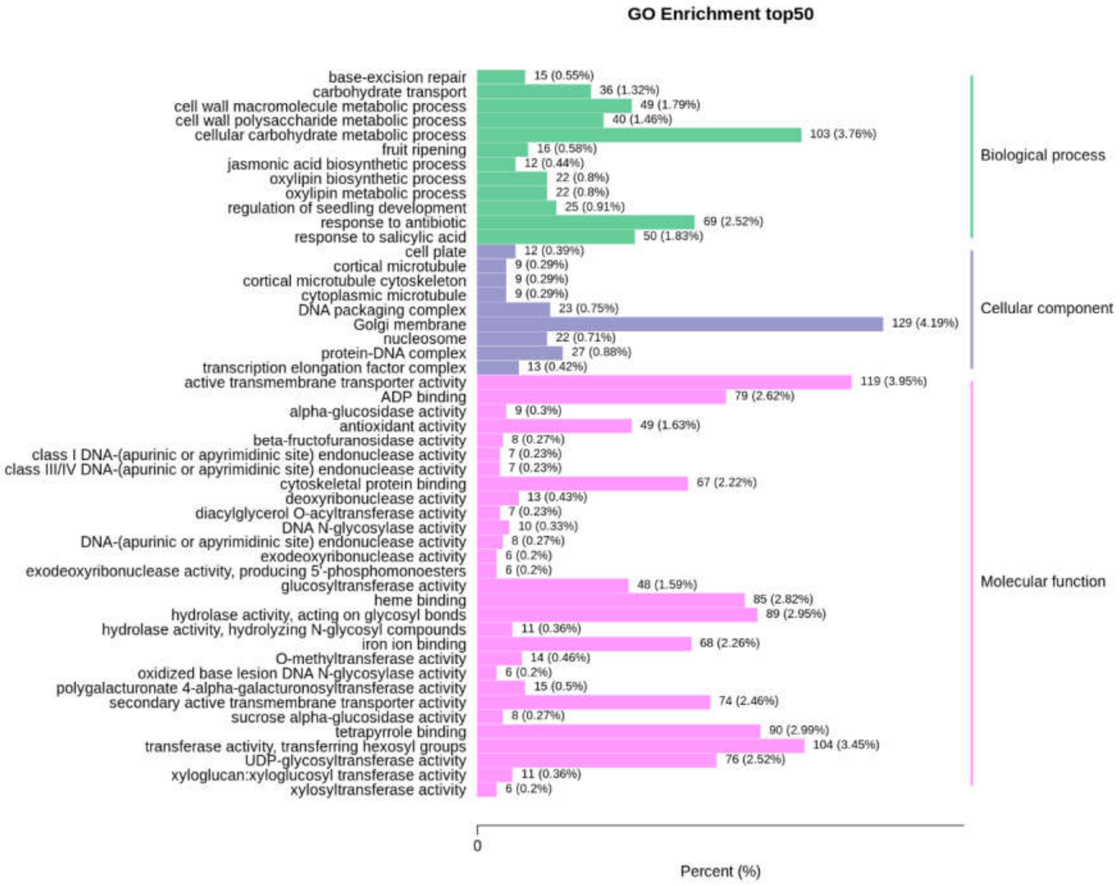

Figure S5: GO enrichment analysis of differentially expressed genes

BL_vs_TN21

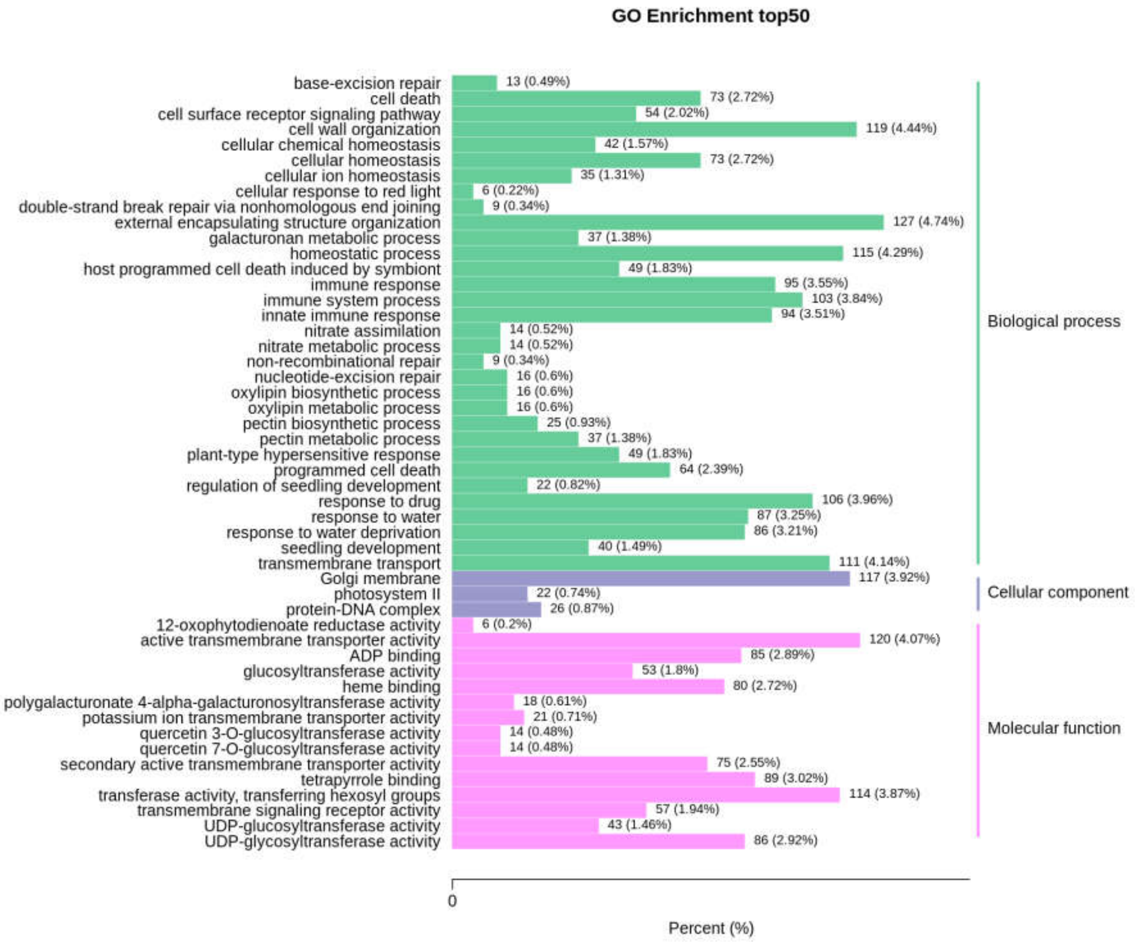

Figure S5: GO enrichment analysis of differentially expressed genes

BL_vs_TN23

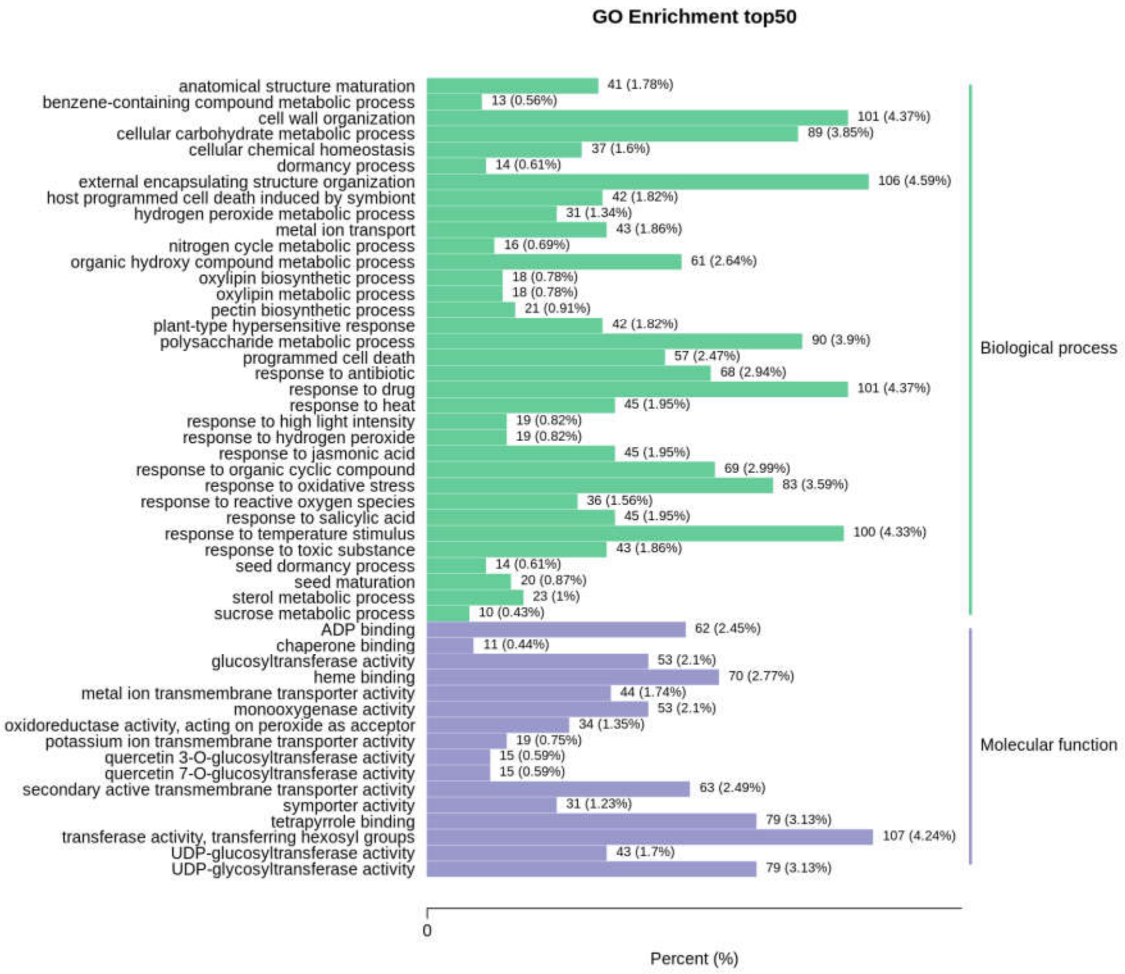

Figure S5: GO enrichment analysis of differentially expressed genes

BL_vs_TN4

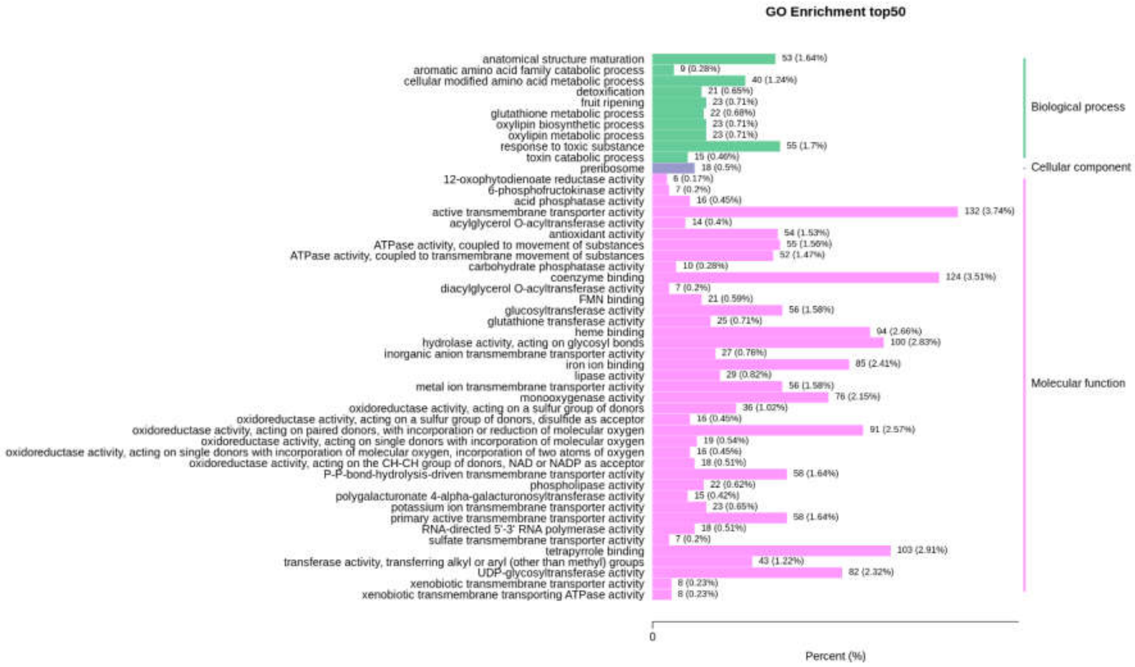

CN_vs_TN17

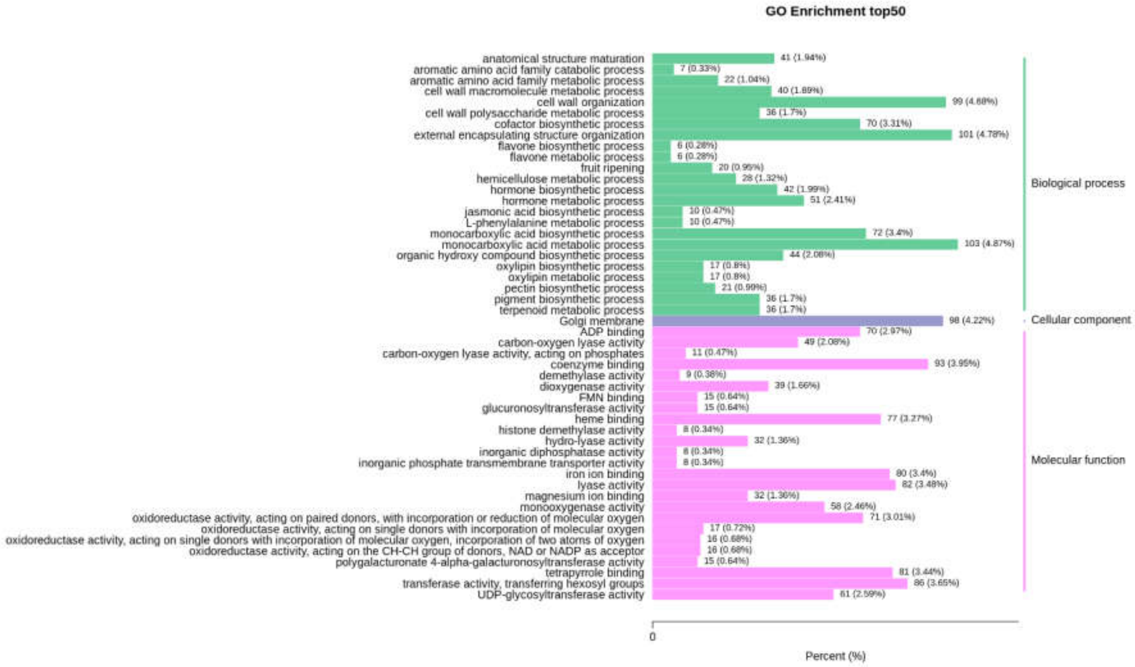

Figure S5: GO enrichment analysis of differentially expressed genes

CN_vs_TN21

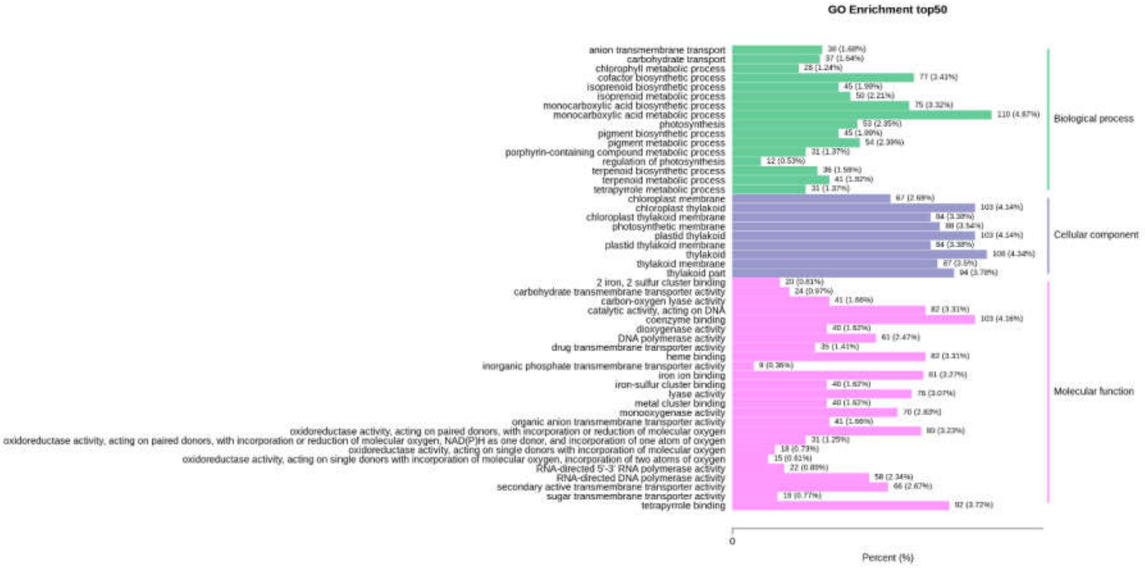

CN_vs_TN23

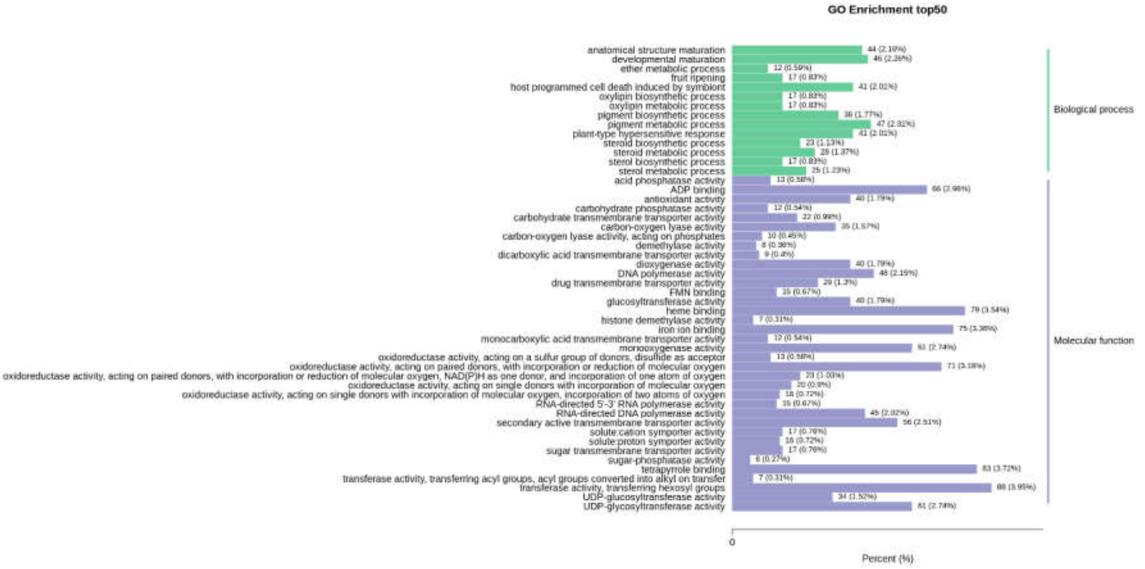

Figure S5: GO enrichment analysis of differentially expressed genes

CN_vs_TN4

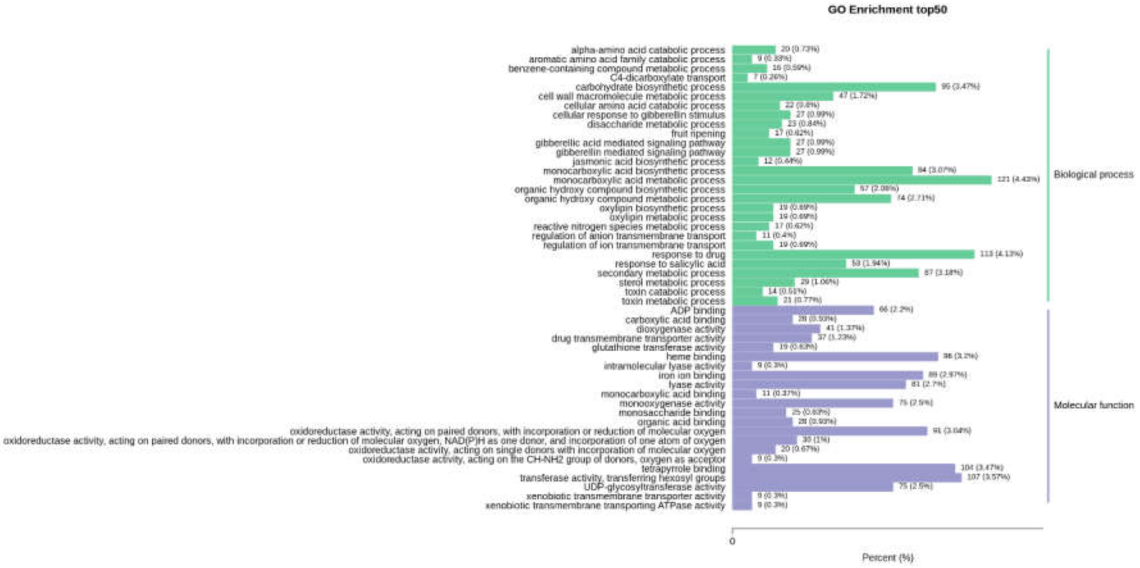

MD-2_vs_BL

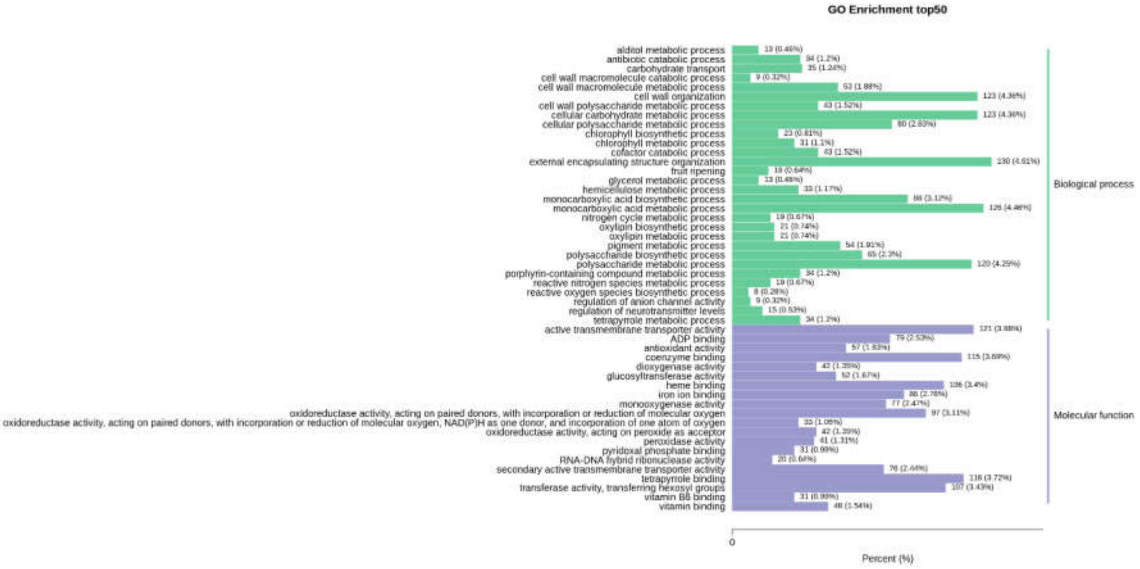

Figure S5: GO enrichment analysis of differentially expressed genes

MD-2_vs_CN

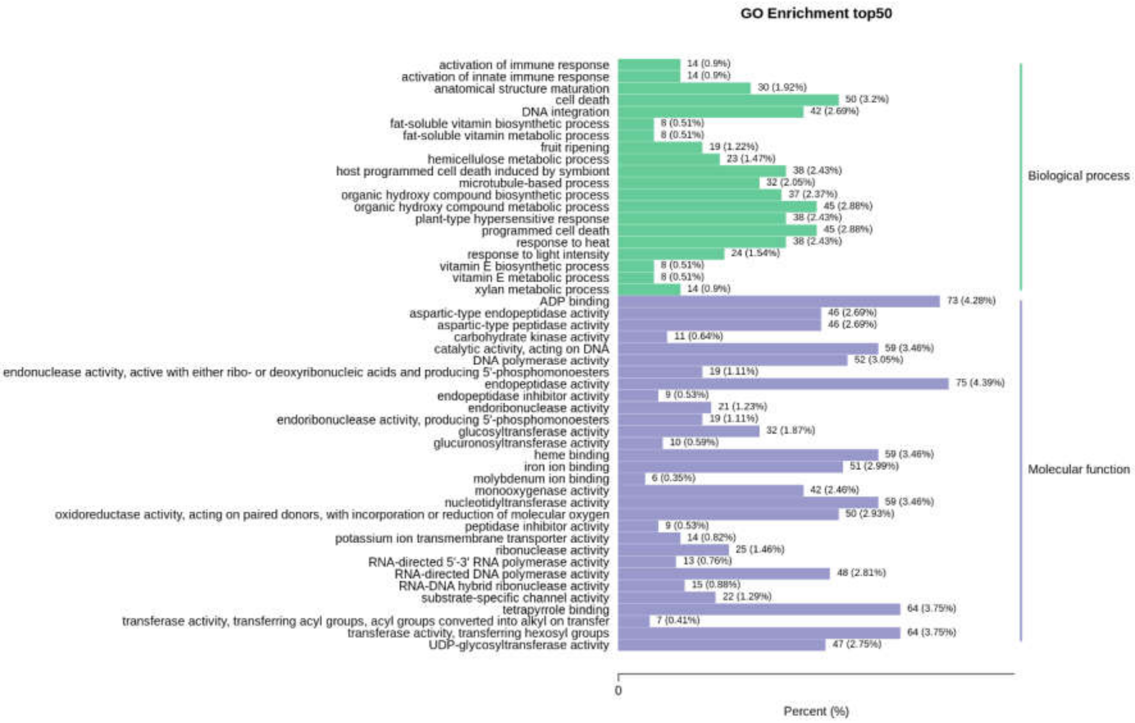

MD-2_vs_TN17

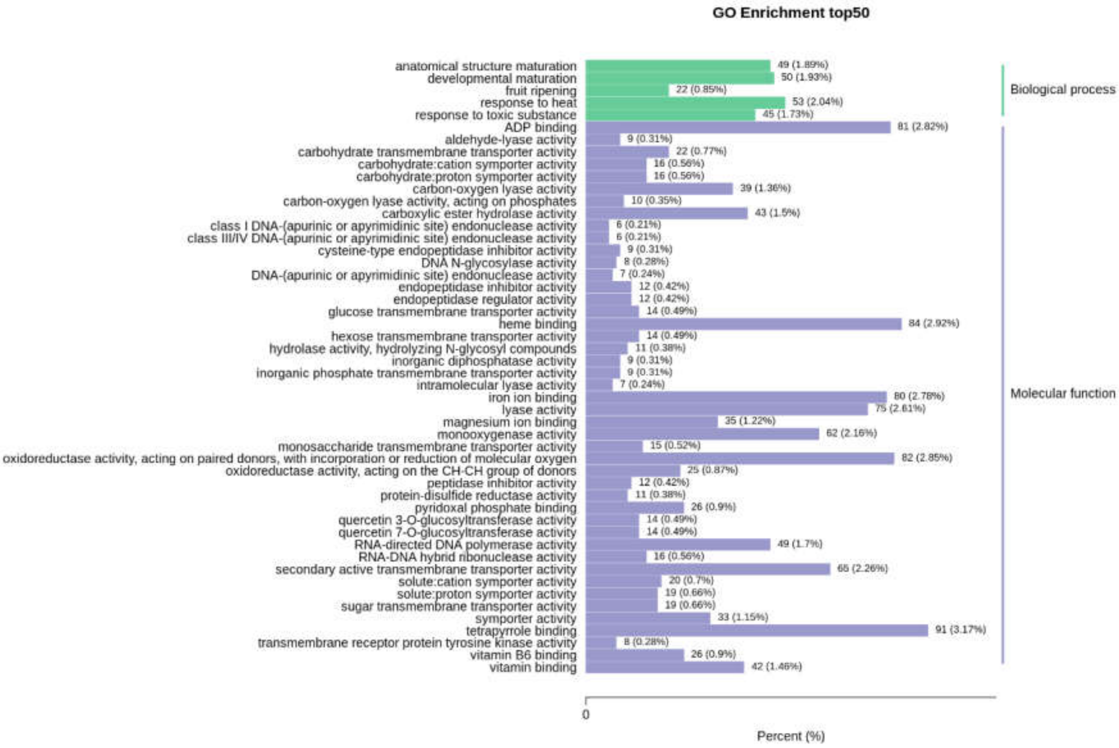

Figure S5: GO enrichment analysis of differentially expressed genes

MD-2_vs_TN21

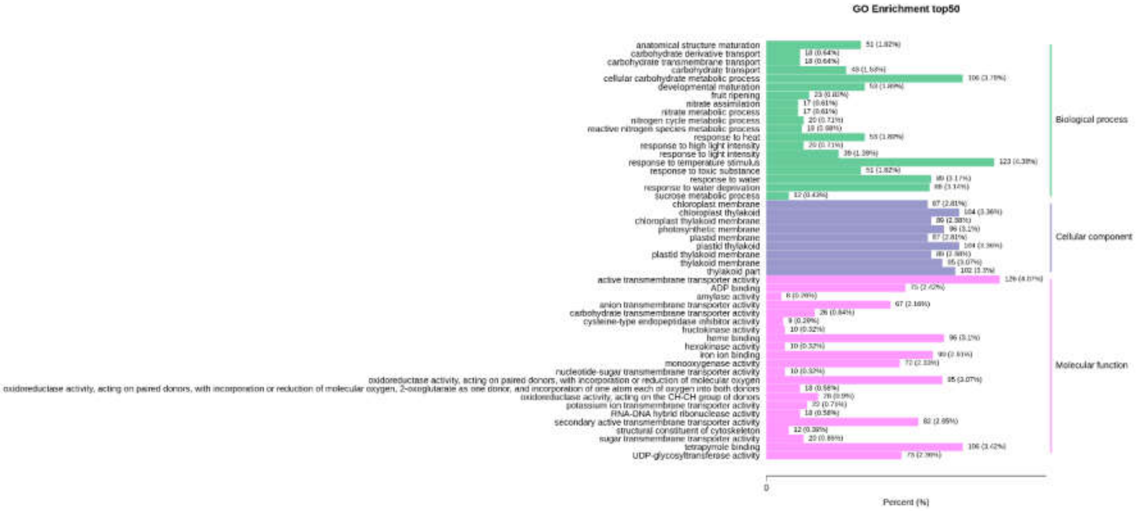

MD-2_vs_TN23

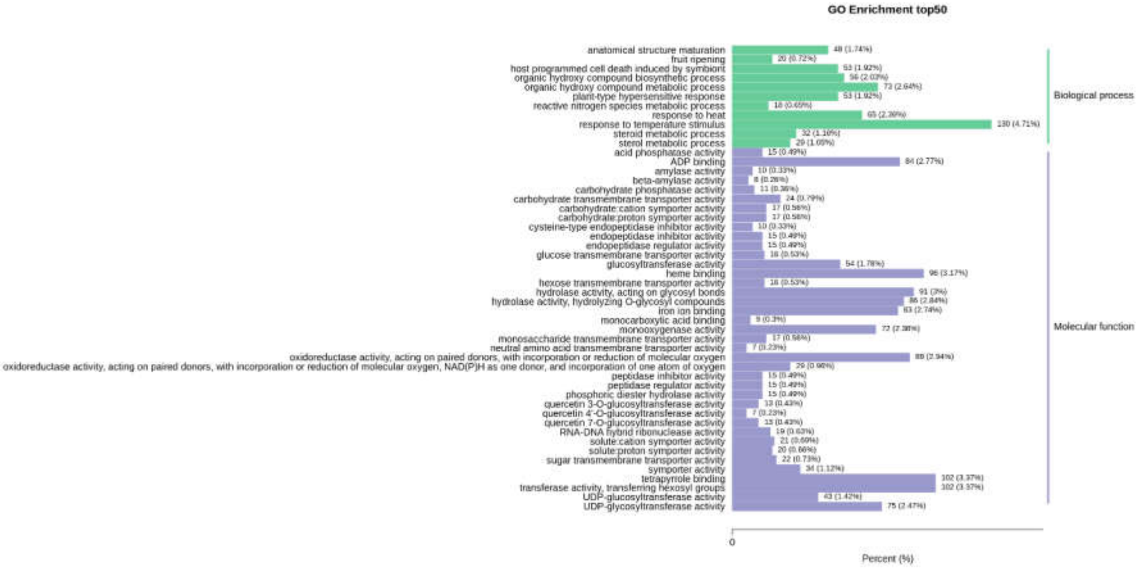

Figure S5: GO enrichment analysis of differentially expressed genes

MD-2_vs_TN4

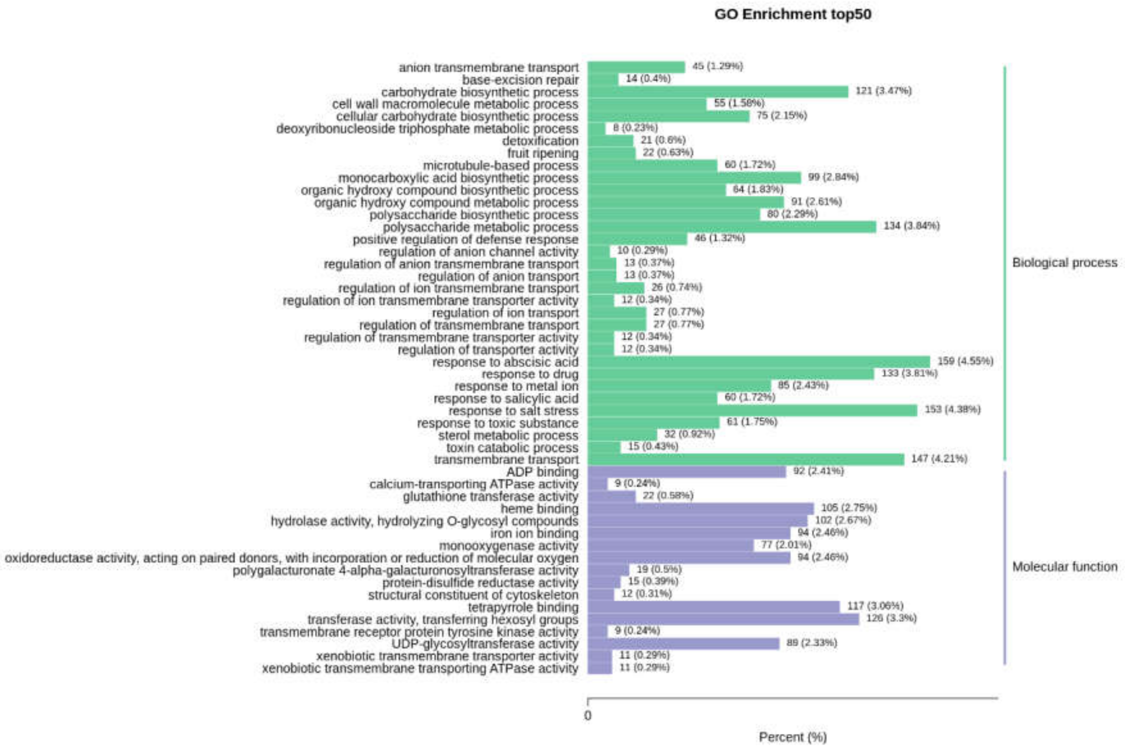

TN17_vs_TN21

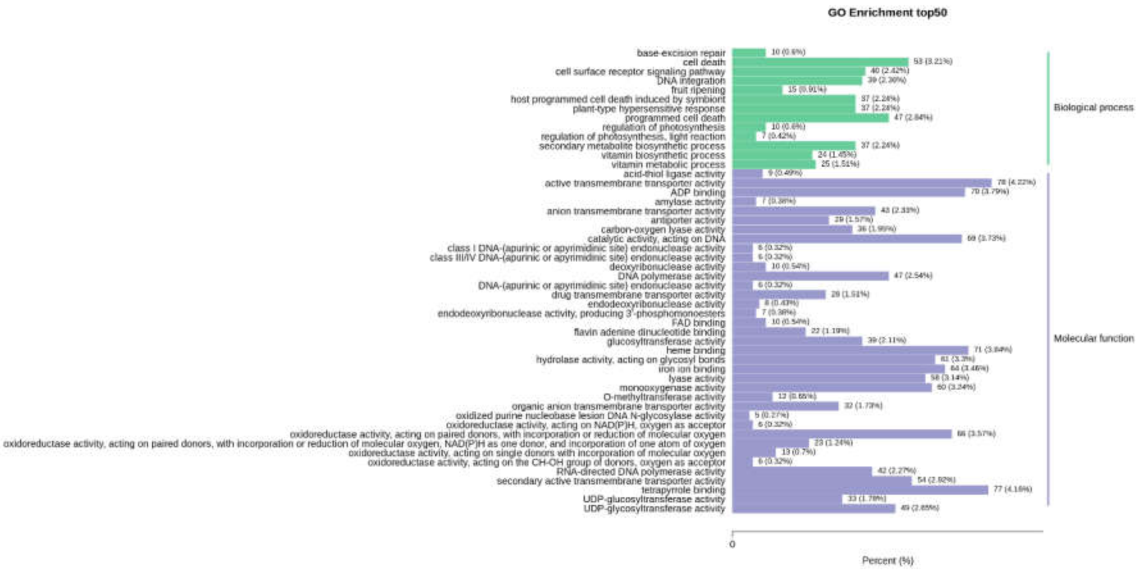

Figure S5: GO enrichment analysis of differentially expressed genes

TN17_vs_TN23

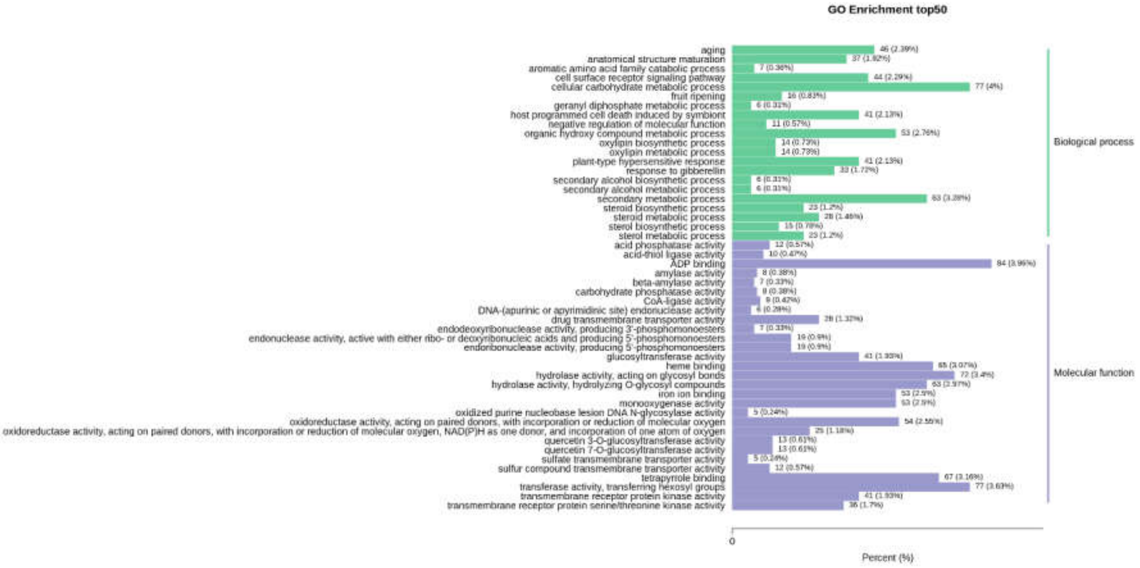

TN21_vs_TN23

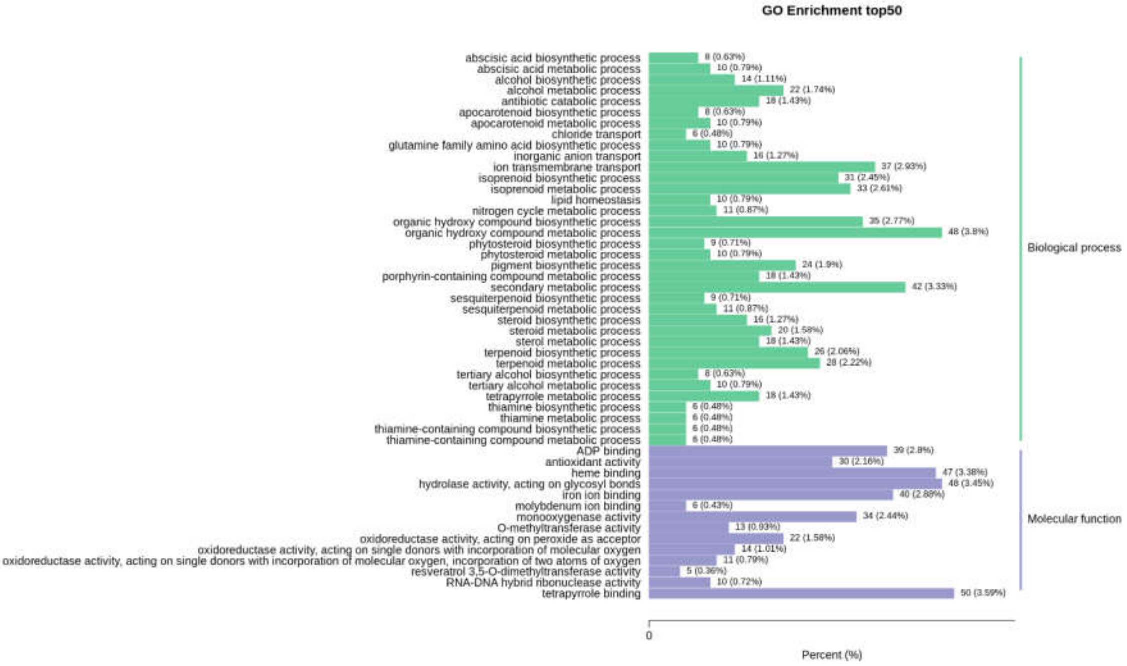

TN4_vs_TN17

Figure S5: GO enrichment analysis of differentially expressed genes

TN4_vs_TN21

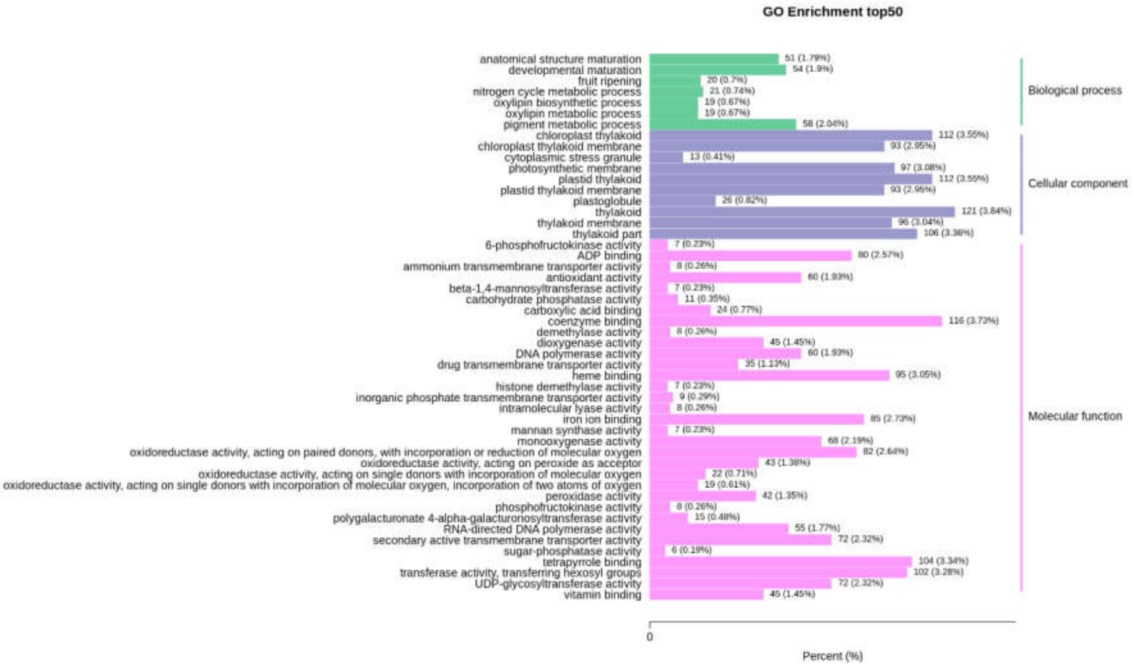

Figure S5: GO enrichment analysis of differentially expressed genes

TN4_vs_TN23

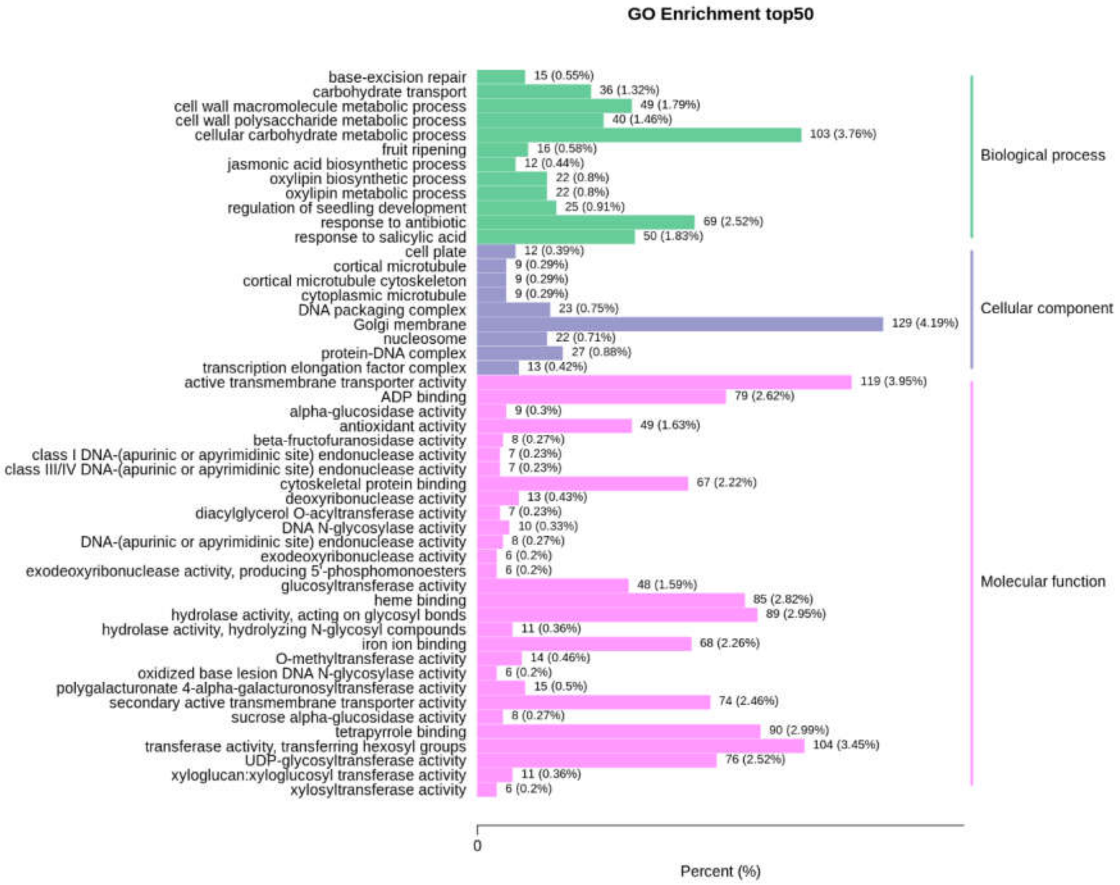

Figure S5: GO enrichment analysis of differentially expressed genes

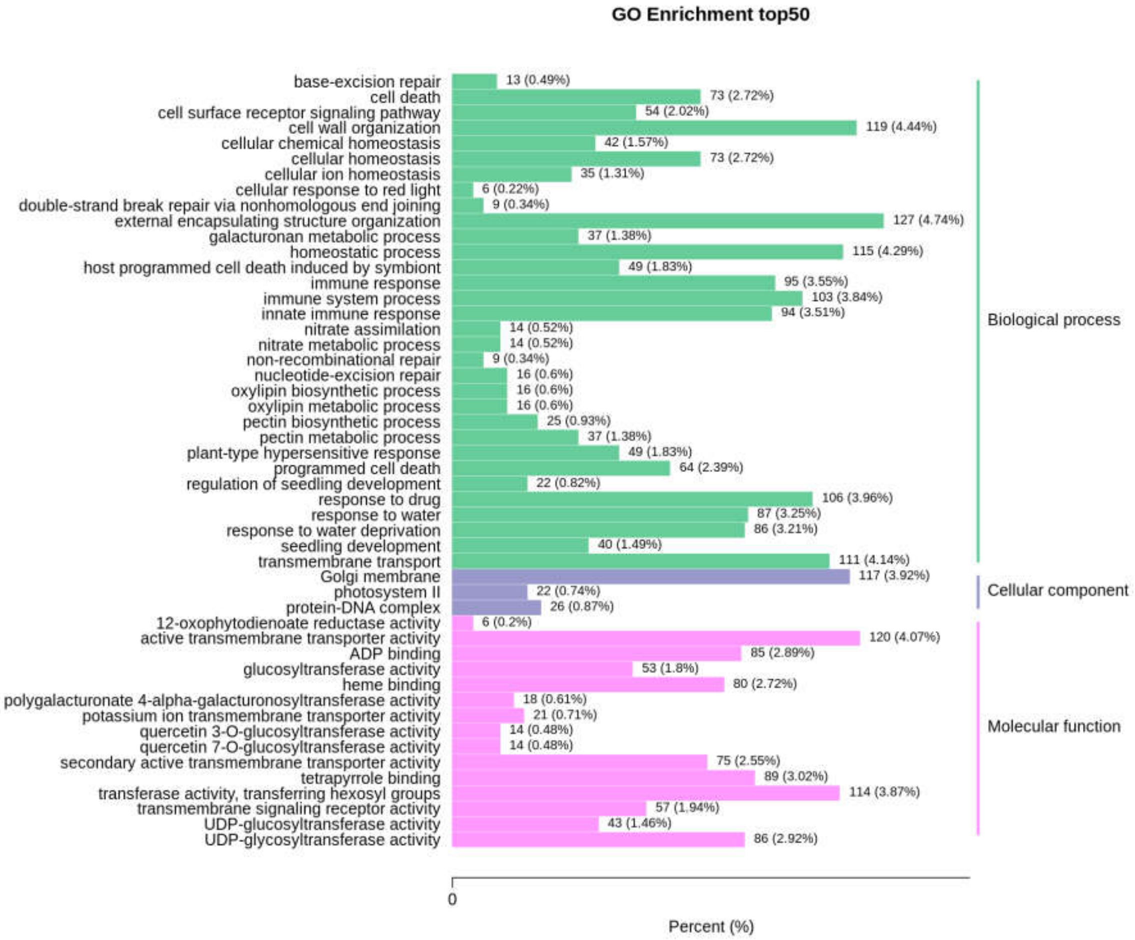

Figure S5: GO enrichment analysis of differentially expressed genes

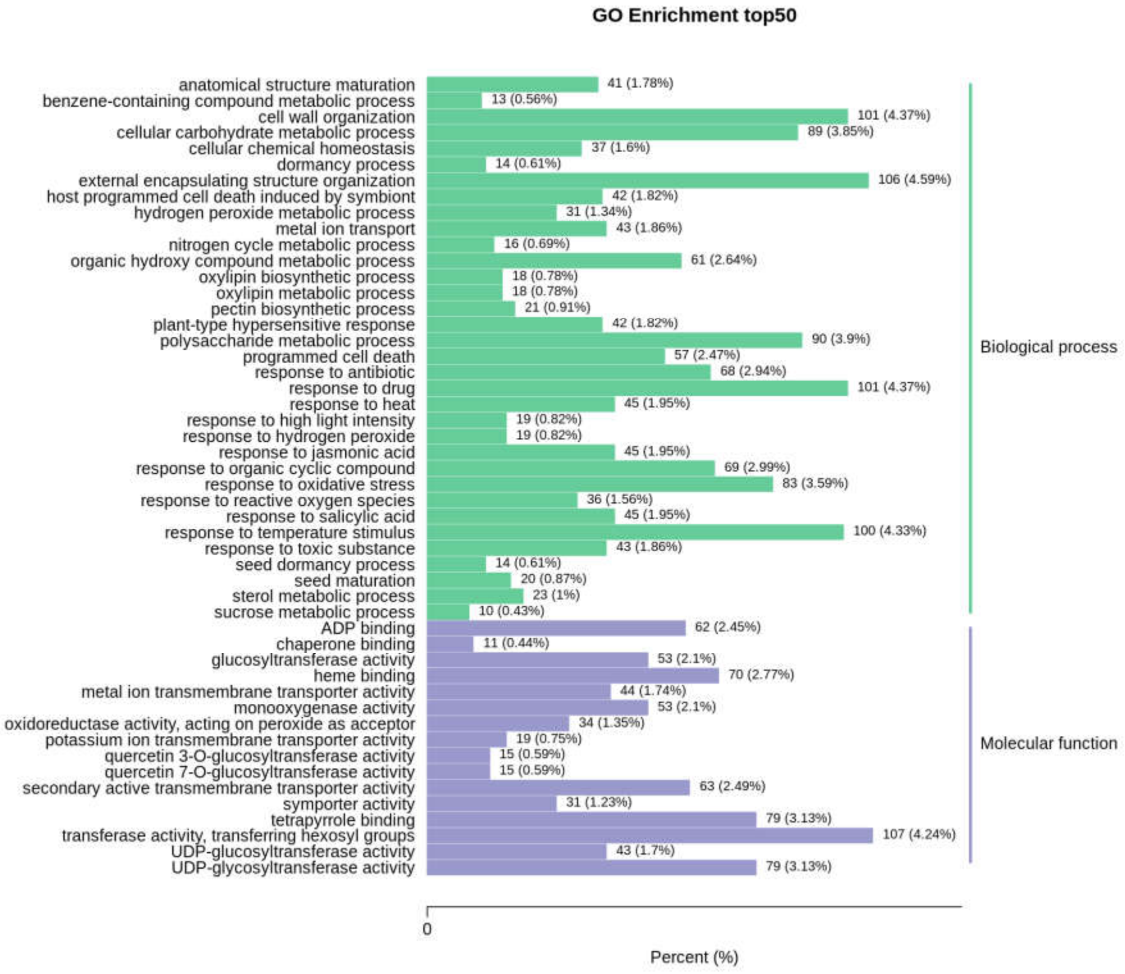

Figure S5: GO enrichment analysis of differentially expressed genes

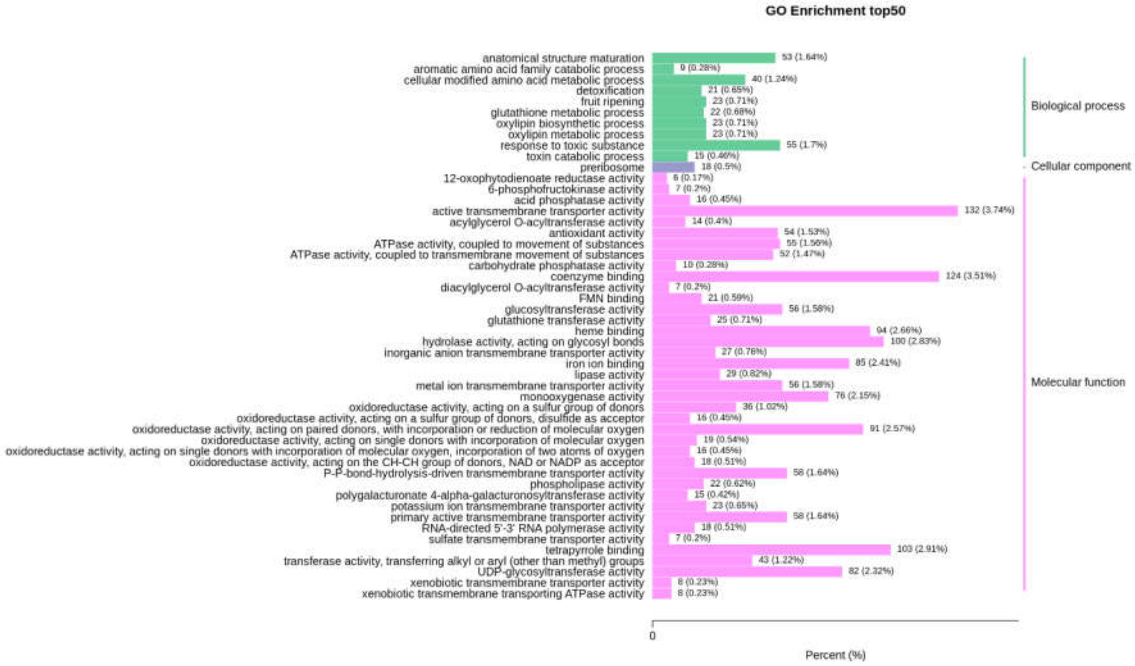

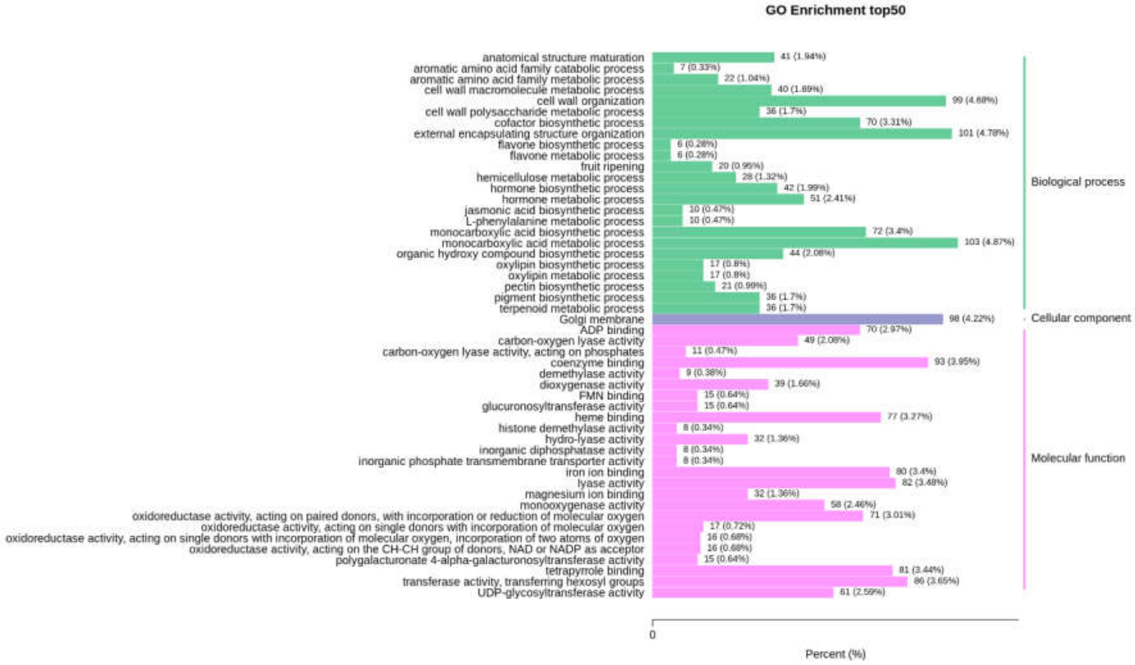

Figure S5: GO enrichment analysis of differentially expressed genes

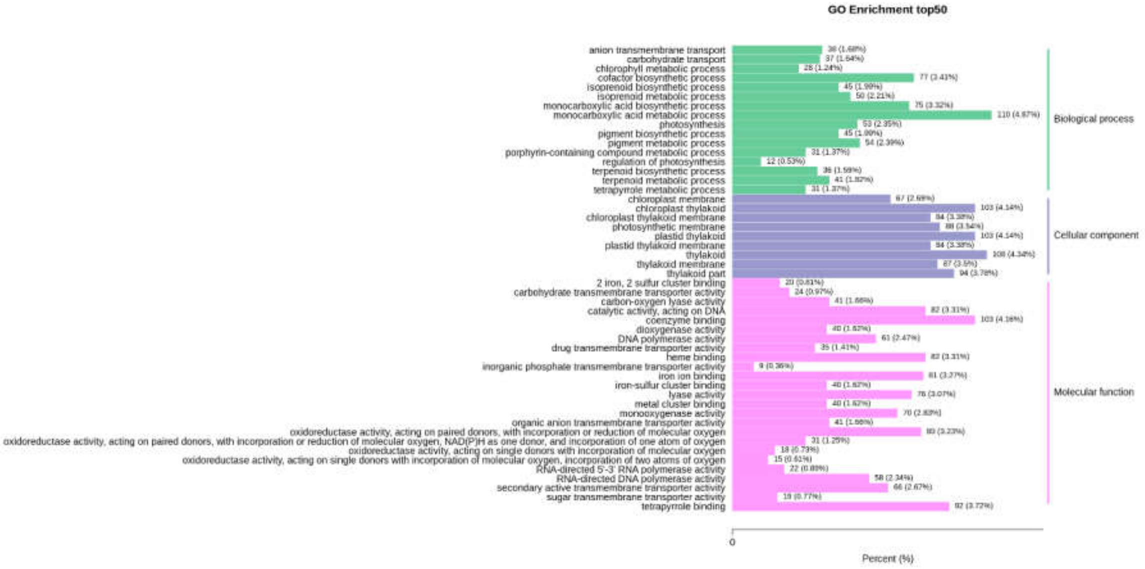

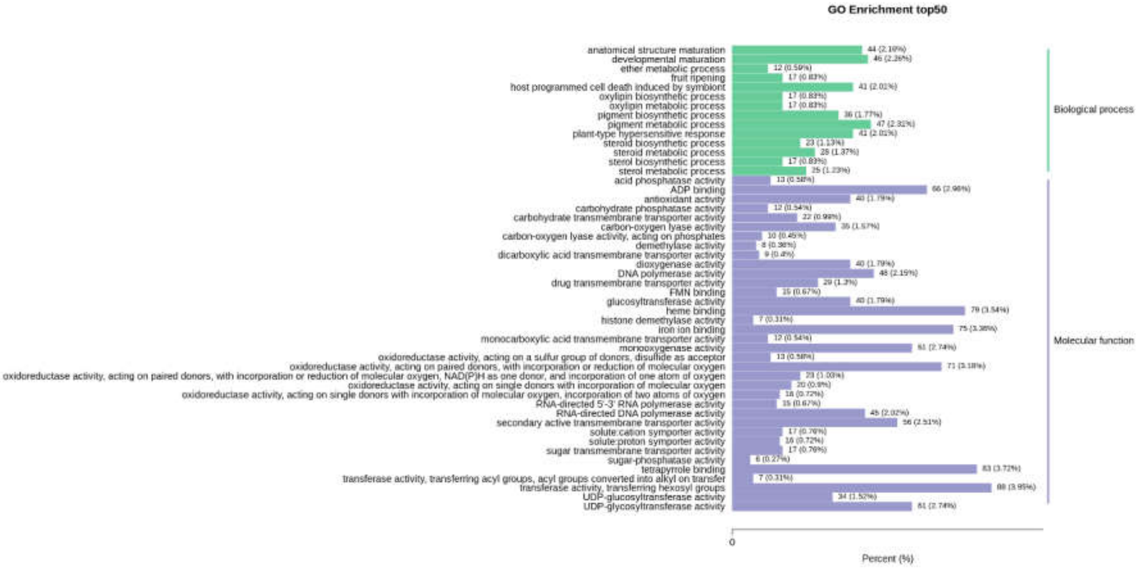

Figure S5: GO enrichment analysis of differentially expressed genes

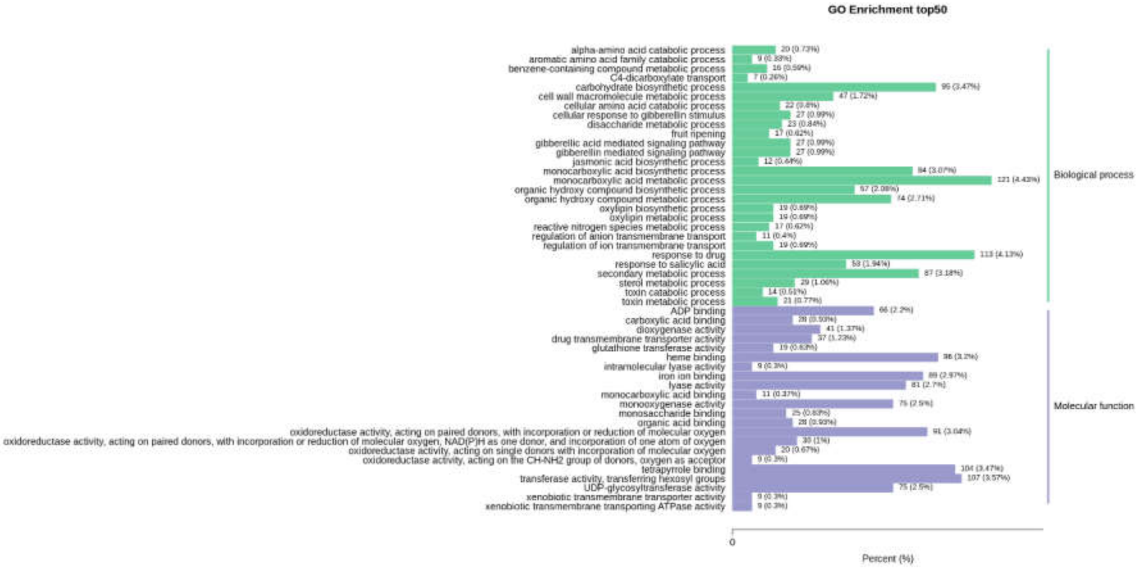

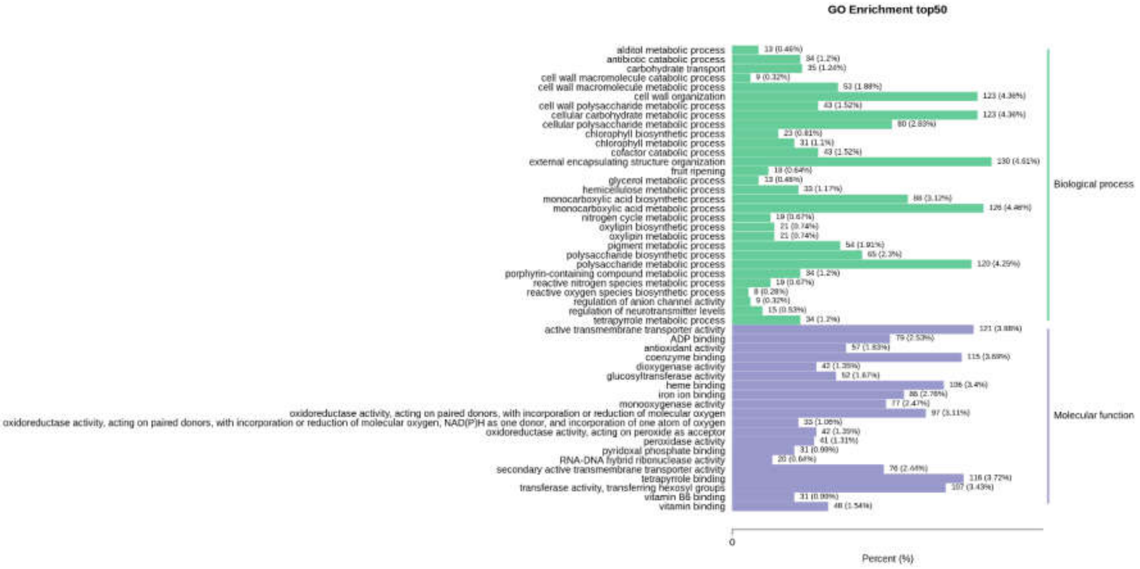

Figure S5: GO enrichment analysis of differentially expressed genes

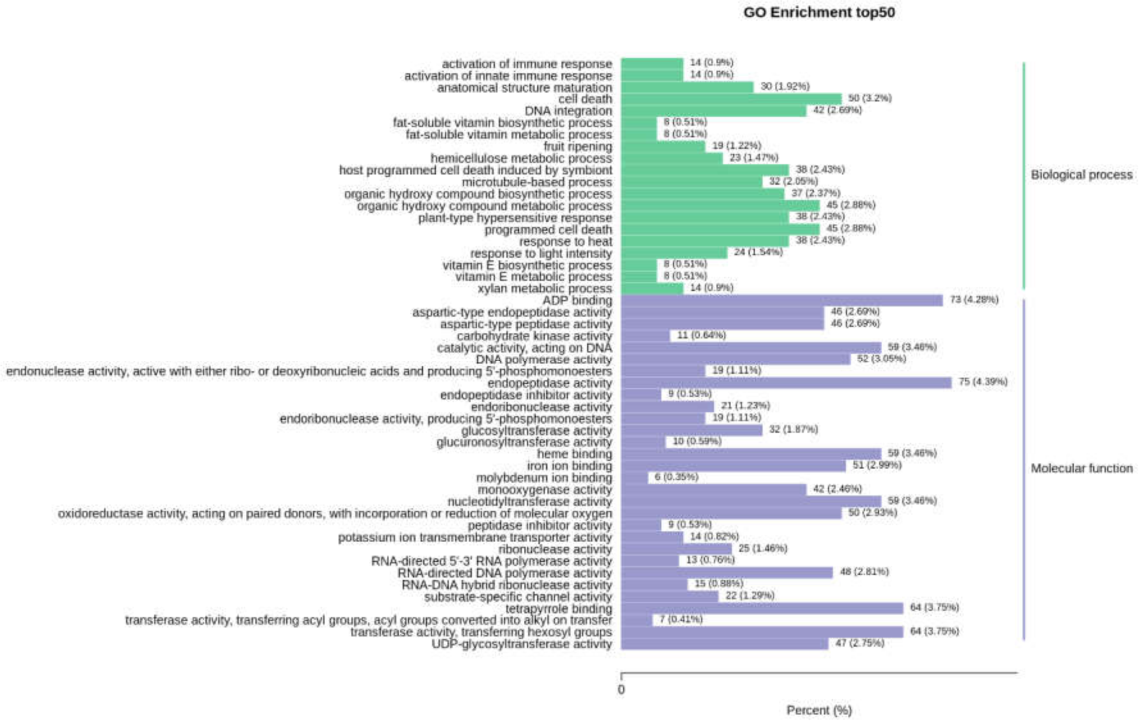

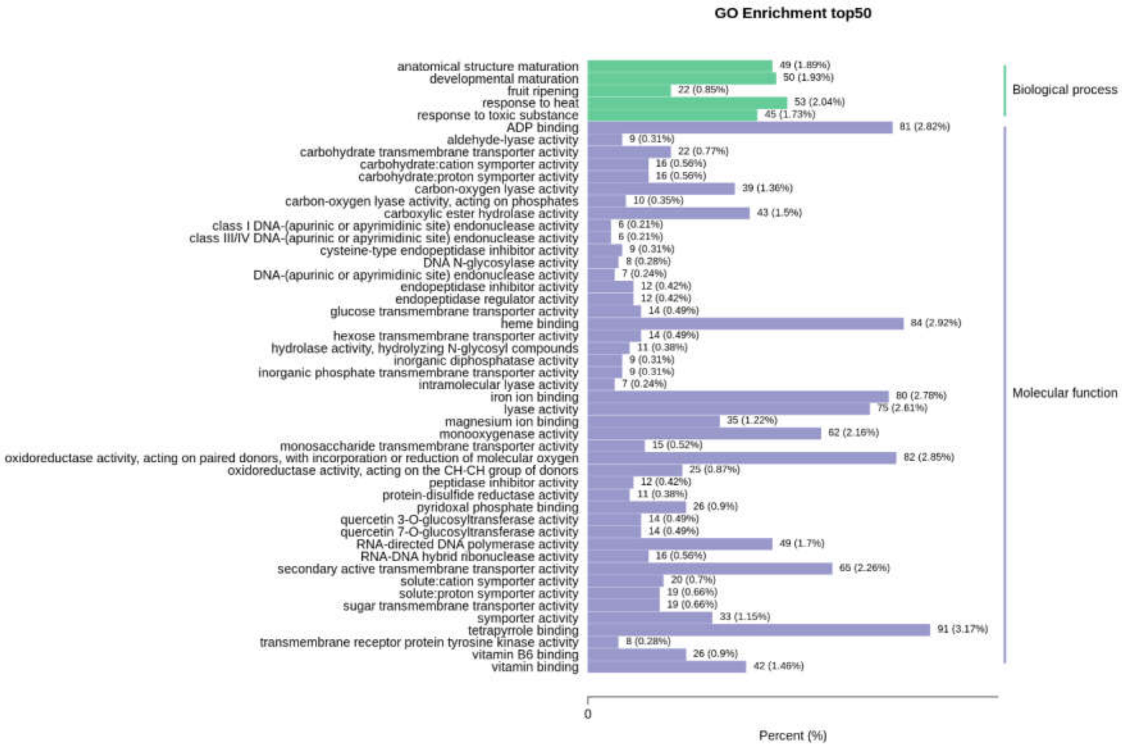

Figure S5: GO enrichment analysis of differentially expressed genes

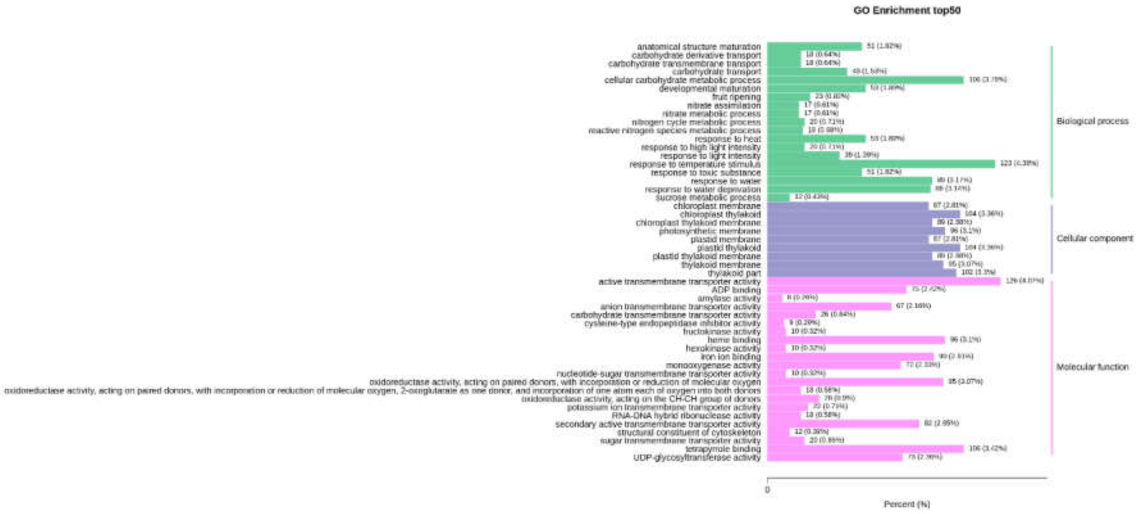

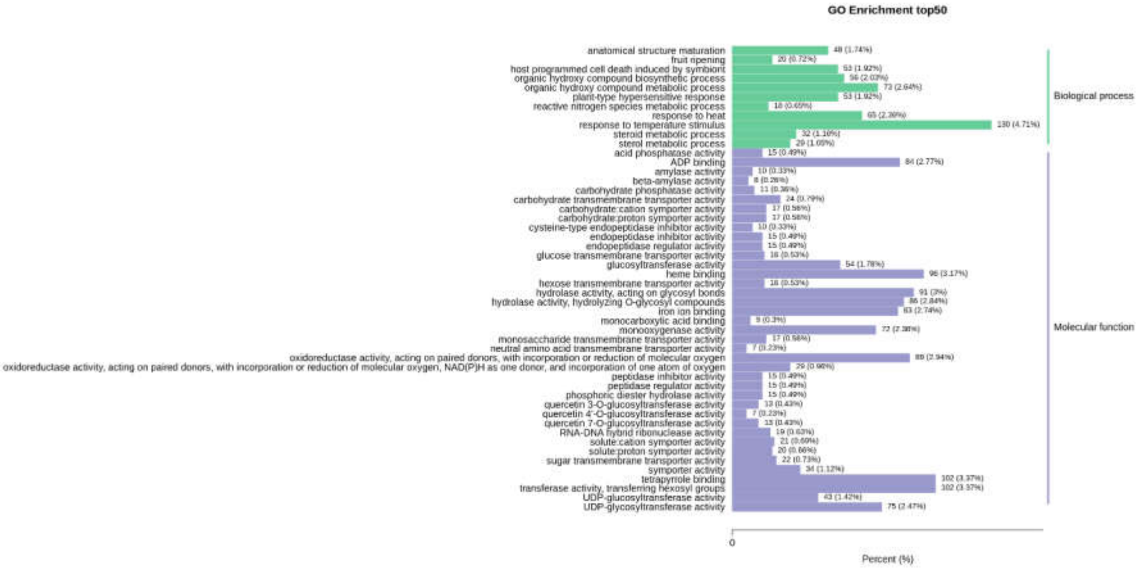

Figure S5: GO enrichment analysis of differentially expressed genes

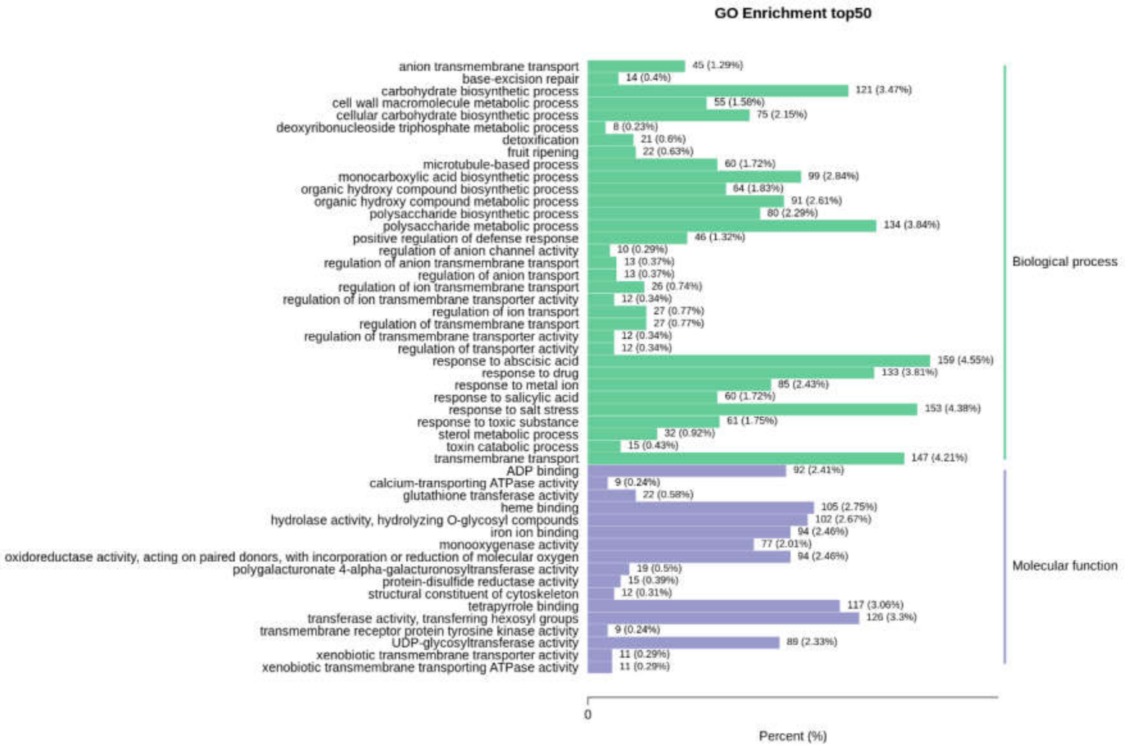

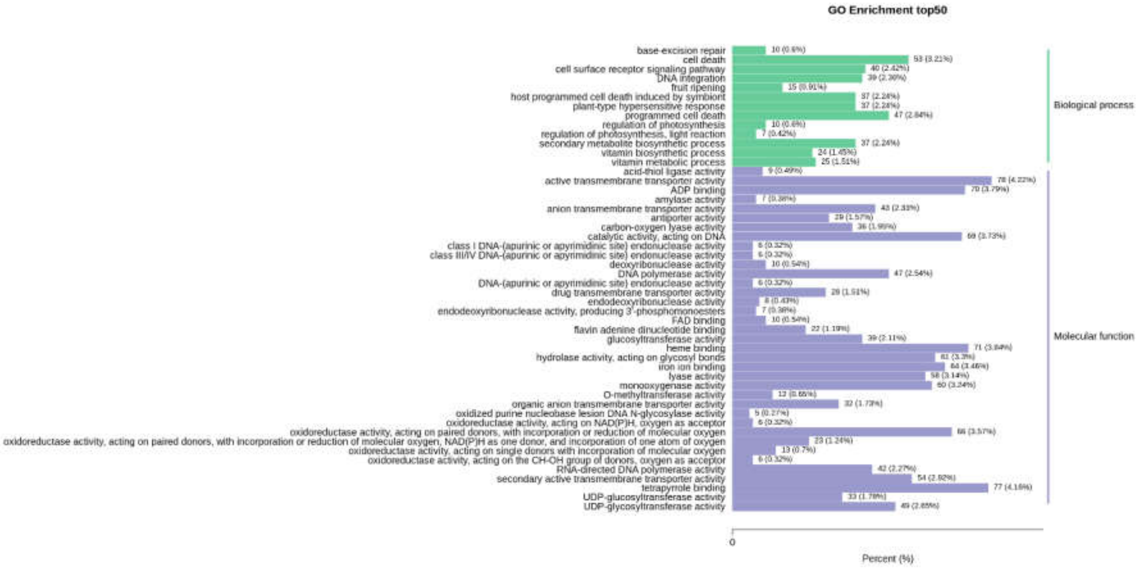

Figure S5: GO enrichment analysis of differentially expressed genes

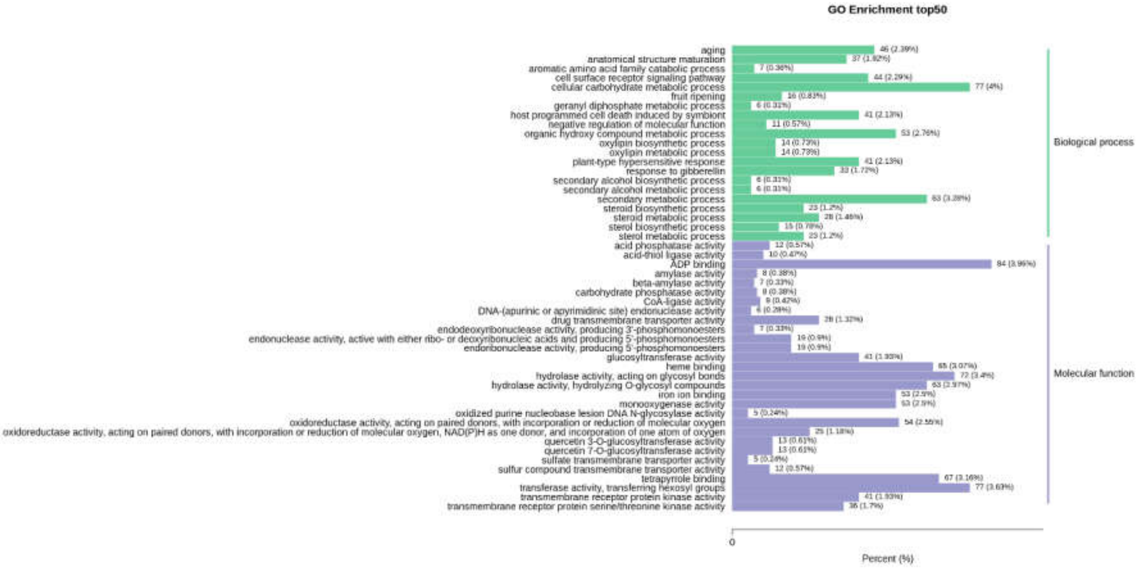

TN4_vs_TN21 Statistical plot of the number of differential genes contained in the secondary GO entry

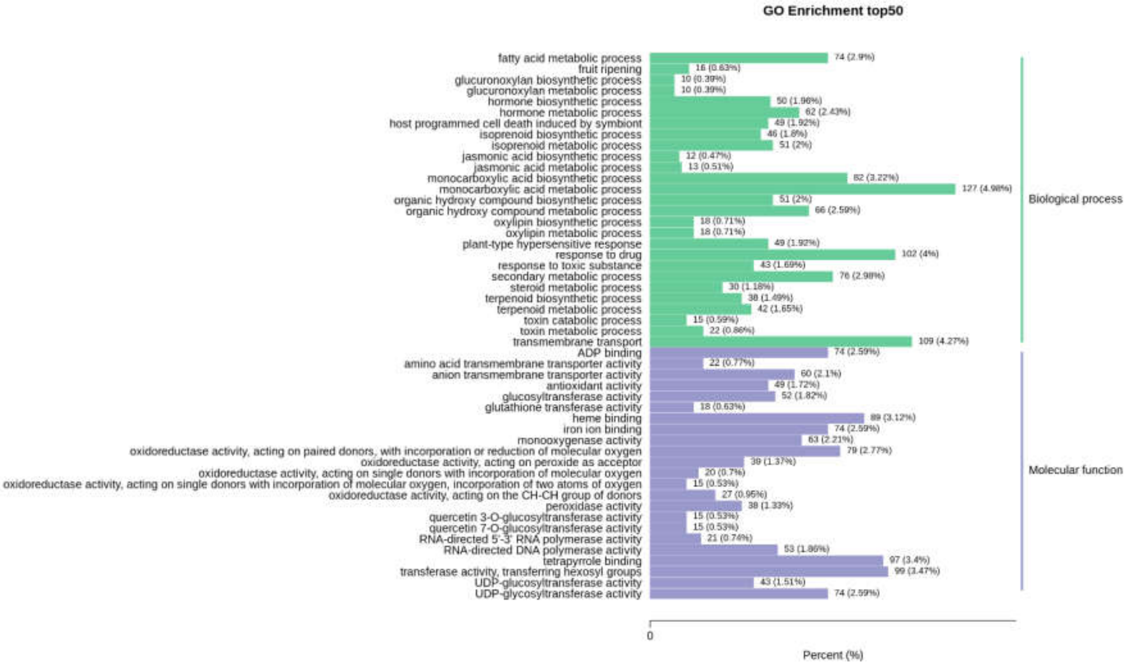

TN4_vs_TN23 Statistical plot of the number of differential genes contained in the secondary GO entry

Figure S5: GO enrichment analysis of differentially expressed genes

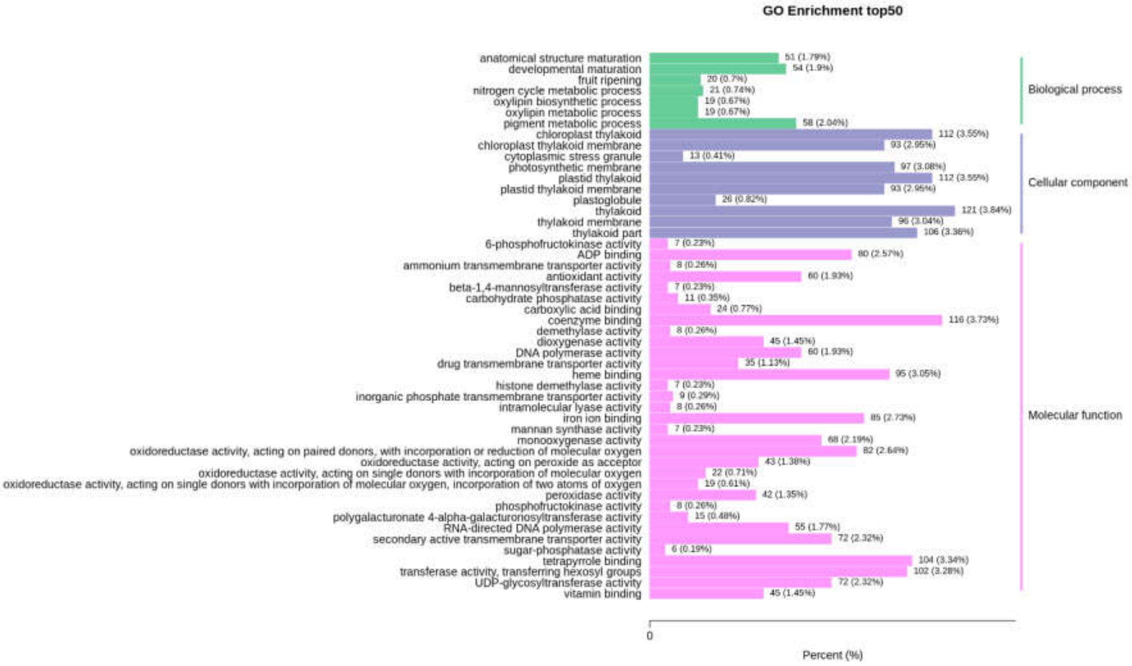

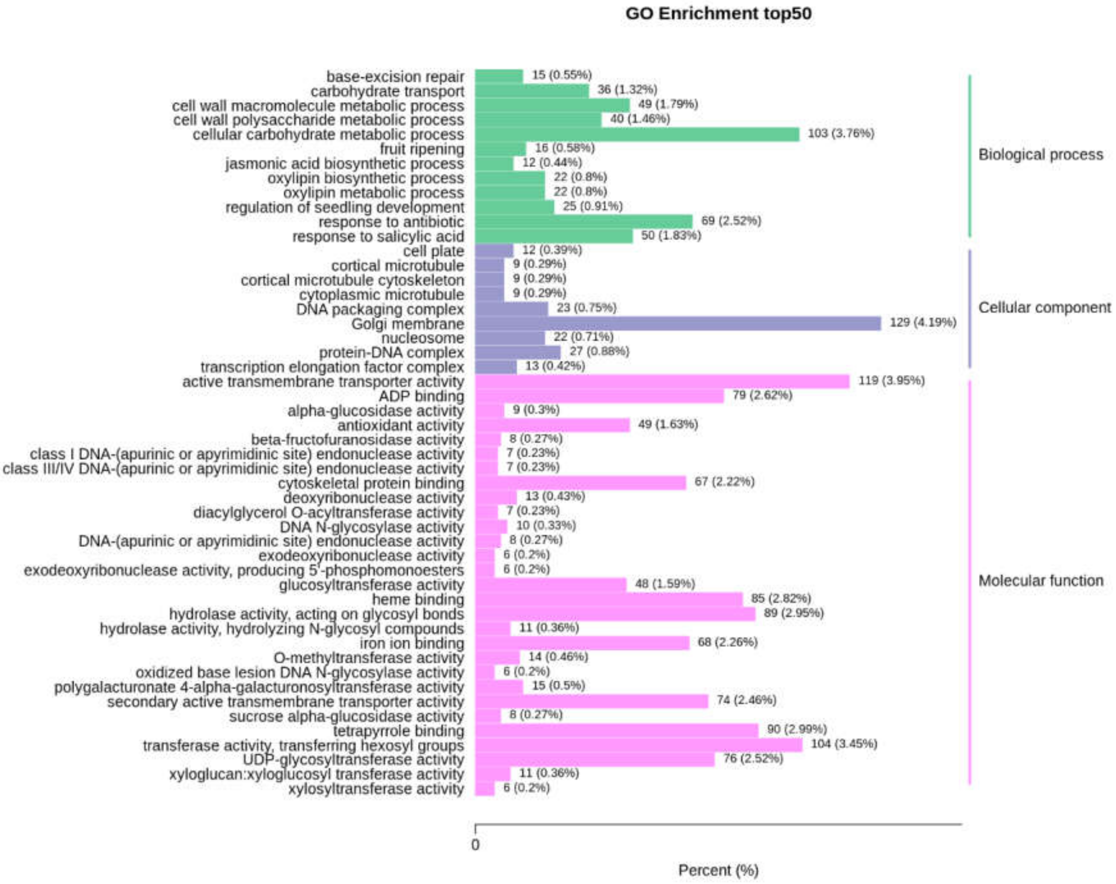

Figure S5: GO enrichment analysis of differentially expressed genes

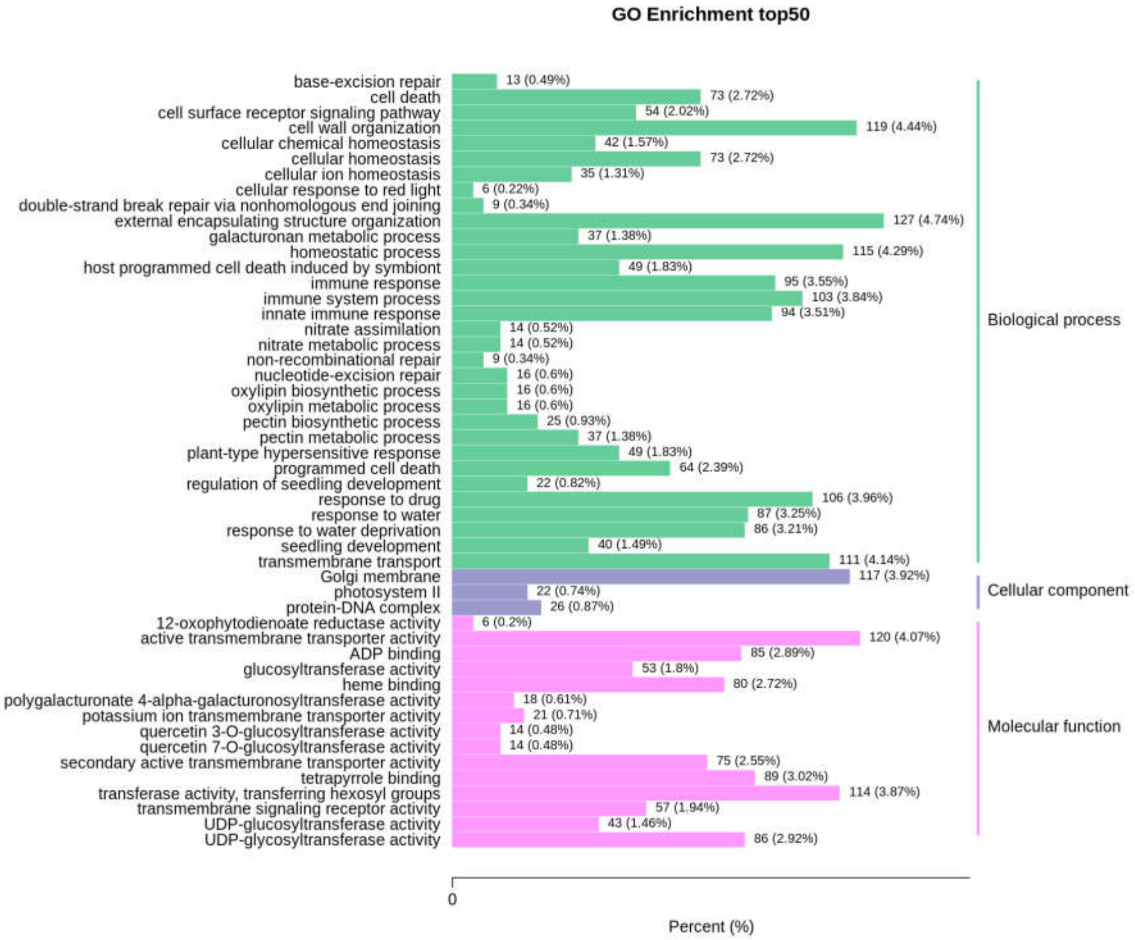

Figure S5: GO enrichment analysis of differentially expressed genes

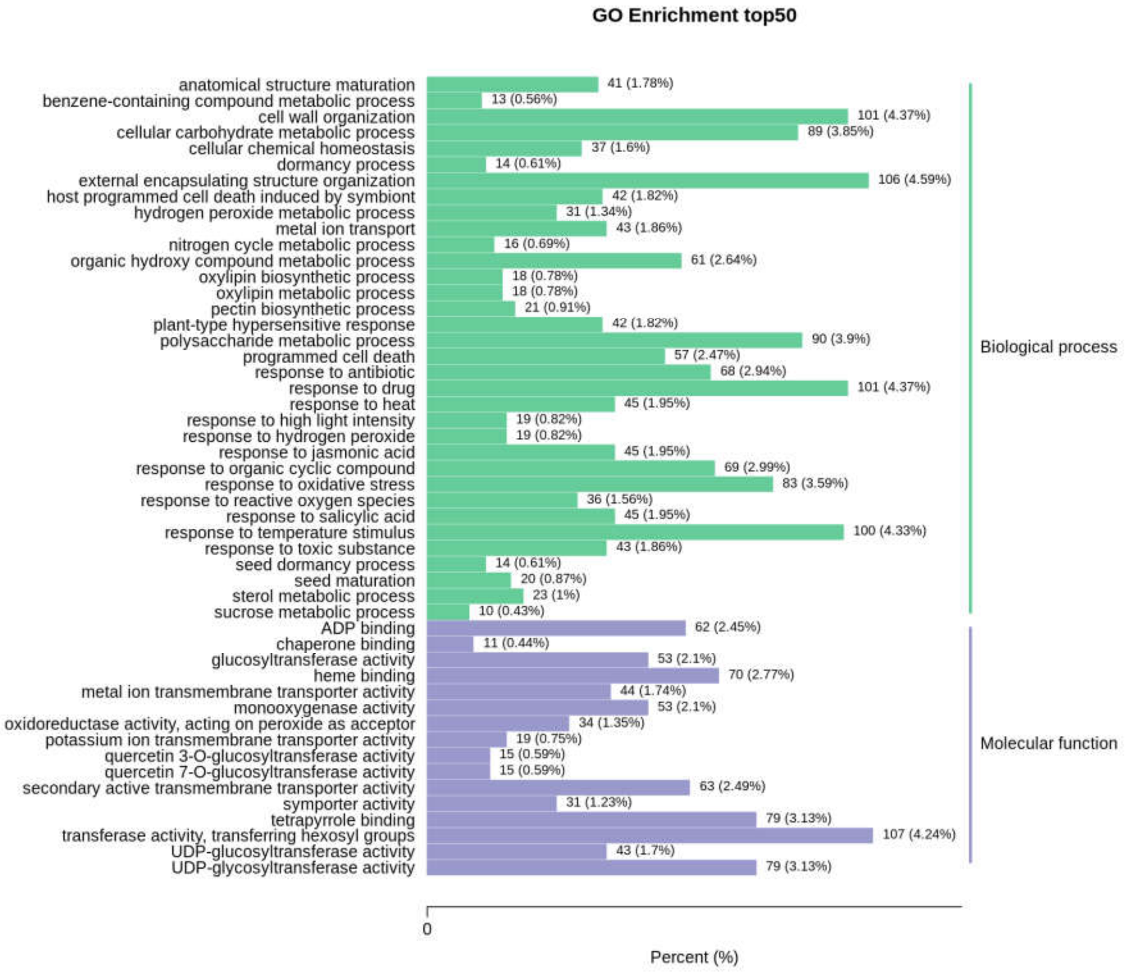

Figure S5: GO enrichment analysis of differentially expressed genes

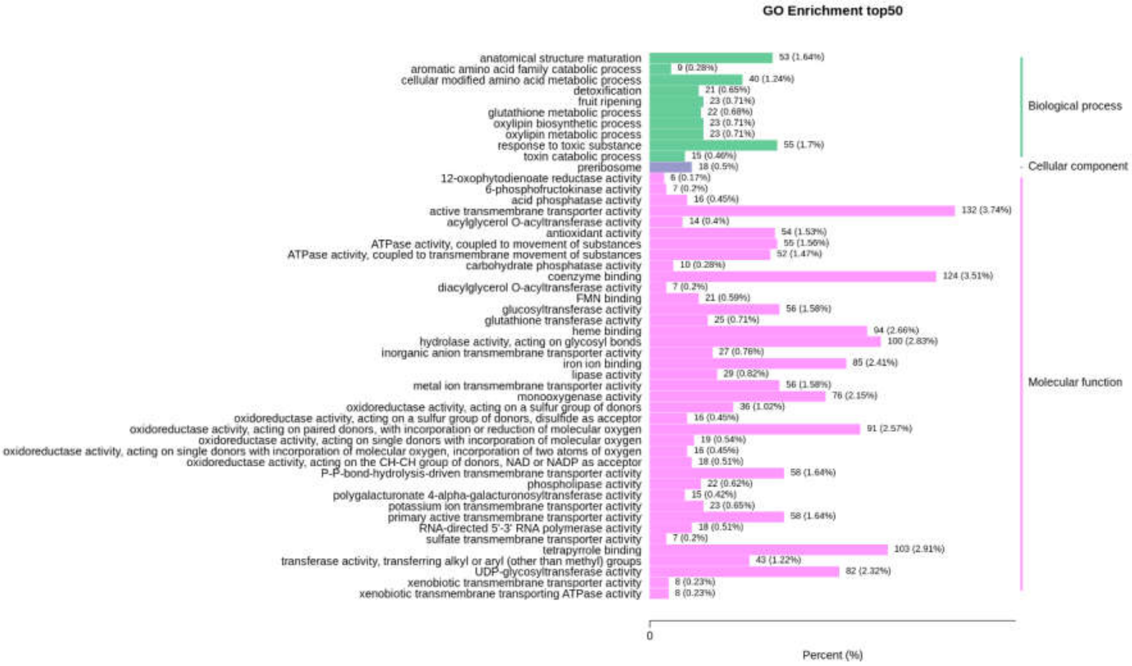

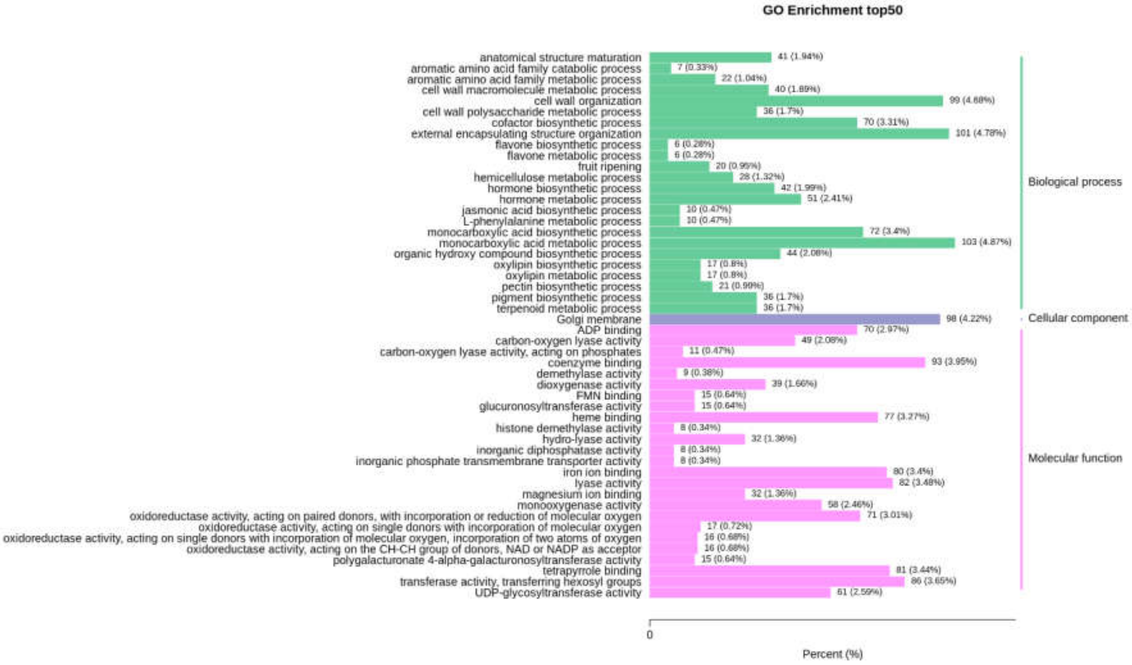

Figure S5: GO enrichment analysis of differentially expressed genes

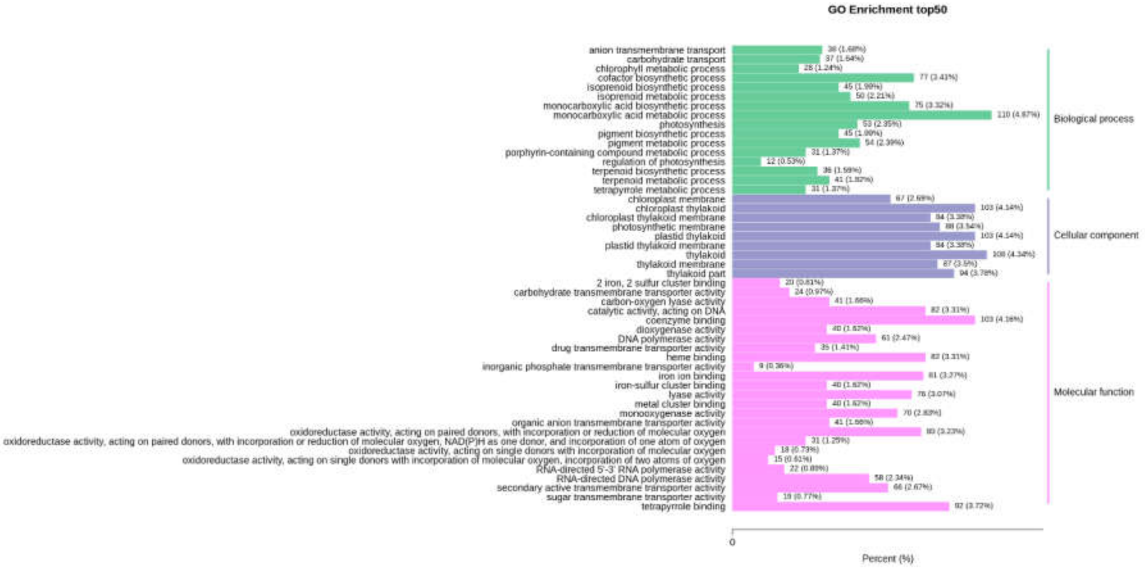

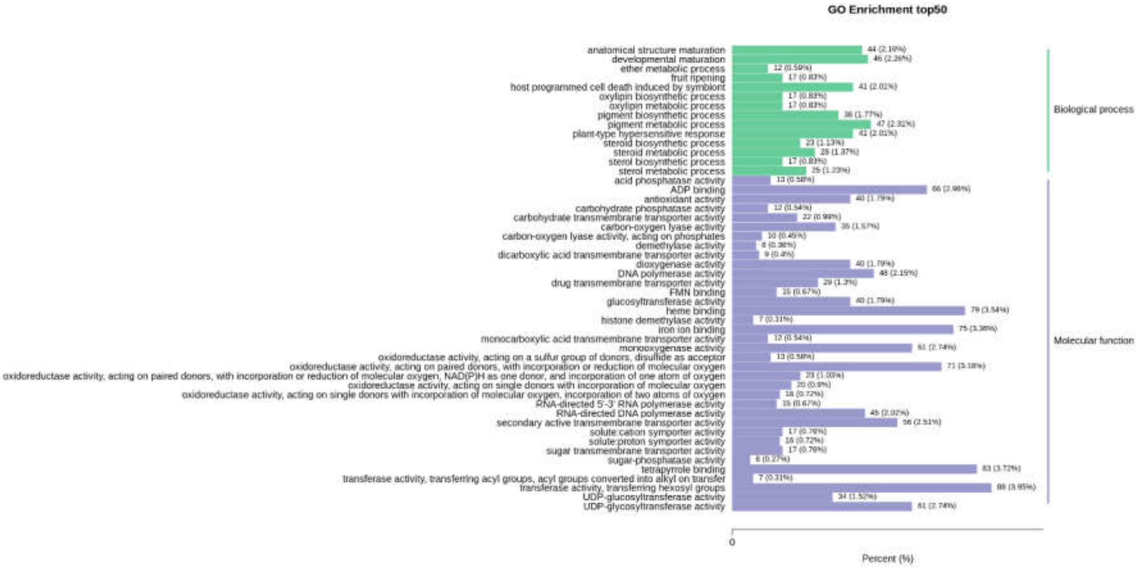

Figure S5: GO enrichment analysis of differentially expressed genes

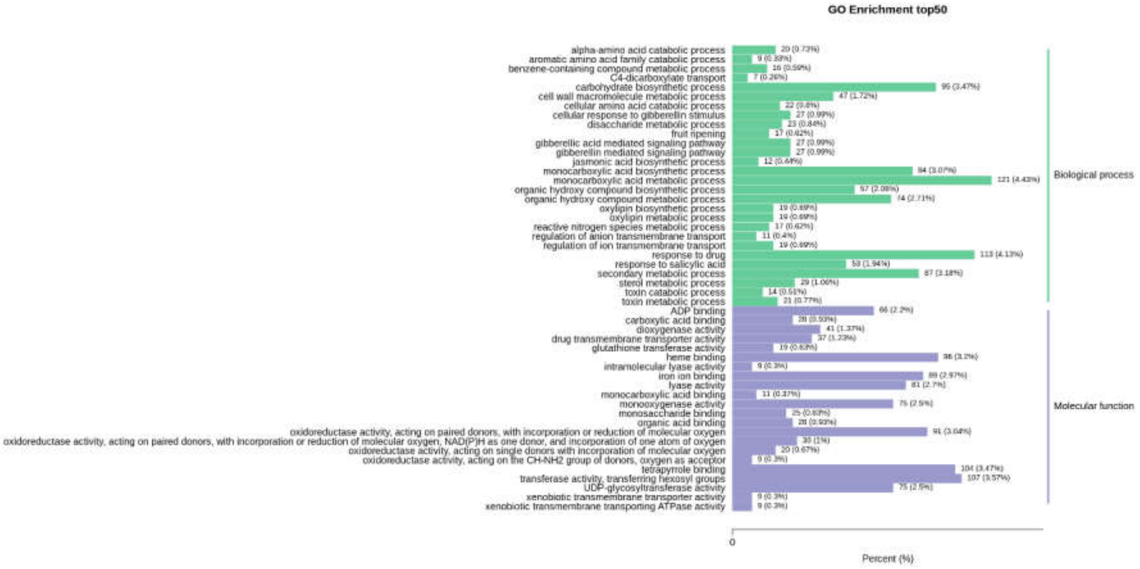

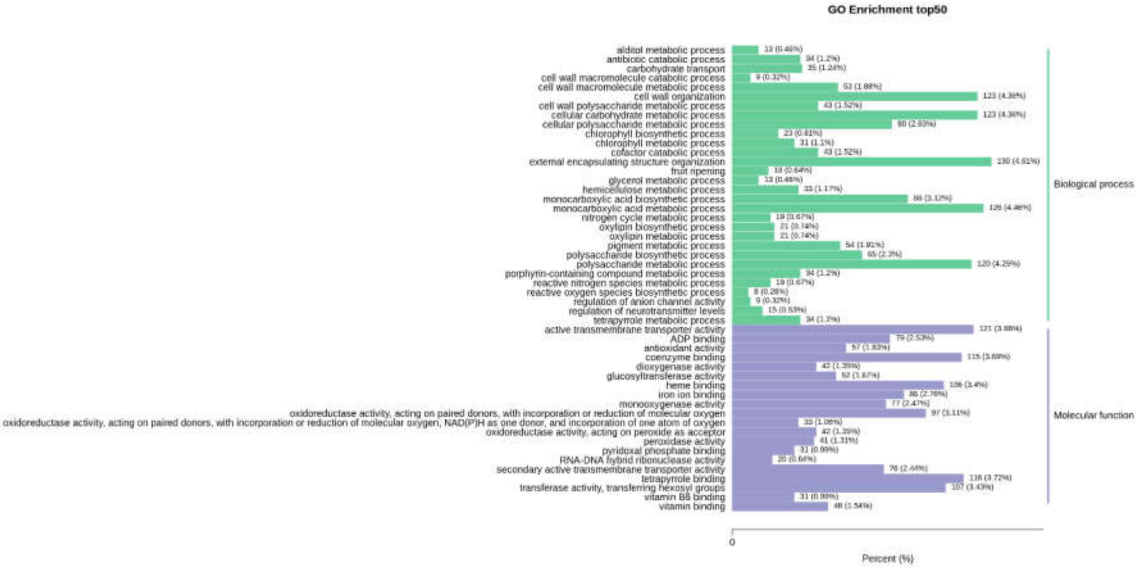

Figure S5: GO enrichment analysis of differentially expressed genes

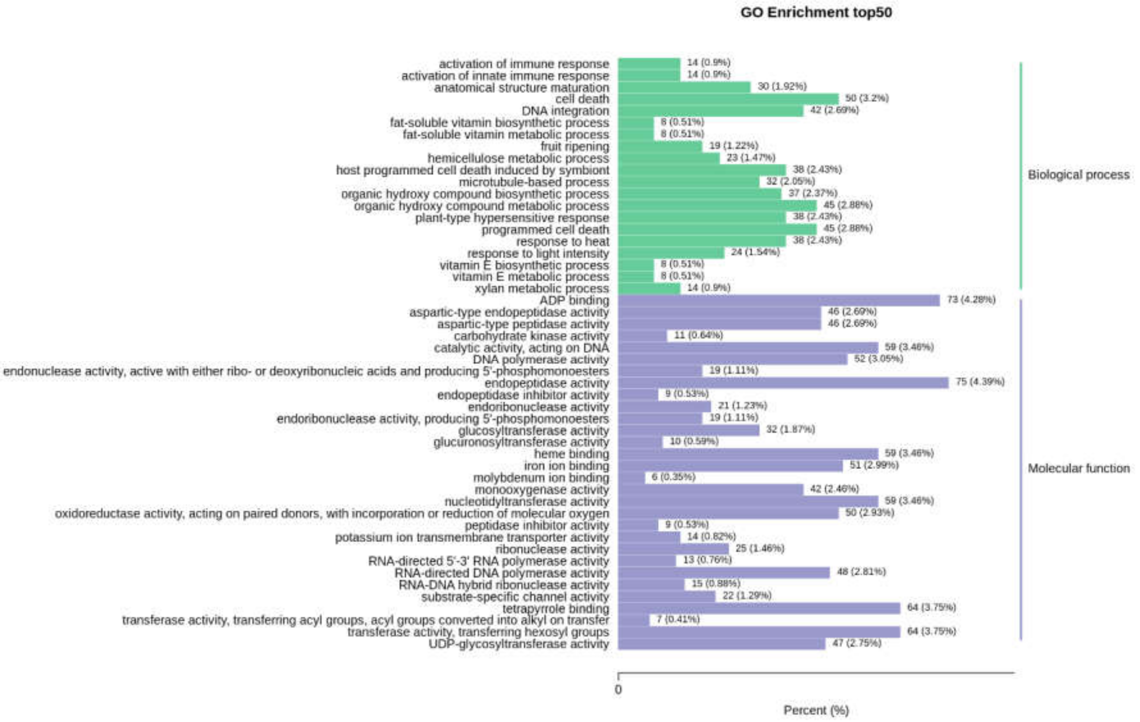

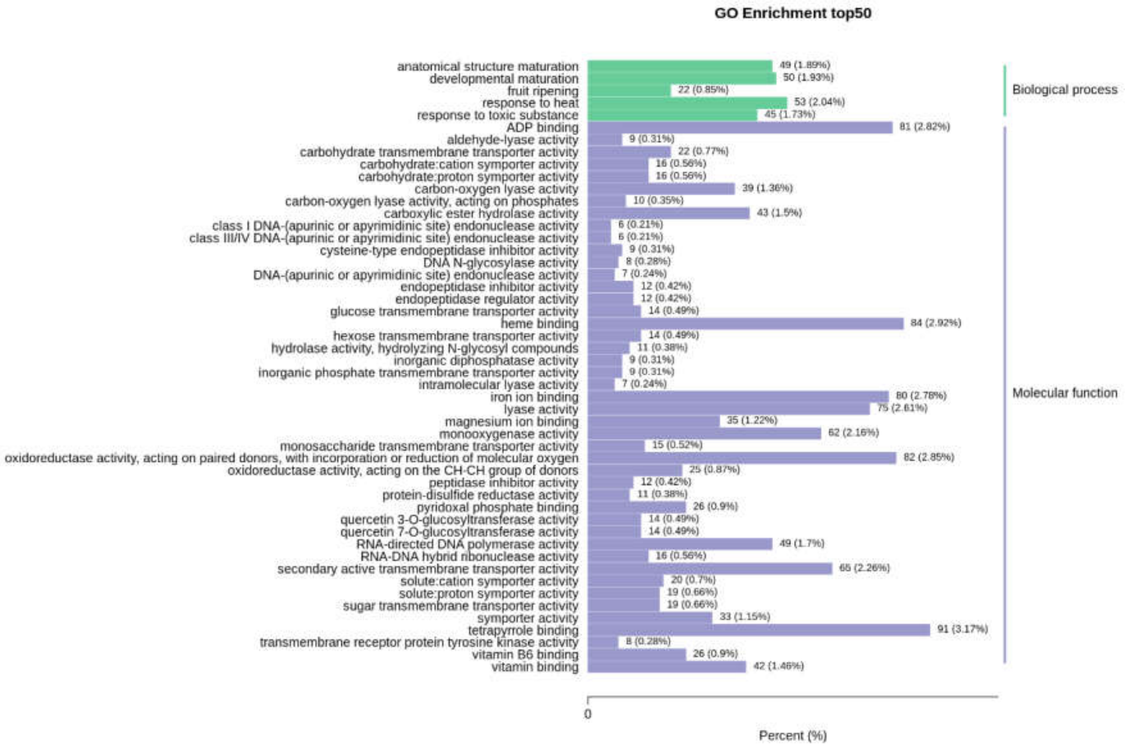

Figure S5: GO enrichment analysis of differentially expressed genes

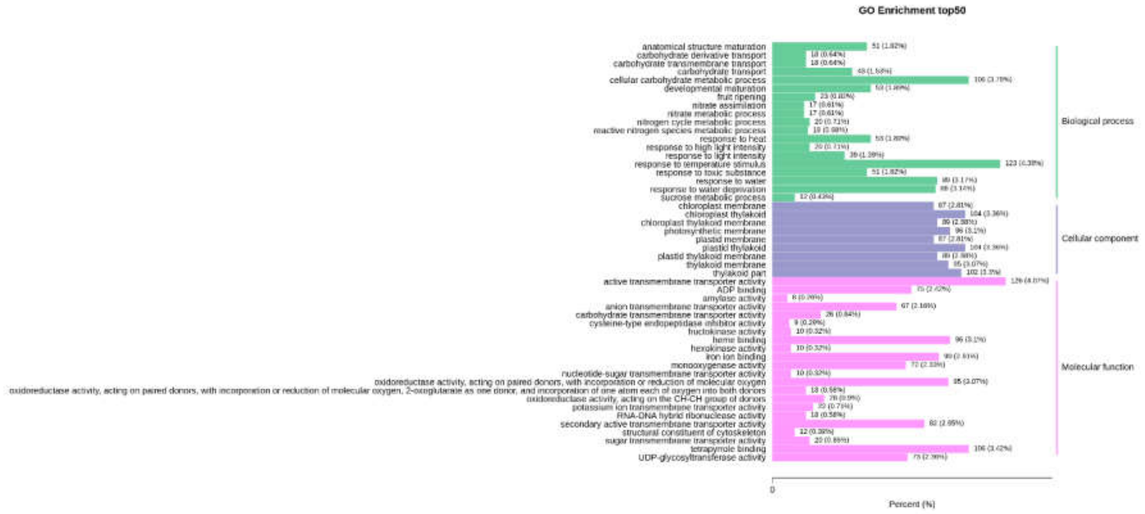

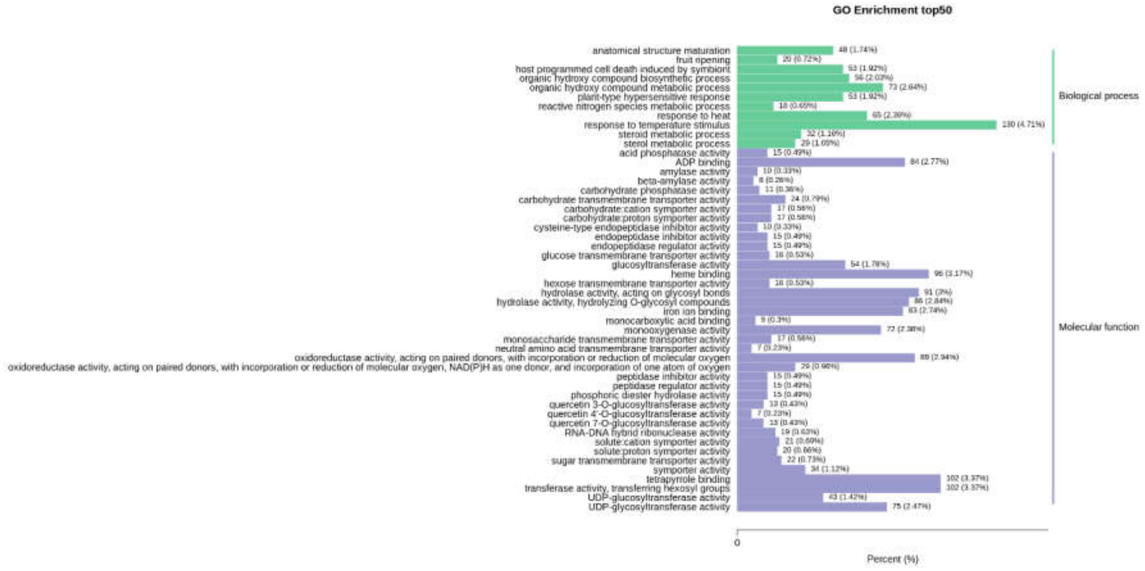

Figure S5: GO enrichment analysis of differentially expressed genes

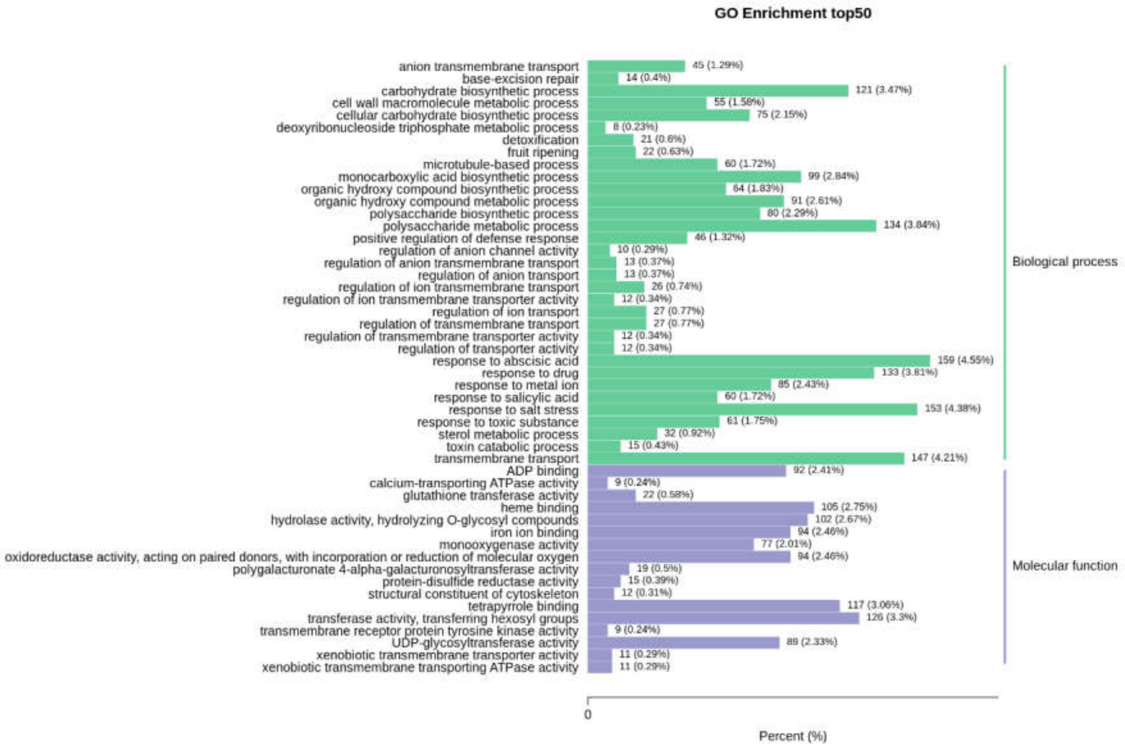

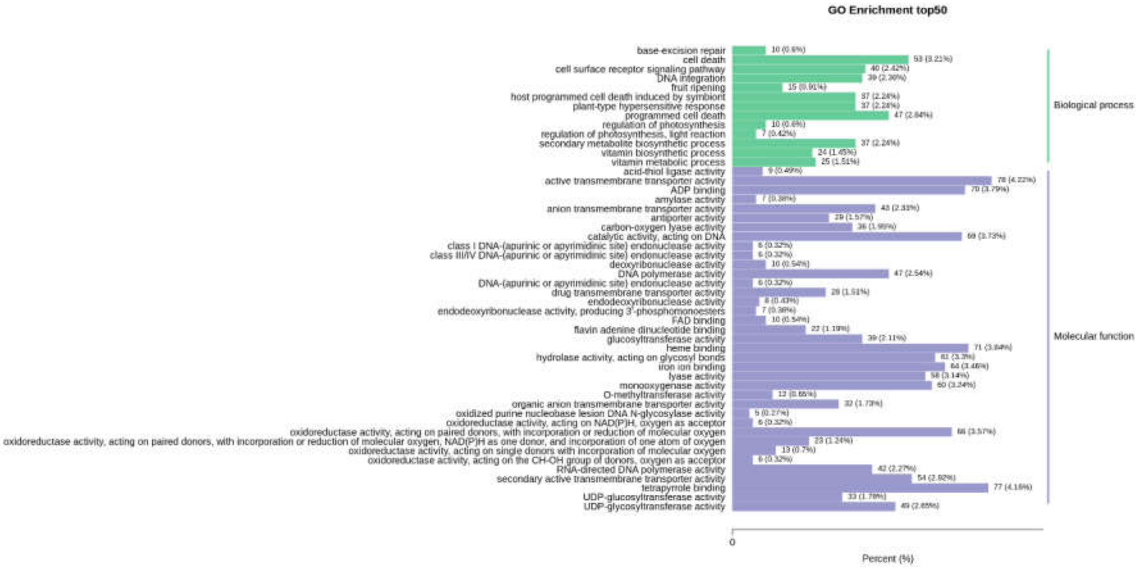

Figure S5: GO enrichment analysis of differentially expressed genes

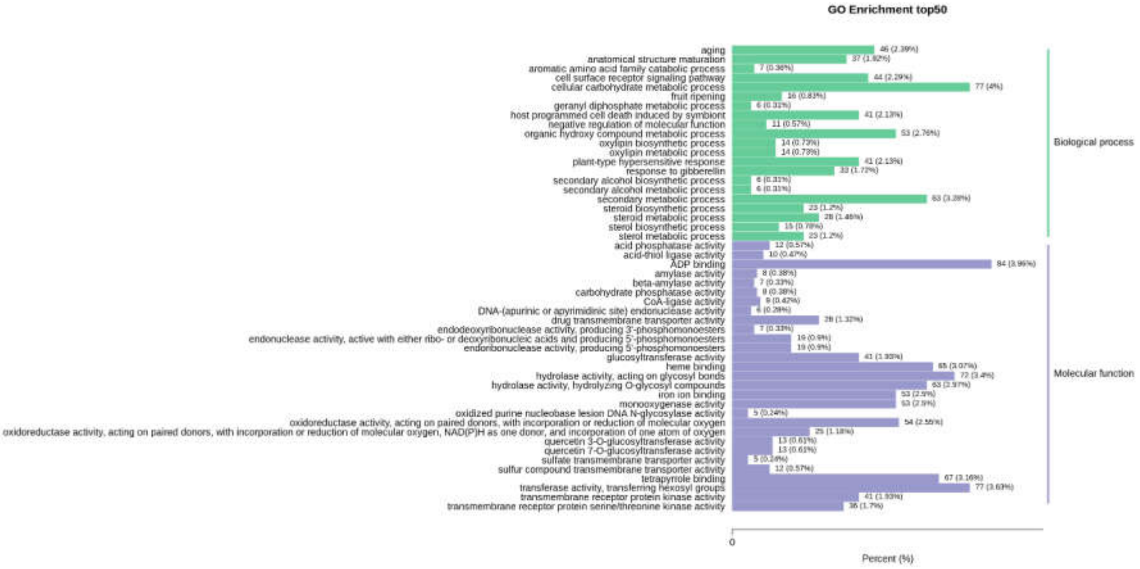

TN4_vs_TN23 Statistical plot of the number of differential genes contained in the secondary GO entry

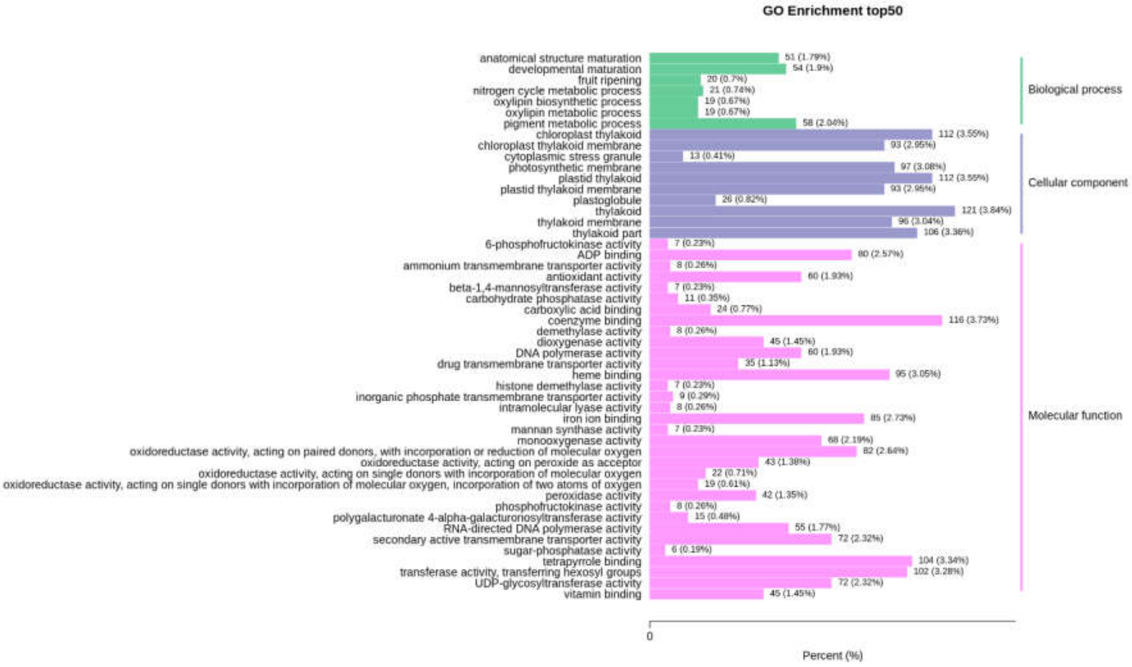

Figure S5: GO enrichment analysis of differentially expressed genes

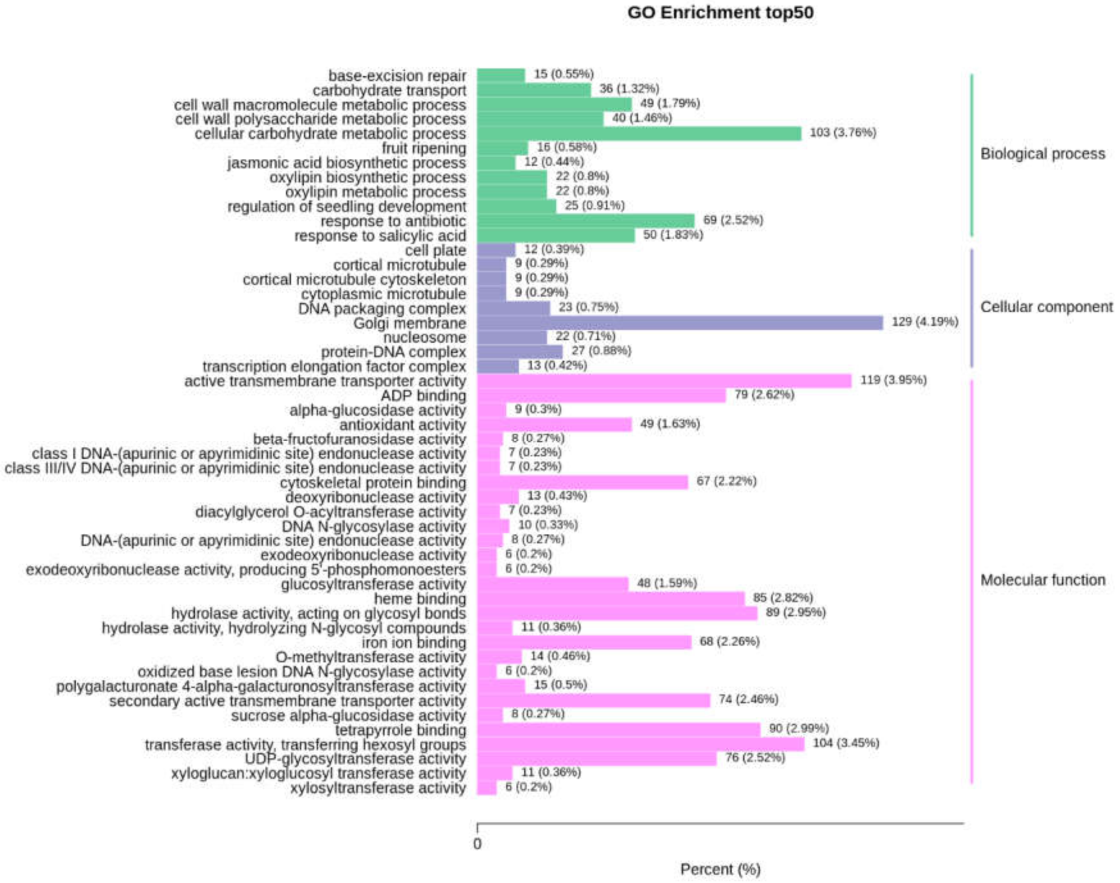

Figure S5: GO enrichment analysis of differentially expressed genes

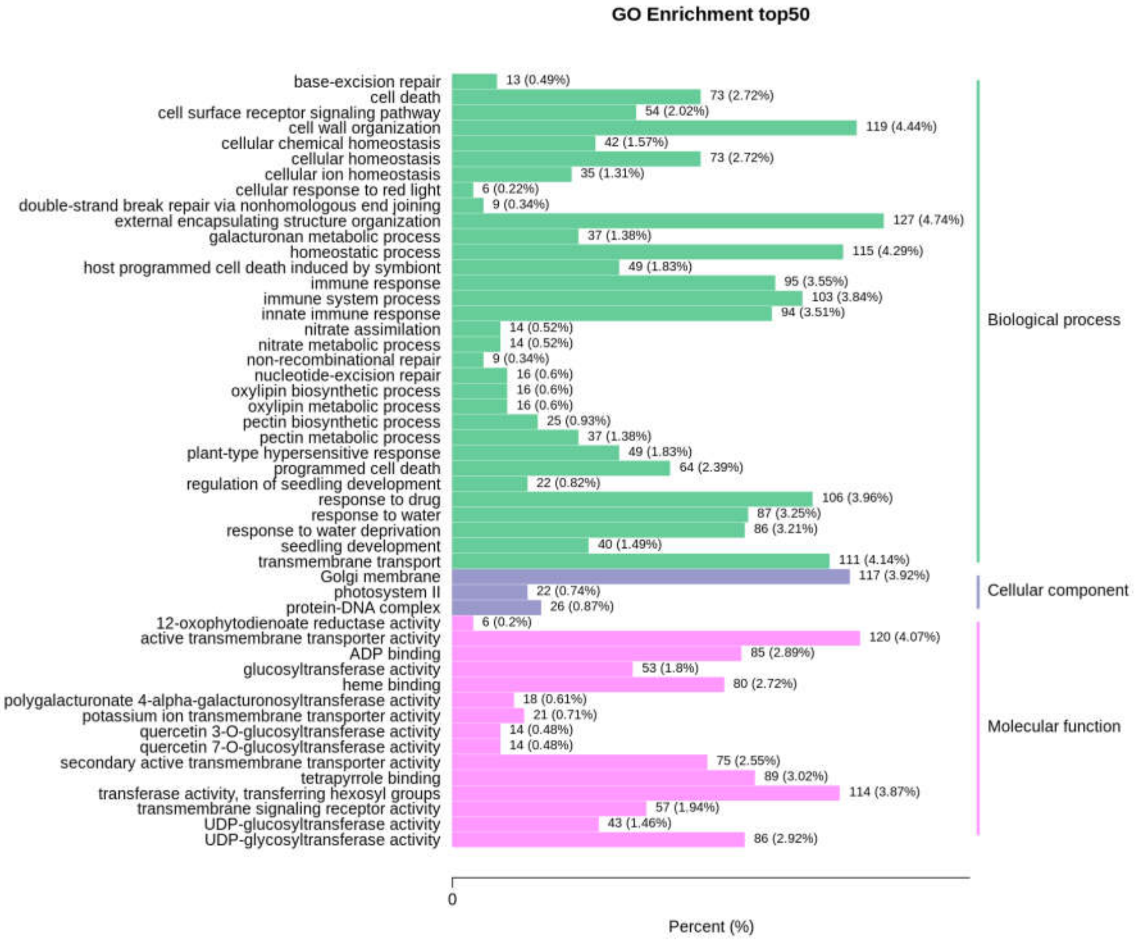

Figure S5: GO enrichment analysis of differentially expressed genes

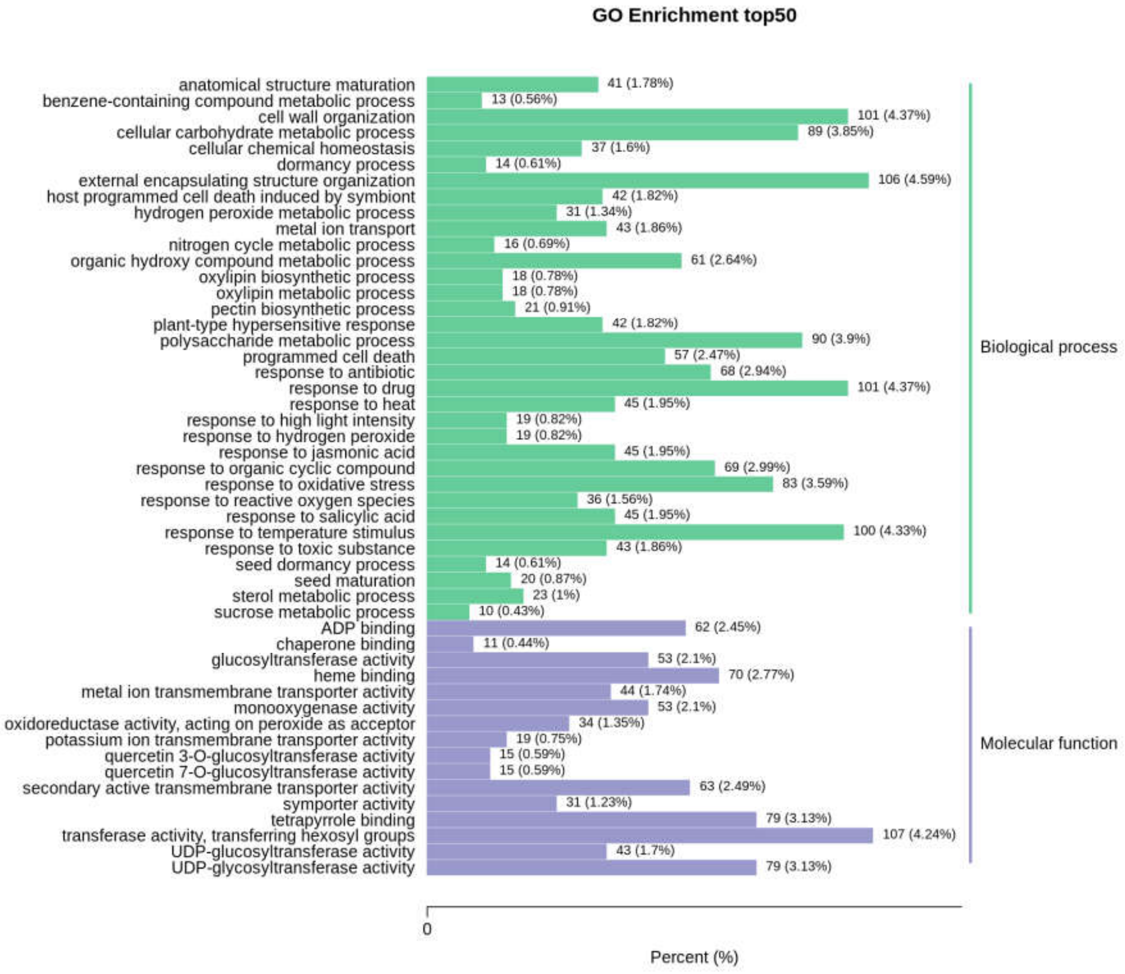

Figure S5: GO enrichment analysis of differentially expressed genes

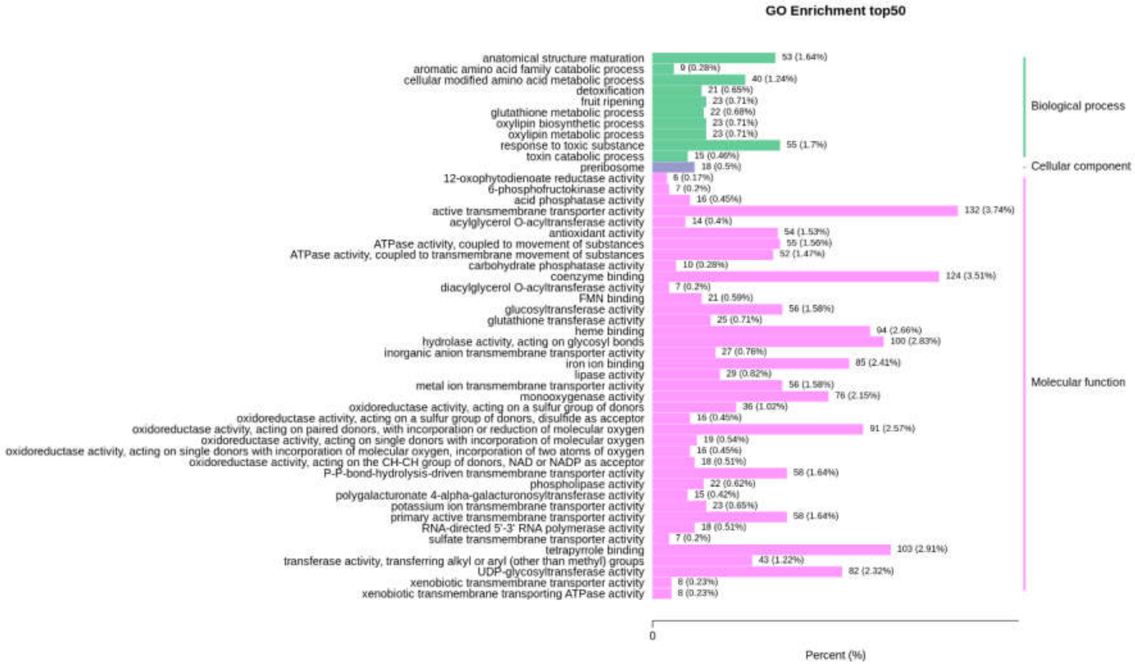

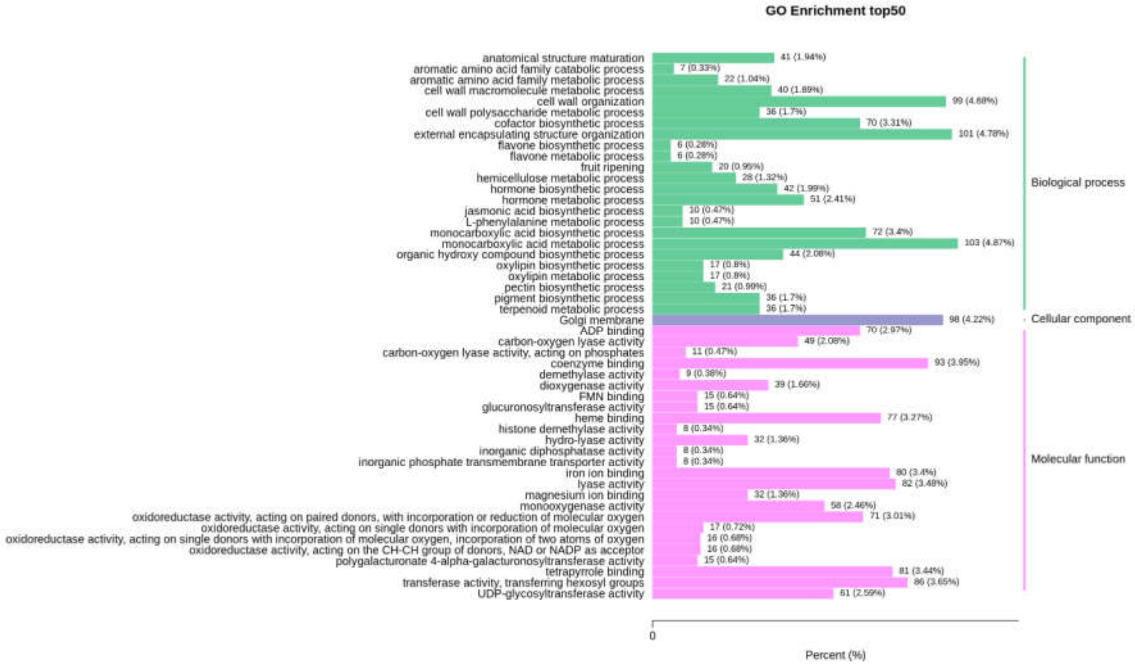

Figure S5: GO enrichment analysis of differentially expressed genes

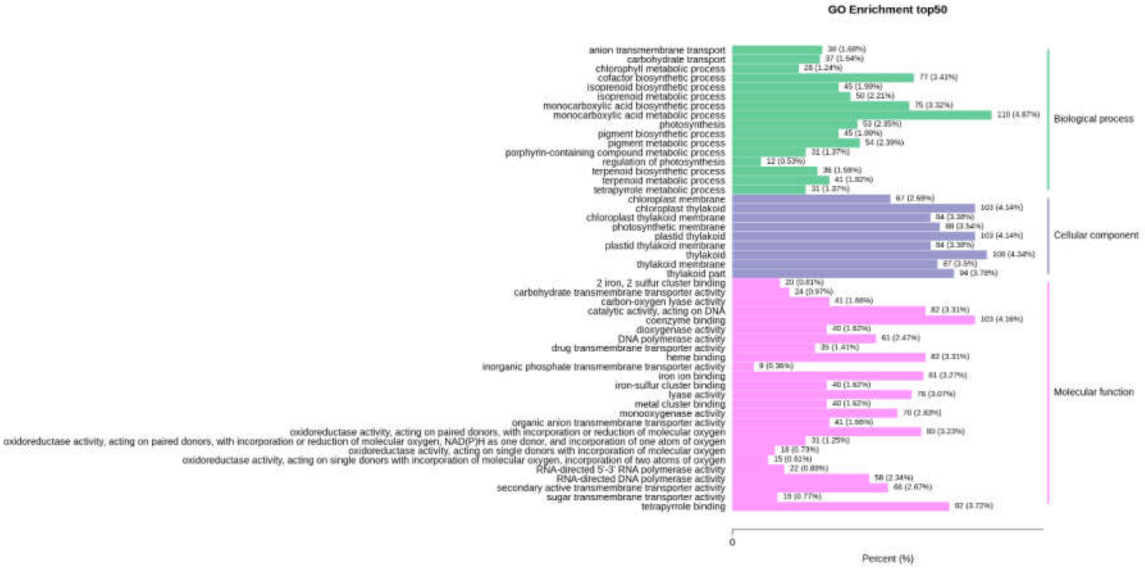

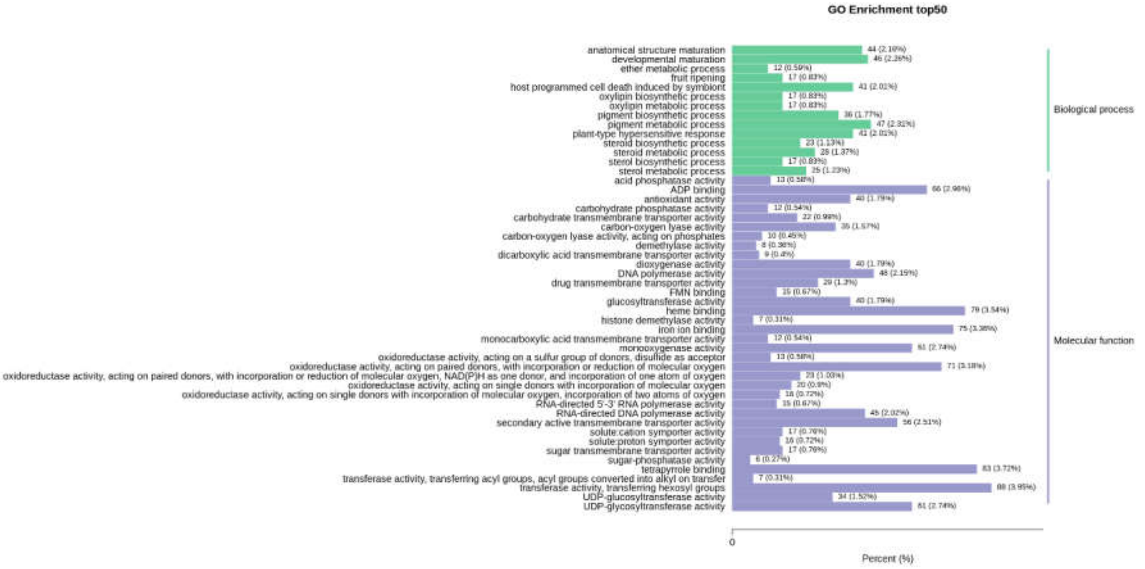

Figure S5: GO enrichment analysis of differentially expressed genes

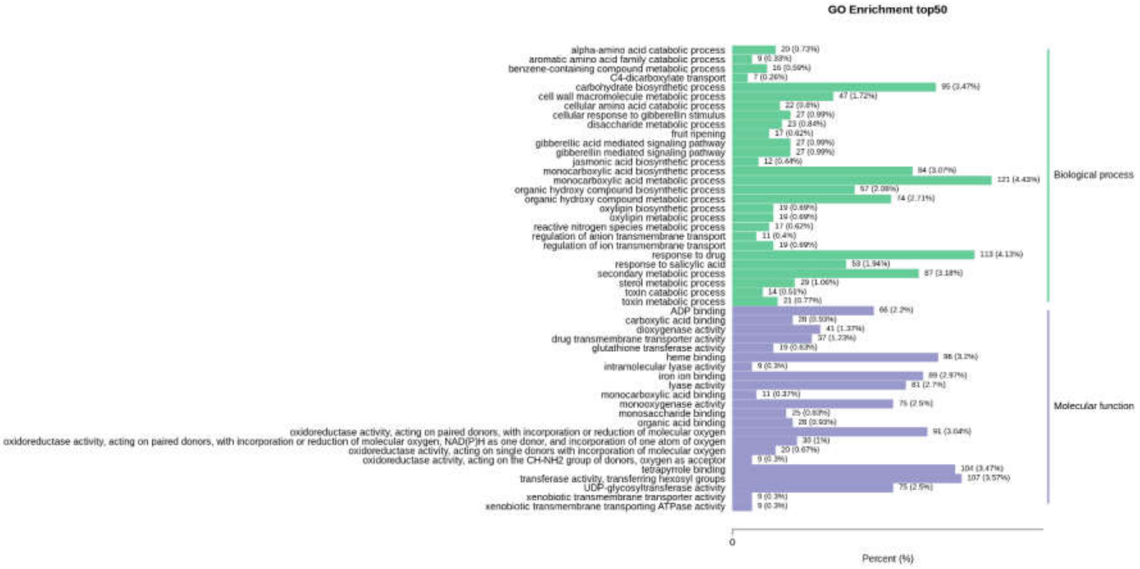

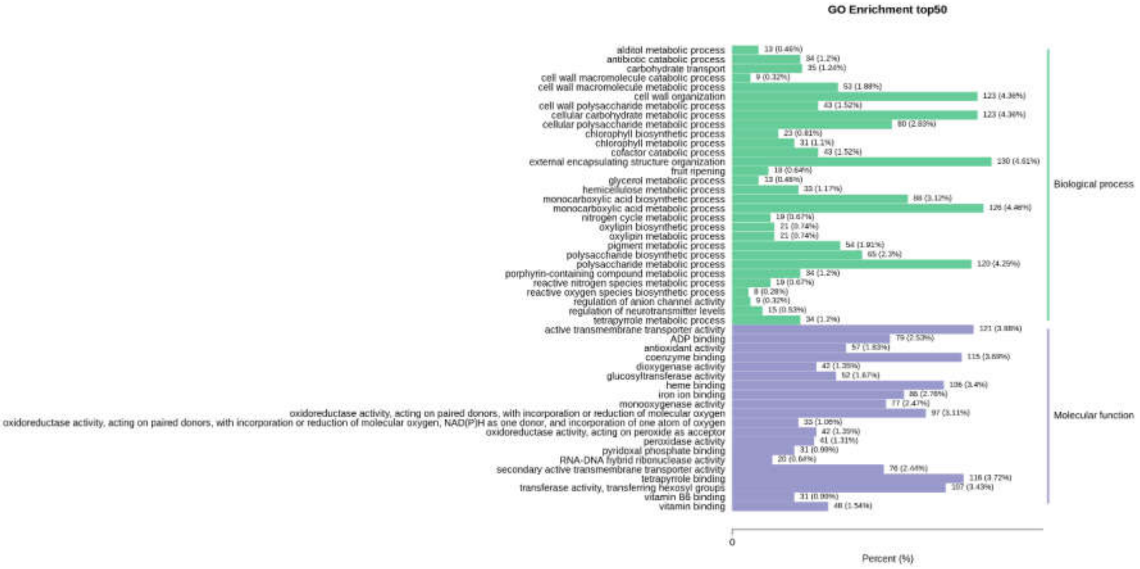

Figure S5: GO enrichment analysis of differentially expressed genes

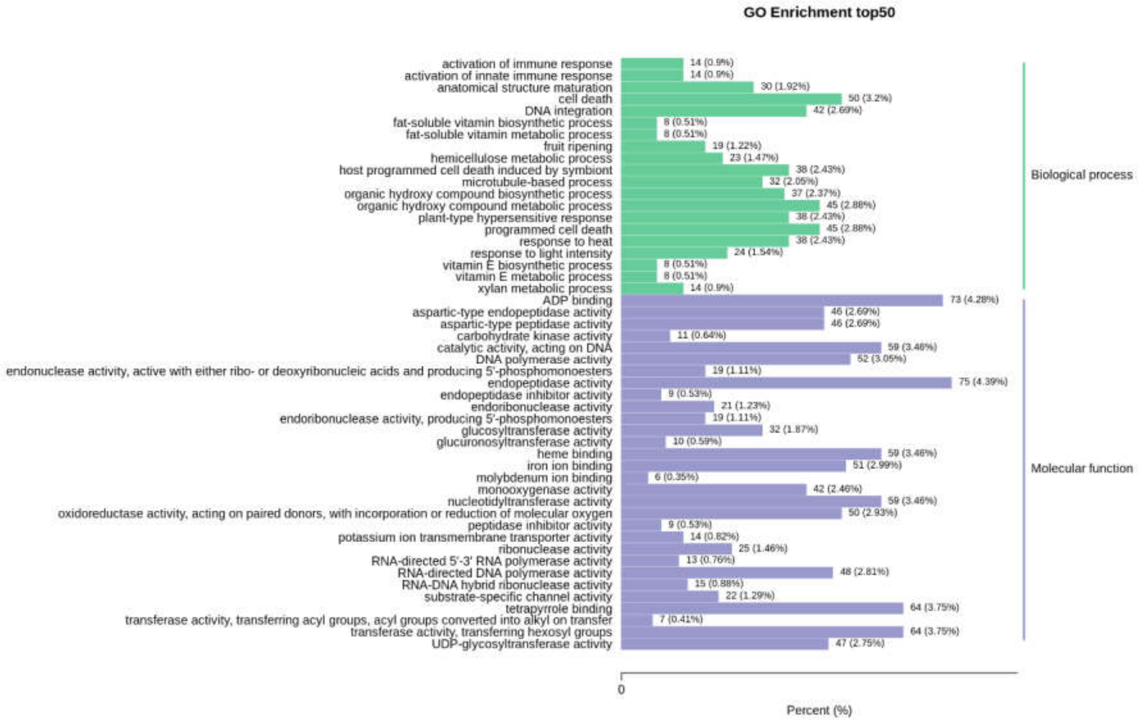

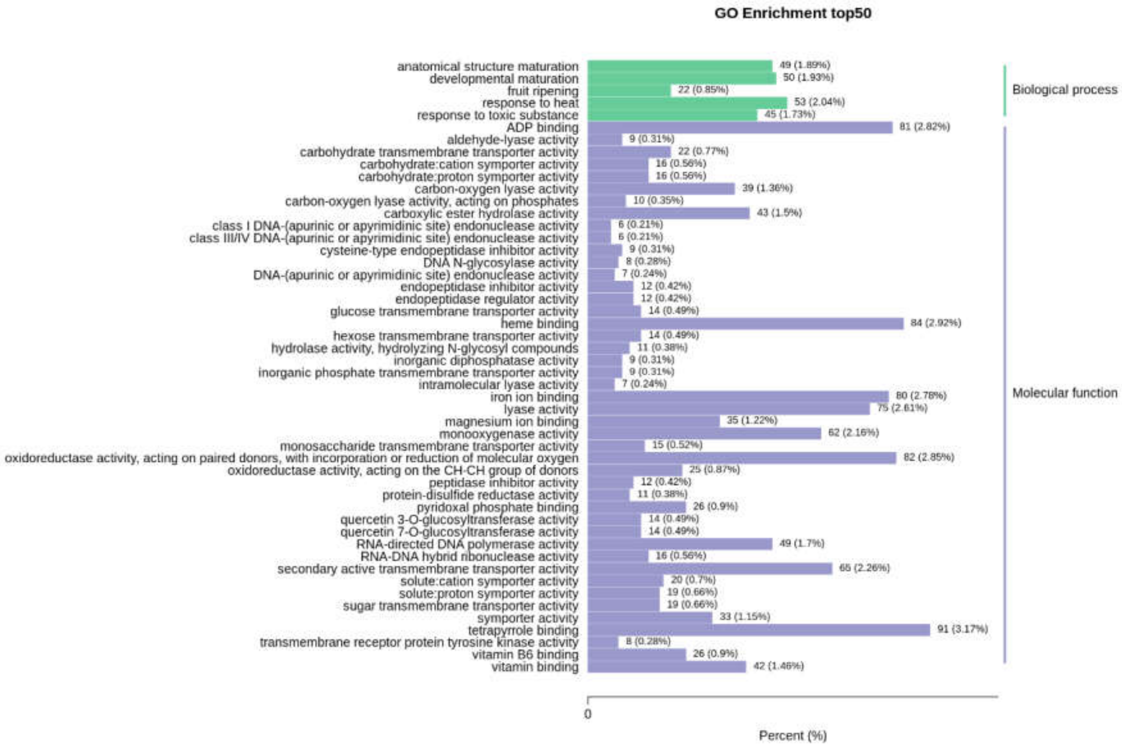

Figure S5: GO enrichment analysis of differentially expressed genes

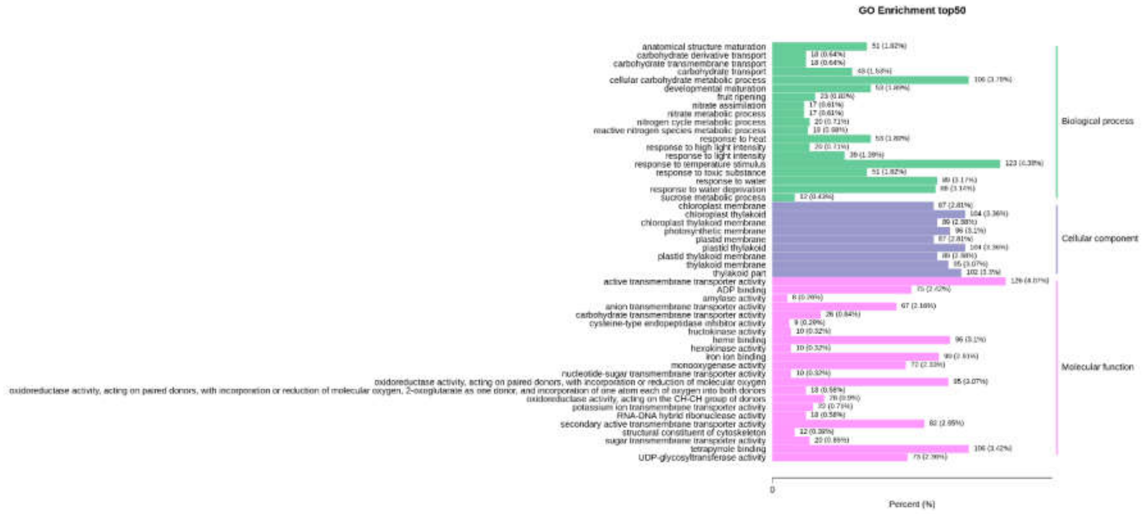

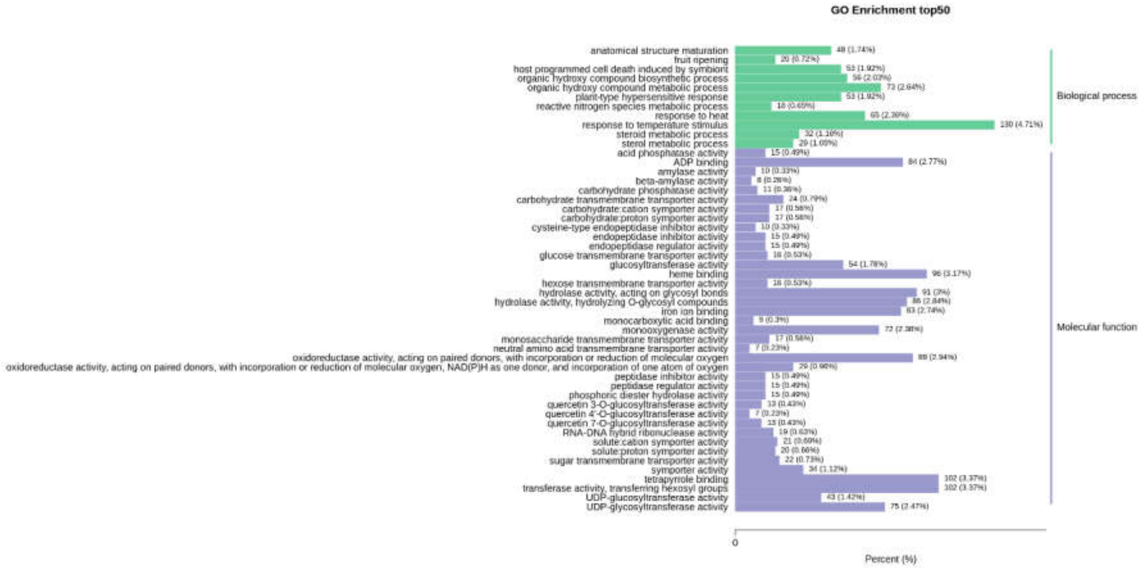

Figure S5: GO enrichment analysis of differentially expressed genes

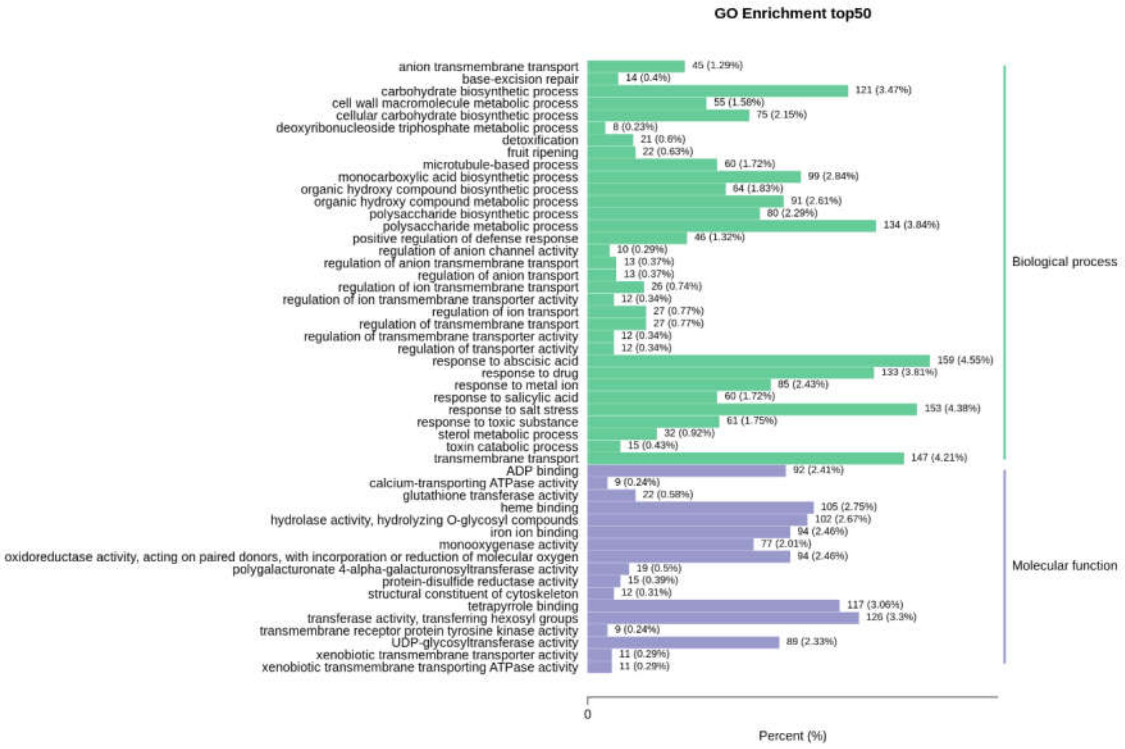

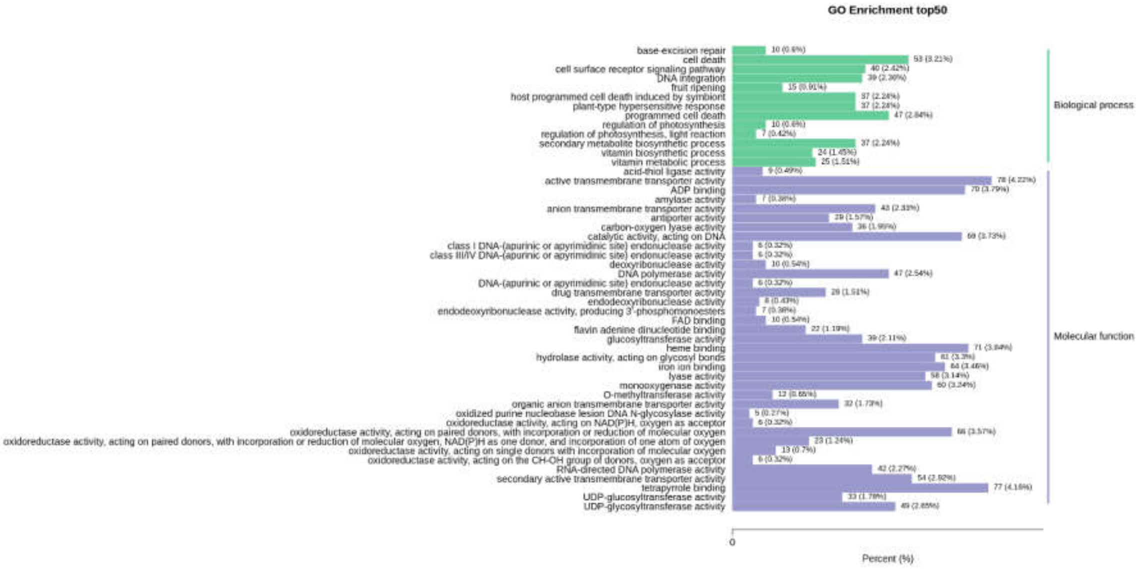

Figure S5: GO enrichment analysis of differentially expressed genes

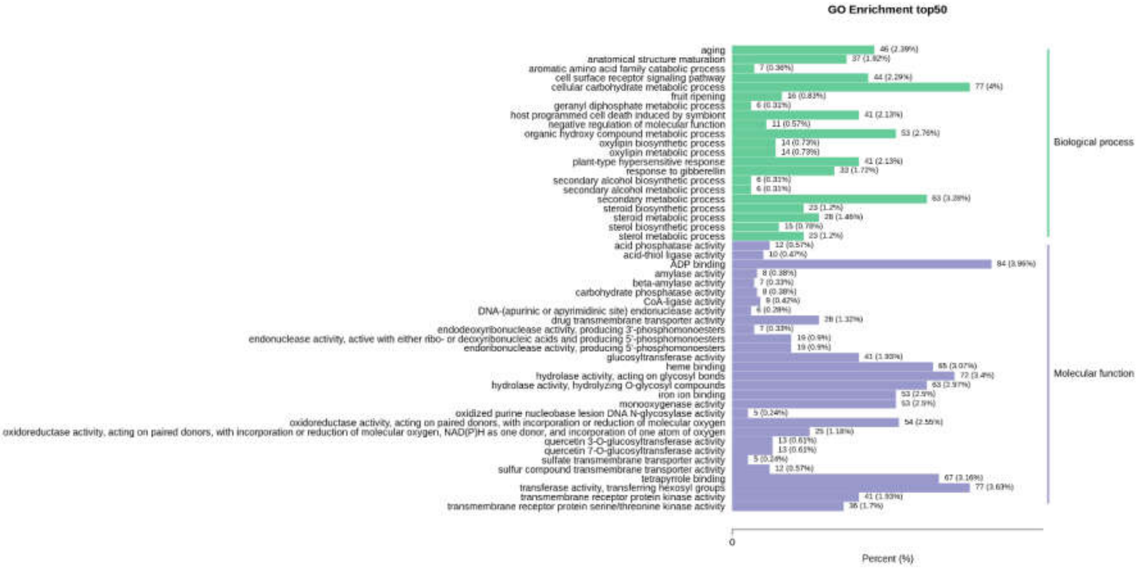

Supplement: Supplementary file 1 [file ijms-24-07199-s001.zip › ijms-2168485-supplementary/Supplementary Files/Figure S5.pdf]
